# Long‐term changes in autumn–winter harvest distributions vary among duck species, months, and subpopulations

**DOI:** 10.1002/ece3.11331

**Published:** 2024-06-03

**Authors:** Bram H. F. Verheijen, Elisabeth B. Webb, Michael G. Brasher, Heath M. Hagy

**Affiliations:** ^1^ Missouri Cooperative Fish and Wildlife Research Unit, School of Natural Resources University of Missouri Columbia Missouri USA; ^2^ U.S. Geological Survey, Missouri Cooperative Fish and Wildlife Research Unit, School of Natural Resources University of Missouri Columbia Missouri USA; ^3^ Ducks Unlimited, Inc. Memphis Tennessee USA; ^4^ U.S. Fish and Wildlife Service, Habitat and Population Evaluation Team Bismarck North Dakota USA

**Keywords:** Anatidae, autumn, band recovery, distribution, duck, harvest, nonbreeding, subpopulations, waterfowl, winter

## Abstract

Our aim was to describe shifts in autumn and winter harvest distributions of three species of dabbling ducks (blue‐winged teal [*Spatula discors*], mallard [*Anas platyrhynchos*], and northern pintail [*Anas acuta*]) in the Central and Mississippi flyways of North America during 1960–2019. We measured shifts in band recovery distributions corrected for changes in hunting season dates and zones by using kernel density estimators to calculate 10 distributional metrics. We then assessed interannual and intraspecific variation by comparing species‐specific changes in distributional metrics for 4 months (October–January) and three geographically based subpopulations. During 1960–2019, band recovery distributions shifted west‐ and southwards (blue‐winged teal) or east‐ and northwards (mallard and northern pintail) by one hundred to several hundred kilometers. For all three species, the broad (95% isopleth) and core distributions (50% isopleth) showed widespread decreases in overlap and increases in relative area compared to a 1960–1979 baseline period. Shifts in band recovery distributions varied by month, with southward shifts for blue‐winged teal most pronounced in October and northward shifts for mallard and northern pintail greatest during December and January. Finally, distributional metric response varied considerably among mallard subpopulations, including 2–4‐fold differences in longitude, latitude, and overlap, whereas differences among subpopulations were minimal for blue‐winged teal and northern pintail. Our findings support the popular notion that winter (December–January) distributions of duck species have shifted north; however, the extent and direction of distributional changes vary among species and subpopulations. Long‐term distributional changes are therefore complex and summarizing shifts across species, months, or subpopulations could mask underlying finer‐scale patterns that are important to habitat conservation and population management. A detailed understanding of how species distributions have changed over time will help quantify important drivers of species occurrence, identify habitat management options, and could inform decisions on where to focus conservation or restoration efforts.

## Graphical abstract

Maps of 95% isopleths of utilization distributions of blue‐winged teal, mallard, and northern pintail bands recovered in the Central and Mississippi flyways of North America between 1960 and 2019 for the banding region and month in which species‐specific shifts were greatest. Shown are isopleths (polygons) and centroid locations (points) of bands recovered during 1960–1969 (gray/black) or 2010–2019 (red).

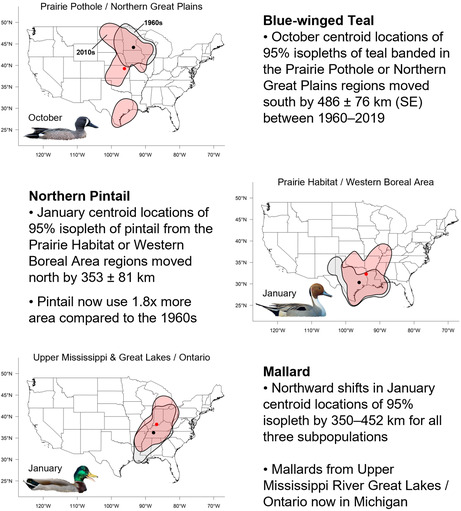

## 1 INTRODUCTION

Shifts in the distributions of wildlife can have implications for ecosystem functioning and conservation efforts. Distributional shifts are caused by a variety of natural and anthropogenic factors, including changes in habitat quality, quantity, and juxtaposition (de Moreas et al., 2020; Studds et al., 2016), climate and weather (Hickling et al., 2006; Horton et al., 2020; La Sorte & Thompson III, 2007), predator and prey communities (Myers et al., 2007; Stantial et al., 2021), introduction of species into novel environments (Elliott & Arbib Jr., 1953), diseases or parasites (Anagnostakis, 2001; Valenta et al., 2016), pesticide use (raptors; Rosenberg et al., 2019), and human disturbance (Burger & Niles, 2013; Gallego‐Zamorano et al., 2020; Kays et al., 2016). Because of the variety of underlying causes, distributional shifts may be of different magnitudes and occur in multiple directions depending on environmental drivers and the ecology of affected species. For example, many species are shifting their distributions toward the poles or higher elevations in response to a warming climate (although often at variable rates), whereas distributions of other species remain unchanged or are moving in unexpected directions, thereby creating a range of management and conservation challenges (Donelson et al., 2019; Horton et al., 2020; La Sorte & Thompson III, 2007; Lenoir & Svenning, 2015; Rubenstein et al., 2023; Tayleur et al., 2015).

In addition to interspecific variation, distributional shifts may also vary among subpopulations within species. Intraspecific variation in distributional shifts is likely because species are often affected by landscape‐level changes that occur in only parts of their range (Barlow et al., 2016; Masden et al., 2009). In contrast to non‐migratory species, migratory species introduce the additional complexity of individuals using multiple landscapes throughout their annual cycle, including breeding, nonbreeding and migratory stopover sites. Subpopulations of migratory species can be partially or completely isolated from one another because of fidelity to discrete migratory routes, as seen in some avian (e.g., mallards (Roberts et al., 2022), sea ducks (Lamb et al., 2019), purple martins (Fraser et al., 2013) and golden‐winged warblers (Kramer et al., 2017)) and non‐avian taxa (e.g., mule deer and pronghorn (Collins, 2016; Sawyer et al., 2005, 2016)). Consequently, migratory species are often affected by drivers that operate at greater spatial scales and multiple discrete locations compared to non‐migratory species, increasing the potential for intraspecific variation in distributional shifts. Moreover, variation in changes in demographic rates among breeding regions could affect distributions during other parts of the life cycle (i.e., migration, nonbreeding) through carryover effects. For species with great intraspecific variation in distributional shifts, measuring the extent and direction of shifts for only part of the population might therefore not be representative for the entire species.

Understanding shifts in geographic distributions is especially important for waterfowl during autumn and winter. Waterfowl in North America are the focus of significant investments in habitat conservation and are among the most heavily harvested and managed groups of animals on the continent. Effective decisions regarding their conservation and management require a thorough understanding of spatiotemporal patterns of distributions and population dynamics (Alisauskas et al., 2013; Roberts et al., 2022; Roy et al., 2017; USFWS, 2022). During autumn and winter, waterfowl distributions are determined by an innate migration response as well as environmental drivers such as weather conditions, the number, distribution, and diversity of available wetlands on the landscape, and anthropogenic disturbance (e.g., hunting; Åkesson & Helm, 2020; Beatty et al., 2014; Hagy et al., 2014; Haugen et al., 2014; Pearse et al., 2012). Although, inherent spatiotemporal variation in these drivers causes waterfowl distributions to vary substantially from year to year (Haugen et al., 2014; Masto et al., 2022; Schummer et al., 2010; Weller et al., 2022), the combined effects of directional change in weather conditions, availability and hydrology of wetlands, agricultural and urban land use, and restoration of historically altered habitat can result in more persistent shifts in waterfowl distributions (Dale, 1997; Duncan et al., 1999; Guillemain, Champagnon et al., 2015; Guillemain, Pernollet et al., 2015; King et al., 2006; McKenna et al., 2017; NOAA, 2023).

Because of key differences in morphology and life‐history traits among species (Baldassarre & Bolen, 2006), waterfowl can exhibit substantial levels of interspecific variation in distributional response to changing environmental conditions (Guillemain, Champagnon et al., 2015; Guillemain, Pernollet et al., 2015; Masto et al., 2022; Schummer et al., 2017). Winter distributions of some waterfowl species have shifted northwards (Brook et al., 2009; Cox et al., 2023; Gunnarsson et al., 2012; Lehikoinen et al., 2013; Meehan et al., 2021; Sauter et al., 2010; Švažas et al., 2001; Verheijen et al., 2024) or are predicted to do so (Notaro et al., 2016; Reese & Skagen, 2017), while southward shifts or lack of distributional change have been documented for other species and regions (Cox et al., 2023; Green & Krementz, 2008; Guillemain, Champagnon et al., 2015; Guillemain, Pernollet et al., 2015; Verheijen et al., 2024). However, whether and to what extent distributional shifts vary within species is less understood. Many duck species in North America show relatively low breeding site fidelity in adult males and first‐year birds (Anderson et al., 1992; Doherty Jr. et al., 2002; Greenwood & Harvey, 1982; Toay et al., 2019) and regular inter‐flyway movements between breeding and wintering grounds, although at low rates (Roberts et al., 2022). On the other hand, the longitude of the winter location of some species is partially influenced by the regional association during the breeding season (Cox et al., 2023; Munro & Kimball, 1982; Roberts et al., 2022; Szymanski & Dubovsky, 2013; Verheijen et al., 2024). In addition, the increased prevalence of game‐farm mallards and game‐farm × wild mallard hybrids—who both might lack an innate behavior to migrate—in the Mississippi flyway might result in variation in migratory response of mid‐continental mallards across a longitudinal gradient (Schummer et al., 2023). Subpopulations of breeding ducks may therefore spend winter in separate locations and be exposed to different environmental conditions, whereas mallard populations also vary in the relative abundance of game‐farm mallard genes; all factors that could lead to intraspecific variation in shifts in duck distributions during autumn and winter.

Here, we describe and quantify distributional shifts in autumn and winter for three species of dabbling ducks (blue‐winged teal [*Spatula discors*], mallard [*Anas platyrhynchos*], and northern pintail [*Anas acuta*]) in the Central and Mississippi flyways of North America during 1960–2019. Together, the Central and Mississippi flyways encompass the majority of the autumn and winter range of mid‐continent waterfowl and annually account for >60% of the total U.S. duck harvest (Raftovich et al., 2022). Inclusion of both flyways allows for the evaluation of potential longitudinal shifts in distributions across this shared region. While correcting for changes in hunting season dates and zones through time, we measured changes in band recovery distributions using kernel density estimators to calculate 10 distributional metrics. We assessed intra‐annual and intraspecific variation in distributional shifts by comparing species‐specific changes in distributional metrics across 4 months (October–January) and three geographically defined subpopulations in the USA and Canada. A detailed understanding of how species distributions have changed over time could help identify important drivers of species occurrence (e.g., resource selection analysis), inform habitat management, enable more effective communication with waterfowl hunters and non‐consumptive recreationists, and aid decision making on where to focus conservation efforts.

## 2 METHODS

### 2.1 Study area

To promote collaborative management and conservation of migratory birds and their habitat, the U.S. Fish and Wildlife Service (USFWS) partners with state agencies and other organizations to coordinate the management of waterfowl populations within four administrative flyways (Atlantic, Mississippi, Central, and Pacific) based on prevailing north–south bird migration routes. The Central and Mississippi flyways encompass the central United States from the Continental Divide in the west to Ohio in the east, the Canadian provinces of Alberta, Saskatchewan, Manitoba, Ontario, and Northwest Territories to the north, and large portions of Mexico in the south. For our analysis, we included band recovery data from 10 U.S. states in the central flyway (Colorado, Kansas, Montana, Nebraska, New Mexico, North Dakota, Oklahoma, South Dakota, Texas, and Wyoming) and 14 U.S. states in the Mississippi flyway (Alabama, Arkansas, Illinois, Indiana, Iowa, Kentucky, Louisiana, Michigan, Minnesota, Mississippi, Missouri, Ohio, Tennessee, and Wisconsin).

### 2.2 Band recovery data

**FIGURE 1:**
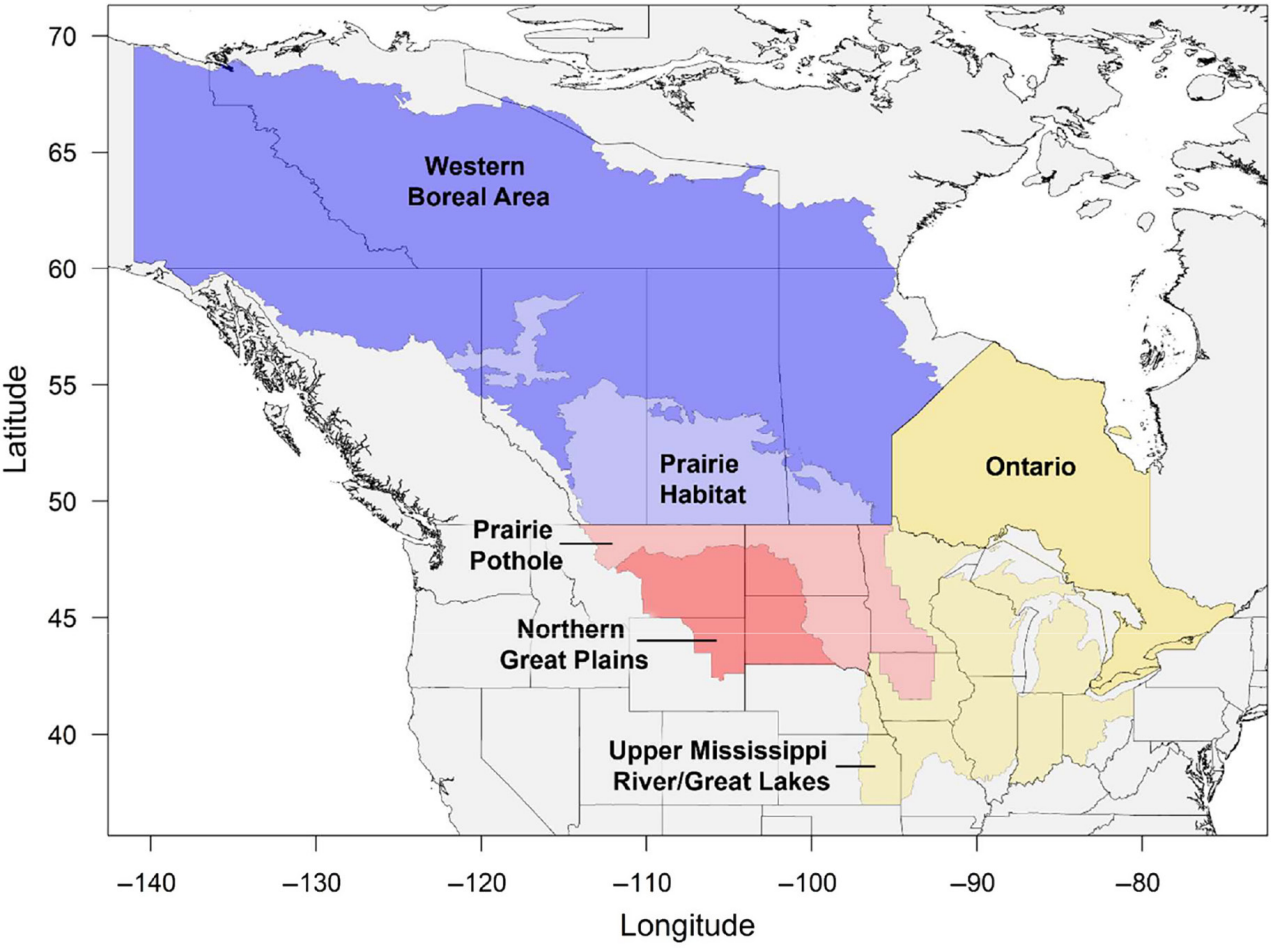
Our analysis includes band recoveries of blue‐winged teal, mallard, and northern pintail collected throughout the Central and Mississippi flyways of North America between 1960 and 2019 from ducks banded in one of three geographically defined regions in which duck banding occurs: (1) the Prairie Habitat and Western Boreal Area Joint Ventures (PHWB; blue), (2) the Prairie Pothole and Northern Great Plains Joint Ventures (PPNP; red), and (3) the Upper Mississippi River/Great Lakes Joint Venture and Ontario (UMON; yellow).

Our analysis included three species of migratory dabbling ducks that are among the most commonly leg‐banded and harvested species throughout the Central and Mississippi flyways of North America: blue‐winged teal, mallard, and northern pintail. For each species, we obtained band recovery data for birds recovered within the U.S. portion of the Central or Mississippi flyway from the U.S. Geological Survey (USGS) Bird Banding Laboratory (BBL; pwrc.usgs.gov/BBL/Bander_Portal, Accessed 21 September 2021). Location data from annual band recoveries can serve as useful indices of waterfowl distributions when viewed across large spatial and temporal scales (e.g., Cox et al., 2023; Green & Krementz, 2008; Verheijen et al., 2024). To test for differences in distributional change over time among sub‐populations of ducks, we partitioned band recovery data into three geographically defined groups corresponding to broad regions where banding occurred (hereafter “banding regions”). We used geographies of several Migratory Bird Joint Ventures of the North American Waterfowl Management Plan (NAWMP) to define the following banding regions for our study: the Prairie Habitat and Western Boreal area (hereafter “PHWB”), the Prairie Pothole and Northern Great Plains area (hereafter “PPNP”), and the Upper Mississippi/Great Lakes and Ontario area (hereafter “UMON”; Figure 1). Although we considered other politically or ecologically based delineations, we ultimately chose combinations of joint venture boundaries to increase the relevancy of our results to conservation interests and because these regions corresponded generally to areas important to other aspects of waterfowl management (e.g., management of breeding and migratory stopover habitats).

We included direct and indirect band recoveries of birds that were either shot (90.14% of all recoveries) or found dead (1.56% of all recoveries) during the hunting seasons (October–January) of 1960–2019. We excluded band recoveries from September, because of analytical challenges caused by spatially inconsistent sampling efforts resulting from the introduction of an early (i.e., September) teal hunting season in the late 1960s and early 1970s. Excluding bands recovered in September lowered the sample size by 6.20% (of which 72.74% were from blue‐winged teal). Most band recoveries were reported with a spatial accuracy of a 10‐min latitude block or finer (~14 × 19 km), but we excluded a small percentage of bands with recovery locations reported at coarser spatial scales (0.25% of all recoveries). We included only recoveries from birds banded during the preseason banding period (i.e., July–September, when birds finished breeding but had not yet started fall migration; 77.02% of all recoveries). We excluded band recoveries from ducks banded during other times of the year because of potential differences in survival probability and other uncertain effects on recovery distributions (Devers et al., 2021). To avoid potential effects that banding efforts may have on migration behavior and resulting winter distributions, we further restricted our dataset to include only birds that were held <24 h during banding and were released within the same 10‐min block from which they were captured (94.15% of all recoveries). We pooled data across sex and age classes because of low sample sizes for some combinations of species, month, and banding region. Finally, many drivers of hunter distributions in North America—including hunting season dates and zones—have seen large changes since the 1960s (Verheijen et al., 2024). Moreover, we acknowledge that some ducks persist at latitudes after hunting seasons close and are therefore unavailable to be harvested. To account for differences in the availability of bands for harvest caused by spatiotemporal variation in hunting season length among hunting zones, we weighted recovery locations by a “hunting correction factor” previously described in Verheijen et al. (2024). In brief, we delineated hunting zones using descriptive information from state agencies and the Federal Register (https://www.federalregister.gov, Accessed 23 February 2022), obtained zone‐ and year‐specific hunting season dates from the Federal Register, and calculated the number of days that a given zone was open for hunting for each month of interest (October–January) in a given year. We then used the inverse of the proportion of days that the hunting zone was open during the month of recovery as a weighting factor for individual recoveries in our statistical models (Verheijen et al., 2024).

### 2.3 Estimating band recovery utilization distributions

For each species, month, banding region, and year combination with at least 30 unique band recovery locations, we used kernel density estimators to estimate the 50%, 70%, 90%, and 95% isopleths of band recovery utilization distributions on a 1000 × 1000‐cell grid with the “ks” package in R (Duong, 2022; R Core Team, 2023). We chose these four isopleth levels because they represent the core area (50% isopleth), two intermediate segments, and the broad range (95%) of the estimated utilization distribution. Previous work has shown that 20–30 unique locations can provide an unbiased estimate of space use, while 50% and 95% isopleths provide unbiased estimates of the core area and overall space use, respectively, with the latter excluding only locations from rarely used areas (Fletcher Jr. & Koford, 2003; Seaman et al., 1999; Seaman & Powell, 1996; Worton, 1989). When using kernel density estimation, the selection of an appropriate smoothing parameter is especially important, because smoothing restricts the distance at which individual recovery locations influence the surface grid (Fieberg, 2007; Hemson et al., 2005; Leonard et al., 2008; Silverman, 1986). During preliminary analyses, least squares cross‐validation (Hlscv) techniques regularly failed to converge because of duplicate recovery locations, while visual assessment revealed that a normal‐scale bandwidth (Hns) or smoothed cross‐validation bandwidth (Hscv) were too large and led to considerable oversmoothing. We therefore used the plug‐in bandwidth selector (Hpi; default option) to estimate the smoothing parameter for each species, banding region, month, year combination (Duong, 2022). We then re‐estimated utilization distributions for each combination of species, banding region, month, and year by manually setting the smoothing parameter to the mean of all plug‐in bandwidth selectors estimated during the previous step to improve comparability of results.

### 2.4 Calculating distribution metrics

Many studies that quantify shifts in geographical distributions have focused on changes in central location and range extent (i.e., leading and trailing edge) along a north–south gradient, especially when evaluating the effects of climate change (Donelson et al., 2019; Lenoir & Svenning, 2015; Rubenstein et al., 2023). However, the shape of species distributions is often complex, as they are regulated by multiple interacting drivers and encompass heterogeneous landscapes. Distributional shifts might therefore not be fully captured by only one or two parameters (Cox et al., 2023). We therefore evaluated changes in the geographical distribution of band recoveries over time by using estimated utilization distributions to calculate 10 metrics that characterize different aspects of distributional change for each combination of species, banding region, month, and year, and for all four isopleth levels of interest using the “sf” package and base functions in R (Pebesma, 2018; R Core Team, 2023).

First, we estimated the mean shifts of distributions by calculating the centroid longitude (1) and latitude (2) of each isopleth in degrees. Second, we calculated the northern (3), southern (4), eastern (5), and western extent (6) in degrees to estimate whether boundaries of recovery distributions have shifted in certain directions independent of mean distributional shifts. Third, to assess changes in area over which bands have been recovered through time, we calculated the relative area (7) of each isopleth compared to the average isopleth area of the years within a baseline period (1960–1979) for the same species, banding region, and month. Using a 1960–1979 baseline allowed us to make use of the earliest years with sufficient band recoveries for analyses and is less biased than comparing metrics from years of interest to a single year (e.g., 1960) because of the substantial annual variation in duck winter distributions (Haugen et al., 2014; Masto et al., 2022; Schummer et al., 2010). Estimates of the absolute area of isopleths can be biased because they strongly depend on the chosen smoothing parameter (Worton, 1989). Instead, we calculated the relative area by comparing the area of two isopleths (year of interest vs the average of the baseline period) which were both estimated with the same smoothing parameter using the following equation:

$$\frac{A_{t}}{\left(\sum_{i=1960}^{i=1979}A_{i}\right)/20}$$

where *t* = 1960 to 2019. Finally, we calculated three metrics of overlap with the 1960–1979 baseline period that form a gradient for assessing how the overall recovery area has changed through time (Fieberg & Kochanny, 2005). Specifically, we compared distributional overlap between each year of interest and each of the 20 years within the baseline period (or 19 years if the year of interest fell within the baseline period to avoid comparing years to themselves). We then averaged these overlap estimates to obtain a single overlap metric for each year of interest. The simplest overlap metric considers only the extent to which total area of year‐specific isopleths overlap each year within the baseline period (8; hereafter “HR” to match the notation in Fieberg & Kochanny, 2005). As an alternative, we considered an overlap metric that accounted for the underlying probability density function of each kernel density estimate (9; hereafter “PHR”), effectively estimating how much of the total volume of year‐specific isopleths overlap each year within the baseline period. Finally, we calculated the Bhattacharyya's affinity (10; hereafter “BA”), which is a function of the product of two utilization distributions under the assumption that both distributions are independent of each other (Bhattacharyya, 1943). Effectively, the BA metric determines the similarity between two distributions, considering both non‐overlapping areas and differences in probability densities in overlapping areas. A BA score of zero indicates no overlap whereas a score of one indicates identical utilization distributions (see Table S1 or Fieberg & Kochanny, 2005, for specific calculations).

### 2.5 Correlations among distribution metrics

During preliminary analysis, we found strong correlations among many of the 10 distribution‐metrics of interest for all three species (Figure S1). Within isopleth levels, centroid longitude was generally positively correlated with the western and eastern extent of distributions (blue‐winged teal: mean = 0.50, range = −0.12 to 0.88; mallard: mean = 0.77, range = 0.65 to 0.84; northern pintail: mean = 0.56, range = 0.25 to 0.81), centroid latitude was strongly correlated with northern and southern extent (blue‐winged teal: mean = 0.86, range = 0.78 to 0.92; mallard: mean = 0.83, range = 0.75 to 0.90; northern pintail: mean = 0.82, range = 0.76 to 0.90), and overlap metrics of interest for all three species were strongly positively correlated with each other (blue‐winged teal: mean = 0.74, range = 0.42 to 1.00; mallard: mean = 0.90, range = 0.81 to 0.99; northern pintail: mean = 0.92, range = 0.81 to 1.00). Because of strong correlations among metrics, we limited our analysis to the following four metrics: centroid longitude, centroid latitude, relative area, and Bhattacharyya's affinity (as an index of overlap). Finally, these four remaining metrics of interest were strongly positively correlated across isopleth levels for all three species (blue‐winged teal: mean = 0.90, range = 0.61 to 1.00; mallard: mean = 0.93, range = 0.83 to 1.00; northern pintail: mean = 0.91, range = 0.76 to 1.00). Because correlations between metrics were weakest for the 50% and 95% isopleths, we restricted analyses to those isopleth levels.

### 2.6 Statistical analysis

**FIGURE 2:**
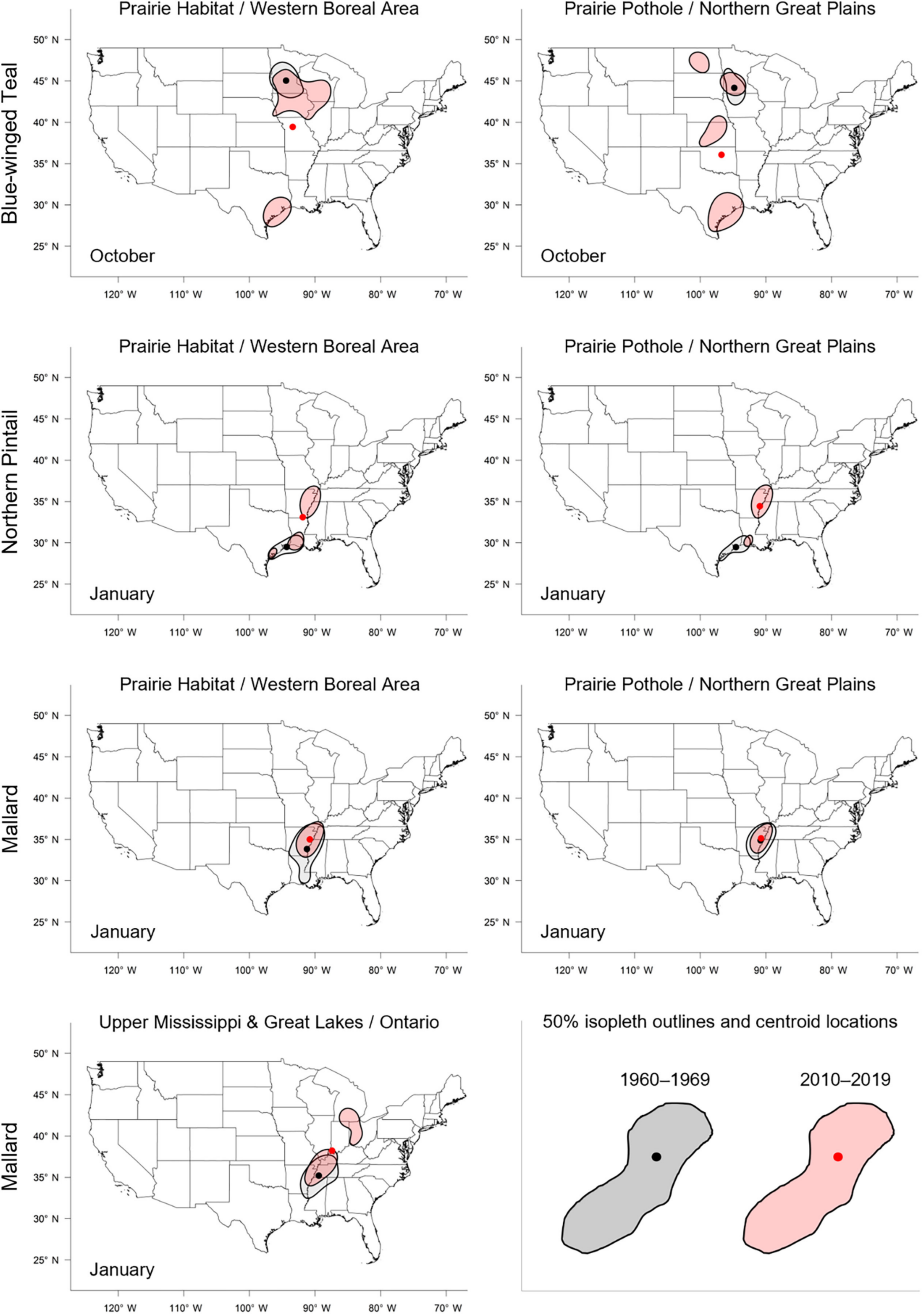
Maps of 50% isopleths of utilization distributions of blue‐winged teal, mallards, and northern pintail bands recovered in the Central and Mississippi flyways of North America between 1960 and 2019 for each banding region and for the month in which species‐specific shifts were greatest (October for blue‐winged teal and January for mallard and northern pintail). Shown are isopleths (polygons) and centroid locations (points) of bands recovered during 1960–1969 (gray/black) or 2010–2019 (red). Geographically defined banding regions include the Prairie Habitat and Western Boreal Area Joint Ventures (PHWB), the Prairie Pothole and Northern Great Plains Joint Ventures (PPNP), and the Upper Mississippi River/Great Lakes Joint Venture and Ontario (UMON; mallard only).

**FIGURE 3:**
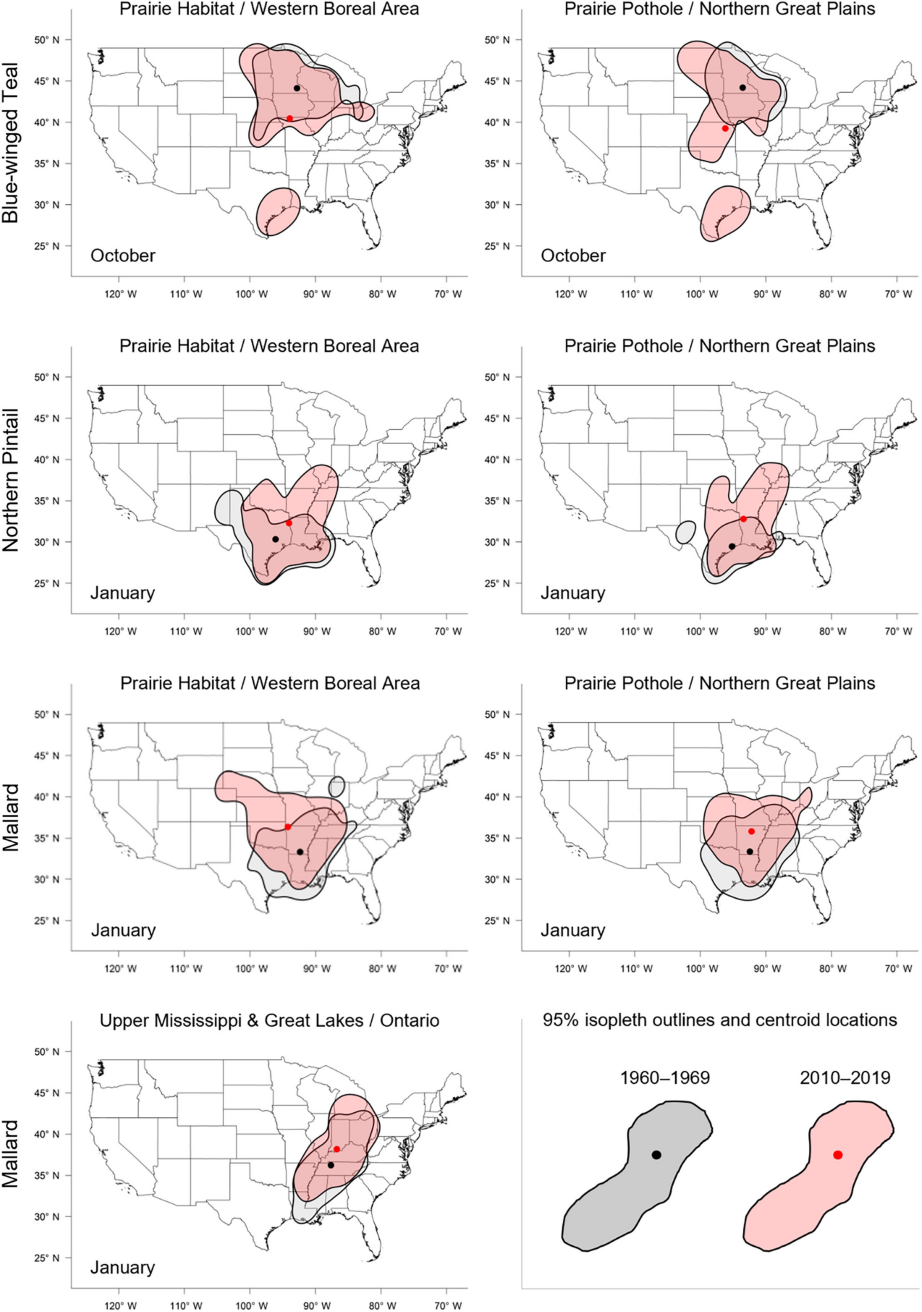
Maps of 95% isopleths of utilization distributions of blue‐winged teal, mallards, and northern pintail bands recovered in the Central and Mississippi flyways of North America between 1960 and 2019 for each banding region and for the month in which species‐specific shifts were greatest (October for blue‐winged teal and January for mallard and northern pintail). Shown are isopleths (polygons) and centroid locations (points) of bands recovered during 1960–1969 (gray/black) or 2010–2019 (red). Geographically defined banding regions include the Prairie Habitat and Western Boreal Area Joint Ventures (PHWB), the Prairie Pothole and Northern Great Plains Joint Ventures (PPNP), and the Upper Mississippi River/Great Lakes Joint Venture and Ontario (UMON; mallard only).

For each species, we used a separate set of generalized linear regression models to test whether distribution metrics of interest changed between 1960 and 2019 and whether temporal effects were month‐ or banding region‐specific (Kutner et al., 2004). We assumed a normal distribution when modeling changes in latitude, longitude, and relative area, and a beta distribution for changes in relative overlap, because Bhattacharyya's affinity values only range from 0 to 1. The global model for each metric included fixed effects of recovery year (continuous), recovery month (October–January; categorical), banding region (PHWB, PPNP, or UMON; categorical), two‐way interactions between year and month, year and banding region, and month and banding region, and a three‐way interaction between all variables. In our model sets, we considered an intercept‐only model, the global model, and all possible sub‐models of the global model (see Tables S3–S10 for model structures). To test whether the effects of banding region on recovery location were unique to each region or were instead gradual along a continuum of banding latitude or longitude, we added a series of similar models to our model sets in which we included continuous effects of either banding longitude or latitude rather than a categorical effect of banding region. We tested for correlations among explanatory variables and did not include models containing correlated variables (*r* > .5) in model sets. We visually examined quantile‐quantile and residual plots for normality and homoscedasticity in the residuals of the global models for each combination of species, banding region, and month. To improve model fit, we modeled the natural logarithm of relative area, but present back‐transformed results. To determine which combination of variables best‐explained variation in each distribution metric, we compared AIC_c_‐values of all models (Burnham & Anderson, 2002) and used model averaging based on AIC_c_‐weights if multiple models were considered equally parsimonious (ΔAIC_c_ ≤ 2). We considered beta coefficients to be significant if their 95% confidence interval did not overlap zero. Finally, to visualize distributional changes over time, we constructed distribution maps of 50% and 95% isopleths and their centroid locations for each species, banding region, and month combination based on band recoveries during 1960–1969 or 2010–2019 (Figures 2 and 3, Figure S2). All statistical analyses and visualizations were conducted with base functions in R (R Core Team, 2023).

## 3 RESULTS

### 3.1 Sample sizes

Our analysis included band recoveries from blue‐winged teal (*n* = 13,768), mallard (*n* = 325,512), and northern pintail (*n* = 15,674) collected during 1960–2019 (Table S2). Spatiotemporal variation in band recoveries resulted in 189, 700, and 204 year, month, banding region combinations (of a possible 720) with at least 30 band recoveries for blue‐winged teal, mallard, and northern pintail, respectively (Table 1). We excluded blue‐winged teal and northern pintail band recoveries from the UMON banding region from our analysis because of the limited number of years with ≥30 recoveries. For the same reason, we do not present blue‐winged teal data from December and January, or northern pintail data from October (except for changes in centroid latitude and longitude of band recoveries from the PPNP banding region).

**TABLE 1:** The number of years during 1960–2019 with at least 30 band recoveries for blue‐winged teal, mallard, and northern pintail recovered in the Central or Mississippi flyways for each banding region and month of recovery (October–January).

Species	Month	PHWB	PPNP	UMON
Blue‐winged teal	Oct	40	27	15
	Nov	41	21	0
	Dec	21	7	0
	Jan	12	5	0
Mallard	Oct	60	60	60
	Nov	60	60	60
	Dec	60	60	60
	Jan	53	53	54
Northern pintail	Oct	2	19	0
	Nov	39	17	0
	Dec	48	24	0
	Jan	33	22	0

*Note*: Banding regions include the Prairie Habitat and Western Boreal Area Joint Venture (PHWB), the Prairie Pothole and Northern Great Plains Joint Venture (PPNP), and the Upper Mississippi River/Great Lakes Joint Venture and Ontario (UMON) geographies.

### 3.2 Patterns in distribution metrics

**FIGURE 4:**
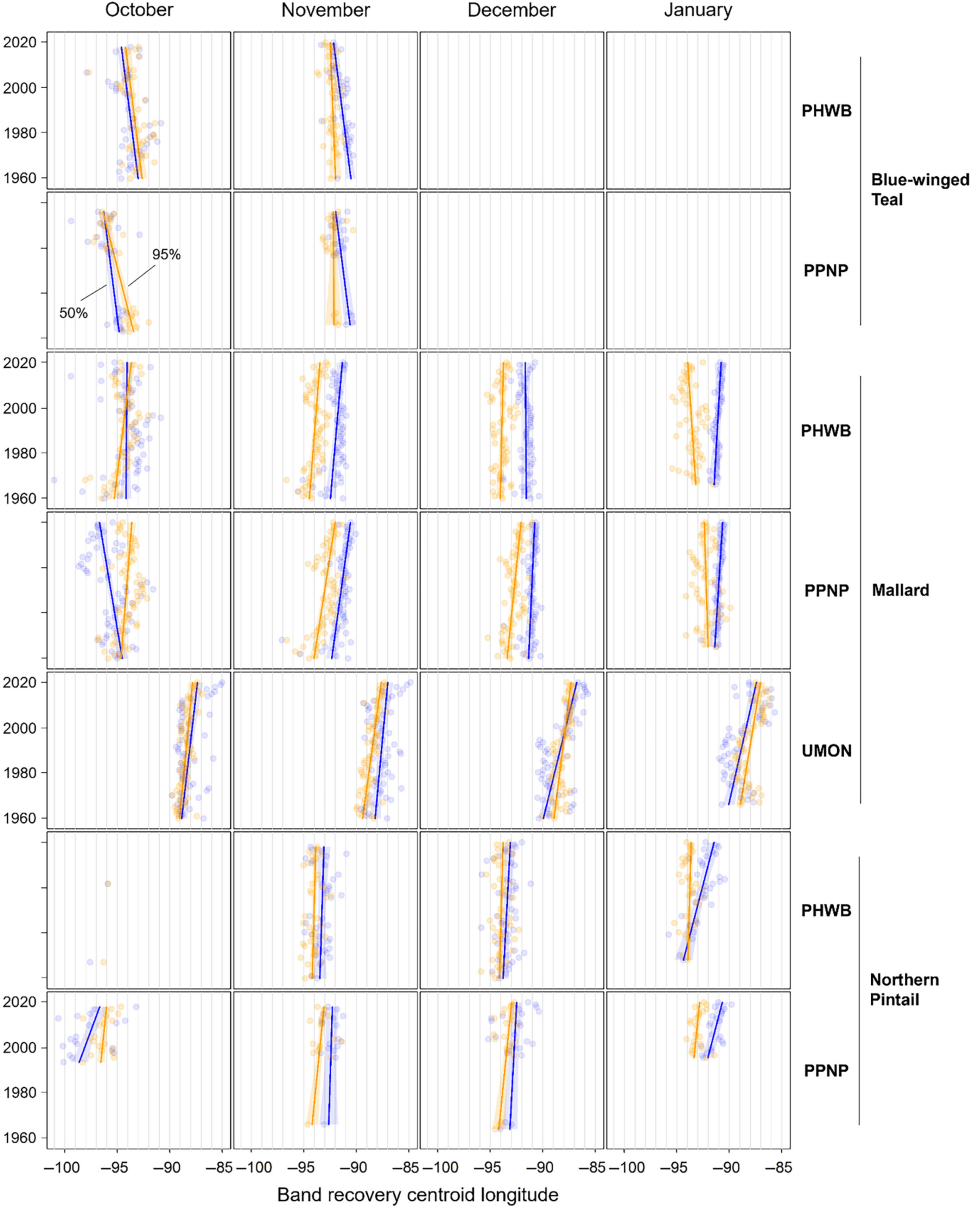
Changes in centroid longitude of band recoveries from blue‐winged teal, mallards, and Northern Pintail in the Central and Mississippi flyways of North America between 1960 and 2019. Shown are the centroid longitude of the 50% (blue) and 95% (orange) isopleths of kernel density estimator‐based utilization distributions for each combination of year, month of recovery (October–January), and banding region with 30 or more band recoveries. Banding regions include the Prairie Habitat and Western Boreal Area Joint Ventures (PHWB), the Prairie Pothole and Northern Great Plains Joint Ventures (PPNP), and the Upper Mississippi River/Great Lakes Joint Venture and Ontario (UMON).

**FIGURE 5:**
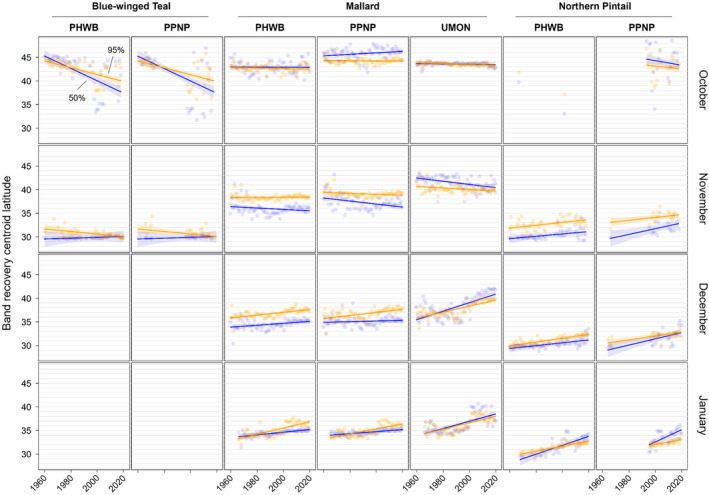
Changes in centroid latitude of band recoveries from blue‐winged teal, mallards, and Northern Pintail in the Central and Mississippi flyways of North America between 1960 and 2019. Shown are the centroid latitude of the 50% (blue) and 95% (orange) isopleths of kernel density estimator‐based utilization distributions for each combination of year, month of recovery (October–January), and banding region with 30 or more band recoveries. See caption of Figure 2 for full names of banding regions.

**FIGURE 6:**
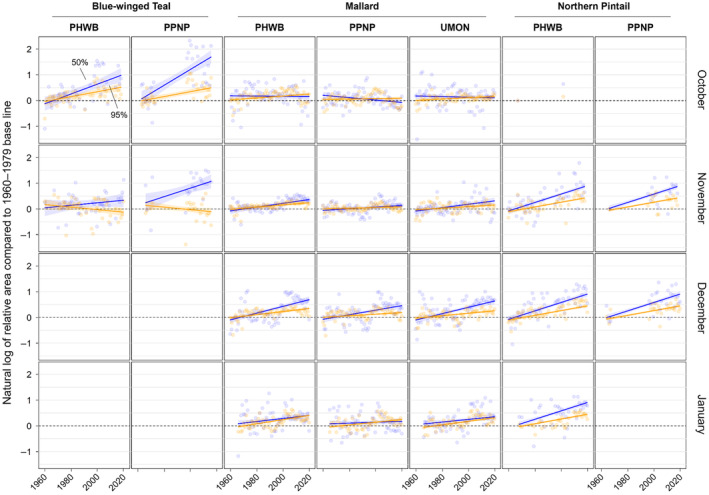
Changes in the relative area of band recoveries from blue‐winged teal, mallards, and Northern Pintail in the Central and Mississippi flyways of North America for each year between 1960 and 2019 compared to a historical base line (1960–1979). Shown are the relative areas for the 50% (blue) and 95% (orange) isopleths of kernel density estimator‐based utilization distributions on a natural logarithmic scale for each combination of year, month of recovery (October–January), and banding region with 30 or more band recoveries. See caption of Figure 2 for full names of banding regions.

**FIGURE 7:**
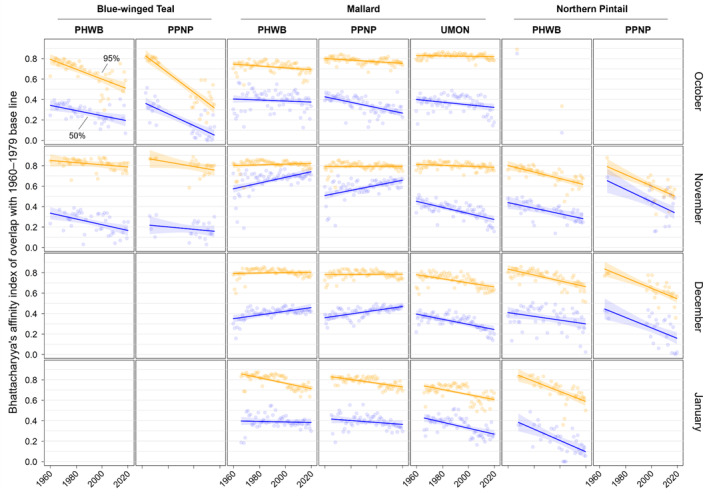
Changes in overlap of band recoveries from blue‐winged teal, mallards, and Northern Pintail in the Central and Mississippi flyways of North America for each year between 1960 and 2019 compared to a historical base line (1960–1979). Shown are the Bhattacharyya's affinities (as indices of overlap) for the 50% (blue) and 95% (orange) isopleths of kernel density estimator‐based utilization distributions for each combination of year, month of recovery (October–January), and banding region with 30 or more band recoveries. See caption of Figure 2 for full names of banding regions.

We found substantial support for effects of year, month, and banding region on centroid longitude and latitude, relative area, and distributional overlap for all three species with only a few exceptions (Tables S3–S10). Overall, band recovery centroid locations for blue‐winged teal shifted southwest and centroid locations for mallard and northern pintail shifted northeast by one hundred to several hundred kilometers for most banding regions, months, and isopleth levels (Figures 4 and 5; Tables S11 and S12). Relative area increased and distributional overlap decreased for all species, months, banding regions, and isopleth levels with only a few exceptions (Figures 6 and 7; Tables S13 and S14), with changes in metrics generally being greater for blue‐winged teal and northern pintail compared to mallards. Distributional overlap had greater annual variation compared to the other three metrics. Finally, changes in distributional metrics were frequently more pronounced at the 50% isopleth level than at the 95% isopleth level, especially longitudinal shifts for northern pintail, latitudinal shifts for blue‐winged teal and northern pintail, changes in relative area for all three species, and changes in overlap for mallard (Tables S11–S14). Banding region explained little variation in centroid latitude for blue‐winged teal and relative area for both blue‐winged teal (95% isopleth only) and northern pintail, whereas we found only limited support for an effect of month on relative area for northern pintail.

#### 3.2.1 October

Changes in band recovery distribution metrics often varied by month, and month‐specific patterns regularly varied among species (Figures 2, 3, 4, 5, 6, 7; Figure S2, Tables S11–S14). In October, centroid locations for blue‐winged teal shifted westwards by 131–265 km (1.63–3.30 degrees) and southwards by 486–874 km (4.37–7.85 degrees), whereas centroids shifted comparable distances eastwards for northern pintail (102–375 km; 1.31–4.80 degrees). Latitudinal shifts for northern pintail were insignificant because of wide confidence intervals, while shifts in centroid locations for mallards were minimal and often insignificant. During 1960–2019, relative area of October band recovery isopleths increased for blue‐winged teal by 77%–536% but not for mallards. Over the same period, decreases in distributional overlap were ~2–3× greater for blue‐winged teal (0.15–0.58) than for mallards (0.08–0.16).

#### 3.2.2 November

In November, centroid locations shifted south for blue‐winged teal (179 ± 88 km; 1.61 ± 0.79 degrees; 95% isopleths only) and mallard (111–225 km; 0.99–2.02 degrees), but ~2× those distances northwards for northern pintail (204–404 km; 1.83–3.63 degrees). Centroid locations shifted westward by 158 ± 29 km (1.65 ± 0.31 degrees; 50% isopleths only) for blue‐winged teal but eastward by 88–173 km (1.01–2.02 degrees) for mallard, and rarely changed for northern pintail. Relative area increased by 172 ± 103% for blue‐winged teal (50% isopleth only), 73%–166% for northern pintail, and only 20%–55% for mallard (except for bands from the PPNP). Finally, distributional overlap in November decreased for northern pintail by 0.16–0.36 for most isopleth levels and banding regions, but only rarely for blue‐winged teal (50% isopleth from PWHB: 0.17 ± 0.05), whereas changes in overlap for mallard were mixed (−0.18 to 0.17) depending on banding region and isopleth level.

#### 3.2.3 December

In December, centroid locations showed consistent and substantial northward shifts for both mallard (134–603 km; 1.21–5.42 degrees) and northern pintail (200–438 km; 1.80–3.93 degrees), but with large variation among banding regions and isopleth levels. Centroid locations of mallard and northern pintail band recovery distributions either shifted eastwards (mallard: 119–282 km; 1.32–3.18 degrees, northern pintail: 121 ± 38 km; 1.31 ± 0.42 degrees) or did not shift depending on banding region and isopleth level. Increases in relative area tended to be greater for northern pintail (+73% to 166%) than for mallard (+21% to 118%). Similar to November, distributional overlap decreased substantially for northern pintail (0.11–0.31), whereas changes in overlap for mallard varied among banding regions and isopleth levels.

#### 3.2.4 January

Similar to December, centroid locations in January shifted northwards for mallard (143–514 km; 1.28–4.62 degrees) and especially northern pintail (353–868 km; 3.17–7.80 degrees) for all banding regions and isopleth levels. Centroid locations of northern pintail shifted eastwards by 123–319 km (1.31–3.38 degrees), while comparable eastward shifts for mallard occurred in only one of three banding regions (UMON; 192–266 km; 2.13–2.94 degrees). Finally, increases in relative area (+73% to 166%) and decreases in overlap (0.30–0.33) for northern pintail were up to 2× greater than for mallard (relative area: +36% to 63%; overlap: 0.11–0.18) depending on banding region.

#### 3.2.5 Banding regions

When comparing changes in metrics across banding regions, recoveries of mallard bands originating from the UMON shifted ~3.5× farther east across all months (169 ± 37 vs. 49 ± 37 km; 1.95 ± 0.43 vs. 0.56 ± 0.43 degrees), were recovered ~4 degrees (445 km) farther north in November, shifted ~2.3× farther north during December and January (499 ± 56 vs. 216 ± 56 km; 4.48 ± 0.50 vs. 1.94 ± 0.50 degrees), and had lower distributional overlap compared to bands from the PHWB and PPNP (Figure 2, Figure S2). Moreover, increases in relative area tended to be smaller for mallard bands from the PPNP (+16 ± 11%) compared to bands from the PHWB (+43 ± 13%) and UMON (+35 ± 13%) across all months. In contrast, we observed little systematic variation in changes in distribution metrics among banding regions for blue‐winged teal and northern pintail in any month, except for a 2–3× steeper decline in distributional overlap for birds banded in the PPNP compared to the PWHB for blue‐winged teal in October (0.35 ± 0.05 vs. 0.15 ± 0.05) and northern pintail in November (0.35 ± 0.07 vs. 0.18 ± 0.05) and December (0.31 ± 0.06 vs. 0.14 ± 0.04). Finally, distributional overlap increased for mallard bands from the PPNP and PHWB in both November and December, whereas it did not change or decreased for all other combinations of species, month, and banding region.

## 4 DISCUSSION

Using 60 years of band recovery data for three duck species in the Central and Mississippi flyways of North America, we showed that fall migration and winter distributions have shifted and highlighted important differences among species, months, and banding regions (i.e., subpopulations). Between 1960 and 2019, band recovery centroid locations for blue‐winged teal shifted southwest and centroid locations for mallard and northern pintail shifted northeast by one hundred to several hundred kilometers for most banding regions, months, and isopleth levels. Relative area compared to the 1960–1979 baseline increased and distributional overlap decreased for all species, months, banding regions, and isopleth levels with only a few exceptions. Distributional shifts varied by month, with southward shifts for blue‐winged teal most pronounced in October and northward shifts for mallard and northern pintail greatest during December and January. For mallards, distributional metric response varied among subpopulations, including 2–4‐fold differences in longitude, latitude, and overlap, while differences among subpopulations were minimal for blue‐winged teal and northern pintail. However, magnitude of observed shifts was small relative to the annual home ranges of our study species, and we did not find evidence of wholesale abandonment of large wintering regions or switching between flyways by mallard or northern pintail.

Distributional shifts in band recovery data from December and January support the popular notion that winter distributions of duck species have shifted north (Brook et al., 2009; Gunnarsson et al., 2012; La Sorte & Thompson III, 2007; Lehikoinen et al., 2013; Meehan et al., 2021; Sauter et al., 2010; Švažas et al., 2001; Verheijen et al., 2024), and past trends suggest they may continue to do so in the future (Notaro et al., 2016; Reese & Skagen, 2017). At the same time, variation in response among species and subpopulations join previously reported inconsistencies in the extent and direction of distributional change across taxa (Lenoir & Svenning, 2015; Pecl et al., 2017; Rubenstein et al., 2023), duck species in particular (Green & Krementz, 2008; Guillemain, Champagnon et al., 2015; Guillemain, Pernollet et al., 2015; Verheijen et al., 2024), or subpopulations of ducks (Cox et al., 2023). We therefore conclude that long‐term distributional changes are often complex and that summarizing shifts across species, months, and distinct breeding regions could mask underlying fine‐scale patterns important to conservation. Moreover, because of our focus on variation in distributional shifts among breeding regions, we did not have sufficient sample sizes to assess whether distributional shifts vary among age and sex classes, despite temporal variation in the relative proportion of band recoveries from different age classes and sexes (Alisauskas et al., 2013). Thus, continued deployment of bands across broad geographic areas occupied by subpopulations with different responses and pressures during the preseason period will be increasingly important to better understanding distributional shifts in response to land use alternations, climate change, and other factors. In particular, ensuring that band deployments encompass a large sample of all age and sex cohorts within each biologically relevant banding region will better enable future analysis to differentiate between more subtle differences in distributional changes (USFWS, 2016).

Shifts in centroid locations and decreases in distributional overlap indicate that core areas and broad ranges of all three duck species have expanded into previously less‐used areas. At the same time, increases in the relative area compared to the 1960–1979 baseline, especially for blue‐winged teal and northern pintail, suggest that these species are not fully abandoning historically used areas, likely because of a lack of change in the southern extent of their broad range (Figure 3, Figure S2). Northward shifts, as seen in mallard and northern pintail, and range expansions have been widely documented in waterfowl (Brook et al., 2009; Gunnarsson et al., 2012; La Sorte & Thompson III, 2007; Lehikoinen et al., 2013; Meehan et al., 2021; Sauter et al., 2010; Švažas et al., 2001; Verheijen et al., 2024), other bird species, and taxa (Lenoir & Svenning, 2015; Pecl et al., 2017; Rubenstein et al., 2023). Although evaluating drivers of distributional change was beyond the scope of this study, northward shifts and range expansions are likely caused by a range of factors. Climate warming has led to more favorable winter habitat conditions (e.g., availability of open water) at northern latitudes during the last several decades (NOAA, 2023). Simultaneously, favorable climatic conditions have led to the northward expansion of corn, rice, and other grain crops (USDA NASS, 2017), which are consumed by many duck species, including mallard and northern pintail (Baldassarre & Bolen, 2006; Hitchcock Jr. et al., 2021; Tidwell et al., 2013). However, unless solely caused by increases in energy availability due to agricultural expansion, observed northwards shifts of mallard and northern pintail band recoveries in December and January could lead to greater energetic and habitat demands at higher latitudes during those months, and could increase the frequency and strength of interactions (e.g., competition, predator/prey interactions) with resident or other migratory species that have not shifted their winter distributions (Berger et al., 2001; Charter et al., 2016).

All four distributional metrics varied substantially among species in absolute values and extent of change. Interspecific variation in the direction and extent of change in centroid locations documented in this study aligns with earlier findings for ducks based on band and wing recoveries (Green & Krementz, 2008; Verheijen et al., 2024) or other data types (Brook et al., 2009; Guillemain, Champagnon et al., 2015; Guillemain, Pernollet et al., 2015; Gunnarsson et al., 2012; La Sorte & Thompson III, 2007; Lehikoinen et al., 2013; Meehan et al., 2021; Sauter et al., 2010; Švažas et al., 2001). In contrast, few studies have documented interspecific variation in changes in relative area and distributional overlap in ducks (but see Cox et al., 2023). Although band recovery distributions of mallard and northern pintail shifted north during December and January, increases in relative area compared to the 1960–1979 baseline were 2–3× greater for northern pintail, which could have several potential explanations. First, increases in relative area could suggest that new regions at higher latitudes have become more widely available as wintering sites to both species over the past 60 years, but also that historical wintering sites were not abandoned and still provide wintering habitat for northern pintail during a portion of the autumn–winter period. On the other hand, mallards appear to have partially abandoned portions of their historical wintering ranges, especially the southmost extents (e.g., southern Louisiana; Hagy et al., 2014). Second, the timing and extent of autumn migration by mallards may be driven more by weather than photoperiodic cues compared to northern pintail, with a substantial portion of mallards often not migrating farther south than necessary to obtain food (Bellrose, 1980; Jorde et al., 1983, 1984; Pearse et al., 2023; Schummer et al., 2010; Weller et al., 2022). Finally, mallards could have greater plasticity in the timing and length of autumn migration than northern pintail, which could lead to a greater percentage of mallards staying farther north during the winter compared to northern pintail when environmental conditions are favorable. Because of interspecific variation in response to changing environmental conditions, distributional shifts for one species are not easily deduced from responses by other closely related species or taxa, emphasizing the importance of a species‐specific understanding of the drivers of geographic distributions to improve population management and habitat conservation.

December and January distributions of mallard band recoveries from the Upper Mississippi River/Great Lakes Joint Venture and Ontario geographies shifted 2–3× farther east and 2–5× farther north compared to the two more western subpopulations. As a result, December and January core areas of mallard band recovery distributions from the Upper Mississippi River/Great Lakes Joint Venture and Ontario geographies now include states as far north as Illinois, Michigan, Ohio, and Wisconsin, whereas core areas for mallard band recoveries from the other two subpopulations have largely remained in the Lower Mississippi Valley (Figure S2). We identified several possible explanations for observed differences in changes in distributional metrics among mallard subpopulations. First, the landscapes that each subpopulation most frequently occupies during autumn–winter may have changed in different ways and at different rates. Portions of the Central and Mississippi flyways have changed substantially in climate (NOAA, 2023), land use (USDA NASS, 2017), and the quality and quantity of wetlands on the landscape (Dahl, 2011) during 1960–2019, but not necessarily along a longitudinal gradient. Second, genetic differences among mallard subpopulations may cause differences in migratory behavior, regardless of whether they are in response to landscape‐level changes on the wintering grounds (Lavretsky & Sedinger, 2023; Merlin & Liedvogel, 2019; Schummer et al., 2023). Recovery longitude of mallard bands is partially dependent on breeding regions of banding, whether defined by longitude, banding reference area, Canadian Province, or migratory flyway (Cox et al., 2023; Munro & Kimball, 1982; Roberts et al., 2022; Szymanski & Dubovsky, 2013; Verheijen et al., 2024). However, it is presently unknown whether variation in gene frequencies among subpopulations is sufficient to produce differential migratory behavior in response to changing environmental conditions. An important exception may be the long‐term mixing of game‐farm and wild mallard populations, especially in the eastern portions of our study area (Heusmann, 1991; Schummer et al., 2023). Originally restricted to the east coast, game‐farm mallard genes have become more prevalent in some eastern portions of the Mississippi flyway, with only 35% of hatch‐year mallards harvested in northwestern Ohio being genetically wild mallards (Schummer et al., 2023). If game‐farm mallards lack an innate behavior to migrate to traditional wintering grounds (Schummer et al., 2023), the high proportion of game‐farm mallard genes among mallards from the Upper Mississippi River/Great Lakes Joint Venture and Ontario geographies could help explain their more northward shift in winter distributions compared to the other two subpopulations. That said, variation in distributional shifts among subpopulations highlights the need for the use of representative samples when making whole‐population level inference. Future researchers should be cognizant of lumping banding data across breeding ranges without controlling for changes in band deployment among subpopulations over time, as observed in our study (Figure S3).

Variation in response between the core area (50%) and broad range (95%) of band recovery distributions show the complexities in geographical shifts at different spatial scales. Increases in relative area compared to the 1960–1979 baseline were often much greater for the core area than the broad range for all three species (Table S13), suggesting an overall larger and more uniform distribution of ducks throughout their range compared to historical conditions. Shifts in centroid latitude and longitude of the core area were also greater than those of the broad range for blue‐winged teal and northern pintail during several months (Tables S11 and S12), with band recovery distributions becoming more or less lopsided depending on whether the centroid of the core area moved away or toward the centroid of the broad range. Both an expansion of the core area and greater shift in centroid location than for the broad range suggest that band recovery distributions of blue‐winged teal and northern pintail have not only shifted over time but also changed how they are distributed throughout their broad range. An increase in relative area could either indicate a need for animals to distribute themselves over a larger area because of habitat loss or a decrease in habitat quality, or that more habitat is available and animals are able to distribute themselves more freely, perhaps to reduce competition for food resources (Fretwell & Lucas Jr., 1969; Křivan et al., 2008). Aside from underlying causes, recent blue‐winged teal population estimates have been comparable or slightly above long‐term averages, whereas northern pintail estimates in 2022 were ~46% below their long‐term average population estimate (1955–2019; USFWS, 2022). Due to the occupancy of a greater area during winter in recent years, densities of both species are likely to be lower in the core areas now than in the 1960s. Thus, distributional shifts of species are complex, and conclusions based on simple range limits could overlook important considerations for landscape conservation and population management.

Distributional metrics were frequently correlated and therefore changing in the same direction. Specifically, we observed strong correlations between centroid latitude and the northern and southern extent of band recovery distributions, between centroid longitude and eastern and western extent of distributions, and among all three metrics of distributional overlap. These strong correlations complement shifts in distributional overlap and strengthen our conclusion that movements are not restricted to within historical boundaries, but rather that species are shifting their range. Correlations among distributional metrics appear common for many species and taxa, especially when distributional shifts are driven by climate change or confounding factors (e.g., northward expansion of crops enabled by climate change), but perhaps less so if regulated by other drivers (Donelson et al., 2019; Fieberg & Kochanny, 2005; Lenoir & Svenning, 2015; Rubenstein et al., 2023; Saunders et al., 2022).

In conclusion, distributional shifts of several hundred kilometers, increases in relative area compared to the 1960–1979 baseline, and decreases in distributional overlap observed in the band recovery distributions of three duck species join previous literature demonstrating temporal changes in geographic distributions of waterfowl (Brook et al., 2009; Cox et al., 2023; Guillemain, Champagnon et al., 2015; Guillemain, Pernollet et al., 2015; Gunnarsson et al., 2012; La Sorte & Thompson III, 2007; Lehikoinen et al., 2013; Meehan et al., 2021; Moore et al., 2023; Sauter et al., 2010; Švažas et al., 2001; Verheijen et al., 2024) and other taxa (Lenoir & Svenning, 2015; Pecl et al., 2017; Rubenstein et al., 2023). Variation in the direction and extent of change in distributional metrics among subpopulations indicates that intraspecific variation in distributional change might be more common than previously thought, thus challenging the belief that distribution shifts can be understood and managed exclusively at the species level (Cox et al., 2023; Meehan et al., 2021). Moreover, the observed intraspecific variation further stresses the importance of selecting representative samples of animals when drawing population‐level inference on animal movements, habitat selection, and species occurrence. This is especially true when using individual tracking methods such as satellite‐transmitters that (although providing highly detailed information) are generally deployed on a relatively small number of individuals representing perhaps the most optimal condition or a targeted group (e.g., heaviest, adult cohort). Overall, quantifying patterns of historical change is a necessary first step to understanding temporal, interspecific, and intraspecific variation in species distributions. Linking observed distributional shifts to potential drivers could then not only improve our understanding of how species interact with changing environments, but also help inform landscape‐scale conservation of biodiversity and management efforts.

## AUTHOR CONTRIBUTIONS

**Bram H. F. Verheijen:** Formal analysis (lead); investigation (lead); methodology (lead); validation (lead); visualization (lead); writing – original draft (lead); writing – review and editing (equal). **Elisabeth B. Webb:** Conceptualization (equal); funding acquisition (equal); investigation (supporting); project administration (lead); supervision (lead); writing – review and editing (equal). **Michael G. Brasher:** Conceptualization (equal); funding acquisition (equal); investigation (supporting); supervision (supporting); writing – review and editing (equal). **Heath M. Hagy:** Conceptualization (equal); funding acquisition (equal); investigation (supporting); supervision (supporting); writing – review and editing (equal).

## CONFLICT OF INTEREST STATEMENT

There are no conflicts of interest that we know of for this manuscript.

## Supporting information


Data S1:

## Data Availability

Our research used publicly available band recovery data curated by the United States Geological Survey Bird Banding Laboratory. Year‐specific sample sizes and estimates of distributional metrics for each species, month, and banding region can be found in supplemental_table_15.xlsx.
