# Supplementary material for: Long‐term changes in autumn–winter harvest distributions vary among duck species, months, and subpopulations
Source: Ecol Evol. 2024 Jun 3;14(6):e11331. doi: 10.1002/ece3.11331 (PMC11145621; doi:10.1002/ece3.11331)

**Supplemental Table 1.** Calculations of the three overlap metrics used in our study (HR, PHR, and Bhattacharyya's affinity [BA]; adapted from Fieberg and Kochanny 2005).

**Overlap Metric --- HR**

$${\left( 1 \right) HR}_{i,j}= A_{i,j} / A_{i}$$

where HR_i,j_ is the proportion of animal i’s home range that is overlapped by animal j’s home range, A_i_ is the area of animal i’s home range, and A_i,j_ is the area of overlap between the two animals' home ranges.

**Overlap Metric --- PHR**

Let UD_i_ be the estimated utilization distribution (UD) for animal i and UD_j_ the estimated UD for animal j. Further, assume that UD_i_ is >0 only in A_i_ and UD_j_ is >0 only in A_j_. In other words, A_i_ and A_j_ represent the full extent of spatial use for animals i and j.

Rather than measure the proportion of animal i's home range overlapped by animal j, one can calculate the probability of animal j being located in animal i's home range:

$${\left( 2 \right) PHR}_{i,j}=\iint_{A_{i}} \hat{U}D_{j} \left( x,y \right) dxdy$$

PHR_i,j_ and PHR_j,i_ will be equivalent to HR_i,j_ and HR_j,i_ when the habitat utilization distributions are constant across space for both individuals (i.e., when UD_i_ is a uniform distribution for i = 1, 2).

These measures can be modified for home ranges based on smaller probability contours (e.g., a 50% or 95% probability contour). Let A_i,p_ be the area associated with animal i's home range as defined by the p^th^ probability contour of UD_i_, and let UD_i,p_ be the estimated conditional UD for animal i (i.e., the probability distribution for animal i given that it is in A_i,p_):

$$(3) \hat{U}D_{i,p}(x,y)= \left\{ \begin{aligned} 0 \mathrm{if} \left( x,y \right)\notin A_{i,p} \\ \hat{U}D_{i,p}\left( x,y \right) / p \mathrm{if} (x,y) \in A_{i,p} \end{aligned} \right.$$

By replacing the UDs in equation (2) with the conditional UDs from equation (3), we can calculate appropriate overlap indices that limit inference to home ranges defined by smaller probability contours.

**Overlap Metric --- Bhattacharyya's affinity (BA)**

Bhattacharyya's affinity (Bhattacharyya 1943), a statistical measure of affinity between 2 populations, is a function of the product of the 2 UDs, UD_i_(x,y) × UD_j_(x,y) = the joint distribution of the UDs of two animals under the assumption that they use space independently of one another:

$$\left( 4 \right) BA=\iint_{-\infty-\infty}^{\infty\infty} \sqrt{\hat{U}D_{i} (x,y)} \times\sqrt{\hat{U}D_{j} \left( x,y \right) dxdy}$$

Conditional estimates of the BA index for specific probability contours can also be measured by replacing the UD_i_ values in equation (4) with the conditional UD estimates from equation (3).

**LITERATURE CITED**

Bhattacharyya, A. 1943. On a measure of divergence between two statistical populations defined by their probability distributions. Bulletin of the Calcutta Mathematical Society 35:99-109.

Fieberg, J., & C. O. Kochanny. 2005. Quantifying home-range overlap: The importance of the utilization distribution. Journal of Wildlife Management 69:1346–1359.

**Supplemental Table 2.** The number of band recoveries for Blue-winged Teal (BWTE), Mallards (MALL), and Northern Pintail (NOPI) recovered in the Central or Mississippi Flyways between 1960–2019 split by banding region and month of recovery (October–January). Banding regions include the Prairie Habitat and Western Boreal Area Joint Ventures (PHWB), the Prairie Pothole and Northern Great Plains Joint Ventures (PPNP), and the Upper Mississippi River/Great Lakes Joint Venture and Ontario (UMON).

| **Species** | **Banding Region** | **Year** | **N_October_** | **N_November_** | **N_December_** | **N_January_** | **N_Total_** |
| --- | --- | --- | --- | --- | --- | --- | --- |
| BWTE | PHWB | All | 3192 | 2526 | 1564 | 992 | 8274 |
| BWTE | PPNP | All | 2714 | 1394 | 843 | 543 | 5494 |
| **BWTE** | **All** | **All** | **5906** | **3920** | **2407** | **1535** | **13768** |
|  |  |  |  |  |  |  |  |
| MALL | PHWB | All | 15677 | 42787 | 50725 | 38740 | 147929 |
| MALL | PPNP | All | 21254 | 22521 | 24972 | 18421 | 87168 |
| MALL | UMON | All | 38159 | 21498 | 17974 | 12784 | 90415 |
| **MALL** | **All** | **All** | **75090** | **86806** | **93671** | **69945** | **325512** |
|  |  |  |  |  |  |  |  |
| NOPI | PHWB | All | 998 | 2399 | 2807 | 2860 | 9064 |
| NOPI | PPNP | All | 1485 | 1447 | 1772 | 1906 | 6610 |
| **NOPI** | **All** | **All** | **2483** | **3846** | **4579** | **4766** | **15674** |

**Supplemental Table 3.** Model selection table of models that explain variation in the centroid longitude of the 50% isopleth of kernel density estimator-based utilization distributions of band recoveries from Blue-winged Teal, Mallards, and Northern Pintail in the Central and Mississippi Flyways of North America between 1960 and 2019. Model structure terms include year (continuous), month (October–January; categorical), Joint Venture of banding origin (JVB), banding latitude, banding longitude, and several possible two-way and three-way interaction terms. Model statistics include the number of parameters (K), the log likelihood, AIC_c_ values, difference in AIC_c_ values compared to the model with the lowest AIC_c_ value (ΔAICc), and the model weight.

| **Blue-winged Teal – Model structure** | | | | | | | | | |  | **Model statistics** | | | | |
| --- | --- | --- | --- | --- | --- | --- | --- | --- | --- | --- | --- | --- | --- | --- | --- |
| **Intercept** | **Year** | **Month** | **JVB** | **Year × Month** | **Year × JVB** | **Month × JVB** | **Year × Month × JVB** | **Banding latitude** | **Banding longitude** |  | **K** | **Log likelihood** | **AICc** | **ΔAICc** | **Model weight** |
| + | + | + | + |  |  | + |  |  |  |  | 6 | -179.03 | 370.76 | 0 | 0.537 |
| + | + | + | + |  | + | + |  |  |  |  | 7 | -178.91 | 372.75 | 1.999 | 0.198 |
| + | + | + | + | + |  | + |  |  |  |  | 7 | -179.03 | 372.99 | 2.231 | 0.176 |
| + | + | + | + | + | + | + |  |  |  |  | 8 | -178.90 | 375.00 | 4.249 | 0.064 |
| + | + | + | + | + | + | + | + |  |  |  | 9 | -178.68 | 376.87 | 6.115 | 0.025 |
| + | + | + | + |  |  |  |  |  |  |  | 5 | -191.05 | 392.60 | 21.840 | 0 |
| + | + | + | + | + |  |  |  |  |  |  | 6 | -190.89 | 394.46 | 23.706 | 0 |
| + | + | + | + |  | + |  |  |  |  |  | 6 | -190.95 | 394.60 | 23.841 | 0 |
| + |  | + | + |  |  | + |  |  |  |  | 5 | -192.69 | 395.87 | 25.110 | 0 |
| + | + | + |  |  |  |  |  | + |  |  | 5 | -192.84 | 396.17 | 25.411 | 0 |
| + | + | + | + | + | + |  |  |  |  |  | 7 | -190.75 | 396.43 | 25.669 | 0 |
| + | + | + |  | + |  |  |  | + |  |  | 6 | -192.60 | 397.89 | 27.132 | 0 |
| + | + | + |  |  |  |  |  |  | + |  | 5 | -198.86 | 408.21 | 37.457 | 0 |
| + | + | + |  |  |  |  |  |  |  |  | 4 | -200.71 | 409.74 | 38.985 | 0 |
| + | + | + |  | + |  |  |  |  | + |  | 6 | -198.65 | 409.98 | 39.228 | 0 |
| + | + | + |  | + |  |  |  |  |  |  | 5 | -200.64 | 411.77 | 41.017 | 0 |
| + |  | + | + |  |  |  |  |  |  |  | 4 | -202.02 | 412.37 | 41.613 | 0 |
| + |  | + |  |  |  |  |  | + |  |  | 4 | -206.35 | 421.02 | 50.261 | 0 |
| + |  | + |  |  |  |  |  |  |  |  | 3 | -212.70 | 431.58 | 60.827 | 0 |
| + |  | + |  |  |  |  |  |  | + |  | 4 | -212.68 | 433.69 | 62.932 | 0 |
| + | + |  |  |  |  |  |  |  | + |  | 4 | -261.32 | 530.95 | 160.198 | 0 |
| + |  |  | + |  |  |  |  |  |  |  | 3 | -265.23 | 536.66 | 165.903 | 0 |
| + |  |  |  |  |  |  |  |  | + |  | 3 | -265.65 | 537.50 | 166.743 | 0 |
| + | + |  | + |  |  |  |  |  |  |  | 4 | -264.63 | 537.58 | 166.828 | 0 |
| + | + |  |  |  |  |  |  | + |  |  | 4 | -265.00 | 538.33 | 167.574 | 0 |
| + |  |  |  |  |  |  |  | + |  |  | 3 | -266.25 | 538.68 | 167.929 | 0 |
| + | + |  | + |  | + |  |  |  |  |  | 5 | -264.57 | 539.62 | 168.867 | 0 |
| + |  |  |  |  |  |  |  |  |  |  | 2 | -271.26 | 546.62 | 175.868 | 0 |
| + | + |  |  |  |  |  |  |  |  |  | 3 | -270.23 | 546.65 | 175.892 | 0 |

| **Mallard – Model structure** | | | | | | | | | |  | **Model statistics** | | | | |
| --- | --- | --- | --- | --- | --- | --- | --- | --- | --- | --- | --- | --- | --- | --- | --- |
| **Intercept** | **Year** | **Month** | **JVB** | **Year × Month** | **Year × JVB** | **Month × JVB** | **Year × Month × JVB** | **Banding latitude** | **Banding longitude** |  | **K** | **Log likelihood** | **AICc** | **ΔAICc** | **Model weight** |
| + | + | + | + | + | + | + | + |  |  |  | 25 | -1029.68 | 2111.29 | 0 | 1 |
| + | + | + | + | + | + | + |  |  |  |  | 19 | -1045.69 | 2130.49 | 19.200 | 0 |
| + | + | + | + |  | + | + |  |  |  |  | 16 | -1057.16 | 2147.13 | 35.837 | 0 |
| + | + | + | + | + |  | + |  |  |  |  | 17 | -1063.79 | 2162.49 | 51.197 | 0 |
| + | + | + | + |  |  | + |  |  |  |  | 14 | -1074.79 | 2178.19 | 66.902 | 0 |
| + |  | + | + |  |  | + |  |  |  |  | 13 | -1094.40 | 2215.33 | 104.045 | 0 |
| + | + | + | + | + | + |  |  |  |  |  | 13 | -1223.15 | 2472.84 | 361.551 | 0 |
| + | + | + | + |  | + |  |  |  |  |  | 10 | -1230.26 | 2480.85 | 369.559 | 0 |
| + | + | + | + | + |  |  |  |  |  |  | 11 | -1231.23 | 2484.84 | 373.554 | 0 |
| + | + | + | + |  |  |  |  |  |  |  | 8 | -1238.22 | 2492.65 | 381.364 | 0 |
| + |  | + | + |  |  |  |  |  |  |  | 7 | -1250.86 | 2515.88 | 404.586 | 0 |
| + | + | + |  | + |  |  |  |  | + |  | 10 | -1335.93 | 2692.19 | 580.899 | 0 |
| + | + | + |  |  |  |  |  |  | + |  | 7 | -1341.91 | 2697.98 | 586.694 | 0 |
| + |  | + |  |  |  |  |  |  | + |  | 6 | -1353.23 | 2718.58 | 607.290 | 0 |
| + | + |  | + |  | + |  |  |  |  |  | 7 | -1373.10 | 2760.37 | 649.078 | 0 |
| + | + |  | + |  |  |  |  |  |  |  | 5 | -1378.34 | 2766.77 | 655.477 | 0 |
| + |  |  | + |  |  |  |  |  |  |  | 4 | -1388.57 | 2785.20 | 673.910 | 0 |
| + | + | + |  | + |  |  |  | + |  |  | 10 | -1419.03 | 2858.38 | 747.092 | 0 |
| + | + | + |  |  |  |  |  | + |  |  | 7 | -1423.86 | 2861.88 | 750.591 | 0 |
| + |  | + |  |  |  |  |  | + |  |  | 6 | -1432.46 | 2877.04 | 765.753 | 0 |
| + | + |  |  |  |  |  |  |  | + |  | 4 | -1460.02 | 2928.10 | 816.807 | 0 |
| + |  |  |  |  |  |  |  |  | + |  | 3 | -1469.48 | 2944.99 | 833.704 | 0 |
| + | + |  |  |  |  |  |  | + |  |  | 4 | -1519.04 | 3046.13 | 934.840 | 0 |
| + |  |  |  |  |  |  |  | + |  |  | 3 | -1526.85 | 3059.73 | 948.442 | 0 |
| + | + | + |  |  |  |  |  |  |  |  | 6 | -1603.54 | 3219.21 | 1107.917 | 0 |
| + | + | + |  | + |  |  |  |  |  |  | 9 | -1601.21 | 3220.67 | 1109.384 | 0 |
| + |  | + |  |  |  |  |  |  |  |  | 5 | -1607.87 | 3225.82 | 1114.529 | 0 |
| + | + |  |  |  |  |  |  |  |  |  | 3 | -1659.75 | 3325.53 | 1214.239 | 0 |
| + |  |  |  |  |  |  |  |  |  |  | 2 | -1664.21 | 3332.45 | 1221.157 | 0 |

| **Northern Pintail – Model structure** | | | | | | | | | |  | **Model statistics** | | | | |
| --- | --- | --- | --- | --- | --- | --- | --- | --- | --- | --- | --- | --- | --- | --- | --- |
| **Intercept** | **Year** | **Month** | **JVB** | **Year × Month** | **Year × JVB** | **Month × JVB** | **Year × Month × JVB** | **Banding latitude** | **Banding longitude** |  | **K** | **Log likelihood** | **AICc** | **ΔAICc** | **Model weight** |
| + | + | + | + | + |  | + |  |  |  |  | 13 | -306.81 | 641.53 | 0 | 0.424 |
| + | + | + | + | + | + | + |  |  |  |  | 14 | -305.88 | 641.98 | 0.456 | 0.338 |
| + | + | + | + | + | + |  |  |  |  |  | 11 | -310.71 | 644.80 | 3.278 | 0.082 |
| + | + | + |  | + |  |  |  |  | + |  | 10 | -312.13 | 645.40 | 3.878 | 0.061 |
| + | + | + | + | + |  |  |  |  |  |  | 10 | -312.29 | 645.73 | 4.203 | 0.052 |
| + | + | + | + | + | + | + | + |  |  |  | 17 | -305.37 | 648.03 | 6.503 | 0.016 |
| + | + | + |  | + |  |  |  | + |  |  | 10 | -313.67 | 648.48 | 6.951 | 0.013 |
| + | + | + |  |  |  |  |  |  | + |  | 7 | -318.46 | 651.50 | 9.975 | 0.003 |
| + | + | + | + |  | + | + |  |  |  |  | 11 | -314.14 | 651.66 | 10.135 | 0.003 |
| + | + | + | + |  |  | + |  |  |  |  | 10 | -315.32 | 651.79 | 10.261 | 0.003 |
| + | + | + | + |  | + |  |  |  |  |  | 8 | -317.58 | 651.90 | 10.373 | 0.002 |
| + | + | + |  |  |  |  |  |  |  |  | 7 | -319.19 | 652.96 | 11.434 | 0.001 |
| + | + | + |  | + |  |  |  |  |  |  | 9 | -317.66 | 654.24 | 12.716 | 0.001 |
| + | + | + |  |  |  |  |  | + |  |  | 7 | -320.14 | 654.85 | 13.327 | 0.001 |
| + | + | + |  |  |  |  |  |  |  |  | 6 | -324.78 | 661.99 | 20.462 | 0 |
| + |  | + | + |  |  |  |  |  |  |  | 6 | -325.48 | 663.40 | 21.869 | 0 |
| + |  | + | + | + |  | + |  |  |  |  | 9 | -322.62 | 664.16 | 22.632 | 0 |
| + |  | + |  |  |  |  |  |  | + |  | 6 | -327.90 | 668.22 | 26.696 | 0 |
| + |  | + |  |  |  |  |  | + |  |  | 6 | -329.11 | 670.65 | 29.120 | 0 |
| + |  | + |  |  |  |  |  |  |  |  | 5 | -336.61 | 683.52 | 41.999 | 0 |
| + | + |  | + |  |  |  |  |  |  |  | 4 | -423.68 | 855.55 | 214.027 | 0 |
| + | + |  |  |  |  |  |  |  | + |  | 4 | -423.76 | 855.73 | 214.200 | 0 |
| + | + |  |  |  |  |  |  |  |  |  | 3 | -425.20 | 856.52 | 214.990 | 0 |
| + | + |  |  |  |  |  |  | + |  |  | 4 | -424.21 | 856.61 | 215.086 | 0 |
| + | + |  | + |  | + |  |  |  |  |  | 5 | -423.34 | 856.98 | 215.453 | 0 |
| + |  |  |  |  |  |  |  |  |  |  | 2 | -427.91 | 859.88 | 218.351 | 0 |
| + |  |  |  |  |  |  |  |  | + |  | 3 | -427.27 | 860.66 | 219.131 | 0 |
| + |  |  |  |  |  |  |  | + |  |  | 3 | -427.62 | 861.36 | 219.831 | 0 |
| + |  |  | + |  |  |  |  |  |  |  | 3 | -427.66 | 861.44 | 219.912 | 0 |

**Supplemental Table 4.** Model selection table of models that explain variation in the centroid longitude of the 95% isopleth of kernel density estimator-based utilization distributions of band recoveries from Blue-winged Teal, Mallards, and Northern Pintail in the Central and Mississippi Flyways of North America between 1960 and 2019. Model structure terms include year (continuous), month (October–January; categorical), Joint Venture of banding origin (JVB), banding latitude, banding longitude, and several possible two-way and three-way interaction terms. Model statistics include the number of parameters (K), the log likelihood, AIC_c_ values, difference in AIC_c_ values compared to the model with the lowest AIC_c_ value (ΔAICc), and the model weight.

| **Blue-winged Teal – Model structure** | | | | | | | | | |  | **Model statistics** | | | | |
| --- | --- | --- | --- | --- | --- | --- | --- | --- | --- | --- | --- | --- | --- | --- | --- |
| **Intercept** | **Year** | **Month** | **JVB** | **Year × Month** | **Year × JVB** | **Month × JVB** | **Year × Month × JVB** | **Banding latitude** | **Banding longitude** |  | **K** | **Log likelihood** | **AICc** | **ΔAICc** | **Model weight** |
| + | + | + | + | + | + | + | + |  |  |  | 9 | -154.96 | 329.42 | 0 | 0.562 |
| + | + | + | + | + | + | + |  |  |  |  | 8 | -156.95 | 331.10 | 1.676 | 0.243 |
| + | + | + | + | + |  | + |  |  |  |  | 7 | -158.31 | 331.55 | 2.130 | 0.194 |
| + | + | + | + |  | + | + |  |  |  |  | 7 | -163.50 | 341.92 | 12.499 | 0.001 |
| + | + | + | + |  |  | + |  |  |  |  | 6 | -165.77 | 344.23 | 14.811 | 0 |
| + | + | + | + | + |  |  |  |  |  |  | 6 | -170.62 | 353.93 | 24.511 | 0 |
| + | + | + | + | + | + |  |  |  |  |  | 7 | -170.51 | 355.95 | 26.527 | 0 |
| + | + | + |  | + |  |  |  | + |  |  | 6 | -172.30 | 357.28 | 27.858 | 0 |
| + | + | + | + |  |  |  |  |  |  |  | 5 | -179.31 | 369.10 | 39.678 | 0 |
| + | + | + |  | + |  |  |  |  | + |  | 6 | -178.29 | 369.26 | 39.841 | 0 |
| + | + | + |  | + |  |  |  |  |  |  | 5 | -179.76 | 370.01 | 40.589 | 0 |
| + | + | + | + |  | + |  |  |  |  |  | 6 | -178.86 | 370.42 | 40.995 | 0 |
| + |  | + | + |  |  | + |  |  |  |  | 5 | -180.75 | 371.99 | 42.571 | 0 |
| + | + | + |  |  |  |  |  | + |  |  | 5 | -181.18 | 372.85 | 43.432 | 0 |
| + | + | + |  |  |  |  |  |  |  |  | 4 | -186.79 | 381.91 | 52.486 | 0 |
| + | + | + |  |  |  |  |  |  | + |  | 5 | -186.21 | 382.91 | 53.484 | 0 |
| + |  | + | + |  |  |  |  |  |  |  | 4 | -191.10 | 390.52 | 61.099 | 0 |
| + |  | + |  |  |  |  |  | + |  |  | 4 | -195.20 | 398.71 | 69.290 | 0 |
| + |  | + |  |  |  |  |  |  |  |  | 3 | -199.65 | 405.50 | 76.079 | 0 |
| + |  | + |  |  |  |  |  |  | + |  | 4 | -199.47 | 407.26 | 77.836 | 0 |
| + | + |  | + |  |  |  |  |  |  |  | 4 | -221.55 | 451.42 | 121.995 | 0 |
| + | + |  |  |  |  |  |  |  | + |  | 4 | -221.98 | 452.29 | 122.864 | 0 |
| + | + |  |  |  |  |  |  | + |  |  | 4 | -222.45 | 453.22 | 123.798 | 0 |
| + | + |  | + |  | + |  |  |  |  |  | 5 | -221.37 | 453.24 | 123.812 | 0 |
| + |  |  | + |  |  |  |  |  |  |  | 3 | -224.12 | 454.43 | 125.006 | 0 |
| + |  |  |  |  |  |  |  | + |  |  | 3 | -226.22 | 458.63 | 129.205 | 0 |
| + | + |  |  |  |  |  |  |  |  |  | 3 | -227.66 | 461.51 | 132.084 | 0 |
| + |  |  |  |  |  |  |  |  | + |  | 3 | -228.69 | 463.58 | 134.152 | 0 |
| + |  |  |  |  |  |  |  |  |  |  | 2 | -230.95 | 466.00 | 136.579 | 0 |

| **Mallard – Model structure** | | | | | | | | | |  | **Model statistics** | | | | |
| --- | --- | --- | --- | --- | --- | --- | --- | --- | --- | --- | --- | --- | --- | --- | --- |
| **Intercept** | **Year** | **Month** | **JVB** | **Year × Month** | **Year × JVB** | **Month × JVB** | **Year × Month × JVB** | **Banding latitude** | **Banding longitude** |  | **K** | **Log likelihood** | **AICc** | **ΔAICc** | **Model weight** |
| + | + | + | + | + | + | + | + |  |  |  | 25 | -826.02 | 1703.97 | 0 | 0.999 |
| + | + | + | + | + | + | + |  |  |  |  | 19 | -839.89 | 1718.89 | 14.915 | 0.001 |
| + | + | + | + |  | + | + |  |  |  |  | 16 | -848.54 | 1729.88 | 25.901 | 0 |
| + | + | + | + | + |  | + |  |  |  |  | 17 | -847.79 | 1730.48 | 26.505 | 0 |
| + | + | + | + |  |  | + |  |  |  |  | 14 | -856.13 | 1740.88 | 36.906 | 0 |
| + | + | + | + | + | + |  |  |  |  |  | 13 | -868.94 | 1764.40 | 60.428 | 0 |
| + | + | + | + |  | + |  |  |  |  |  | 10 | -876.81 | 1773.94 | 69.963 | 0 |
| + | + | + | + | + |  |  |  |  |  |  | 11 | -875.96 | 1774.30 | 70.322 | 0 |
| + | + | + | + |  |  |  |  |  |  |  | 8 | -883.58 | 1783.37 | 79.392 | 0 |
| + |  | + | + |  |  | + |  |  |  |  | 13 | -906.96 | 1840.44 | 136.471 | 0 |
| + |  | + | + |  |  |  |  |  |  |  | 7 | -930.92 | 1875.99 | 172.021 | 0 |
| + | + |  | + |  | + |  |  |  |  |  | 7 | -939.23 | 1892.63 | 188.657 | 0 |
| + | + |  | + |  |  |  |  |  |  |  | 5 | -944.80 | 1899.68 | 195.710 | 0 |
| + |  |  | + |  |  |  |  |  |  |  | 4 | -990.10 | 1988.26 | 284.281 | 0 |
| + | + | + |  |  |  |  |  |  | + |  | 7 | -1005.52 | 2025.21 | 321.233 | 0 |
| + | + | + |  | + |  |  |  |  | + |  | 10 | -1002.70 | 2025.73 | 321.752 | 0 |
| + |  | + |  |  |  |  |  |  | + |  | 6 | -1047.41 | 2106.94 | 402.969 | 0 |
| + | + |  |  |  |  |  |  |  | + |  | 4 | -1058.67 | 2125.41 | 421.431 | 0 |
| + |  |  |  |  |  |  |  |  | + |  | 3 | -1098.56 | 2203.15 | 499.181 | 0 |
| + | + | + |  |  |  |  |  | + |  |  | 7 | -1261.25 | 2536.66 | 832.689 | 0 |
| + | + | + |  | + |  |  |  | + |  |  | 10 | -1258.73 | 2537.78 | 833.811 | 0 |
| + |  | + |  |  |  |  |  | + |  |  | 6 | -1281.16 | 2574.45 | 870.476 | 0 |
| + | + |  |  |  |  |  |  | + |  |  | 4 | -1288.82 | 2585.69 | 881.714 | 0 |
| + |  |  |  |  |  |  |  | + |  |  | 3 | -1309.55 | 2625.14 | 921.170 | 0 |
| + | + | + |  |  |  |  |  |  |  |  | 6 | -1674.79 | 3361.70 | 1657.722 | 0 |
| + | + | + |  | + |  |  |  |  |  |  | 9 | -1673.82 | 3365.91 | 1661.932 | 0 |
| + |  | + |  |  |  |  |  |  |  |  | 5 | -1679.81 | 3369.70 | 1665.726 | 0 |
| + | + |  |  |  |  |  |  |  |  |  | 3 | -1681.94 | 3369.91 | 1665.937 | 0 |
| + |  |  |  |  |  |  |  |  |  |  | 2 | -1687.56 | 3379.14 | 1675.170 | 0 |

| **Northern Pintail – Model structure** | | | | | | | | | |  | **Model statistics** | | | | |
| --- | --- | --- | --- | --- | --- | --- | --- | --- | --- | --- | --- | --- | --- | --- | --- |
| **Intercept** | **Year** | **Month** | **JVB** | **Year × Month** | **Year × JVB** | **Month × JVB** | **Year × Month × JVB** | **Banding latitude** | **Banding longitude** |  | **K** | **Log likelihood** | **AICc** | **ΔAICc** | **Model weight** |
| + | + | + | + |  | + |  |  |  |  |  | 8 | -221.27 | 459.28 | 0 | 0.371 |
| + | + | + | + |  | + | + |  |  |  |  | 11 | -218.49 | 460.35 | 1.073 | 0.217 |
| + | + | + | + |  |  |  |  |  |  |  | 7 | -223.37 | 461.30 | 2.021 | 0.135 |
| + | + | + | + |  |  | + |  |  |  |  | 10 | -220.09 | 461.32 | 2.035 | 0.134 |
| + | + | + |  |  |  |  |  |  | + |  | 7 | -224.45 | 463.46 | 4.184 | 0.046 |
| + | + | + | + | + |  | + |  |  |  |  | 13 | -218.50 | 464.91 | 5.626 | 0.022 |
| + | + | + | + | + | + | + |  |  |  |  | 14 | -217.38 | 464.97 | 5.693 | 0.022 |
| + | + | + | + | + | + |  |  |  |  |  | 11 | -221.08 | 465.54 | 6.259 | 0.016 |
| + | + | + |  |  |  |  |  | + |  |  | 7 | -225.55 | 465.67 | 6.389 | 0.015 |
| + |  | + | + |  |  |  |  |  |  |  | 6 | -227.34 | 467.12 | 7.835 | 0.007 |
| + | + | + | + | + |  |  |  |  |  |  | 10 | -223.25 | 467.63 | 8.354 | 0.006 |
| + |  | + | + |  |  | + |  |  |  |  | 9 | -224.74 | 468.41 | 9.126 | 0.004 |
| + | + | + |  | + |  |  |  |  | + |  | 10 | -224.20 | 469.54 | 10.257 | 0.002 |
| + | + | + | + | + | + | + | + |  |  |  | 17 | -217.00 | 471.30 | 12.018 | 0.001 |
| + | + | + |  | + |  |  |  | + |  |  | 10 | -225.41 | 471.96 | 12.675 | 0.001 |
| + |  | + |  |  |  |  |  |  | + |  | 6 | -231.81 | 476.05 | 16.774 | 0 |
| + |  | + |  |  |  |  |  | + |  |  | 6 | -232.31 | 477.05 | 17.765 | 0 |
| + | + | + |  |  |  |  |  |  |  |  | 6 | -232.85 | 478.12 | 18.843 | 0 |
| + | + | + |  | + |  |  |  |  |  |  | 9 | -232.67 | 484.27 | 24.990 | 0 |
| + |  | + |  |  |  |  |  |  |  |  | 5 | -242.64 | 495.58 | 36.301 | 0 |
| + | + |  |  |  |  |  |  |  |  |  | 3 | -314.68 | 635.48 | 176.197 | 0 |
| + | + |  |  |  |  |  |  |  | + |  | 4 | -314.15 | 636.51 | 177.227 | 0 |
| + | + |  | + |  |  |  |  |  |  |  | 4 | -314.39 | 636.97 | 177.691 | 0 |
| + | + |  |  |  |  |  |  | + |  |  | 4 | -314.49 | 637.18 | 177.902 | 0 |
| + |  |  |  |  |  |  |  |  |  |  | 2 | -316.71 | 637.48 | 178.197 | 0 |
| + | + |  | + |  | + |  |  |  |  |  | 5 | -313.83 | 637.96 | 178.684 | 0 |
| + |  |  |  |  |  |  |  |  | + |  | 3 | -316.55 | 639.23 | 179.945 | 0 |
| + |  |  |  |  |  |  |  | + |  |  | 3 | -316.71 | 639.53 | 180.250 | 0 |
| + |  |  | + |  |  |  |  |  |  |  | 3 | -316.71 | 639.53 | 180.253 | 0 |

**Supplemental Table 5.** Model selection table of models that explain variation in the centroid latitude of the 50% isopleth of kernel density estimator-based utilization distributions of band recoveries from Blue-winged Teal, Mallards, and Northern Pintail in the Central and Mississippi Flyways of North America between 1960 and 2019. Model structure terms include year (continuous), month (October–January; categorical), Joint Venture of banding origin (JVB), banding latitude, banding longitude, and several possible two-way and three-way interaction terms. Model statistics include the number of parameters (K), the log likelihood, AIC_c_ values, difference in AIC_c_ values compared to the model with the lowest AIC_c_ value (ΔAICc), and the model weight.

| **Blue-winged Teal – Model structure** | | | | | | | | | |  | **Model statistics** | | | | |
| --- | --- | --- | --- | --- | --- | --- | --- | --- | --- | --- | --- | --- | --- | --- | --- |
| **Intercept** | **Year** | **Month** | **JVB** | **Year × Month** | **Year × JVB** | **Month × JVB** | **Year × Month × JVB** | **Banding latitude** | **Banding longitude** |  | **K** | **Log likelihood** | **AICc** | **ΔAICc** | **Model weight** |
| + | + | + |  | + |  |  |  |  |  |  | 5 | -314.45 | 639.39 | 0 | 0.281 |
| + | + | + | + | + |  |  |  |  |  |  | 6 | -313.72 | 640.12 | 0.732 | 0.195 |
| + | + | + |  | + |  |  |  | + |  |  | 6 | -313.82 | 640.33 | 0.940 | 0.176 |
| + | + | + | + | + |  | + |  |  |  |  | 7 | -312.99 | 640.90 | 1.515 | 0.132 |
| + | + | + |  | + |  |  |  |  | + |  | 6 | -314.45 | 641.58 | 2.192 | 0.094 |
| + | + | + | + | + | + |  |  |  |  |  | 7 | -313.72 | 642.36 | 2.968 | 0.064 |
| + | + | + | + | + | + | + |  |  |  |  | 8 | -312.95 | 643.09 | 3.702 | 0.044 |
| + | + | + | + | + | + | + | + |  |  |  | 9 | -312.92 | 645.36 | 5.968 | 0.014 |
| + | + | + |  |  |  |  |  |  |  |  | 4 | -325.38 | 659.08 | 19.686 | 0 |
| + | + | + | + |  |  | + |  |  |  |  | 6 | -323.45 | 659.58 | 20.192 | 0 |
| + | + | + | + |  |  |  |  |  |  |  | 5 | -324.94 | 660.37 | 20.984 | 0 |
| + | + | + |  |  |  |  |  |  | + |  | 5 | -325.11 | 660.71 | 21.321 | 0 |
| + | + | + |  |  |  |  |  | + |  |  | 5 | -325.13 | 660.75 | 21.365 | 0 |
| + | + | + | + |  | + | + |  |  |  |  | 7 | -322.99 | 660.91 | 21.522 | 0 |
| + | + | + | + |  | + |  |  |  |  |  | 6 | -324.76 | 662.21 | 22.825 | 0 |
| + |  | + |  |  |  |  |  |  | + |  | 4 | -333.44 | 675.20 | 35.815 | 0 |
| + |  | + |  |  |  |  |  |  |  |  | 3 | -335.50 | 677.20 | 37.810 | 0 |
| + |  | + | + |  |  | + |  |  |  |  | 5 | -333.40 | 677.28 | 37.894 | 0 |
| + |  | + | + |  |  |  |  |  |  |  | 4 | -334.49 | 677.31 | 37.920 | 0 |
| + |  | + |  |  |  |  |  | + |  |  | 4 | -335.31 | 678.95 | 39.560 | 0 |
| + | + |  |  |  |  |  |  |  | + |  | 4 | -413.36 | 835.05 | 195.656 | 0 |
| + |  |  |  |  |  |  |  |  | + |  | 3 | -416.66 | 839.51 | 200.120 | 0 |
| + | + |  |  |  |  |  |  |  |  |  | 3 | -420.20 | 846.59 | 207.200 | 0 |
| + | + |  |  |  |  |  |  | + |  |  | 4 | -419.97 | 848.26 | 208.872 | 0 |
| + | + |  | + |  |  |  |  |  |  |  | 4 | -420.10 | 848.53 | 209.141 | 0 |
| + | + |  | + |  | + |  |  |  |  |  | 5 | -420.03 | 850.54 | 211.149 | 0 |
| + |  |  |  |  |  |  |  |  |  |  | 2 | -427.74 | 859.58 | 220.186 | 0 |
| + |  |  |  |  |  |  |  | + |  |  | 3 | -427.47 | 861.14 | 221.751 | 0 |
| + |  |  | + |  |  |  |  |  |  |  | 3 | -427.74 | 861.67 | 222.282 | 0 |

| **Mallard – Model structure** | | | | | | | | | |  | **Model statistics** | | | | |
| --- | --- | --- | --- | --- | --- | --- | --- | --- | --- | --- | --- | --- | --- | --- | --- |
| **Intercept** | **Year** | **Month** | **JVB** | **Year × Month** | **Year × JVB** | **Month × JVB** | **Year × Month × JVB** | **Banding latitude** | **Banding longitude** |  | **K** | **Log likelihood** | **AICc** | **ΔAICc** | **Model weight** |
| + | + | + | + | + | + | + | + |  |  |  | 25 | -1130.55 | 2313.03 | 0 | 1 |
| + | + | + | + | + | + | + |  |  |  |  | 19 | -1155.98 | 2351.08 | 38.043 | 0 |
| + | + | + | + | + |  | + |  |  |  |  | 17 | -1165.15 | 2365.21 | 52.173 | 0 |
| + | + | + | + |  | + | + |  |  |  |  | 16 | -1204.79 | 2442.37 | 129.336 | 0 |
| + | + | + | + |  |  | + |  |  |  |  | 14 | -1212.93 | 2454.47 | 141.437 | 0 |
| + |  | + | + |  |  | + |  |  |  |  | 13 | -1221.28 | 2469.09 | 156.055 | 0 |
| + | + | + | + | + | + |  |  |  |  |  | 13 | -1347.22 | 2720.97 | 407.939 | 0 |
| + | + | + |  | + |  |  |  |  | + |  | 10 | -1351.77 | 2723.85 | 410.817 | 0 |
| + | + | + | + | + |  |  |  |  |  |  | 11 | -1352.33 | 2727.04 | 414.005 | 0 |
| + | + | + |  | + |  |  |  | + |  |  | 10 | -1360.51 | 2741.33 | 428.298 | 0 |
| + | + | + | + |  | + |  |  |  |  |  | 10 | -1376.42 | 2773.15 | 460.119 | 0 |
| + | + | + |  |  |  |  |  |  | + |  | 7 | -1381.90 | 2777.96 | 464.928 | 0 |
| + | + | + | + |  |  |  |  |  |  |  | 8 | -1381.19 | 2778.59 | 465.559 | 0 |
| + |  | + | + |  |  |  |  |  |  |  | 7 | -1386.42 | 2787.00 | 473.962 | 0 |
| + |  | + |  |  |  |  |  |  | + |  | 6 | -1388.07 | 2788.25 | 475.220 | 0 |
| + | + | + |  |  |  |  |  | + |  |  | 7 | -1388.68 | 2791.52 | 478.492 | 0 |
| + |  | + |  |  |  |  |  | + |  |  | 6 | -1394.61 | 2801.33 | 488.301 | 0 |
| + | + | + |  | + |  |  |  |  |  |  | 9 | -1501.37 | 3021.00 | 707.969 | 0 |
| + | + | + |  |  |  |  |  |  |  |  | 6 | -1520.22 | 3052.57 | 739.535 | 0 |
| + |  | + |  |  |  |  |  |  |  |  | 5 | -1523.64 | 3057.36 | 744.324 | 0 |
| + |  |  |  |  |  |  |  |  | + |  | 3 | -1946.15 | 3898.34 | 1585.309 | 0 |
| + |  |  |  |  |  |  |  | + |  |  | 3 | -1946.71 | 3899.46 | 1586.428 | 0 |
| + | + |  |  |  |  |  |  |  | + |  | 4 | -1945.98 | 3900.03 | 1586.993 | 0 |
| + | + |  |  |  |  |  |  | + |  |  | 4 | -1946.55 | 3901.15 | 1588.117 | 0 |
| + |  |  | + |  |  |  |  |  |  |  | 4 | -1949.51 | 3907.07 | 1594.041 | 0 |
| + | + |  | + |  |  |  |  |  |  |  | 5 | -1949.40 | 3908.90 | 1595.863 | 0 |
| + | + |  | + |  | + |  |  |  |  |  | 7 | -1948.39 | 3910.95 | 1597.912 | 0 |
| + |  |  |  |  |  |  |  |  |  |  | 2 | -1981.31 | 3966.64 | 1653.606 | 0 |
| + | + |  |  |  |  |  |  |  |  |  | 3 | -1981.22 | 3968.48 | 1655.450 | 0 |

| **Northern Pintail – Model structure** | | | | | | | | | |  | **Model statistics** | | | | |
| --- | --- | --- | --- | --- | --- | --- | --- | --- | --- | --- | --- | --- | --- | --- | --- |
| **Intercept** | **Year** | **Month** | **JVB** | **Year × Month** | **Year × JVB** | **Month × JVB** | **Year × Month × JVB** | **Banding latitude** | **Banding longitude** |  | **K** | **Log likelihood** | **AICc** | **ΔAICc** | **Model weight** |
| + | + | + | + | + | + | + |  |  |  |  | 14 | -381.52 | 793.27 | 0 | 0.566 |
| + | + | + | + | + |  | + |  |  |  |  | 13 | -383.52 | 794.96 | 1.693 | 0.243 |
| + | + | + | + | + | + | + | + |  |  |  | 17 | -379.08 | 795.46 | 2.191 | 0.189 |
| + | + | + | + |  |  | + |  |  |  |  | 10 | -392.53 | 806.21 | 12.94 | 0.001 |
| + | + | + | + |  | + | + |  |  |  |  | 11 | -391.79 | 806.96 | 13.694 | 0.001 |
| + | + | + |  | + |  |  |  |  | + |  | 10 | -397.19 | 815.51 | 22.246 | 0 |
| + | + | + | + | + |  |  |  |  |  |  | 10 | -397.60 | 816.33 | 23.064 | 0 |
| + | + | + | + | + | + |  |  |  |  |  | 11 | -396.89 | 817.15 | 23.883 | 0 |
| + | + | + |  |  |  |  |  |  | + |  | 7 | -401.68 | 817.93 | 24.660 | 0 |
| + | + | + |  | + |  |  |  | + |  |  | 10 | -398.49 | 818.13 | 24.858 | 0 |
| + | + | + | + |  |  |  |  |  |  |  | 7 | -402.73 | 820.04 | 26.769 | 0 |
| + | + | + | + |  | + |  |  |  |  |  | 8 | -401.98 | 820.70 | 27.429 | 0 |
| + | + | + |  |  |  |  |  | + |  |  | 7 | -403.13 | 820.84 | 27.568 | 0 |
| + | + | + |  | + |  |  |  |  |  |  | 9 | -406.67 | 832.27 | 39.002 | 0 |
| + |  | + | + |  |  | + |  |  |  |  | 9 | -407.65 | 834.23 | 40.963 | 0 |
| + | + | + |  |  |  |  |  |  |  |  | 6 | -411.98 | 836.39 | 43.124 | 0 |
| + |  | + | + |  |  |  |  |  |  |  | 6 | -417.41 | 847.24 | 53.975 | 0 |
| + |  | + |  |  |  |  |  |  | + |  | 6 | -422.44 | 857.30 | 64.029 | 0 |
| + |  | + |  |  |  |  |  | + |  |  | 6 | -422.63 | 857.69 | 64.424 | 0 |
| + |  | + |  |  |  |  |  |  |  |  | 5 | -435.95 | 882.20 | 88.931 | 0 |
| + | + |  |  |  |  |  |  |  | + |  | 4 | -549.81 | 1107.82 | 314.552 | 0 |
| + | + |  | + |  |  |  |  |  |  |  | 4 | -551.76 | 1111.72 | 318.456 | 0 |
| + | + |  | + |  | + |  |  |  |  |  | 5 | -551.40 | 1113.11 | 319.844 | 0 |
| + | + |  |  |  |  |  |  | + |  |  | 4 | -554.94 | 1118.08 | 324.809 | 0 |
| + |  |  | + |  |  |  |  |  |  |  | 3 | -556.41 | 1118.94 | 325.675 | 0 |
| + |  |  |  |  |  |  |  |  | + |  | 3 | -559.26 | 1124.64 | 331.376 | 0 |
| + |  |  |  |  |  |  |  | + |  |  | 3 | -563.57 | 1133.27 | 340.000 | 0 |
| + | + |  |  |  |  |  |  |  |  |  | 3 | -570.96 | 1148.05 | 354.781 | 0 |
| + |  |  |  |  |  |  |  |  |  |  | 2 | -584.93 | 1173.92 | 380.647 | 0 |

**Supplemental Table 6.** Model selection table of models that explain variation in the centroid latitude of the 95% isopleth of kernel density estimator-based utilization distributions of band recoveries from Blue-winged Teal, Mallards, and Northern Pintail in the Central and Mississippi Flyways of North America between 1960 and 2019. Model structure terms include year (continuous), month (October–January; categorical), Joint Venture of banding origin (JVB), banding latitude, banding longitude, and several possible two-way and three-way interaction terms. Model statistics include the number of parameters (K), the log likelihood, AIC_c_ values, difference in AIC_c_ values compared to the model with the lowest AIC_c_ value (ΔAICc), and the model weight.

| **Blue-winged Teal – Model structure** | | | | | | | | | |  | **Model statistics** | | | | |
| --- | --- | --- | --- | --- | --- | --- | --- | --- | --- | --- | --- | --- | --- | --- | --- |
| **Intercept** | **Year** | **Month** | **JVB** | **Year × Month** | **Year × JVB** | **Month × JVB** | **Year × Month × JVB** | **Banding latitude** | **Banding longitude** |  | **K** | **Log likelihood** | **AICc** | **ΔAICc** | **Model weight** |
| + | + | + |  | + |  |  |  |  | + |  | 6 | -247.19 | 507.07 | 0 | 0.241 |
| + | + | + |  | + |  |  |  |  |  |  | 5 | -248.44 | 507.37 | 0.293 | 0.208 |
| + | + | + | + | + |  | + |  |  |  |  | 7 | -246.39 | 507.71 | 0.640 | 0.175 |
| + | + | + |  | + |  |  |  | + |  |  | 6 | -248.35 | 509.39 | 2.313 | 0.076 |
| + | + | + | + | + |  |  |  |  |  |  | 6 | -248.36 | 509.42 | 2.344 | 0.075 |
| + | + | + | + | + | + | + |  |  |  |  | 8 | -246.31 | 509.81 | 2.740 | 0.061 |
| + | + | + |  |  |  |  |  |  | + |  | 5 | -250.15 | 510.78 | 3.704 | 0.038 |
| + | + | + | + |  |  | + |  |  |  |  | 6 | -249.23 | 511.15 | 4.078 | 0.031 |
| + | + | + | + | + | + |  |  |  |  |  | 7 | -248.36 | 511.66 | 4.581 | 0.024 |
| + | + | + | + | + | + | + | + |  |  |  | 9 | -246.25 | 512.02 | 4.941 | 0.020 |
| + | + | + |  |  |  |  |  |  |  |  | 4 | -251.95 | 512.23 | 5.152 | 0.018 |
| + | + | + | + |  | + | + |  |  |  |  | 7 | -248.94 | 512.80 | 5.722 | 0.014 |
| + | + | + |  |  |  |  |  | + |  |  | 5 | -251.77 | 514.03 | 6.951 | 0.007 |
| + | + | + | + |  |  |  |  |  |  |  | 5 | -251.84 | 514.16 | 7.085 | 0.007 |
| + | + | + | + |  | + |  |  |  |  |  | 6 | -251.79 | 516.26 | 9.187 | 0.002 |
| + |  | + |  |  |  |  |  |  | + |  | 4 | -262.82 | 533.95 | 26.88 | 0 |
| + |  | + |  |  |  |  |  |  |  |  | 3 | -268.48 | 543.14 | 36.068 | 0 |
| + |  | + | + |  |  | + |  |  |  |  | 5 | -266.74 | 543.97 | 36.897 | 0 |
| + |  | + |  |  |  |  |  | + |  |  | 4 | -268.31 | 544.95 | 37.876 | 0 |
| + |  | + | + |  |  |  |  |  |  |  | 4 | -268.44 | 545.19 | 38.118 | 0 |
| + | + |  |  |  |  |  |  |  | + |  | 4 | -399.07 | 806.46 | 299.382 | 0 |
| + |  |  |  |  |  |  |  |  | + |  | 3 | -401.42 | 809.03 | 301.952 | 0 |
| + | + |  |  |  |  |  |  |  |  |  | 3 | -408.03 | 822.25 | 315.177 | 0 |
| + | + |  |  |  |  |  |  | + |  |  | 4 | -407.21 | 822.74 | 315.667 | 0 |
| + | + |  | + |  |  |  |  |  |  |  | 4 | -407.42 | 823.17 | 316.094 | 0 |
| + | + |  | + |  | + |  |  |  |  |  | 5 | -407.40 | 825.29 | 318.214 | 0 |
| + |  |  |  |  |  |  |  |  |  |  | 2 | -414.64 | 833.37 | 326.291 | 0 |
| + |  |  |  |  |  |  |  | + |  |  | 3 | -413.79 | 833.77 | 326.691 | 0 |
| + |  |  | + |  |  |  |  |  |  |  | 3 | -414.44 | 835.07 | 327.998 | 0 |

| **Mallard – Model structure** | | | | | | | | | |  | **Model statistics** | | | | |
| --- | --- | --- | --- | --- | --- | --- | --- | --- | --- | --- | --- | --- | --- | --- | --- |
| **Intercept** | **Year** | **Month** | **JVB** | **Year × Month** | **Year × JVB** | **Month × JVB** | **Year × Month × JVB** | **Banding latitude** | **Banding longitude** |  | **K** | **Log likelihood** | **AICc** | **ΔAICc** | **Model weight** |
| + | + | + | + | + | + | + | + |  |  |  | 25 | -867.48 | 1786.88 | 0 | 0.985 |
| + | + | + | + | + |  | + |  |  |  |  | 17 | -880.59 | 1796.07 | 9.190 | 0.010 |
| + | + | + | + | + | + | + |  |  |  |  | 19 | -879.15 | 1797.42 | 10.540 | 0.005 |
| + | + | + | + | + |  |  |  |  |  |  | 11 | -941.05 | 1904.49 | 117.609 | 0 |
| + | + | + | + | + | + |  |  |  |  |  | 13 | -939.38 | 1905.28 | 118.403 | 0 |
| + | + | + |  | + |  |  |  | + |  |  | 10 | -943.32 | 1906.96 | 120.076 | 0 |
| + | + | + |  | + |  |  |  |  | + |  | 10 | -944.65 | 1909.62 | 122.742 | 0 |
| + | + | + | + |  |  | + |  |  |  |  | 14 | -986.51 | 2001.63 | 214.748 | 0 |
| + | + | + | + |  | + | + |  |  |  |  | 16 | -985.36 | 2003.51 | 216.633 | 0 |
| + | + | + |  | + |  |  |  |  |  |  | 9 | -1026.17 | 2070.60 | 283.719 | 0 |
| + |  | + | + |  |  | + |  |  |  |  | 13 | -1024.28 | 2075.09 | 288.213 | 0 |
| + | + | + | + |  |  |  |  |  |  |  | 8 | -1032.08 | 2080.37 | 293.493 | 0 |
| + | + | + | + |  | + |  |  |  |  |  | 10 | -1030.72 | 2081.76 | 294.881 | 0 |
| + | + | + |  |  |  |  |  | + |  |  | 7 | -1034.51 | 2083.18 | 296.301 | 0 |
| + | + | + |  |  |  |  |  |  | + |  | 7 | -1036.65 | 2087.46 | 300.579 | 0 |
| + |  | + | + |  |  |  |  |  |  |  | 7 | -1065.43 | 2145.01 | 358.133 | 0 |
| + |  | + |  |  |  |  |  | + |  |  | 6 | -1068.89 | 2149.90 | 363.020 | 0 |
| + |  | + |  |  |  |  |  |  | + |  | 6 | -1070.95 | 2154.03 | 367.147 | 0 |
| + | + | + |  |  |  |  |  |  |  |  | 6 | -1098.96 | 2210.04 | 423.158 | 0 |
| + |  | + |  |  |  |  |  |  |  |  | 5 | -1126.52 | 2263.12 | 476.242 | 0 |
| + | + |  |  |  |  |  |  | + |  |  | 4 | -1808.72 | 3625.49 | 1838.612 | 0 |
| + |  |  |  |  |  |  |  | + |  |  | 3 | -1809.90 | 3625.84 | 1838.959 | 0 |
| + | + |  |  |  |  |  |  |  | + |  | 4 | -1809.73 | 3627.51 | 1840.633 | 0 |
| + |  |  |  |  |  |  |  |  | + |  | 3 | -1810.91 | 3627.85 | 1840.965 | 0 |
| + | + |  | + |  |  |  |  |  |  |  | 5 | -1810.53 | 3631.14 | 1844.256 | 0 |
| + |  |  | + |  |  |  |  |  |  |  | 4 | -1811.61 | 3631.28 | 1844.396 | 0 |
| + | + |  | + |  | + |  |  |  |  |  | 7 | -1810.34 | 3634.85 | 1847.968 | 0 |
| + | + |  |  |  |  |  |  |  |  |  | 3 | -1818.20 | 3642.43 | 1855.545 | 0 |
| + |  |  |  |  |  |  |  |  |  |  | 2 | -1819.24 | 3642.50 | 1855.622 | 0 |

| **Northern Pintail – Model structure** | | | | | | | | | |  | **Model statistics** | | | | |
| --- | --- | --- | --- | --- | --- | --- | --- | --- | --- | --- | --- | --- | --- | --- | --- |
| **Intercept** | **Year** | **Month** | **JVB** | **Year × Month** | **Year × JVB** | **Month × JVB** | **Year × Month × JVB** | **Banding latitude** | **Banding longitude** |  | **K** | **Log likelihood** | **AICc** | **ΔAICc** | **Model weight** |
| + | + | + | + | + | + | + | + |  |  |  | 17 | -297.01 | 631.31 | 0 | 0.433 |
| + | + | + | + | + |  | + |  |  |  |  | 13 | -302.22 | 632.35 | 1.045 | 0.257 |
| + | + | + | + | + | + | + |  |  |  |  | 14 | -302.11 | 634.45 | 3.138 | 0.090 |
| + | + | + | + |  |  | + |  |  |  |  | 10 | -306.81 | 634.76 | 3.449 | 0.077 |
| + | + | + |  |  |  |  |  |  | + |  | 7 | -310.57 | 635.70 | 4.393 | 0.048 |
| + | + | + | + |  |  |  |  |  |  |  | 7 | -310.79 | 636.15 | 4.839 | 0.039 |
| + | + | + | + |  | + | + |  |  |  |  | 11 | -306.81 | 636.99 | 5.682 | 0.025 |
| + | + | + | + |  | + |  |  |  |  |  | 8 | -310.77 | 638.28 | 6.974 | 0.013 |
| + | + | + |  |  |  |  |  | + |  |  | 7 | -312.19 | 638.95 | 7.640 | 0.009 |
| + | + | + |  | + |  |  |  |  | + |  | 10 | -310.02 | 641.18 | 9.869 | 0.003 |
| + | + | + | + | + |  |  |  |  |  |  | 10 | -310.04 | 641.22 | 9.913 | 0.003 |
| + | + | + | + | + | + |  |  |  |  |  | 11 | -310.04 | 643.45 | 12.137 | 0.001 |
| + | + | + |  | + |  |  |  | + |  |  | 10 | -311.48 | 644.11 | 12.799 | 0.001 |
| + | + | + |  |  |  |  |  |  |  |  | 6 | -318.09 | 648.60 | 17.294 | 0 |
| + | + | + |  | + |  |  |  |  |  |  | 9 | -317.36 | 653.65 | 22.343 | 0 |
| + |  | + | + |  |  | + |  |  |  |  | 9 | -330.29 | 679.51 | 48.205 | 0 |
| + |  | + | + |  |  |  |  |  |  |  | 6 | -334.79 | 682.01 | 50.706 | 0 |
| + |  | + |  |  |  |  |  |  | + |  | 6 | -341.62 | 695.67 | 64.365 | 0 |
| + |  | + |  |  |  |  |  | + |  |  | 6 | -341.94 | 696.31 | 64.999 | 0 |
| + |  | + |  |  |  |  |  |  |  |  | 5 | -352.38 | 715.06 | 83.756 | 0 |
| + | + |  |  |  |  |  |  |  | + |  | 4 | -520.83 | 1049.86 | 418.555 | 0 |
| + | + |  | + |  |  |  |  |  |  |  | 4 | -521.86 | 1051.93 | 420.619 | 0 |
| + | + |  | + |  | + |  |  |  |  |  | 5 | -521.82 | 1053.94 | 422.631 | 0 |
| + |  |  | + |  |  |  |  |  |  |  | 3 | -524.51 | 1055.14 | 423.831 | 0 |
| + |  |  |  |  |  |  |  |  | + |  | 3 | -526.84 | 1059.80 | 428.490 | 0 |
| + | + |  |  |  |  |  |  | + |  |  | 4 | -525.91 | 1060.02 | 428.707 | 0 |
| + |  |  |  |  |  |  |  | + |  |  | 3 | -531.54 | 1069.20 | 437.892 | 0 |
| + | + |  |  |  |  |  |  |  |  |  | 3 | -537.69 | 1081.49 | 450.183 | 0 |
| + |  |  |  |  |  |  |  |  |  |  | 2 | -547.52 | 1099.11 | 467.800 | 0 |

**Supplemental Table 7.** Model selection table of models that explain variation in the natural logarithm of relative area (compared to 1960–1979 base line period) of the 50% isopleth of kernel density estimator-based utilization distributions of band recoveries from Blue-winged Teal, Mallards, and Northern Pintail in the Central and Mississippi Flyways of North America between 1960 and 2019. Model structure terms include year (continuous), month (October–January; categorical), Joint Venture of banding origin (JVB), banding latitude, banding longitude, and several possible two-way and three-way interaction terms. Model statistics include the number of parameters (K), the log likelihood, AIC_c_ values, difference in AIC_c_ values compared to the model with the lowest AIC_c_ value (ΔAICc), and the model weight.

| **Blue-winged Teal – Model structure** | | | | | | | | | |  | **Model statistics** | | | | |
| --- | --- | --- | --- | --- | --- | --- | --- | --- | --- | --- | --- | --- | --- | --- | --- |
| **Intercept** | **Year** | **Month** | **JVB** | **Year × Month** | **Year × JVB** | **Month × JVB** | **Year × Month × JVB** | **Banding latitude** | **Banding longitude** |  | **K** | **Log likelihood** | **AICc** | **ΔAICc** | **Model weight** |
| + | + | + | + | + | + |  |  |  |  |  | 7 | -93.56 | 202.05 | 0 | 0.476 |
| + | + | + | + | + | + | + |  |  |  |  | 8 | -93.37 | 203.95 | 1.891 | 0.185 |
| + | + | + | + | + |  |  |  |  |  |  | 6 | -96.09 | 204.86 | 2.807 | 0.117 |
| + | + | + | + | + | + | + | + |  |  |  | 9 | -93.19 | 205.90 | 3.847 | 0.070 |
| + | + | + | + | + |  | + |  |  |  |  | 7 | -95.52 | 205.97 | 3.918 | 0.067 |
| + | + | + | + |  | + |  |  |  |  |  | 6 | -97.14 | 206.98 | 4.922 | 0.041 |
| + | + | + |  | + |  |  |  | + |  |  | 6 | -97.78 | 208.24 | 6.188 | 0.022 |
| + | + | + | + |  | + | + |  |  |  |  | 7 | -97.13 | 209.19 | 7.136 | 0.013 |
| + | + | + | + |  |  |  |  |  |  |  | 5 | -100.33 | 211.14 | 9.091 | 0.005 |
| + | + | + | + |  |  | + |  |  |  |  | 6 | -100.14 | 212.96 | 10.909 | 0.002 |
| + | + | + |  | + |  |  |  |  | + |  | 6 | -100.66 | 214.01 | 11.954 | 0.001 |
| + | + |  | + |  | + |  |  |  |  |  | 5 | -101.93 | 214.35 | 12.300 | 0.001 |
| + | + | + |  |  |  |  |  | + |  |  | 5 | -102.30 | 215.09 | 13.035 | 0.001 |
| + | + |  | + |  |  |  |  |  |  |  | 4 | -104.82 | 217.96 | 15.902 | 0 |
| + | + |  |  |  |  |  |  | + |  |  | 4 | -106.59 | 221.51 | 19.454 | 0 |
| + | + | + |  |  |  |  |  |  | + |  | 5 | -105.59 | 221.66 | 19.609 | 0 |
| + | + |  |  |  |  |  |  |  | + |  | 4 | -107.65 | 223.63 | 21.574 | 0 |
| + | + | + |  | + |  |  |  |  |  |  | 5 | -108.04 | 226.56 | 24.508 | 0 |
| + | + | + |  |  |  |  |  |  |  |  | 4 | -111.12 | 230.57 | 28.514 | 0 |
| + | + |  |  |  |  |  |  |  |  |  | 3 | -116.08 | 238.35 | 36.296 | 0 |
| + |  | + | + |  |  |  |  |  |  |  | 4 | -119.28 | 246.87 | 44.820 | 0 |
| + |  |  | + |  |  |  |  |  |  |  | 3 | -120.59 | 247.37 | 45.320 | 0 |
| + |  | + | + |  |  | + |  |  |  |  | 5 | -119.03 | 248.55 | 46.495 | 0 |
| + |  | + |  |  |  |  |  | + |  |  | 4 | -124.33 | 256.99 | 54.933 | 0 |
| + |  |  |  |  |  |  |  | + |  |  | 3 | -125.45 | 257.09 | 55.041 | 0 |
| + |  | + |  |  |  |  |  |  |  |  | 3 | -130.59 | 267.38 | 65.323 | 0 |
| + |  |  |  |  |  |  |  |  |  |  | 2 | -132.19 | 268.48 | 66.425 | 0 |
| + |  |  |  |  |  |  |  |  | + |  | 3 | -131.22 | 268.62 | 66.571 | 0 |
| + |  | + |  |  |  |  |  |  | + |  | 4 | -130.26 | 268.85 | 66.793 | 0 |

| **Mallard – Model structure** | | | | | | | | | |  | **Model statistics** | | | | |
| --- | --- | --- | --- | --- | --- | --- | --- | --- | --- | --- | --- | --- | --- | --- | --- |
| **Intercept** | **Year** | **Month** | **JVB** | **Year × Month** | **Year × JVB** | **Month × JVB** | **Year × Month × JVB** | **Banding latitude** | **Banding longitude** |  | **K** | **Log likelihood** | **AICc** | **ΔAICc** | **Model weight** |
| + | + | + | + | + | + |  |  |  |  |  | 13 | -257.49 | 541.52 | 0 | 0.657 |
| + | + | + | + | + |  |  |  |  |  |  | 11 | -260.30 | 542.97 | 1.457 | 0.317 |
| + | + | + |  | + |  |  |  |  |  |  | 9 | -266.07 | 550.40 | 8.878 | 0.008 |
| + | + | + |  | + |  |  |  | + |  |  | 10 | -265.08 | 550.48 | 8.967 | 0.007 |
| + | + | + |  | + |  |  |  |  | + |  | 10 | -265.70 | 551.72 | 10.204 | 0.004 |
| + | + | + | + | + | + | + |  |  |  |  | 19 | -256.62 | 552.35 | 10.831 | 0.003 |
| + | + | + | + | + | + | + | + |  |  |  | 25 | -250.62 | 553.18 | 11.661 | 0.002 |
| + | + | + | + | + |  | + |  |  |  |  | 17 | -259.27 | 553.44 | 11.919 | 0.002 |
| + | + | + | + |  | + |  |  |  |  |  | 10 | -278.03 | 576.38 | 34.864 | 0 |
| + | + | + | + |  |  |  |  |  |  |  | 8 | -280.67 | 577.56 | 36.038 | 0 |
| + | + | + |  |  |  |  |  |  |  |  | 6 | -286.12 | 584.37 | 42.849 | 0 |
| + | + | + |  |  |  |  |  | + |  |  | 7 | -285.14 | 584.44 | 42.921 | 0 |
| + | + | + |  |  |  |  |  |  | + |  | 7 | -285.78 | 585.73 | 44.211 | 0 |
| + | + | + | + |  | + | + |  |  |  |  | 16 | -277.20 | 587.20 | 45.685 | 0 |
| + | + | + | + |  |  | + |  |  |  |  | 14 | -279.71 | 588.02 | 46.508 | 0 |
| + | + |  | + |  | + |  |  |  |  |  | 7 | -287.06 | 588.27 | 46.755 | 0 |
| + | + |  | + |  |  |  |  |  |  |  | 5 | -289.63 | 589.35 | 47.828 | 0 |
| + | + |  |  |  |  |  |  |  |  |  | 3 | -294.94 | 595.92 | 54.405 | 0 |
| + | + |  |  |  |  |  |  | + |  |  | 4 | -293.94 | 595.93 | 54.414 | 0 |
| + | + |  |  |  |  |  |  |  | + |  | 4 | -294.60 | 597.26 | 55.743 | 0 |
| + |  | + | + |  |  |  |  |  |  |  | 7 | -299.09 | 612.34 | 70.819 | 0 |
| + |  | + |  |  |  |  |  | + |  |  | 6 | -303.18 | 618.48 | 76.960 | 0 |
| + |  | + |  |  |  |  |  |  |  |  | 5 | -304.25 | 618.59 | 77.075 | 0 |
| + |  | + |  |  |  |  |  |  | + |  | 6 | -303.84 | 619.80 | 78.279 | 0 |
| + |  | + | + |  |  | + |  |  |  |  | 13 | -298.17 | 622.87 | 81.354 | 0 |
| + |  |  | + |  |  |  |  |  |  |  | 4 | -307.99 | 624.03 | 82.513 | 0 |
| + |  |  |  |  |  |  |  | + |  |  | 3 | -311.93 | 629.89 | 88.375 | 0 |
| + |  |  |  |  |  |  |  |  |  |  | 2 | -313.03 | 630.07 | 88.553 | 0 |
| + |  |  |  |  |  |  |  |  | + |  | 3 | -312.62 | 631.27 | 89.748 | 0 |

| **Northern Pintail – Model structure** | | | | | | | | | |  | **Model statistics** | | | | |
| --- | --- | --- | --- | --- | --- | --- | --- | --- | --- | --- | --- | --- | --- | --- | --- |
| **Intercept** | **Year** | **Month** | **JVB** | **Year × Month** | **Year × JVB** | **Month × JVB** | **Year × Month × JVB** | **Banding latitude** | **Banding longitude** |  | **K** | **Log likelihood** | **AICc** | **ΔAICc** | **Model weight** |
| + | + |  |  |  |  |  |  |  |  |  | 3 | -75.09 | 156.34 | 0 | 0.350 |
| + | + |  |  |  |  |  |  | + |  |  | 4 | -74.65 | 157.55 | 1.215 | 0.191 |
| + | + |  |  |  |  |  |  |  | + |  | 4 | -74.86 | 157.97 | 1.630 | 0.155 |
| + | + |  | + |  |  |  |  |  |  |  | 4 | -74.96 | 158.17 | 1.830 | 0.140 |
| + | + |  | + |  | + |  |  |  |  |  | 5 | -74.66 | 159.70 | 3.364 | 0.065 |
| + | + | + |  |  |  |  |  |  |  |  | 6 | -74.48 | 161.49 | 5.154 | 0.027 |
| + | + | + |  |  |  |  |  | + |  |  | 7 | -73.69 | 162.11 | 5.769 | 0.020 |
| + | + | + |  |  |  |  |  |  | + |  | 7 | -74.01 | 162.75 | 6.408 | 0.014 |
| + | + | + | + |  |  |  |  |  |  |  | 7 | -74.12 | 162.97 | 6.632 | 0.013 |
| + | + | + | + | + | + |  |  |  |  |  | 8 | -73.76 | 164.45 | 8.113 | 0.006 |
| + | + | + | + |  |  | + |  |  |  |  | 8 | -74.12 | 165.18 | 8.842 | 0.004 |
| + | + | + |  |  |  |  |  |  |  |  | 9 | -73.22 | 165.62 | 9.285 | 0.003 |
| + | + | + |  | + |  |  |  | + |  |  | 10 | -72.23 | 165.92 | 9.580 | 0.003 |
| + | + | + |  | + |  |  |  |  | + |  | 10 | -72.61 | 166.67 | 10.337 | 0.002 |
| + | + | + | + |  | + | + |  |  |  |  | 9 | -73.76 | 166.69 | 10.355 | 0.002 |
| + | + | + | + | + |  |  |  |  |  |  | 10 | -72.64 | 166.72 | 10.381 | 0.002 |
| + | + | + | + | + | + |  |  |  |  |  | 11 | -71.86 | 167.46 | 11.127 | 0.001 |
| + | + | + | + | + |  | + |  |  |  |  | 11 | -72.63 | 169.01 | 12.670 | 0.001 |
| + | + | + | + | + | + | + |  |  |  |  | 12 | -71.86 | 169.80 | 13.459 | 0 |
| + | + | + | + | + | + | + | + |  |  |  | 13 | -71.86 | 172.16 | 15.821 | 0 |
| + |  |  | + |  |  |  |  |  |  |  | 3 | -109.84 | 225.84 | 69.503 | 0 |
| + |  |  |  |  |  |  |  |  |  |  | 2 | -111.42 | 226.91 | 70.574 | 0 |
| + |  |  |  |  |  |  |  | + |  |  | 3 | -111.40 | 228.96 | 72.624 | 0 |
| + |  |  |  |  |  |  |  |  | + |  | 3 | -111.42 | 228.99 | 72.649 | 0 |
| + |  | + | + |  |  |  |  |  |  |  | 6 | -109.15 | 230.84 | 74.499 | 0 |
| + |  | + |  |  |  |  |  |  |  |  | 5 | -110.99 | 232.36 | 76.026 | 0 |
| + |  | + | + |  |  | + |  |  |  |  | 7 | -109.15 | 233.01 | 76.676 | 0 |
| + |  | + |  |  |  |  |  | + |  |  | 6 | -110.97 | 234.49 | 78.149 | 0 |
| + |  | + |  |  |  |  |  |  | + |  | 6 | -110.99 | 234.52 | 78.180 | 0 |

**Supplemental Table 8.** Model selection table of models that explain variation in the natural logarithm of relative area (compared to 1960–1979 base line period) of the 95% isopleth of kernel density estimator-based utilization distributions of band recoveries from Blue-winged Teal, Mallards, and Northern Pintail in the Central and Mississippi Flyways of North America between 1960 and 2019. Model structure terms include year (continuous), month (October–January; categorical), Joint Venture of banding origin (JVB), banding latitude, banding longitude, and several possible two-way and three-way interaction terms. Model statistics include the number of parameters (K), the log likelihood, AIC_c_ values, difference in AIC_c_ values compared to the model with the lowest AIC_c_ value (ΔAICc), and the model weight.

| **Blue-winged Teal – Model structure** | | | | | | | | | |  | **Model statistics** | | | | |
| --- | --- | --- | --- | --- | --- | --- | --- | --- | --- | --- | --- | --- | --- | --- | --- |
| **Intercept** | **Year** | **Month** | **JVB** | **Year × Month** | **Year × JVB** | **Month × JVB** | **Year × Month × JVB** | **Banding latitude** | **Banding longitude** |  | **K** | **Log likelihood** | **AICc** | **ΔAICc** | **Model weight** |
| + | + | + |  | + |  |  |  |  |  |  | 5 | -44.74 | 99.97 | 0 | 0.362 |
| + | + | + |  | + |  |  |  |  | + |  | 6 | -44.66 | 102.01 | 2.037 | 0.131 |
| + | + | + | + | + |  |  |  |  |  |  | 6 | -44.69 | 102.07 | 2.099 | 0.127 |
| + | + | + |  | + |  |  |  | + |  |  | 6 | -44.71 | 102.12 | 2.146 | 0.124 |
| + | + | + | + | + |  | + |  |  |  |  | 7 | -43.60 | 102.13 | 2.160 | 0.123 |
| + | + | + | + | + | + | + |  |  |  |  | 8 | -43.14 | 103.49 | 3.516 | 0.062 |
| + | + | + | + | + | + |  |  |  |  |  | 7 | -44.50 | 103.92 | 3.951 | 0.050 |
| + | + | + | + | + | + | + | + |  |  |  | 9 | -43.14 | 105.79 | 5.815 | 0.020 |
| + | + | + |  |  |  |  |  |  |  |  | 4 | -52.62 | 113.57 | 13.595 | 0 |
| + | + | + | + |  |  | + |  |  |  |  | 6 | -50.78 | 114.24 | 14.268 | 0 |
| + | + | + | + |  | + | + |  |  |  |  | 7 | -49.69 | 114.31 | 14.340 | 0 |
| + | + | + |  |  |  |  |  |  | + |  | 5 | -52.19 | 114.87 | 14.897 | 0 |
| + |  | + |  |  |  |  |  |  |  |  | 3 | -54.34 | 114.88 | 14.909 | 0 |
| + |  | + |  |  |  |  |  |  | + |  | 4 | -53.28 | 114.88 | 14.909 | 0 |
| + |  | + | + |  |  | + |  |  |  |  | 5 | -52.58 | 115.66 | 15.685 | 0 |
| + | + | + | + |  |  |  |  |  |  |  | 5 | -52.61 | 115.71 | 15.739 | 0 |
| + | + | + |  |  |  |  |  | + |  |  | 5 | -52.62 | 115.73 | 15.756 | 0 |
| + | + | + | + |  | + |  |  |  |  |  | 6 | -52.05 | 116.78 | 16.810 | 0 |
| + |  | + | + |  |  |  |  |  |  |  | 4 | -54.27 | 116.87 | 16.894 | 0 |
| + |  | + |  |  |  |  |  | + |  |  | 4 | -54.34 | 117.01 | 17.035 | 0 |
| + |  |  |  |  |  |  |  |  |  |  | 2 | -60.75 | 125.59 | 25.618 | 0 |
| + | + |  |  |  |  |  |  |  |  |  | 3 | -60.10 | 126.39 | 26.421 | 0 |
| + |  |  | + |  |  |  |  |  |  |  | 3 | -60.57 | 127.33 | 27.360 | 0 |
| + |  |  |  |  |  |  |  | + |  |  | 3 | -60.70 | 127.60 | 27.629 | 0 |
| + |  |  |  |  |  |  |  |  | + |  | 3 | -60.75 | 127.69 | 27.715 | 0 |
| + | + |  | + |  |  |  |  |  |  |  | 4 | -59.99 | 128.30 | 28.332 | 0 |
| + | + |  |  |  |  |  |  |  | + |  | 4 | -60.02 | 128.36 | 28.386 | 0 |
| + | + |  |  |  |  |  |  | + |  |  | 4 | -60.05 | 128.42 | 28.448 | 0 |
| + | + |  | + |  | + |  |  |  |  |  | 5 | -59.53 | 129.54 | 29.572 | 0 |

| **Mallard – Model structure** | | | | | | | | | |  | **Model statistics** | | | | |
| --- | --- | --- | --- | --- | --- | --- | --- | --- | --- | --- | --- | --- | --- | --- | --- |
| **Intercept** | **Year** | **Month** | **JVB** | **Year × Month** | **Year × JVB** | **Month × JVB** | **Year × Month × JVB** | **Banding latitude** | **Banding longitude** |  | **K** | **Log likelihood** | **AICc** | **ΔAICc** | **Model weight** |
| + | + | + | + | + | + |  |  |  |  |  | 13 | 230.40 | -434.27 | 0 | 0.895 |
| + | + | + | + | + |  |  |  |  |  |  | 11 | 225.64 | -428.89 | 5.385 | 0.061 |
| + | + | + | + | + | + | + |  |  |  |  | 19 | 233.50 | -427.89 | 6.384 | 0.037 |
| + | + | + | + |  | + |  |  |  |  |  | 10 | 221.60 | -422.88 | 11.389 | 0.003 |
| + | + | + | + | + |  | + |  |  |  |  | 17 | 228.59 | -422.27 | 11.998 | 0.002 |
| + | + | + |  | + |  |  |  | + |  |  | 10 | 220.50 | -420.67 | 13.600 | 0.001 |
| + | + |  | + |  | + |  |  |  |  |  | 7 | 216.13 | -418.10 | 16.171 | 0 |
| + | + | + | + | + | + | + | + |  |  |  | 25 | 234.84 | -417.76 | 16.517 | 0 |
| + | + | + | + |  |  |  |  |  |  |  | 8 | 216.97 | -417.74 | 16.538 | 0 |
| + | + | + |  | + |  |  |  |  | + |  | 10 | 218.76 | -417.21 | 17.067 | 0 |
| + | + | + | + |  | + | + |  |  |  |  | 16 | 224.56 | -416.33 | 17.948 | 0 |
| + | + |  | + |  |  |  |  |  |  |  | 5 | 211.57 | -413.06 | 21.216 | 0 |
| + | + | + |  | + |  |  |  |  |  |  | 9 | 214.67 | -411.07 | 23.203 | 0 |
| + | + | + | + |  |  | + |  |  |  |  | 14 | 219.79 | -410.97 | 23.306 | 0 |
| + | + | + |  |  |  |  |  | + |  |  | 7 | 212.08 | -410.00 | 24.275 | 0 |
| + | + | + |  |  |  |  |  |  | + |  | 7 | 210.45 | -406.75 | 27.526 | 0 |
| + | + |  |  |  |  |  |  | + |  |  | 4 | 206.76 | -405.45 | 28.819 | 0 |
| + | + |  |  |  |  |  |  |  | + |  | 4 | 205.03 | -402.00 | 32.273 | 0 |
| + | + | + |  |  |  |  |  |  |  |  | 6 | 206.23 | -400.34 | 33.937 | 0 |
| + | + |  |  |  |  |  |  |  |  |  | 3 | 201.00 | -395.96 | 38.310 | 0 |
| + |  | + | + |  |  |  |  |  |  |  | 7 | 168.51 | -322.87 | 111.407 | 0 |
| + |  | + |  |  |  |  |  | + |  |  | 6 | 164.72 | -317.32 | 116.953 | 0 |
| + |  |  | + |  |  |  |  |  |  |  | 4 | 162.21 | -316.37 | 117.902 | 0 |
| + |  | + | + |  |  | + |  |  |  |  | 13 | 170.90 | -315.26 | 119.012 | 0 |
| + |  | + |  |  |  |  |  |  | + |  | 6 | 163.27 | -314.42 | 119.853 | 0 |
| + |  |  |  |  |  |  |  | + |  |  | 3 | 158.50 | -310.97 | 123.307 | 0 |
| + |  | + |  |  |  |  |  |  |  |  | 5 | 159.10 | -308.10 | 126.168 | 0 |
| + |  |  |  |  |  |  |  |  | + |  | 3 | 156.92 | -307.81 | 126.467 | 0 |
| + |  |  |  |  |  |  |  |  |  |  | 2 | 152.97 | -301.93 | 132.347 | 0 |

| **Northern Pintail – Model structure** | | | | | | | | | |  | **Model statistics** | | | | |
| --- | --- | --- | --- | --- | --- | --- | --- | --- | --- | --- | --- | --- | --- | --- | --- |
| **Intercept** | **Year** | **Month** | **JVB** | **Year × Month** | **Year × JVB** | **Month × JVB** | **Year × Month × JVB** | **Banding latitude** | **Banding longitude** |  | **K** | **Log likelihood** | **AICc** | **ΔAICc** | **Model weight** |
| + | + | + | + |  | + |  |  |  |  |  | 8 | 25.75 | -34.56 | 0 | 0.141 |
| + | + |  |  |  |  |  |  |  |  |  | 3 | 20.26 | -34.38 | 0.180 | 0.129 |
| + | + | + |  |  |  |  |  |  |  |  | 6 | 23.41 | -34.28 | 0.280 | 0.123 |
| + | + | + | + |  |  |  |  |  |  |  | 7 | 24.37 | -34.01 | 0.549 | 0.108 |
| + | + | + |  |  |  |  |  | + |  |  | 7 | 24.15 | -33.58 | 0.975 | 0.087 |
| + | + | + |  |  |  |  |  |  | + |  | 7 | 24.12 | -33.51 | 1.044 | 0.084 |
| + | + |  | + |  |  |  |  |  |  |  | 4 | 20.36 | -32.48 | 2.081 | 0.050 |
| + | + | + | + |  | + | + |  |  |  |  | 9 | 25.81 | -32.44 | 2.114 | 0.049 |
| + | + |  |  |  |  |  |  | + |  |  | 4 | 20.35 | -32.44 | 2.117 | 0.049 |
| + | + |  |  |  |  |  |  |  | + |  | 4 | 20.34 | -32.43 | 2.130 | 0.049 |
| + | + |  | + |  | + |  |  |  |  |  | 5 | 21.31 | -32.23 | 2.323 | 0.044 |
| + | + | + | + |  |  | + |  |  |  |  | 8 | 24.41 | -31.88 | 2.675 | 0.037 |
| + | + | + |  | + |  |  |  |  |  |  | 9 | 24.29 | -29.40 | 5.159 | 0.011 |
| + | + | + | + | + | + |  |  |  |  |  | 11 | 26.46 | -29.17 | 5.387 | 0.010 |
| + | + | + | + | + |  |  |  |  |  |  | 10 | 25.09 | -28.73 | 5.823 | 0.008 |
| + | + | + |  | + |  |  |  |  | + |  | 10 | 24.97 | -28.49 | 6.065 | 0.007 |
| + | + | + |  | + |  |  |  | + |  |  | 10 | 24.95 | -28.45 | 6.103 | 0.007 |
| + | + | + | + | + | + | + |  |  |  |  | 12 | 26.72 | -27.36 | 7.200 | 0.004 |
| + | + | + | + | + |  | + |  |  |  |  | 11 | 25.26 | -26.77 | 7.785 | 0.003 |
| + | + | + | + | + | + | + | + |  |  |  | 13 | 26.73 | -25.01 | 9.549 | 0.001 |
| + |  |  | + |  |  |  |  |  |  |  | 3 | -14.90 | 35.95 | 70.509 | 0 |
| + |  |  |  |  |  |  |  |  |  |  | 2 | -16.60 | 37.28 | 71.838 | 0 |
| + |  |  |  |  |  |  |  | + |  |  | 3 | -16.43 | 39.01 | 73.562 | 0 |
| + |  |  |  |  |  |  |  |  | + |  | 3 | -16.58 | 39.31 | 73.866 | 0 |
| + |  | + | + |  |  |  |  |  |  |  | 6 | -14.34 | 41.21 | 75.767 | 0 |
| + |  | + |  |  |  |  |  |  |  |  | 5 | -15.60 | 41.58 | 76.138 | 0 |
| + |  | + | + |  |  | + |  |  |  |  | 7 | -14.27 | 43.27 | 77.824 | 0 |
| + |  | + |  |  |  |  |  | + |  |  | 6 | -15.57 | 43.67 | 78.230 | 0 |
| + |  | + |  |  |  |  |  |  | + |  | 6 | -15.60 | 43.73 | 78.285 | 0 |

**Supplemental Table 9.** Model selection table of models that explain variation in the Bhattacharyya's affinity indices of overlap (compared to 1960–1979 base line period) of the 50% isopleth of kernel density estimator-based utilization distributions of band recoveries from Blue-winged Teal, Mallards, and Northern Pintail in the Central and Mississippi Flyways of North America between 1960 and 2019. Model structure terms include year (continuous), month (October–January; categorical), Joint Venture of banding origin (JVB), banding latitude, banding longitude, and several possible two-way and three-way interaction terms. Model statistics include the number of parameters (K), the log likelihood, AIC_c_ values, difference in AIC_c_ values compared to the model with the lowest AIC_c_ value (ΔAICc), and the model weight.

| **Blue-winged Teal – Model structure** | | | | | | | | | |  | **Model statistics** | | | | |  |
| --- | --- | --- | --- | --- | --- | --- | --- | --- | --- | --- | --- | --- | --- | --- | --- | --- |
| **Intercept** | **Year** | **Month** | **JVB** | **Year × Month** | **Year × JVB** | **Month × JVB** | **Year × Month × JVB** | **Banding latitude** | **Banding longitude** |  | **K** | **Log likelihood** | **AICc** | **ΔAICc** | **Model weight** | |
| + | + | + | + | + | + | + | + |  |  |  | 9 | 134.29 | -249.07 | 0 | 0.316 | |
| + | + |  | + |  | + |  |  |  |  |  | 5 | 128.93 | -247.37 | 1.699 | 0.135 | |
| + | + |  | + |  |  |  |  |  |  |  | 4 | 127.61 | -246.91 | 2.159 | 0.107 | |
| + | + | + | + | + |  |  |  |  |  |  | 6 | 129.69 | -246.69 | 2.372 | 0.097 | |
| + | + | + | + | + | + |  |  |  |  |  | 7 | 130.67 | -246.41 | 2.655 | 0.084 | |
| + | + | + | + |  | + |  |  |  |  |  | 6 | 128.98 | -245.26 | 3.801 | 0.047 | |
| + | + | + | + |  |  |  |  |  |  |  | 5 | 127.67 | -244.85 | 4.217 | 0.038 | |
| + | + | + | + | + |  | + |  |  |  |  | 7 | 129.74 | -244.55 | 4.512 | 0.033 | |
| + | + | + | + | + | + | + |  |  |  |  | 8 | 130.86 | -244.52 | 4.543 | 0.033 | |
| + | + | + |  | + |  |  |  | + |  |  | 6 | 128.38 | -244.07 | 4.999 | 0.026 | |
| + | + |  |  |  |  |  |  | + |  |  | 4 | 126.19 | -244.05 | 5.015 | 0.026 | |
| + | + | + | + |  | + | + |  |  |  |  | 7 | 129.39 | -243.85 | 5.211 | 0.023 | |
| + | + | + | + |  |  | + |  |  |  |  | 6 | 127.84 | -242.99 | 6.081 | 0.015 | |
| + | + | + |  |  |  |  |  | + |  |  | 5 | 126.24 | -241.99 | 7.079 | 0.009 | |
| + | + | + |  | + |  |  |  |  | + |  | 6 | 126.33 | -239.98 | 9.090 | 0.003 | |
| + | + |  |  |  |  |  |  |  | + |  | 4 | 123.90 | -239.48 | 9.584 | 0.003 | |
| + | + |  |  |  |  |  |  |  |  |  | 3 | 122.46 | -238.72 | 10.342 | 0.002 | |
| + | + | + |  |  |  |  |  |  | + |  | 5 | 124.10 | -237.72 | 11.349 | 0.001 | |
| + | + | + |  | + |  |  |  |  |  |  | 5 | 124.08 | -237.67 | 11.398 | 0.001 | |
| + | + | + |  |  |  |  |  |  |  |  | 4 | 122.46 | -236.59 | 12.471 | 0.001 | |
| + |  |  | + |  |  |  |  |  |  |  | 3 | 104.72 | -203.24 | 45.824 | 0 | |
| + |  | + | + |  |  |  |  |  |  |  | 4 | 105.68 | -203.04 | 46.022 | 0 | |
| + |  | + | + |  |  | + |  |  |  |  | 5 | 105.73 | -200.98 | 48.090 | 0 | |
| + |  |  |  |  |  |  |  | + |  |  | 3 | 101.11 | -196.03 | 53.031 | 0 | |
| + |  | + |  |  |  |  |  | + |  |  | 4 | 102.03 | -195.73 | 53.336 | 0 | |
| + |  |  |  |  |  |  |  |  |  |  | 2 | 98.89 | -193.69 | 55.381 | 0 | |
| + |  | + |  |  |  |  |  |  |  |  | 3 | 99.50 | -192.80 | 56.264 | 0 | |
| + |  |  |  |  |  |  |  |  | + |  | 3 | 99.30 | -192.41 | 56.653 | 0 | |
| + |  | + |  |  |  |  |  |  | + |  | 4 | 99.65 | -190.97 | 58.095 | 0 | |

| **Mallard – Model structure** | | | | | | | | | |  | **Model statistics** | | | | |
| --- | --- | --- | --- | --- | --- | --- | --- | --- | --- | --- | --- | --- | --- | --- | --- |
| **Intercept** | **Year** | **Month** | **JVB** | **Year × Month** | **Year × JVB** | **Month × JVB** | **Year × Month × JVB** | **Banding latitude** | **Banding longitude** |  | **K** | **Log likelihood** | **AICc** | **ΔAICc** | **Model weight** |
| + | + | + | + | + | + | + | + |  |  |  | 25 | 838.41 | -1624.88 | 0 | 1 |
| + | + | + | + | + | + | + |  |  |  |  | 19 | 813.90 | -1588.69 | 36.198 | 0 |
| + | + | + | + |  | + | + |  |  |  |  | 16 | 795.87 | -1558.94 | 65.945 | 0 |
| + | + | + | + | + |  | + |  |  |  |  | 17 | 776.75 | -1518.61 | 106.273 | 0 |
| + | + | + | + |  |  | + |  |  |  |  | 14 | 760.30 | -1491.99 | 132.898 | 0 |
| + |  | + | + |  |  | + |  |  |  |  | 13 | 758.31 | -1490.09 | 134.799 | 0 |
| + | + | + | + | + | + |  |  |  |  |  | 13 | 693.97 | -1361.42 | 263.466 | 0 |
| + | + | + | + |  | + |  |  |  |  |  | 10 | 680.93 | -1341.54 | 283.342 | 0 |
| + | + | + | + | + |  |  |  |  |  |  | 11 | 669.87 | -1317.35 | 307.531 | 0 |
| + | + | + |  | + |  |  |  |  | + |  | 10 | 664.52 | -1308.72 | 316.163 | 0 |
| + | + | + | + |  |  |  |  |  |  |  | 8 | 657.55 | -1298.90 | 325.989 | 0 |
| + |  | + | + |  |  |  |  |  |  |  | 7 | 656.03 | -1297.89 | 326.995 | 0 |
| + | + | + |  |  |  |  |  |  | + |  | 7 | 651.84 | -1289.51 | 335.369 | 0 |
| + |  | + |  |  |  |  |  |  | + |  | 6 | 649.97 | -1287.82 | 337.064 | 0 |
| + | + | + |  | + |  |  |  | + |  |  | 10 | 654.05 | -1287.79 | 337.099 | 0 |
| + | + | + |  |  |  |  |  | + |  |  | 7 | 642.64 | -1271.11 | 353.770 | 0 |
| + |  | + |  |  |  |  |  | + |  |  | 6 | 640.88 | -1269.64 | 355.241 | 0 |
| + | + | + |  | + |  |  |  |  |  |  | 9 | 585.05 | -1151.84 | 473.042 | 0 |
| + | + | + |  |  |  |  |  |  |  |  | 6 | 575.50 | -1138.89 | 485.997 | 0 |
| + |  | + |  |  |  |  |  |  |  |  | 5 | 574.35 | -1138.61 | 486.272 | 0 |
| + | + |  | + |  | + |  |  |  |  |  | 7 | 518.64 | -1023.11 | 601.771 | 0 |
| + | + |  |  |  |  |  |  |  | + |  | 4 | 504.59 | -1001.12 | 623.765 | 0 |
| + |  |  |  |  |  |  |  |  | + |  | 3 | 502.68 | -999.33 | 625.559 | 0 |
| + | + |  | + |  |  |  |  |  |  |  | 5 | 503.88 | -997.67 | 627.211 | 0 |
| + |  |  | + |  |  |  |  |  |  |  | 4 | 502.27 | -996.48 | 628.405 | 0 |
| + | + |  |  |  |  |  |  | + |  |  | 4 | 496.24 | -984.42 | 640.460 | 0 |
| + |  |  |  |  |  |  |  | + |  |  | 3 | 494.41 | -982.78 | 642.105 | 0 |
| + | + |  |  |  |  |  |  |  |  |  | 3 | 448.67 | -891.30 | 733.582 | 0 |
| + |  |  |  |  |  |  |  |  |  |  | 2 | 447.33 | -890.65 | 734.233 | 0 |

| **Northern Pintail – Model structure** | | | | | | | | | |  | **Model statistics** | | | | |
| --- | --- | --- | --- | --- | --- | --- | --- | --- | --- | --- | --- | --- | --- | --- | --- |
| **Intercept** | **Year** | **Month** | **JVB** | **Year × Month** | **Year × JVB** | **Month × JVB** | **Year × Month × JVB** | **Banding latitude** | **Banding longitude** |  | **K** | **Log likelihood** | **AICc** | **ΔAICc** | **Model weight** |
| + | + | + | + | + | + | + |  |  |  |  | 12 | 138.55 | -251.03 | 0 | 0.676 |
| + | + | + | + | + | + | + | + |  |  |  | 13 | 138.66 | -248.89 | 2.140 | 0.232 |
| + | + | + | + | + |  | + |  |  |  |  | 11 | 135.39 | -247.04 | 3.985 | 0.092 |
| + | + | + |  | + |  |  |  |  |  |  | 9 | 126.79 | -234.40 | 16.622 | 0 |
| + | + | + | + | + | + |  |  |  |  |  | 11 | 128.90 | -234.06 | 16.966 | 0 |
| + | + | + | + |  | + | + |  |  |  |  | 9 | 125.94 | -232.71 | 18.318 | 0 |
| + | + | + | + |  |  | + |  |  |  |  | 8 | 124.65 | -232.37 | 18.653 | 0 |
| + | + | + |  | + |  |  |  |  | + |  | 10 | 126.81 | -232.18 | 18.847 | 0 |
| + | + | + | + | + |  |  |  |  |  |  | 10 | 126.80 | -232.16 | 18.866 | 0 |
| + | + | + |  | + |  |  |  | + |  |  | 10 | 126.80 | -232.15 | 18.875 | 0 |
| + | + | + |  |  |  |  |  |  |  |  | 6 | 116.38 | -220.22 | 30.811 | 0 |
| + | + | + | + |  |  |  |  |  |  |  | 7 | 116.55 | -218.37 | 32.653 | 0 |
| + | + | + |  |  |  |  |  | + |  |  | 7 | 116.40 | -218.08 | 32.945 | 0 |
| + | + | + |  |  |  |  |  |  | + |  | 7 | 116.40 | -218.08 | 32.951 | 0 |
| + | + | + | + |  | + |  |  |  |  |  | 8 | 117.50 | -218.07 | 32.959 | 0 |
| + | + |  | + |  |  |  |  |  |  |  | 4 | 102.80 | -197.34 | 53.689 | 0 |
| + | + |  |  |  |  |  |  |  | + |  | 4 | 102.05 | -195.85 | 55.174 | 0 |
| + | + |  | + |  | + |  |  |  |  |  | 5 | 103.07 | -195.75 | 55.274 | 0 |
| + | + |  |  |  |  |  |  |  |  |  | 3 | 100.94 | -195.72 | 55.302 | 0 |
| + | + |  |  |  |  |  |  | + |  |  | 4 | 101.76 | -195.26 | 55.766 | 0 |
| + |  | + | + |  |  | + |  |  |  |  | 7 | 104.69 | -194.65 | 56.373 | 0 |
| + |  | + |  |  |  |  |  |  |  |  | 5 | 97.56 | -184.74 | 66.282 | 0 |
| + |  | + | + |  |  |  |  |  |  |  | 6 | 98.59 | -184.64 | 66.383 | 0 |
| + |  | + |  |  |  |  |  | + |  |  | 6 | 97.80 | -183.06 | 67.964 | 0 |
| + |  | + |  |  |  |  |  |  | + |  | 6 | 97.66 | -182.78 | 68.251 | 0 |
| + |  |  |  |  |  |  |  |  |  |  | 2 | 81.81 | -159.55 | 91.475 | 0 |
| + |  |  |  |  |  |  |  |  | + |  | 3 | 82.24 | -158.32 | 92.704 | 0 |
| + |  |  |  |  |  |  |  | + |  |  | 3 | 81.91 | -157.66 | 93.363 | 0 |
| + |  |  | + |  |  |  |  |  |  |  | 3 | 81.81 | -157.48 | 93.551 | 0 |

**Supplemental Table 10.** Model selection table of models that explain variation in the Bhattacharyya's affinity indices of overlap (compared to 1960–1979 base line period) of the 95% isopleth of kernel density estimator-based utilization distributions of band recoveries from Blue-winged Teal, Mallards, and Northern Pintail in the Central and Mississippi Flyways of North America between 1960 and 2019. Model structure terms include year (continuous), month (October–January; categorical), Joint Venture of banding origin (JVB), banding latitude, banding longitude, and several possible two-way and three-way interaction terms. Model statistics include the number of parameters (K), the log likelihood, AIC_c_ values, difference in AIC_c_ values compared to the model with the lowest AIC_c_ value (ΔAICc), and the model weight.

| **Blue-winged Teal – Model structure** | | | | | | | | | |  | **Model statistics** | | | | |  |
| --- | --- | --- | --- | --- | --- | --- | --- | --- | --- | --- | --- | --- | --- | --- | --- | --- |
| **Intercept** | **Year** | **Month** | **JVB** | **Year × Month** | **Year × JVB** | **Month × JVB** | **Year × Month × JVB** | **Banding latitude** | **Banding longitude** |  | **K** | **Log likelihood** | **AICc** | **ΔAICc** | **Model weight** | |
| + | + | + | + | + | + | + | + |  |  |  | 9 | 134.86 | -250.21 | 0 | 0.665 | |
| + | + | + | + | + | + | + |  |  |  |  | 8 | 132.93 | -248.67 | 1.544 | 0.307 | |
| + | + | + | + | + | + |  |  |  |  |  | 7 | 129.28 | -243.64 | 6.576 | 0.025 | |
| + | + | + | + | + |  | + |  |  |  |  | 7 | 126.49 | -238.06 | 12.153 | 0.002 | |
| + | + | + | + | + |  |  |  |  |  |  | 6 | 124.68 | -236.67 | 13.542 | 0.001 | |
| + | + | + |  | + |  |  |  | + |  |  | 6 | 123.09 | -233.50 | 16.712 | 0 | |
| + | + | + |  | + |  |  |  |  |  |  | 5 | 120.81 | -231.13 | 19.083 | 0 | |
| + | + | + |  | + |  |  |  |  | + |  | 6 | 121.33 | -229.98 | 20.233 | 0 | |
| + | + | + | + |  | + | + |  |  |  |  | 7 | 118.48 | -222.04 | 28.176 | 0 | |
| + | + | + | + |  | + |  |  |  |  |  | 6 | 113.12 | -213.55 | 36.660 | 0 | |
| + | + | + | + |  |  | + |  |  |  |  | 6 | 110.47 | -208.25 | 41.959 | 0 | |
| + | + | + | + |  |  |  |  |  |  |  | 5 | 107.53 | -204.57 | 45.648 | 0 | |
| + | + | + |  |  |  |  |  | + |  |  | 5 | 106.14 | -201.79 | 48.421 | 0 | |
| + | + | + |  |  |  |  |  |  |  |  | 4 | 105.03 | -201.74 | 48.469 | 0 | |
| + | + | + |  |  |  |  |  |  | + |  | 5 | 105.04 | -199.60 | 50.615 | 0 | |
| + |  | + | + |  |  | + |  |  |  |  | 5 | 79.20 | -147.91 | 102.302 | 0 | |
| + |  | + | + |  |  |  |  |  |  |  | 4 | 77.73 | -147.14 | 103.069 | 0 | |
| + |  | + |  |  |  |  |  |  | + |  | 4 | 76.45 | -144.58 | 105.631 | 0 | |
| + |  | + |  |  |  |  |  |  |  |  | 3 | 74.16 | -142.12 | 108.093 | 0 | |
| + |  | + |  |  |  |  |  | + |  |  | 4 | 74.79 | -141.25 | 108.962 | 0 | |
| + | + |  | + |  | + |  |  |  |  |  | 5 | 64.98 | -119.47 | 130.740 | 0 | |
| + | + |  |  |  |  |  |  |  | + |  | 4 | 63.72 | -119.12 | 131.095 | 0 | |
| + | + |  | + |  |  |  |  |  |  |  | 4 | 62.47 | -116.61 | 133.599 | 0 | |
| + | + |  |  |  |  |  |  | + |  |  | 4 | 61.60 | -114.88 | 135.338 | 0 | |
| + | + |  |  |  |  |  |  |  |  |  | 3 | 59.86 | -113.52 | 136.693 | 0 | |
| + |  |  | + |  |  |  |  |  |  |  | 3 | 52.00 | -97.80 | 152.411 | 0 | |
| + |  |  |  |  |  |  |  | + |  |  | 3 | 49.83 | -93.46 | 156.752 | 0 | |
| + |  |  |  |  |  |  |  |  |  |  | 2 | 48.55 | -93.01 | 157.205 | 0 | |
| + |  |  |  |  |  |  |  |  | + |  | 3 | 48.82 | -91.45 | 158.760 | 0 | |

| **Mallard – Model structure** | | | | | | | | | |  | **Model statistics** | | | | |
| --- | --- | --- | --- | --- | --- | --- | --- | --- | --- | --- | --- | --- | --- | --- | --- |
| **Intercept** | **Year** | **Month** | **JVB** | **Year × Month** | **Year × JVB** | **Month × JVB** | **Year × Month × JVB** | **Banding latitude** | **Banding longitude** |  | **K** | **Log likelihood** | **AICc** | **ΔAICc** | **Model weight** |
| + | + | + | + | + | + | + | + |  |  |  | 25 | 1145.78 | -2239.63 | 0 | 0.997 |
| + | + | + | + | + | + | + |  |  |  |  | 19 | 1133.35 | -2227.59 | 12.036 | 0.002 |
| + | + | + | + | + |  | + |  |  |  |  | 17 | 1129.47 | -2224.04 | 15.583 | 0 |
| + | + | + | + |  | + | + |  |  |  |  | 16 | 1106.42 | -2180.04 | 59.585 | 0 |
| + | + | + | + |  |  | + |  |  |  |  | 14 | 1102.68 | -2176.74 | 62.884 | 0 |
| + |  | + | + |  |  | + |  |  |  |  | 13 | 1078.84 | -2131.15 | 108.476 | 0 |
| + | + | + | + | + | + |  |  |  |  |  | 13 | 982.53 | -1938.53 | 301.101 | 0 |
| + | + | + | + | + |  |  |  |  |  |  | 11 | 978.07 | -1933.75 | 305.881 | 0 |
| + | + | + |  | + |  |  |  |  | + |  | 10 | 976.19 | -1932.05 | 307.573 | 0 |
| + | + | + |  | + |  |  |  | + |  |  | 10 | 970.96 | -1921.59 | 318.034 | 0 |
| + | + | + |  | + |  |  |  |  |  |  | 9 | 965.10 | -1911.93 | 327.696 | 0 |
| + | + | + | + |  | + |  |  |  |  |  | 10 | 965.38 | -1910.44 | 329.188 | 0 |
| + | + | + | + |  |  |  |  |  |  |  | 8 | 961.01 | -1905.82 | 333.808 | 0 |
| + | + | + |  |  |  |  |  |  | + |  | 7 | 958.81 | -1903.45 | 336.177 | 0 |
| + | + | + |  |  |  |  |  | + |  |  | 7 | 954.24 | -1894.31 | 345.317 | 0 |
| + | + | + |  |  |  |  |  |  |  |  | 6 | 948.80 | -1885.48 | 354.151 | 0 |
| + |  | + | + |  |  |  |  |  |  |  | 7 | 945.20 | -1876.24 | 363.384 | 0 |
| + |  | + |  |  |  |  |  |  | + |  | 6 | 942.72 | -1873.32 | 366.307 | 0 |
| + |  | + |  |  |  |  |  | + |  |  | 6 | 938.50 | -1864.88 | 374.748 | 0 |
| + |  | + |  |  |  |  |  |  |  |  | 5 | 933.59 | -1857.09 | 382.536 | 0 |
| + | + |  | + |  | + |  |  |  |  |  | 7 | 933.02 | -1851.88 | 387.746 | 0 |
| + | + |  | + |  |  |  |  |  |  |  | 5 | 929.12 | -1848.15 | 391.477 | 0 |
| + | + |  |  |  |  |  |  |  | + |  | 4 | 927.84 | -1847.61 | 392.012 | 0 |
| + | + |  |  |  |  |  |  | + |  |  | 4 | 923.00 | -1837.93 | 401.693 | 0 |
| + | + |  |  |  |  |  |  |  |  |  | 3 | 917.85 | -1829.66 | 409.967 | 0 |
| + |  |  | + |  |  |  |  |  |  |  | 4 | 911.99 | -1815.92 | 423.704 | 0 |
| + |  |  |  |  |  |  |  |  | + |  | 3 | 910.41 | -1814.79 | 424.841 | 0 |
| + |  |  |  |  |  |  |  | + |  |  | 3 | 905.93 | -1805.82 | 433.809 | 0 |
| + |  |  |  |  |  |  |  |  |  |  | 2 | 901.31 | -1798.61 | 441.019 | 0 |

| **Northern Pintail – Model structure** | | | | | | | | | |  | **Model statistics** | | | | |
| --- | --- | --- | --- | --- | --- | --- | --- | --- | --- | --- | --- | --- | --- | --- | --- |
| **Intercept** | **Year** | **Month** | **JVB** | **Year × Month** | **Year × JVB** | **Month × JVB** | **Year × Month × JVB** | **Banding latitude** | **Banding longitude** |  | **K** | **Log likelihood** | **AICc** | **ΔAICc** | **Model weight** |
| + | + | + | + | + | + |  |  |  |  |  | 11 | 191.62 | -359.50 | 0 | 0.443 |
| + | + | + |  | + |  |  |  |  | + |  | 10 | 189.80 | -358.15 | 1.349 | 0.225 |
| + | + | + | + | + | + | + |  |  |  |  | 12 | 191.65 | -357.21 | 2.280 | 0.142 |
| + | + | + | + | + | + | + | + |  |  |  | 13 | 192.33 | -356.22 | 3.273 | 0.086 |
| + | + | + | + | + |  |  |  |  |  |  | 10 | 188.51 | -355.57 | 3.926 | 0.062 |
| + | + | + | + | + |  | + |  |  |  |  | 11 | 188.60 | -353.44 | 6.054 | 0.021 |
| + | + | + |  | + |  |  |  | + |  |  | 10 | 187.25 | -353.05 | 6.444 | 0.018 |
| + | + | + |  |  |  |  |  |  | + |  | 7 | 181.11 | -347.50 | 11.996 | 0.001 |
| + | + | + | + |  | + |  |  |  |  |  | 8 | 181.83 | -346.73 | 12.767 | 0.001 |
| + | + | + | + |  |  |  |  |  |  |  | 7 | 180.25 | -345.78 | 13.712 | 0 |
| + | + | + | + |  | + | + |  |  |  |  | 9 | 181.89 | -344.61 | 14.885 | 0 |
| + | + | + |  |  |  |  |  | + |  |  | 7 | 179.41 | -344.10 | 15.392 | 0 |
| + | + | + | + |  |  | + |  |  |  |  | 8 | 180.34 | -343.74 | 15.754 | 0 |
| + | + |  |  |  |  |  |  |  | + |  | 4 | 173.89 | -339.53 | 19.968 | 0 |
| + | + |  | + |  | + |  |  |  |  |  | 5 | 174.41 | -338.43 | 21.064 | 0 |
| + | + |  | + |  |  |  |  |  |  |  | 4 | 172.80 | -337.34 | 22.155 | 0 |
| + | + | + |  | + |  |  |  |  |  |  | 9 | 178.17 | -337.17 | 22.328 | 0 |
| + | + |  |  |  |  |  |  | + |  |  | 4 | 171.57 | -334.88 | 24.611 | 0 |
| + | + | + |  |  |  |  |  |  |  |  | 6 | 171.95 | -331.36 | 28.138 | 0 |
| + | + |  |  |  |  |  |  |  |  |  | 3 | 165.25 | -324.34 | 35.156 | 0 |
| + |  | + | + |  |  |  |  |  |  |  | 6 | 139.65 | -266.77 | 92.729 | 0 |
| + |  | + | + |  |  | + |  |  |  |  | 7 | 139.75 | -264.78 | 94.712 | 0 |
| + |  |  | + |  |  |  |  |  |  |  | 3 | 133.71 | -261.27 | 98.226 | 0 |
| + |  | + |  |  |  |  |  | + |  |  | 6 | 130.80 | -249.06 | 110.437 | 0 |
| + |  | + |  |  |  |  |  |  | + |  | 6 | 130.36 | -248.18 | 111.313 | 0 |
| + |  |  |  |  |  |  |  |  | + |  | 3 | 126.28 | -246.42 | 113.080 | 0 |
| + |  |  |  |  |  |  |  | + |  |  | 3 | 126.22 | -246.29 | 113.210 | 0 |
| + |  |  |  |  |  |  |  |  |  |  | 2 | 119.43 | -234.78 | 124.712 | 0 |
| + |  | + |  |  |  |  |  |  |  |  | 5 | 122.08 | -233.78 | 125.715 | 0 |

**Supplemental Table 11.** 60-year change in centroid longitude of the 50% and 95% isopleths of kernel density estimator-based utilization distributions of band recoveries from Blue-winged Teal, Mallards, and Northern Pintail in the Central and Mississippi Flyways of North America between 1960 and 2019. Shown are the estimated effects of year on centroid longitude, and accompanying variance, standard error (SE), and 95% confidence interval for each month of recovery (October–January) and banding region with 30 or more band recoveries. Banding regions include the Prairie Habitat and Western Boreal Area Joint Ventures (PHWB), the Prairie Pothole and Northern Great Plains Joint Ventures (PPNP), and the Upper Mississippi River/Great Lakes Joint Venture and Ontario (UMON).

| **Month** | **Species** | **Banding Region** | **Isopleth** | **60-Year**  **Effect** | **Variance** | **SE** | **95%**  **Confidence Interval** | |
| --- | --- | --- | --- | --- | --- | --- | --- | --- |
| October | Blue-winged Teal | PHWB | 50% | -1.6495 | 0.0931 | 0.3051 | -2.2474 | -1.0516 |
|  |  |  | 95% | -1.6273 | 0.2155 | 0.4643 | -2.5372 | -0.7173 |
|  |  | PPNP | 50% | -1.6495 | 0.0931 | 0.3051 | -2.2474 | -1.0516 |
|  |  |  | 95% | -3.3023 | 0.2367 | 0.4865 | -4.2558 | -2.3487 |
|  | Mallard | PHWB | 50% | 0.1202 | 0.2223 | 0.4715 | -0.8039 | 1.0443 |
|  |  |  | 95% | 1.6144 | 0.1242 | 0.3524 | 0.9236 | 2.3052 |
|  |  | PPNP | 50% | -2.1658 | 0.2223 | 0.4715 | -3.0898 | -1.2417 |
|  |  |  | 95% | 1.0211 | 0.1242 | 0.3524 | 0.3303 | 1.7119 |
|  |  | UMON | 50% | 1.5024 | 0.2223 | 0.4715 | 0.5783 | 2.4265 |
|  |  |  | 95% | 1.2405 | 0.1242 | 0.3524 | 0.5497 | 1.9313 |
|  | Northern Pintail | PPNP | 50% | 4.7989 | 2.6704 | 1.6341 | 1.5960 | 8.0018 |
|  |  |  | 95% | 1.3092 | 0.1740 | 0.4172 | 0.4915 | 2.1269 |
|  |  |  |  |  |  |  |  |  |
| November | Blue-winged Teal | PHWB | 50% | -1.6495 | 0.0931 | 0.3051 | -2.2474 | -1.0516 |
|  |  |  | 95% | -0.5120 | 0.2316 | 0.4813 | -1.4553 | 0.4313 |
|  |  | PPNP | 50% | -1.6495 | 0.0931 | 0.3051 | -2.2474 | -1.0516 |
|  |  |  | 95% | -0.0768 | 0.4881 | 0.6986 | -1.4461 | 1.2926 |
|  | Mallard | PHWB | 50% | 1.1334 | 0.2223 | 0.4715 | 0.2093 | 2.0575 |
|  |  |  | 95% | 1.0130 | 0.1242 | 0.3524 | 0.3222 | 1.7038 |
|  |  | PPNP | 50% | 1.7793 | 0.2223 | 0.4715 | 0.8552 | 2.7034 |
|  |  |  | 95% | 2.0192 | 0.1242 | 0.3524 | 1.3284 | 2.7100 |
|  |  | UMON | 50% | 1.2101 | 0.2223 | 0.4715 | 0.2860 | 2.1341 |
|  |  |  | 95% | 1.7785 | 0.1242 | 0.3524 | 1.0877 | 2.4693 |
|  | Northern Pintail | PHWB | 50% | 0.4023 | 0.3121 | 0.5587 | -0.6927 | 1.4972 |
|  |  |  | 95% | 0.3545 | 0.0523 | 0.2287 | -0.0937 | 0.8027 |
|  |  | PPNP | 50% | 0.4023 | 0.3121 | 0.5587 | -0.6927 | 1.4972 |
|  |  |  | 95% | 1.3092 | 0.1740 | 0.4172 | 0.4915 | 2.1269 |
|  |  |  |  |  |  |  |  |  |
| December | Mallard | PHWB | 50% | -0.1067 | 0.2223 | 0.4715 | -1.0308 | 0.8174 |
|  |  |  | 95% | 0.3013 | 0.1242 | 0.3524 | -0.3895 | 0.9921 |
|  |  | PPNP | 50% | 0.5827 | 0.2223 | 0.4715 | -0.3414 | 1.5067 |
|  |  |  | 95% | 1.3213 | 0.1242 | 0.3524 | 0.6305 | 2.0121 |
|  |  | UMON | 50% | 3.1834 | 0.2223 | 0.4715 | 2.2593 | 4.1075 |
|  |  |  | 95% | 1.6270 | 0.1242 | 0.3524 | 0.9362 | 2.3178 |
|  | Northern Pintail | PHWB | 50% | 0.6955 | 0.2063 | 0.4542 | -0.1947 | 1.5857 |
|  |  |  | 95% | 0.3545 | 0.0523 | 0.2287 | -0.0937 | 0.8027 |
|  |  | PPNP | 50% | 0.6955 | 0.2063 | 0.4542 | -0.1947 | 1.5857 |
|  |  |  | 95% | 1.3092 | 0.1740 | 0.4172 | 0.4915 | 2.1269 |
|  |  |  |  |  |  |  |  |  |
| January | Mallard | PHWB | 50% | 0.7398 | 0.3211 | 0.5667 | -0.3709 | 1.8506 |
|  |  |  | 95% | -0.8485 | 0.1795 | 0.4236 | -1.6788 | -0.0181 |
|  |  | PPNP | 50% | 0.8135 | 0.3159 | 0.5620 | -0.2881 | 1.9151 |
|  |  |  | 95% | -0.4049 | 0.1765 | 0.4202 | -1.2284 | 0.4186 |
|  |  | UMON | 50% | 2.9432 | 0.3049 | 0.5522 | 1.8609 | 4.0255 |
|  |  |  | 95% | 2.1261 | 0.1704 | 0.4128 | 1.3170 | 2.9352 |
|  | Northern Pintail | PHWB | 50% | 3.3788 | 0.5514 | 0.7426 | 1.9233 | 4.8343 |
|  |  |  | 95% | 0.3545 | 0.0523 | 0.2287 | -0.0937 | 0.8027 |
|  |  | PPNP | 50% | 3.3788 | 0.5514 | 0.7426 | 1.9233 | 4.8343 |
|  |  |  | 95% | 1.3092 | 0.1740 | 0.4172 | 0.4915 | 2.1269 |

**Supplemental Table 12.** 60-year change in centroid latitude of the 50% and 95% isopleths of kernel density estimator-based utilization distributions of band recoveries from Blue-winged Teal, Mallards, and Northern Pintail in the Central and Mississippi Flyways of North America between 1960 and 2019. Shown are the estimated effects of year on centroid latitude, and accompanying variance, standard error (SE), and 95% confidence interval for each month of recovery (October–January) and banding region with 30 or more band recoveries. See caption of Supplemental Table 10 for full names of banding regions.

| **Month** | **Species** | **Banding Region** | **Isopleth** | **60-Year**  **Effect** | **Variance** | **SE** | **95%**  **Confidence Limits** | |
| --- | --- | --- | --- | --- | --- | --- | --- | --- |
| October | Blue-winged Teal | PHWB | 50% | -7.8513 | 1.2817 | 1.1321 | -10.0702 | -5.6323 |
|  |  |  | 95% | -4.3683 | 0.4606 | 0.6787 | -5.6985 | -3.0382 |
|  |  | PPNP | 50% | -7.8513 | 1.2817 | 1.1321 | -10.0702 | -5.6323 |
|  |  |  | 95% | -4.3683 | 0.4606 | 0.6787 | -5.6985 | -3.0382 |
|  | Mallard | PHWB | 50% | -0.1000 | 0.2965 | 0.5445 | -1.1673 | 0.9673 |
|  |  |  | 95% | -0.4065 | 0.1398 | 0.3740 | -1.1394 | 0.3265 |
|  |  | PPNP | 50% | 0.9434 | 0.2965 | 0.5445 | -0.1239 | 2.0107 |
|  |  |  | 95% | -0.0949 | 0.1398 | 0.3740 | -0.8278 | 0.6381 |
|  |  | UMON | 50% | -0.2473 | 0.2965 | 0.5445 | -1.3146 | 0.8200 |
|  |  |  | 95% | -0.4937 | 0.1398 | 0.3740 | -1.2266 | 0.2393 |
|  | Northern Pintail | PPNP | 50% | -2.8718 | 5.7339 | 2.3946 | -7.5652 | 1.8215 |
|  |  |  | 95% | -1.8456 | 2.5530 | 1.5978 | -4.9773 | 1.2861 |
|  |  |  |  |  |  |  |  |  |
| November | Blue-winged Teal | PHWB | 50% | 0.5142 | 1.7514 | 1.3234 | -2.0797 | 3.1080 |
|  |  |  | 95% | -1.6071 | 0.6294 | 0.7933 | -3.1620 | -0.0522 |
|  |  | PPNP | 50% | 0.5142 | 1.7514 | 1.3234 | -2.0797 | 3.1080 |
|  |  |  | 95% | -1.6071 | 0.6294 | 0.7933 | -3.1620 | -0.0522 |
|  | Mallard | PHWB | 50% | -0.8872 | 0.2965 | 0.5445 | -1.9545 | 0.1801 |
|  |  |  | 95% | 0.0639 | 0.1398 | 0.3740 | -0.6691 | 0.7968 |
|  |  | PPNP | 50% | -1.9339 | 0.2965 | 0.5445 | -3.0012 | -0.8666 |
|  |  |  | 95% | -0.6063 | 0.1398 | 0.3740 | -1.3393 | 0.1266 |
|  |  | UMON | 50% | -2.0213 | 0.2965 | 0.5445 | -3.0886 | -0.9540 |
|  |  |  | 95% | -0.9939 | 0.1398 | 0.3740 | -1.7268 | -0.2609 |
|  | Northern Pintail | PHWB | 50% | 1.4909 | 0.6859 | 0.8282 | -0.1323 | 3.1141 |
|  |  |  | 95% | 1.8345 | 0.2984 | 0.5462 | 0.7638 | 2.9051 |
|  |  | PPNP | 50% | 3.6260 | 1.4913 | 1.2212 | 1.2326 | 6.0195 |
|  |  |  | 95% | 1.8345 | 0.2984 | 0.5462 | 0.7638 | 2.9051 |
|  |  |  |  |  |  |  |  |  |
| December | Mallard | PHWB | 50% | 1.2072 | 0.2965 | 0.5445 | 0.1399 | 2.2745 |
|  |  |  | 95% | 1.7715 | 0.1398 | 0.3740 | 1.0385 | 2.5044 |
|  |  | PPNP | 50% | 0.4165 | 0.2965 | 0.5445 | -0.6508 | 1.4838 |
|  |  |  | 95% | 1.9846 | 0.1398 | 0.3740 | 1.2516 | 2.7175 |
|  |  | UMON | 50% | 5.4174 | 0.2965 | 0.5445 | 4.3501 | 6.4847 |
|  |  |  | 95% | 3.8365 | 0.1398 | 0.3740 | 3.1035 | 4.5694 |
|  | Northern Pintail | PHWB | 50% | 1.7993 | 0.5200 | 0.7211 | 0.3859 | 3.2127 |
|  |  |  | 95% | 2.3989 | 0.1972 | 0.4441 | 1.5285 | 3.2693 |
|  |  | PPNP | 50% | 3.9344 | 1.0719 | 1.0353 | 1.9051 | 5.9637 |
|  |  |  | 95% | 2.3989 | 0.1972 | 0.4441 | 1.5285 | 3.2693 |
|  |  |  |  |  |  |  |  |  |
| January | Mallard | PHWB | 50% | 1.7151 | 0.4284 | 0.6545 | 0.4322 | 2.9980 |
|  |  |  | 95% | 3.9762 | 0.2020 | 0.4495 | 3.0952 | 4.8572 |
|  |  | PPNP | 50% | 1.2838 | 0.4214 | 0.6492 | 0.0114 | 2.5561 |
|  |  |  | 95% | 3.1360 | 0.1987 | 0.4458 | 2.2623 | 4.0098 |
|  |  | UMON | 50% | 4.6213 | 0.4068 | 0.6378 | 3.3713 | 5.8714 |
|  |  |  | 95% | 4.0563 | 0.1918 | 0.4380 | 3.1978 | 4.9148 |
|  | Northern Pintail | PHWB | 50% | 5.6599 | 1.1853 | 1.0887 | 3.5260 | 7.7938 |
|  |  |  | 95% | 3.1684 | 0.5272 | 0.7261 | 1.7453 | 4.5915 |
|  |  | PPNP | 50% | 7.7950 | 1.9972 | 1.4132 | 5.0251 | 10.5650 |
|  |  |  | 95% | 3.1684 | 0.5272 | 0.7261 | 1.7453 | 4.5915 |

**Supplemental Table 13.** 60-year change in the natural logarithm of relative area (compared to 1960–1979 base line period) of the 50% and 95% isopleths of kernel density estimator-based utilization distributions of band recoveries from Blue-winged Teal, Mallards, and Northern Pintail in the Central and Mississippi Flyways of North America between 1960 and 2019. Shown are the estimated effects of year on the natural logarithm of relative area, and accompanying variance, standard error (SE), and 95% confidence interval for each month of recovery (October–January) and banding region with 30 or more band recoveries. See caption of Supplemental Table 10 for full names of banding regions.

| **Month** | **Species** | **Joint Venture** | **Isopleth** | **60-Year Effect** | **Variance** | **SE** | **95%**  **Confidence Limits** | |
| --- | --- | --- | --- | --- | --- | --- | --- | --- |
| October | Blue-winged Teal | PHWB | 50% | 1.1419 | 0.0647 | 0.2543 | 0.6435 | 1.6404 |
|  |  |  | 95% | 0.5745 | 0.0196 | 0.1399 | 0.3002 | 0.8487 |
|  |  | PPNP | 50% | 1.8452 | 0.0712 | 0.2669 | 1.3220 | 2.3684 |
|  |  |  | 95% | 0.5745 | 0.0196 | 0.1399 | 0.3002 | 0.8487 |
|  | Mallard | PHWB | 50% | -0.0272 | 0.0123 | 0.1110 | -0.2447 | 0.1904 |
|  |  |  | 95% | 0.2262 | 0.0031 | 0.0553 | 0.1179 | 0.3346 |
|  |  | PPNP | 50% | -0.2779 | 0.0123 | 0.1110 | -0.4954 | -0.0604 |
|  |  |  | 95% | 0.0532 | 0.0031 | 0.0553 | -0.0552 | 0.1615 |
|  |  | UMON | 50% | -0.0728 | 0.0123 | 0.1109 | -0.2902 | 0.1446 |
|  |  |  | 95% | 0.1539 | 0.0031 | 0.0552 | 0.0456 | 0.2622 |
|  |  |  |  |  |  |  |  |  |
| November | Blue-winged Teal | PHWB | 50% | 0.2953 | 0.0705 | 0.2656 | -0.2252 | 0.8157 |
|  |  |  | 95% | -0.2930 | 0.0268 | 0.1636 | -0.6136 | 0.0275 |
|  |  | PPNP | 50% | 0.9985 | 0.1024 | 0.3200 | 0.3713 | 1.6258 |
|  |  |  | 95% | -0.2930 | 0.0268 | 0.1636 | -0.6136 | 0.0275 |
|  | Mallard | PHWB | 50% | 0.4420 | 0.0123 | 0.1110 | 0.2245 | 0.6595 |
|  |  |  | 95% | 0.2555 | 0.0031 | 0.0553 | 0.1472 | 0.3639 |
|  |  | PPNP | 50% | 0.1913 | 0.0123 | 0.1110 | -0.0262 | 0.4088 |
|  |  |  | 95% | 0.0825 | 0.0031 | 0.0553 | -0.0258 | 0.1908 |
|  |  | UMON | 50% | 0.3964 | 0.0123 | 0.1109 | 0.1790 | 0.6138 |
|  |  |  | 95% | 0.1832 | 0.0031 | 0.0552 | 0.0749 | 0.2915 |
|  | Northern Pintail | PHWB | 50% | 0.9816 | 0.0107 | 0.1032 | 0.7793 | 1.1840 |
|  |  |  | 95% | 0.5519 | 0.0033 | 0.0575 | 0.4392 | 0.6646 |
|  |  | PPNP | 50% | 0.9816 | 0.0107 | 0.1032 | 0.7793 | 1.1840 |
|  |  |  | 95% | 0.5519 | 0.0033 | 0.0575 | 0.4392 | 0.6646 |
|  |  |  |  |  |  |  |  |  |
| December | Mallard | PHWB | 50% | 0.7847 | 0.0123 | 0.1110 | 0.5672 | 1.0023 |
|  |  |  | 95% | 0.3601 | 0.0031 | 0.0553 | 0.2517 | 0.4684 |
|  |  | PPNP | 50% | 0.5340 | 0.0123 | 0.1110 | 0.3166 | 0.7515 |
|  |  |  | 95% | 0.1870 | 0.0031 | 0.0553 | 0.0787 | 0.2953 |
|  |  | UMON | 50% | 0.7391 | 0.0123 | 0.1109 | 0.5217 | 0.9565 |
|  |  |  | 95% | 0.2877 | 0.0031 | 0.0552 | 0.1794 | 0.3960 |
|  | Northern Pintail | PHWB | 50% | 0.9816 | 0.0107 | 0.1032 | 0.7793 | 1.1840 |
|  |  |  | 95% | 0.5519 | 0.0033 | 0.0575 | 0.4392 | 0.6646 |
|  |  | PPNP | 50% | 0.9816 | 0.0107 | 0.1032 | 0.7793 | 1.1840 |
|  |  |  | 95% | 0.5519 | 0.0033 | 0.0575 | 0.4392 | 0.6646 |
|  |  |  |  |  |  |  |  |  |
| January | Mallard | PHWB | 50% | 0.3574 | 0.0157 | 0.1253 | 0.1118 | 0.6031 |
|  |  |  | 95% | 0.4892 | 0.0039 | 0.0624 | 0.3669 | 0.6116 |
|  |  | PPNP | 50% | 0.1067 | 0.0156 | 0.1250 | -0.1384 | 0.3518 |
|  |  |  | 95% | 0.3162 | 0.0039 | 0.0623 | 0.1941 | 0.4383 |
|  |  | UMON | 50% | 0.3118 | 0.0155 | 0.1245 | 0.0679 | 0.5557 |
|  |  |  | 95% | 0.4169 | 0.0038 | 0.0620 | 0.2954 | 0.5384 |
|  | Northern Pintail | PHWB | 50% | 0.9816 | 0.0107 | 0.1032 | 0.7793 | 1.1840 |
|  |  |  | 95% | 0.5519 | 0.0033 | 0.0575 | 0.4392 | 0.6646 |

**Supplemental Table 14.** 60-year change in Bhattacharyya's affinity indices of overlap (compared to 1960–1979 base line period) of the 50% and 95% isopleths of kernel density estimator-based utilization distributions of band recoveries from Blue-winged Teal, Mallards, and Northern Pintail in the Central and Mississippi Flyways of North America between 1960 and 2019. Shown are the estimated effects of year on the Bhattacharyya's affinity index, and accompanying variance, standard error (SE), and 95% confidence interval for each month of recovery (October–January) and banding region with 30 or more band recoveries. See caption of Supplemental Table 10 for full names of banding regions.

| **Month** | **Species** | **Banding Region** | **Isopleth** | **60-Year Effect** | **Variance** | **SE** | **95%**  **Confidence Limits** | |
| --- | --- | --- | --- | --- | --- | --- | --- | --- |
| October | Blue-winged Teal | PHWB | 50% | -0.1524 | 0.0024 | 0.0493 | -0.2491 | -0.0558 |
|  |  |  | 95% | -0.2911 | 0.0024 | 0.0491 | -0.3873 | -0.1949 |
|  |  | PPNP | 50% | -0.3502 | 0.0027 | 0.0517 | -0.4515 | -0.2489 |
|  |  |  | 95% | -0.5774 | 0.0026 | 0.0514 | -0.6783 | -0.4766 |
|  | Mallard | PHWB | 50% | -0.0296 | 0.0011 | 0.0327 | -0.0937 | 0.0345 |
|  |  |  | 95% | -0.0539 | 0.0004 | 0.0211 | -0.0952 | -0.0126 |
|  |  | PPNP | 50% | -0.1595 | 0.0011 | 0.0327 | -0.2235 | -0.0954 |
|  |  |  | 95% | -0.0483 | 0.0004 | 0.0211 | -0.0896 | -0.0070 |
|  |  | UMON | 50% | -0.0773 | 0.0011 | 0.0327 | -0.1413 | -0.0132 |
|  |  |  | 95% | -0.0104 | 0.0004 | 0.0211 | -0.0517 | 0.0309 |
|  |  |  |  |  |  |  |  |  |
| November | Blue-winged Teal | PHWB | 50% | -0.1694 | 0.0026 | 0.0511 | -0.2696 | -0.0692 |
|  |  |  | 95% | -0.0636 | 0.0026 | 0.0509 | -0.1634 | 0.0362 |
|  |  | PPNP | 50% | -0.0716 | 0.0055 | 0.0742 | -0.2171 | 0.0738 |
|  |  |  | 95% | -0.1305 | 0.0055 | 0.0739 | -0.2754 | 0.0143 |
|  | Mallard | PHWB | 50% | 0.1672 | 0.0011 | 0.0327 | 0.1032 | 0.2313 |
|  |  |  | 95% | 0.0209 | 0.0004 | 0.0211 | -0.0204 | 0.0622 |
|  |  | PPNP | 50% | 0.1498 | 0.0011 | 0.0327 | 0.0858 | 0.2139 |
|  |  |  | 95% | 0.0043 | 0.0004 | 0.0211 | -0.0370 | 0.0456 |
|  |  | UMON | 50% | -0.1782 | 0.0011 | 0.0327 | -0.2423 | -0.1142 |
|  |  |  | 95% | -0.0292 | 0.0004 | 0.0211 | -0.0705 | 0.0121 |
|  | Northern Pintail | PHWB | 50% | -0.1632 | 0.0030 | 0.0550 | -0.2710 | -0.0555 |
|  |  |  | 95% | -0.1907 | 0.0015 | 0.0381 | -0.2655 | -0.1160 |
|  |  | PPNP | 50% | -0.3608 | 0.0074 | 0.0858 | -0.5290 | -0.1926 |
|  |  |  | 95% | -0.3319 | 0.0036 | 0.0599 | -0.4493 | -0.2145 |
|  |  |  |  |  |  |  |  |  |
| December | Mallard | PHWB | 50% | 0.1083 | 0.0011 | 0.0327 | 0.0442 | 0.1724 |
|  |  |  | 95% | 0.0122 | 0.0004 | 0.0211 | -0.0291 | 0.0535 |
|  |  | PPNP | 50% | 0.1095 | 0.0011 | 0.0327 | 0.0454 | 0.1735 |
|  |  |  | 95% | 0.0036 | 0.0004 | 0.0211 | -0.0377 | 0.0449 |
|  |  | UMON | 50% | -0.1508 | 0.0011 | 0.0327 | -0.2149 | -0.0867 |
|  |  |  | 95% | -0.1195 | 0.0004 | 0.0211 | -0.1608 | -0.0782 |
|  | Northern Pintail | PHWB | 50% | -0.1101 | 0.0024 | 0.0485 | -0.2053 | -0.0150 |
|  |  |  | 95% | -0.1714 | 0.0012 | 0.0340 | -0.2381 | -0.1048 |
|  |  | PPNP | 50% | -0.3077 | 0.0053 | 0.0730 | -0.4508 | -0.1646 |
|  |  |  | 95% | -0.3126 | 0.0027 | 0.0524 | -0.4154 | -0.2098 |
|  |  |  |  |  |  |  |  |  |
| January | Mallard | PHWB | 50% | -0.0157 | 0.0015 | 0.0393 | -0.0927 | 0.0613 |
|  |  |  | 95% | -0.1606 | 0.0006 | 0.0253 | -0.2103 | -0.1110 |
|  |  | PPNP | 50% | -0.0572 | 0.0015 | 0.0390 | -0.1336 | 0.0192 |
|  |  |  | 95% | -0.1086 | 0.0006 | 0.0251 | -0.1579 | -0.0594 |
|  |  | UMON | 50% | -0.1753 | 0.0015 | 0.0383 | -0.2504 | -0.1003 |
|  |  |  | 95% | -0.1482 | 0.0006 | 0.0247 | -0.1966 | -0.0998 |
|  | Northern Pintail | PHWB | 50% | -0.3320 | 0.0060 | 0.0775 | -0.4839 | -0.1802 |
|  |  |  | 95% | -0.2961 | 0.0031 | 0.0558 | -0.4054 | -0.1869 |

**Supplemental Figure 1.** Correlation matrices of select metrics describing distributions and distributional changes of kernel density estimator-based utilization distributions of band recoveries from Blue-winged Teal, Mallards, and Northern Pintail in the Central and Mississippi Flyways of North America between 1960 and 2019. Shown are the following distribution metrics: centroid longitude (ctr50x), centroid latitude (ctr50y), eastern extent (eex50), western extent (wex50), northern extent (nex50), southern extent (sex50), relative area compared to 1960–1979 baseline (area50), Bhattacharyya's affinity overlap with 1960–1979 baseline (BA50), absolute overlap in area with 1960–1979 baseline (HR50), and absolute overlap in volume with 1960–1979 baseline (PHR50). The “50” indicates that this metric was calculated for the 50% isopleth. In other plots they represent metrics calculated for the 70%, 90%, or 95% isopleth of the utilization distribution. Data has been pooled across sexes, ages, and Joint Ventures of banding origin.

Blue-winged Teal – Correlations among metrics for each potential isopleth level


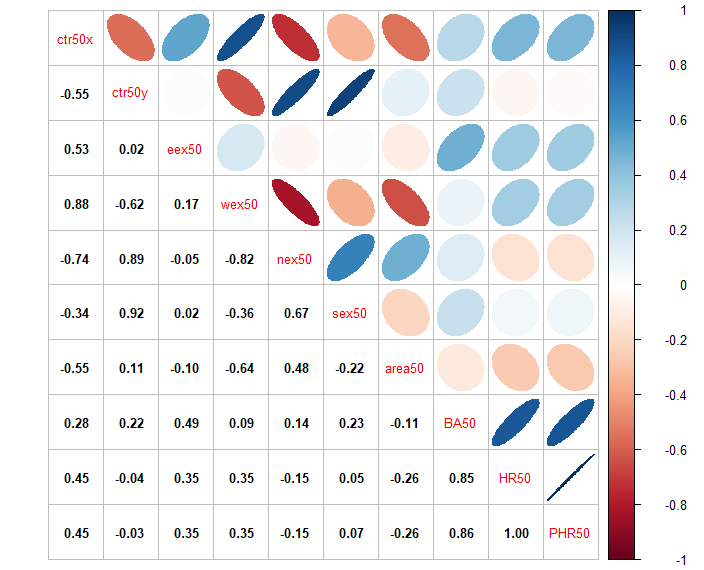


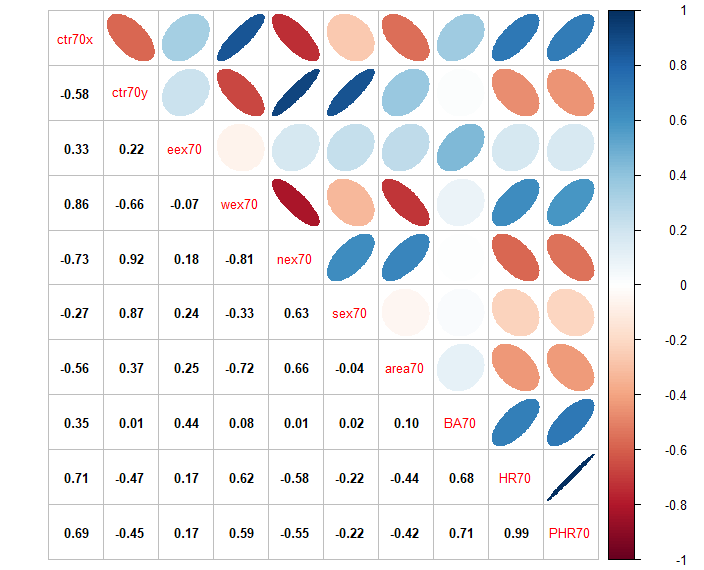


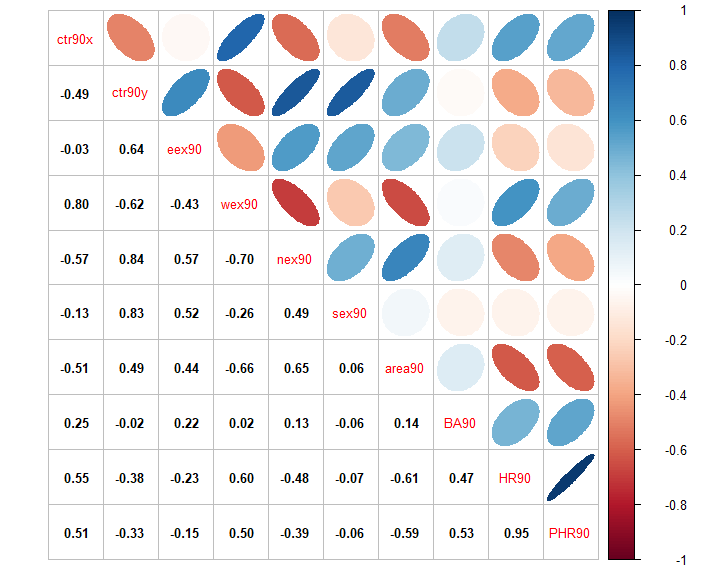


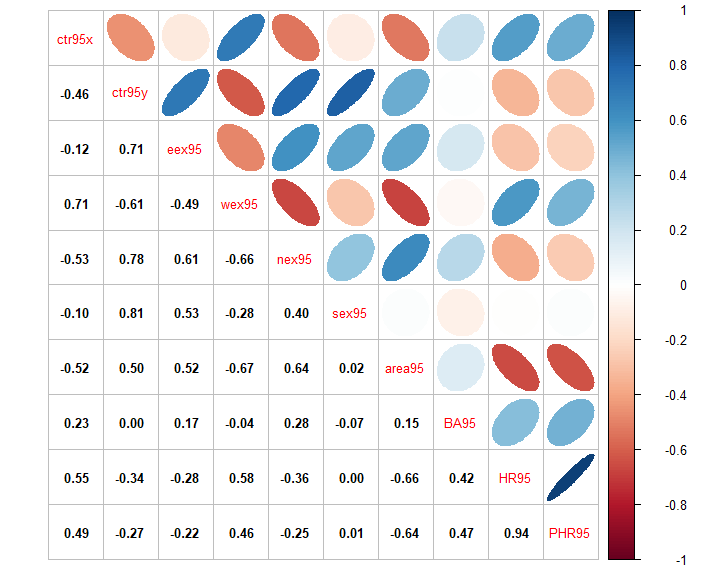


Blue-winged Teal – Correlations among isopleth levels for each metric


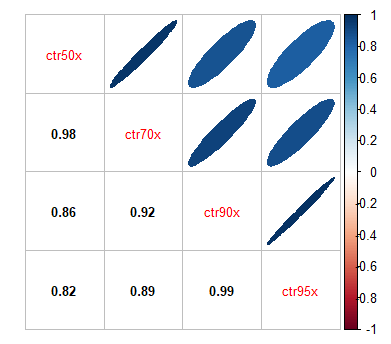

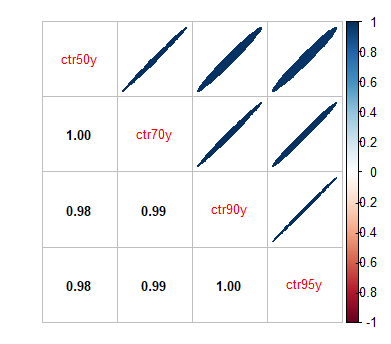


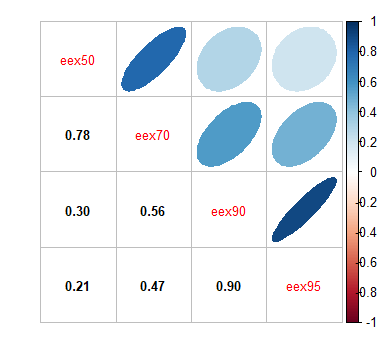

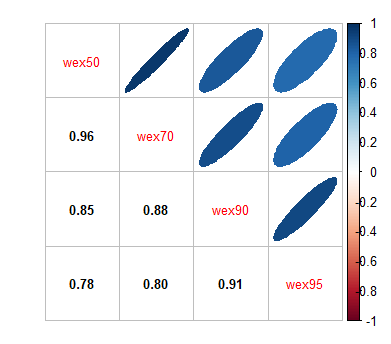


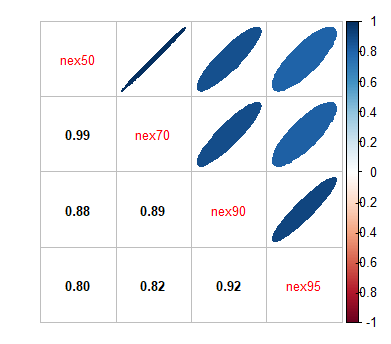

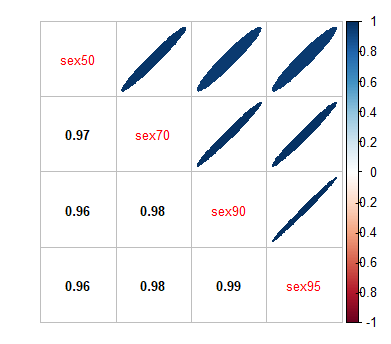


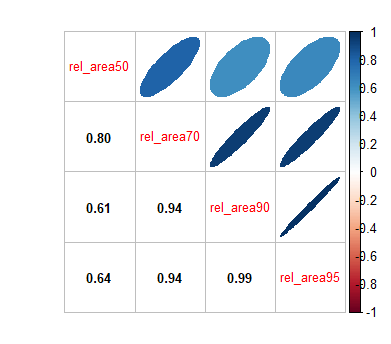

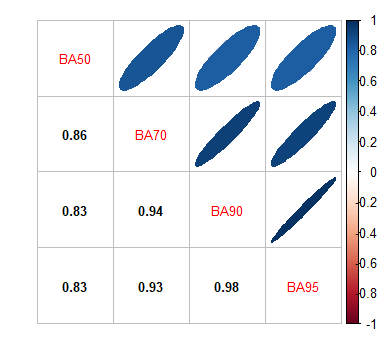


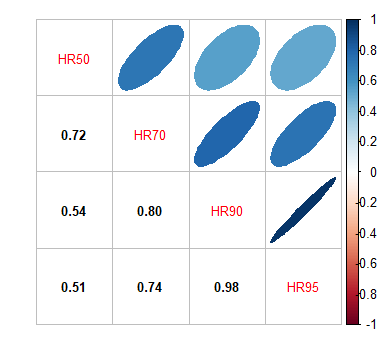

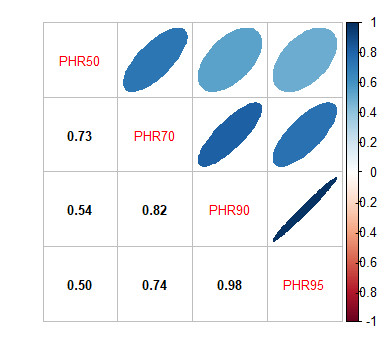


Mallard – Correlations among metrics for each potential isopleth level


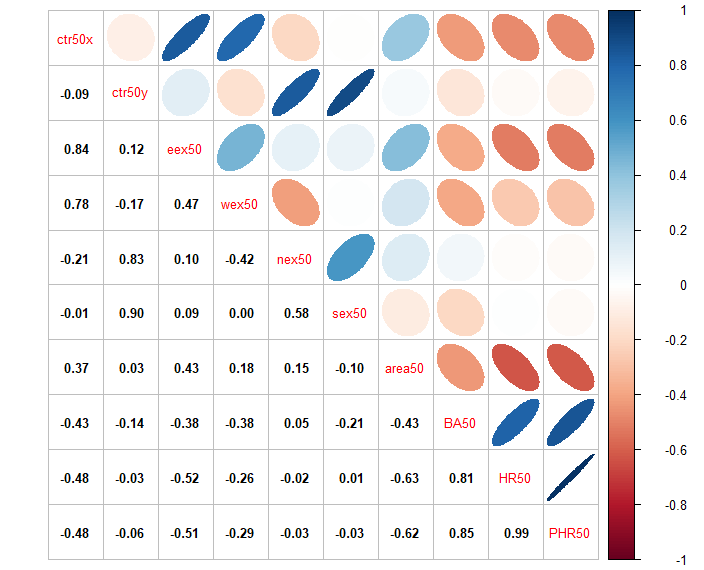


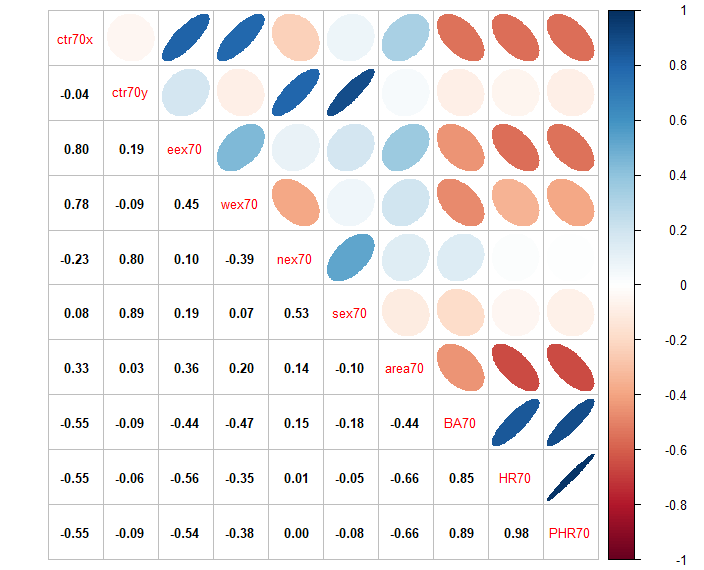


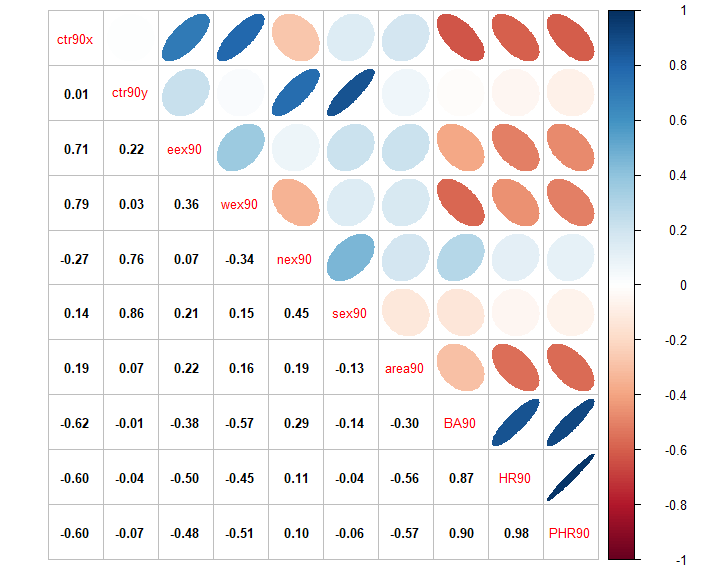


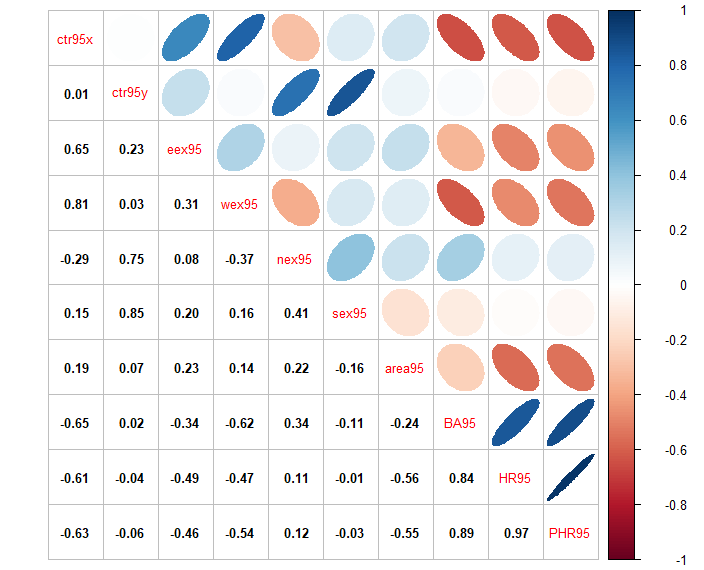


Mallards – Correlations among isopleth levels for each metric


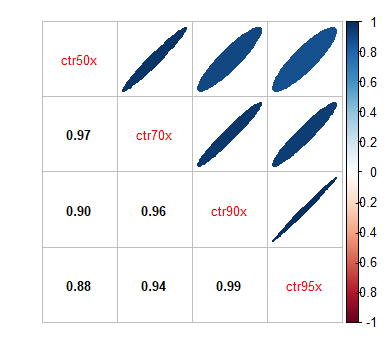

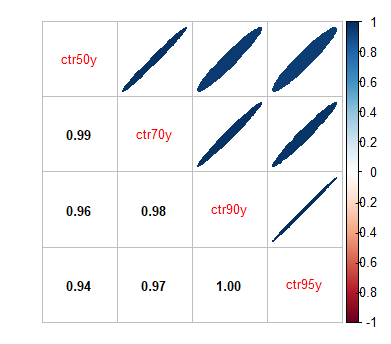


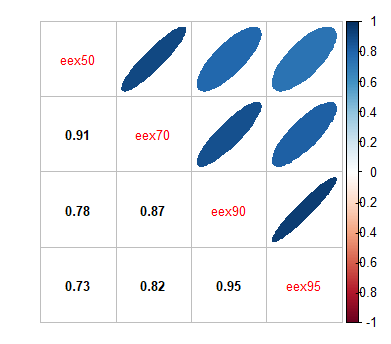

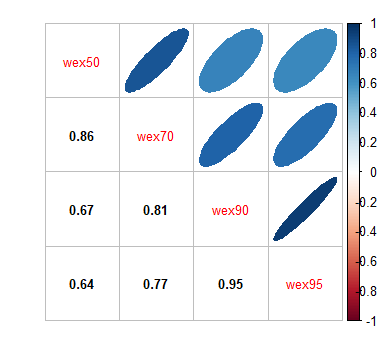


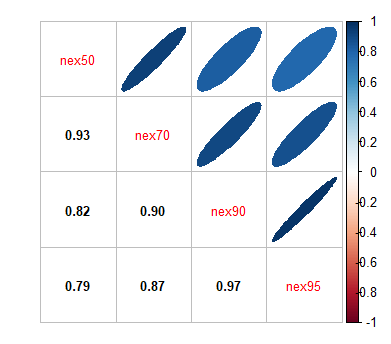

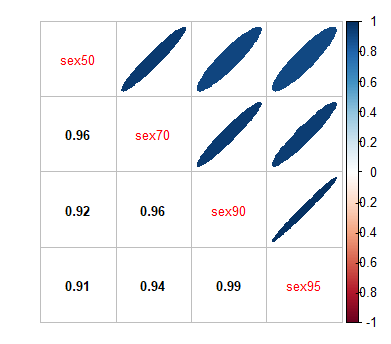


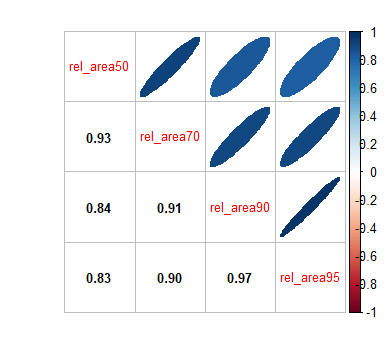

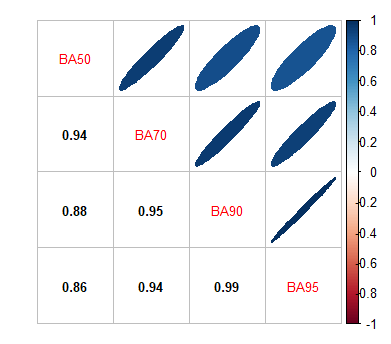


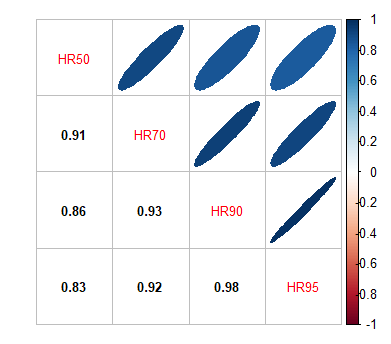

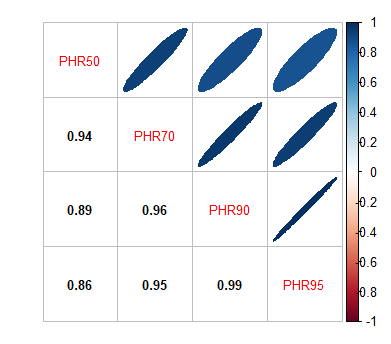


Northern Pintail – Correlations among metrics for each potential isopleth level


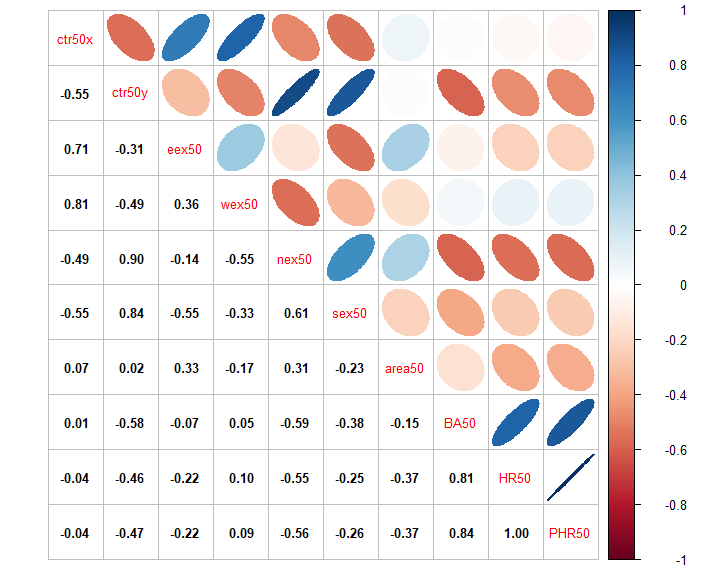

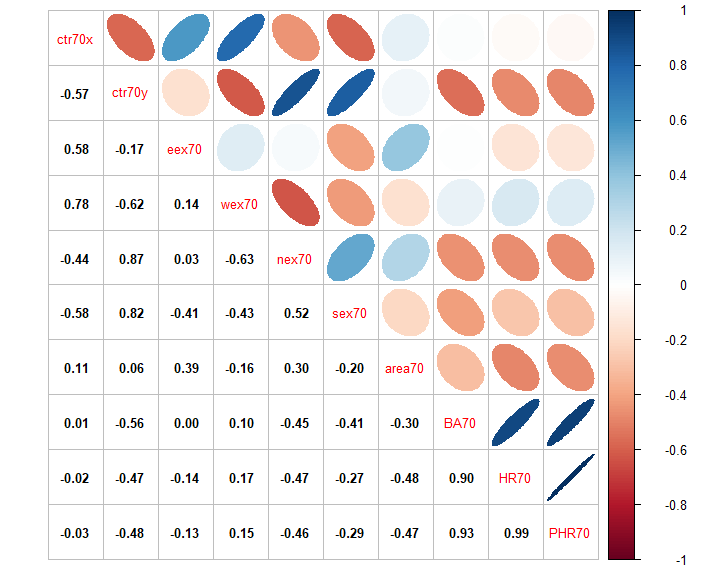


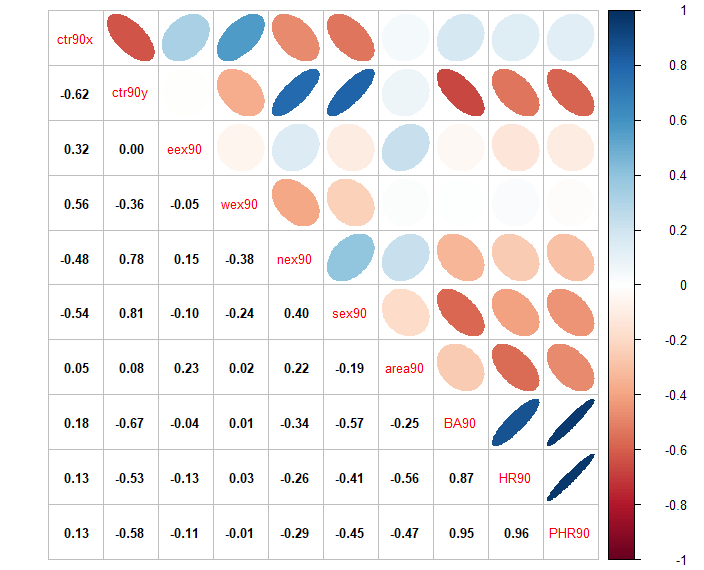


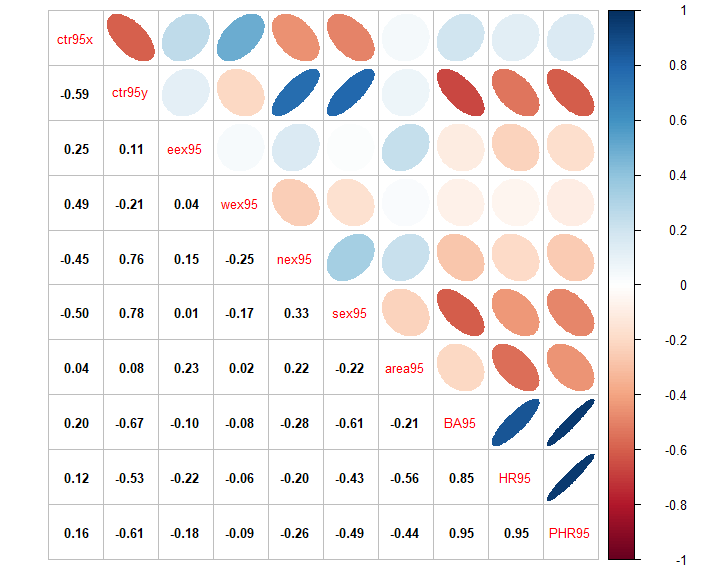


Northern Pintail – Correlations among isopleth levels for each metric


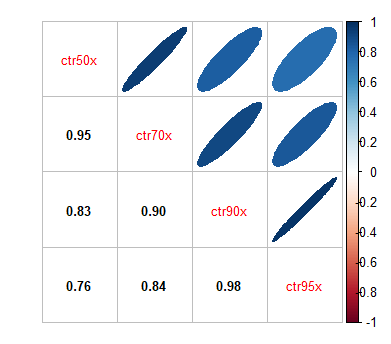

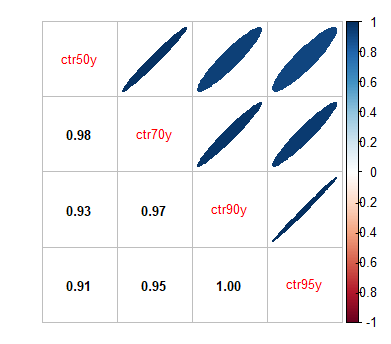


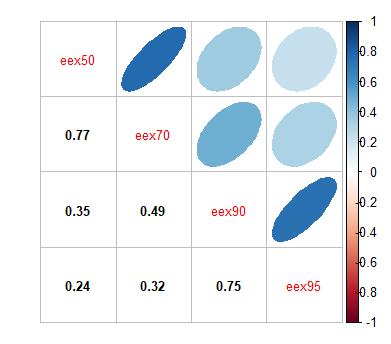

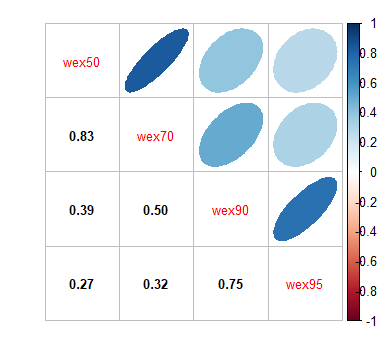


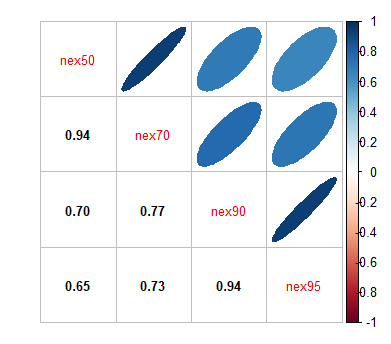

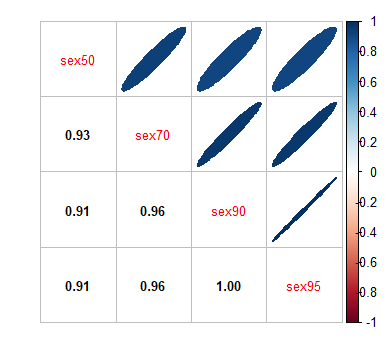


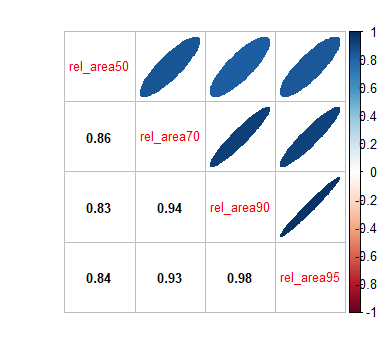

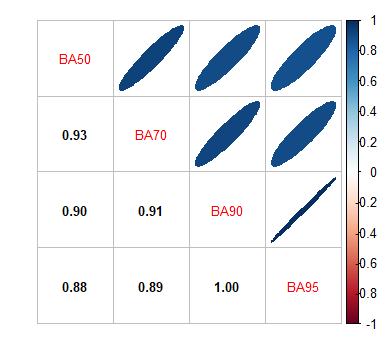


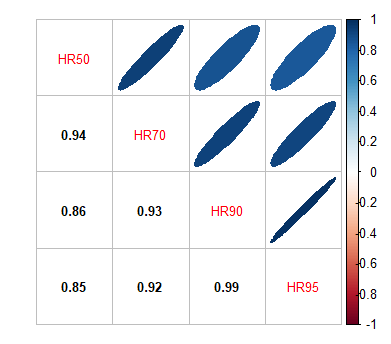

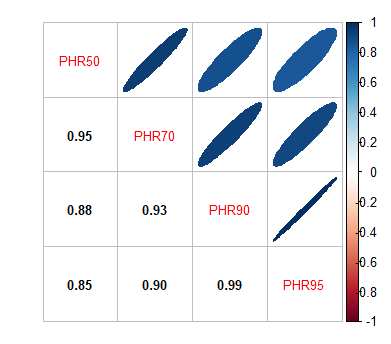


**Supplemental Figure 2.** Maps of 50% and 95% isopleths of kernel density estimator-based utilization distributions of band recoveries from blue-winged teal (BWTE), mallards (MALL), and northern pintail (NOPI) in the Central and Mississippi Flyways of North America between 1960 and 2019, split by month of recovery (October–January) and banding region. Shown are isopleths (polygons), centroid locations (points), and sample sizes (N) of bands recovered during 1960–1969 (gray/black) or 2010–2019 (red). Geographically defined banding regions include the Prairie Habitat and Western Boreal Area Joint Ventures (PHWB), the Prairie Pothole and Northern Great Plains Joint Ventures (PPNP), and the Upper Mississippi River / Great Lakes Joint Venture and Ontario (UMON; mallard only).

Blue-winged teal – 1960–1969 versus 2010–2019 – 50% isopleths


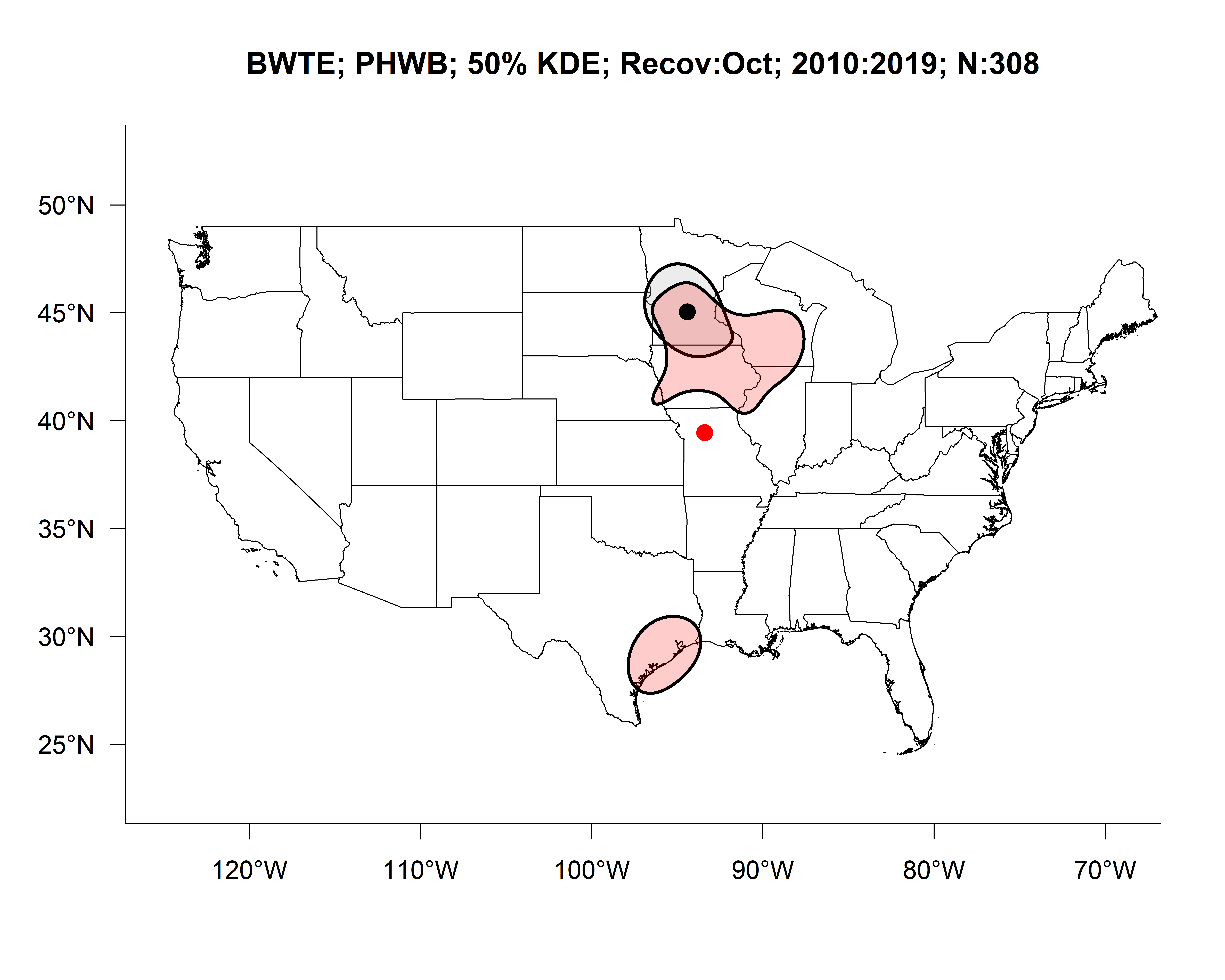

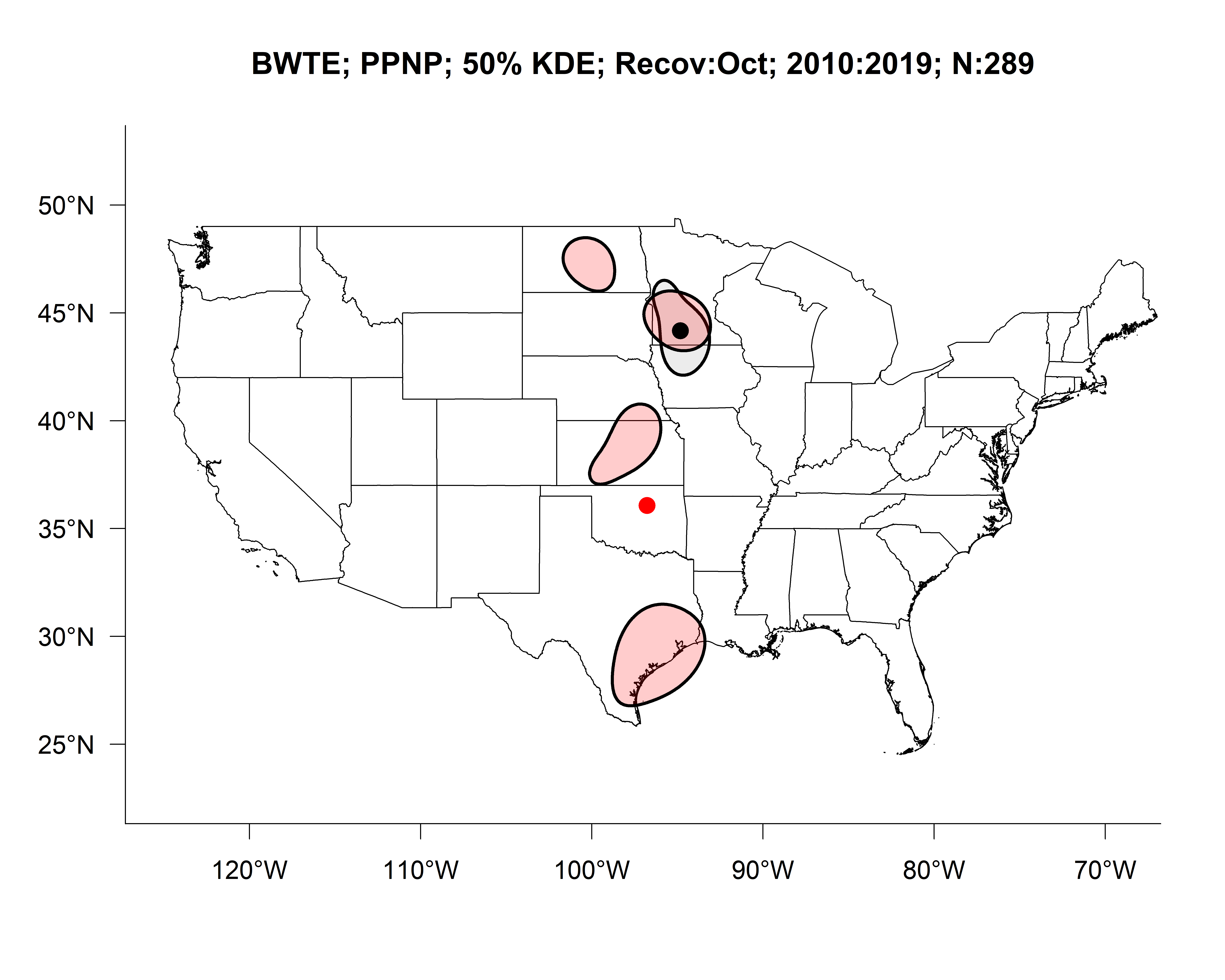

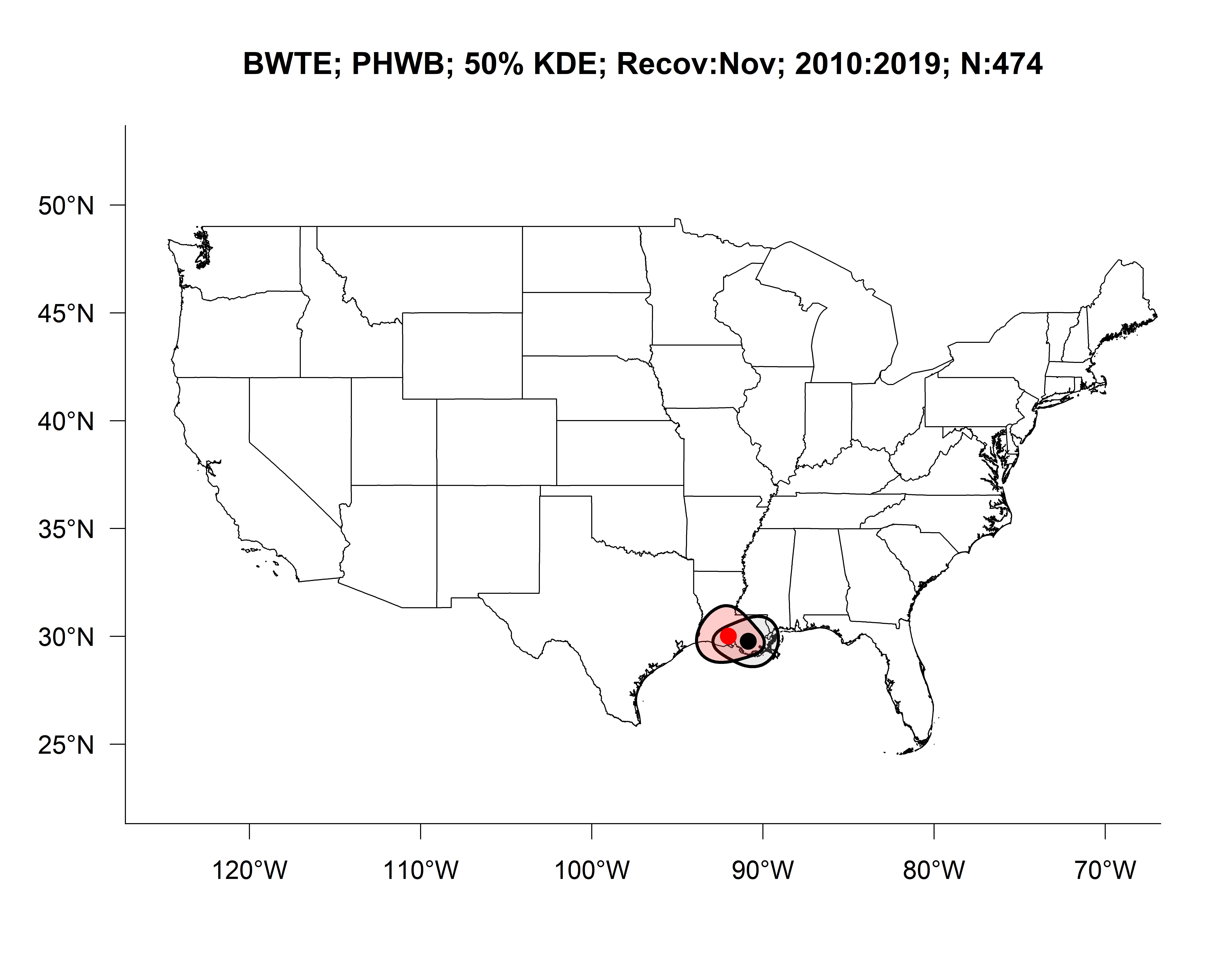

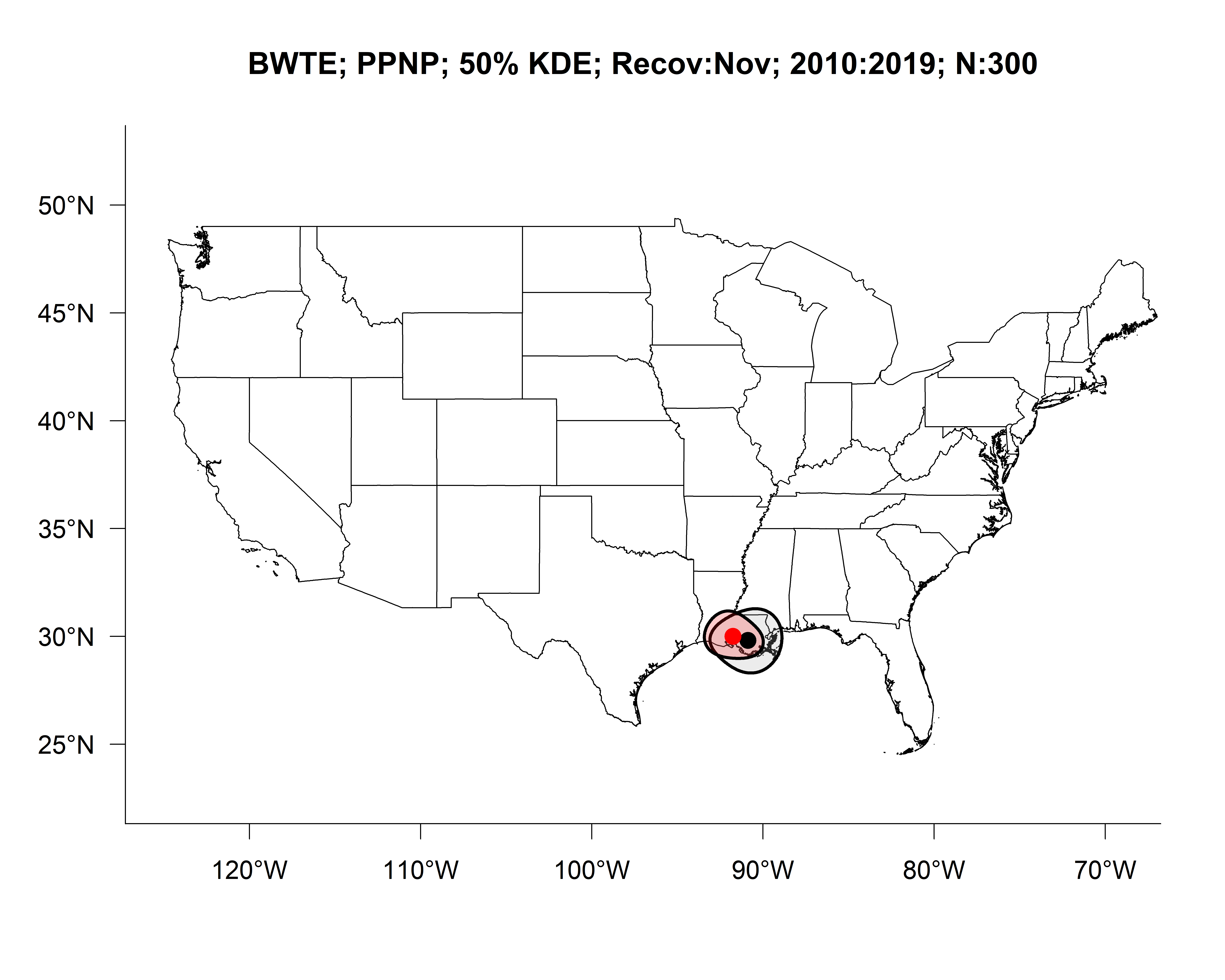

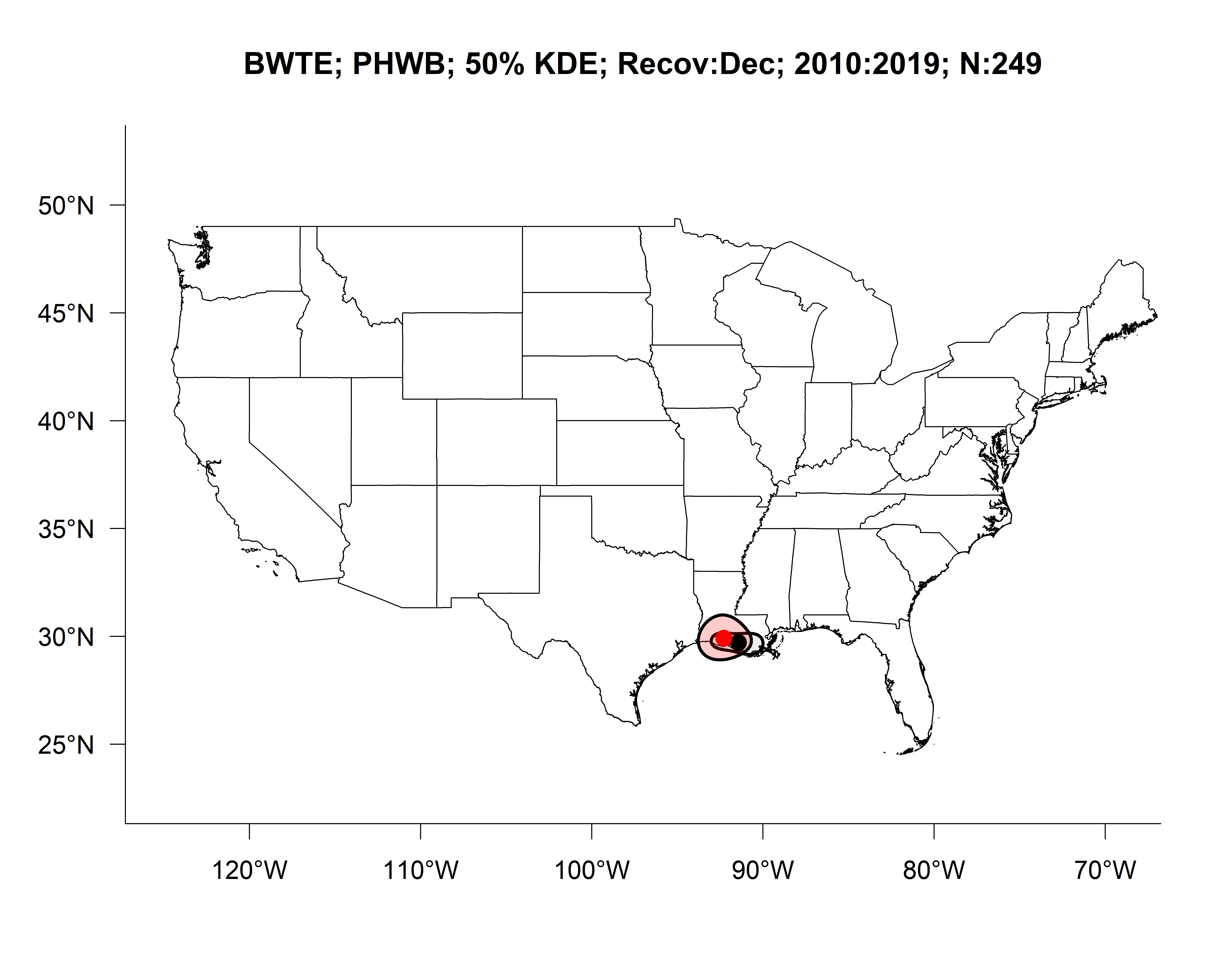

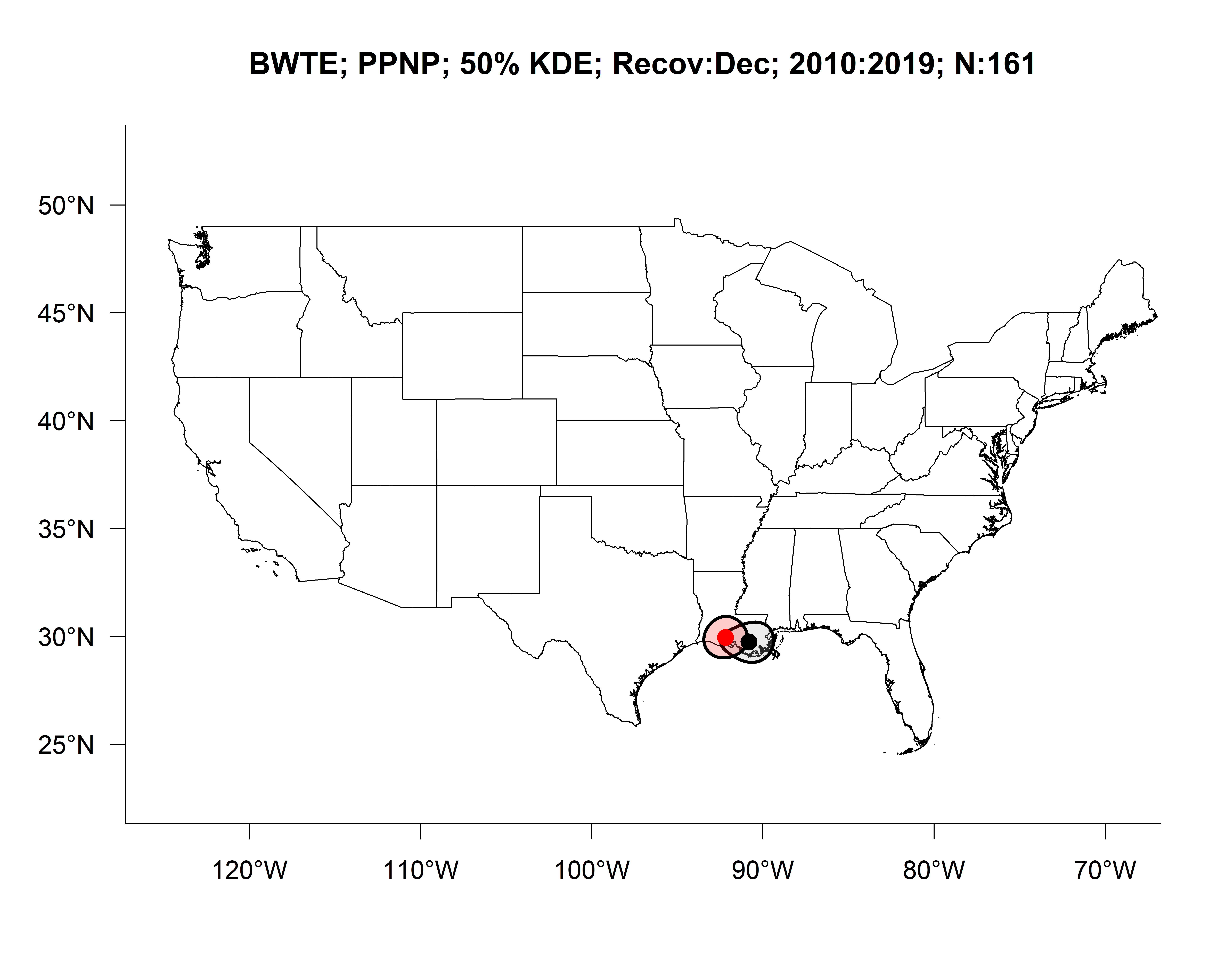

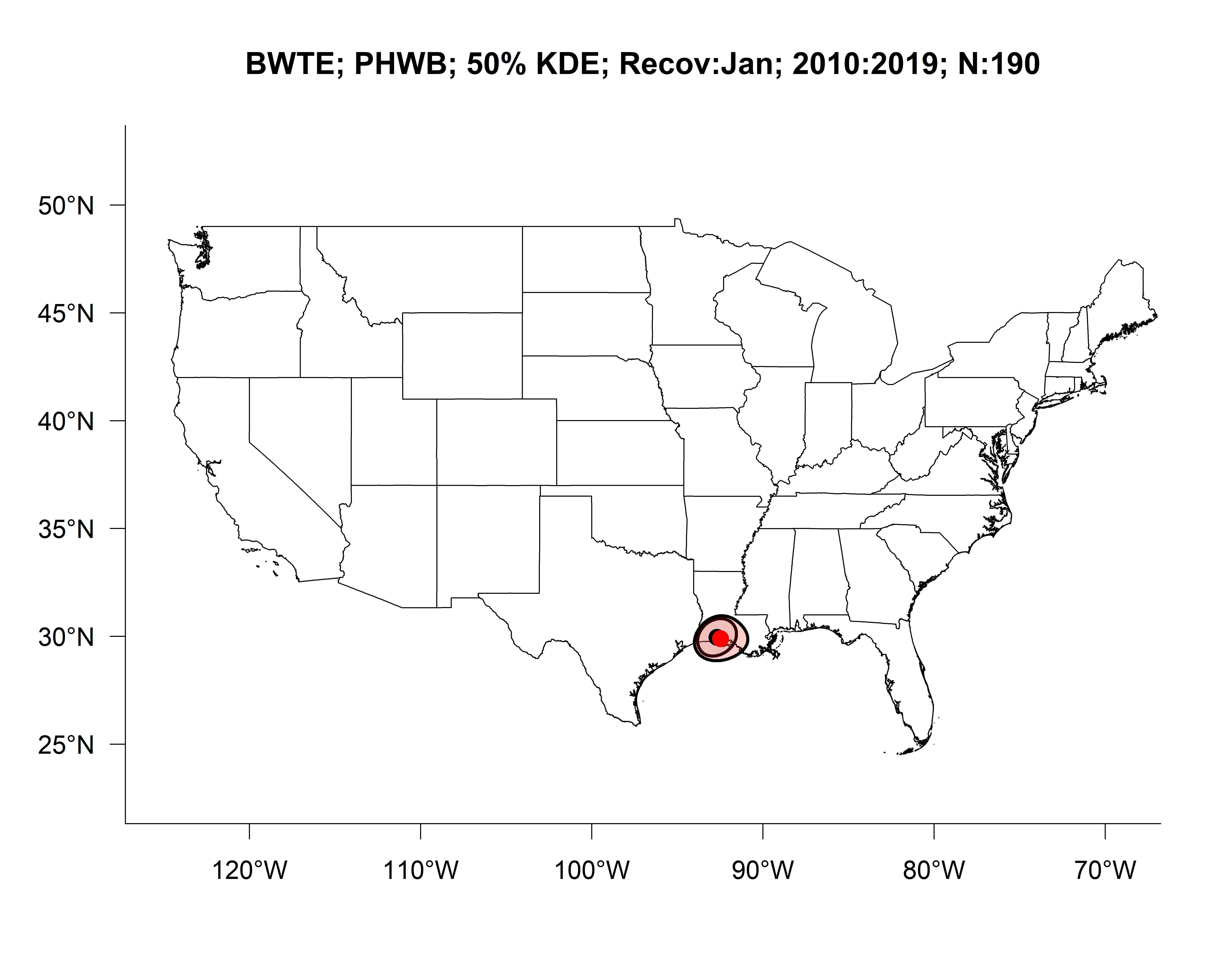

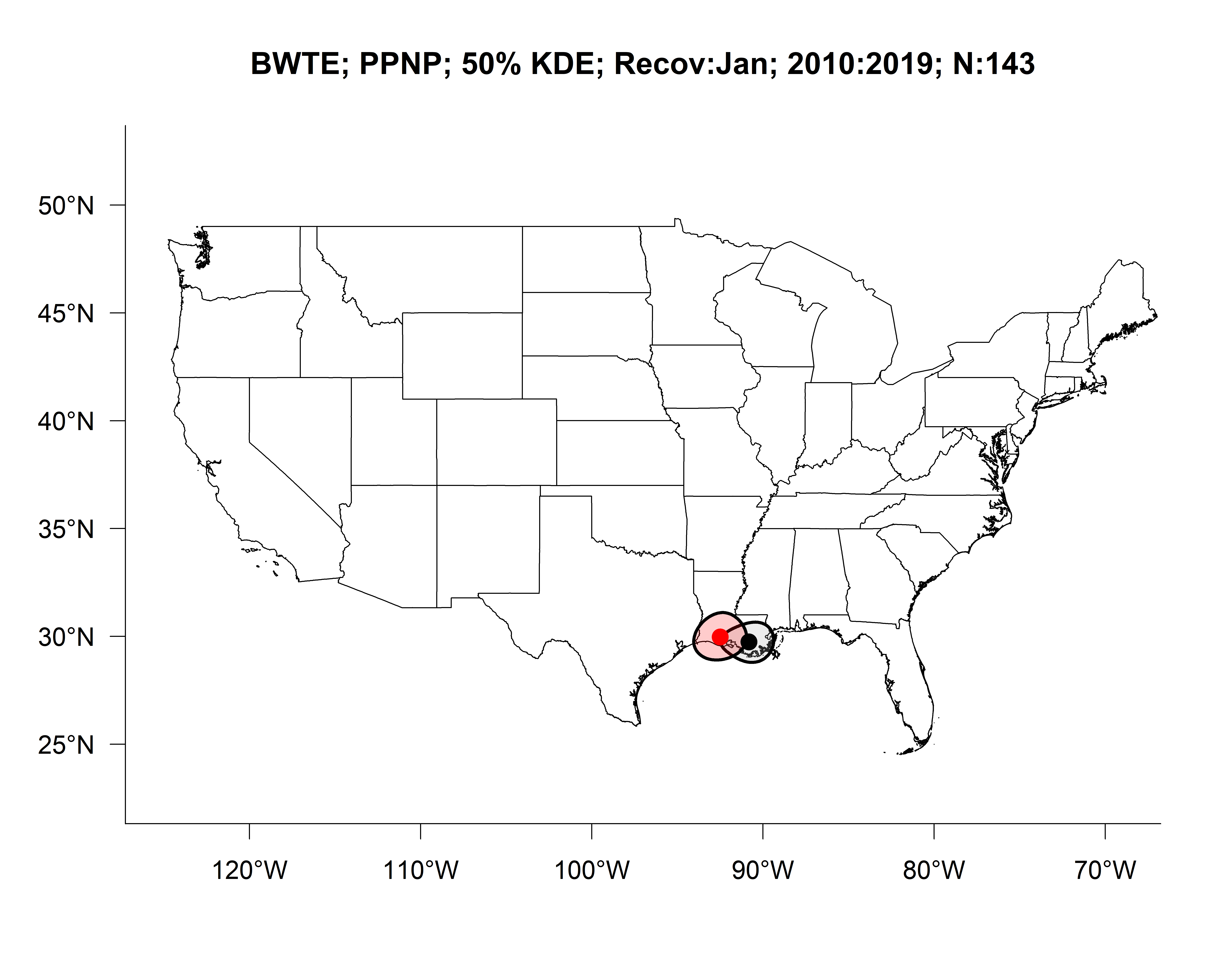


Blue-winged teal – 1960–1969 versus 2010–2019 – 95% isopleths


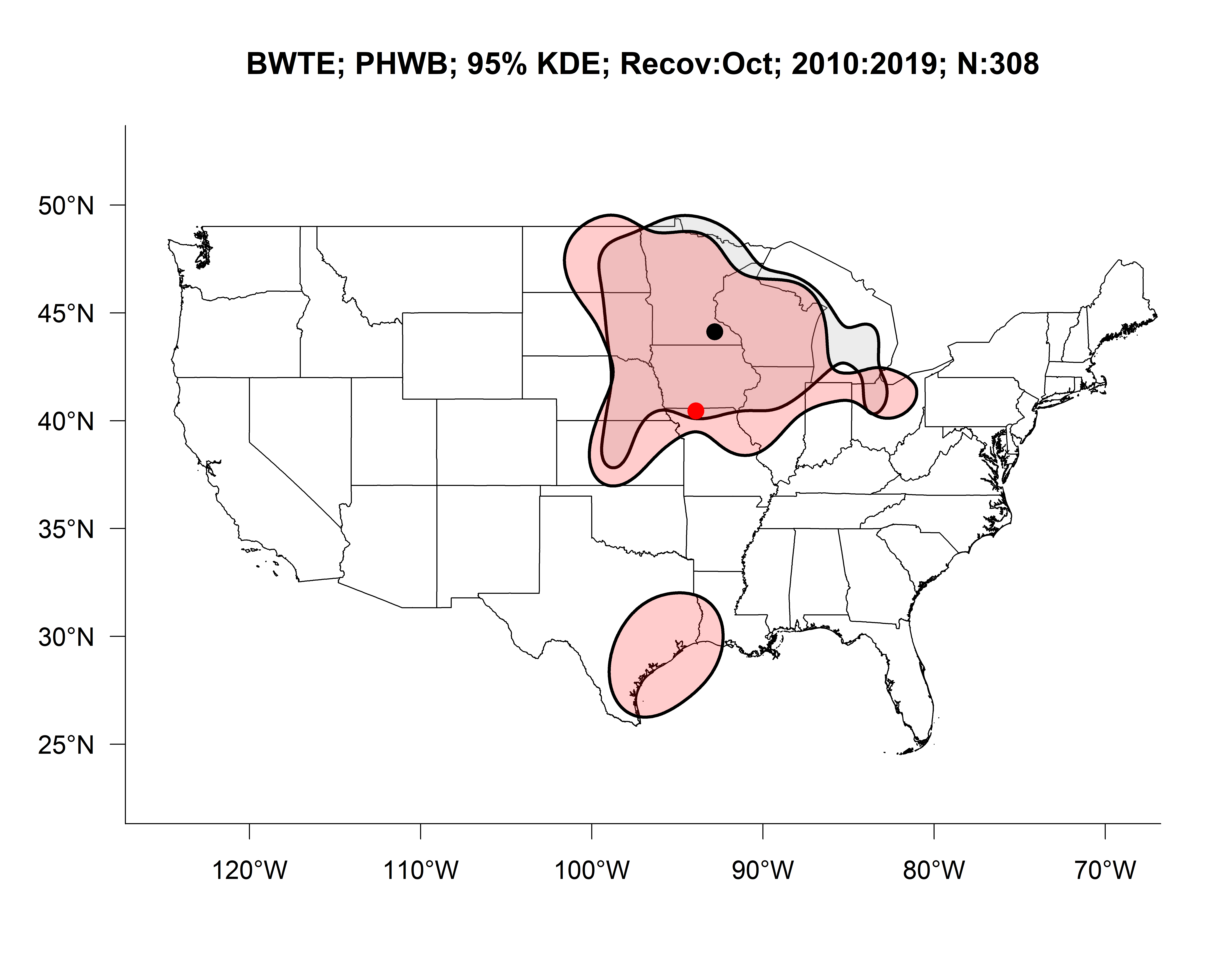

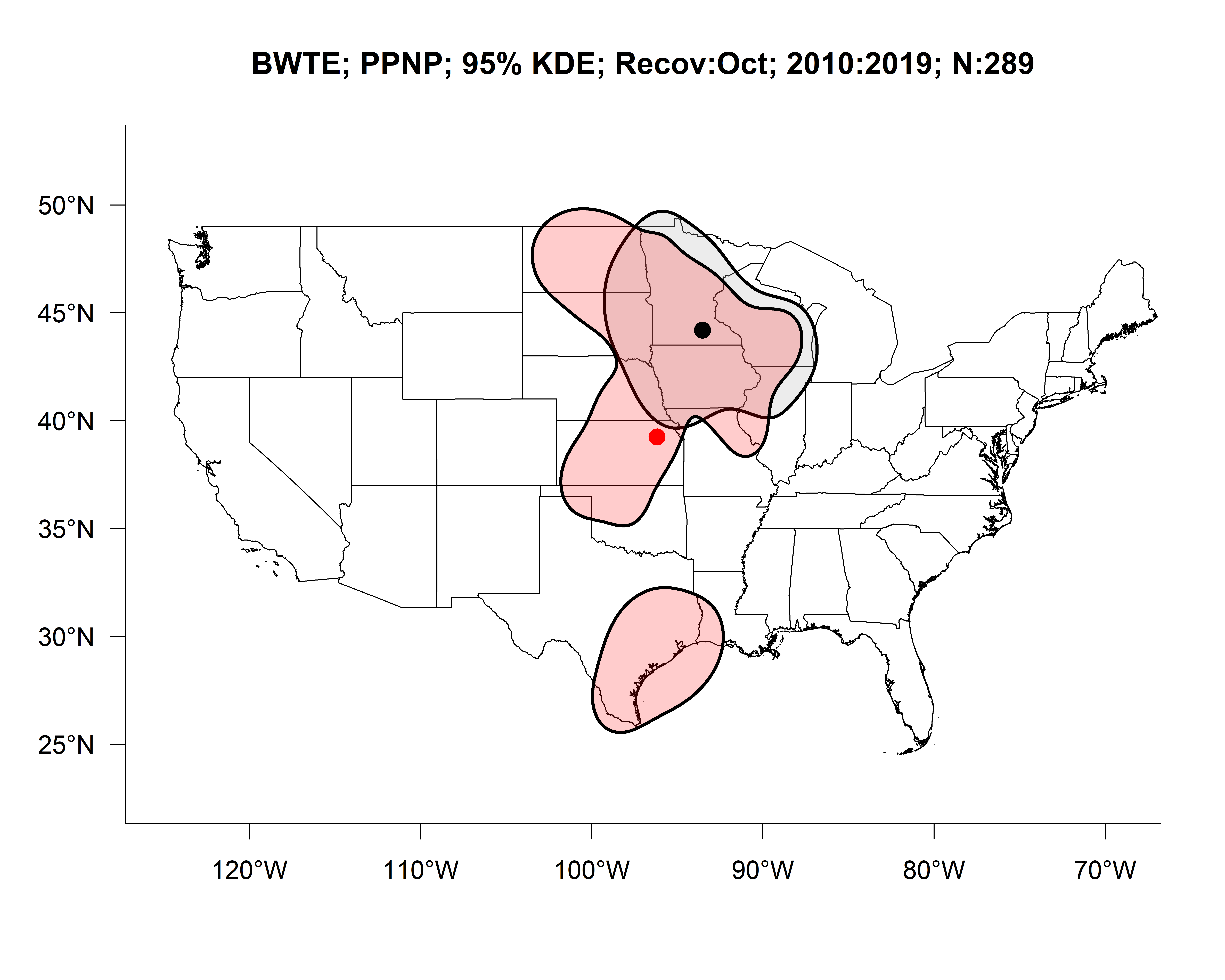

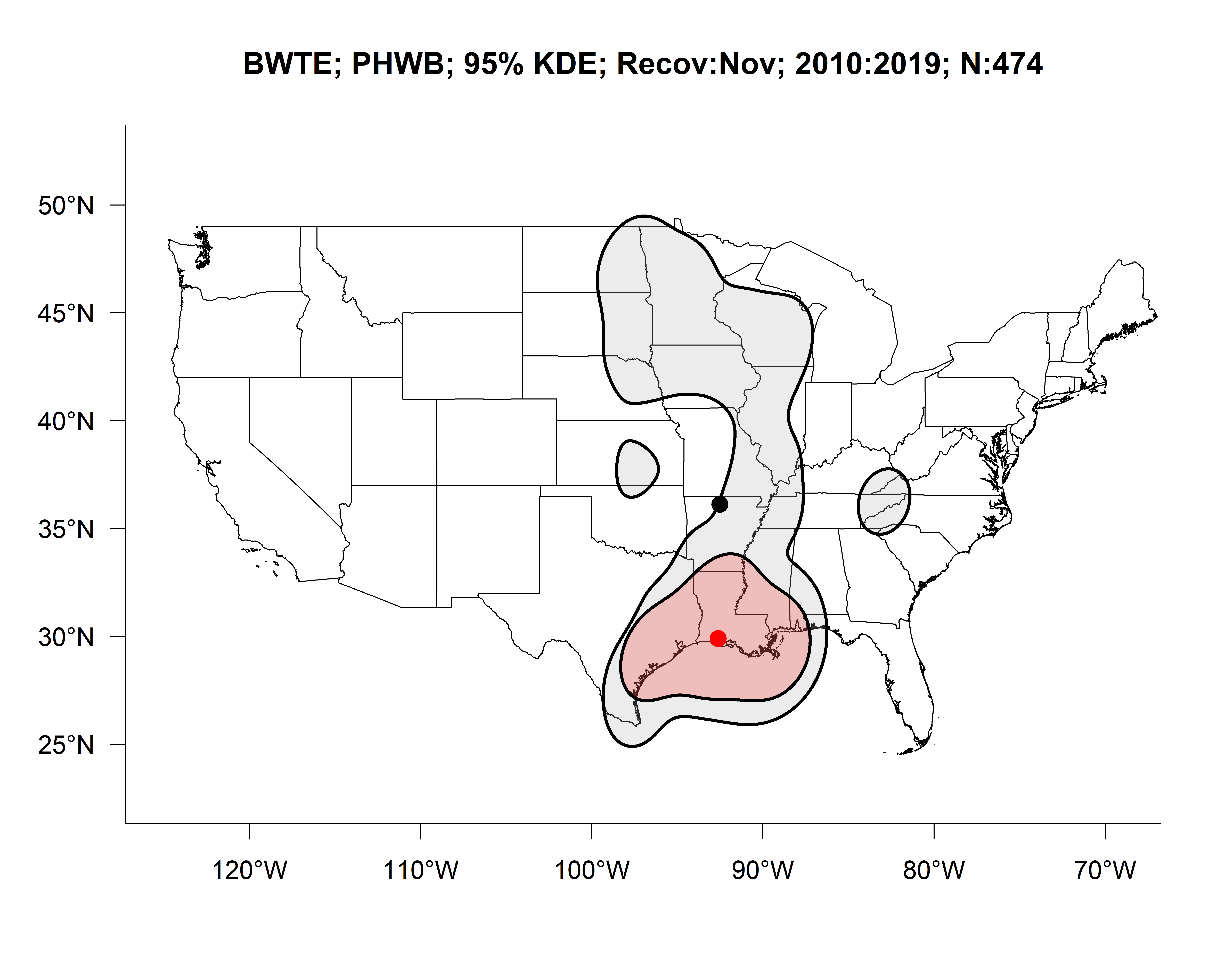

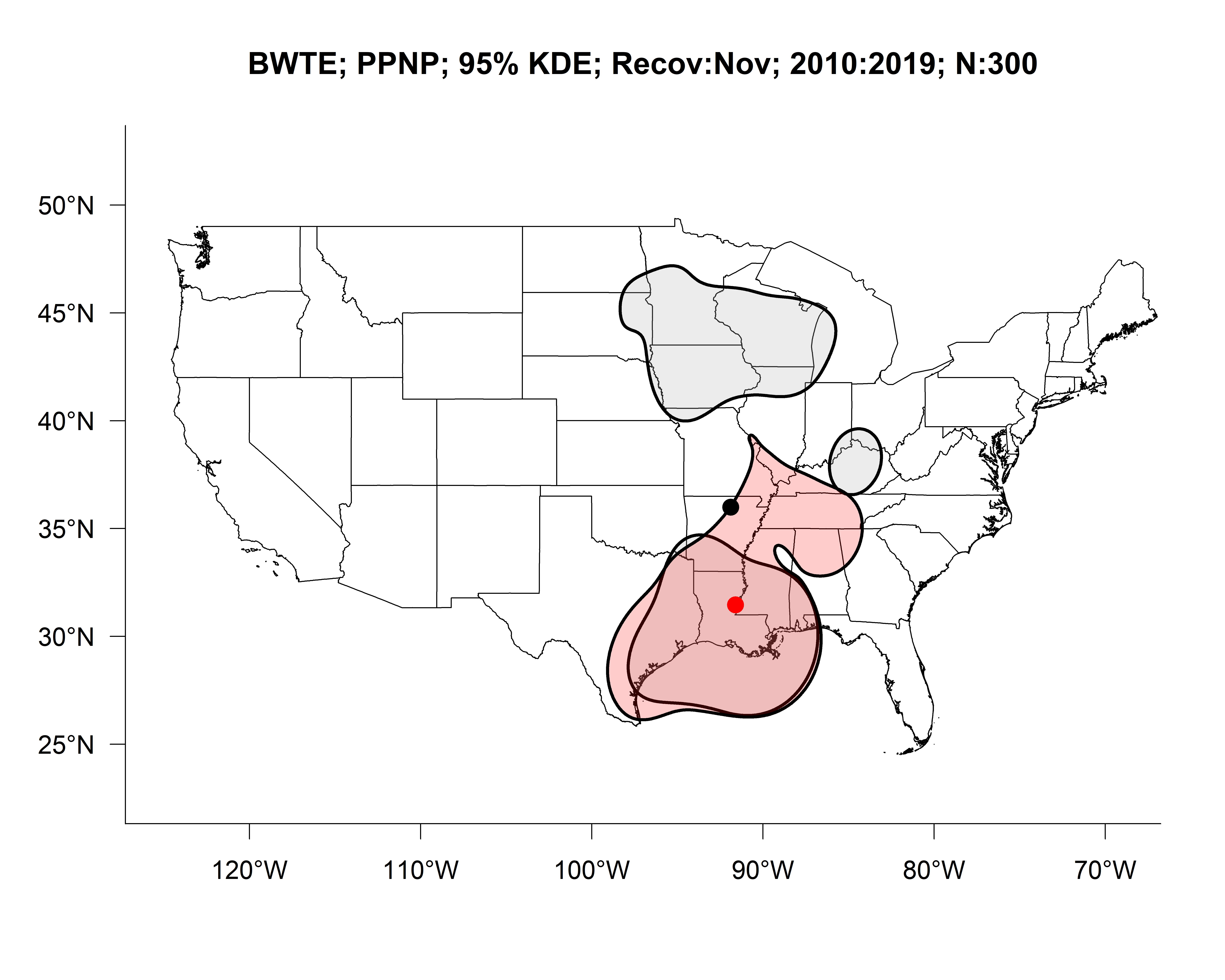

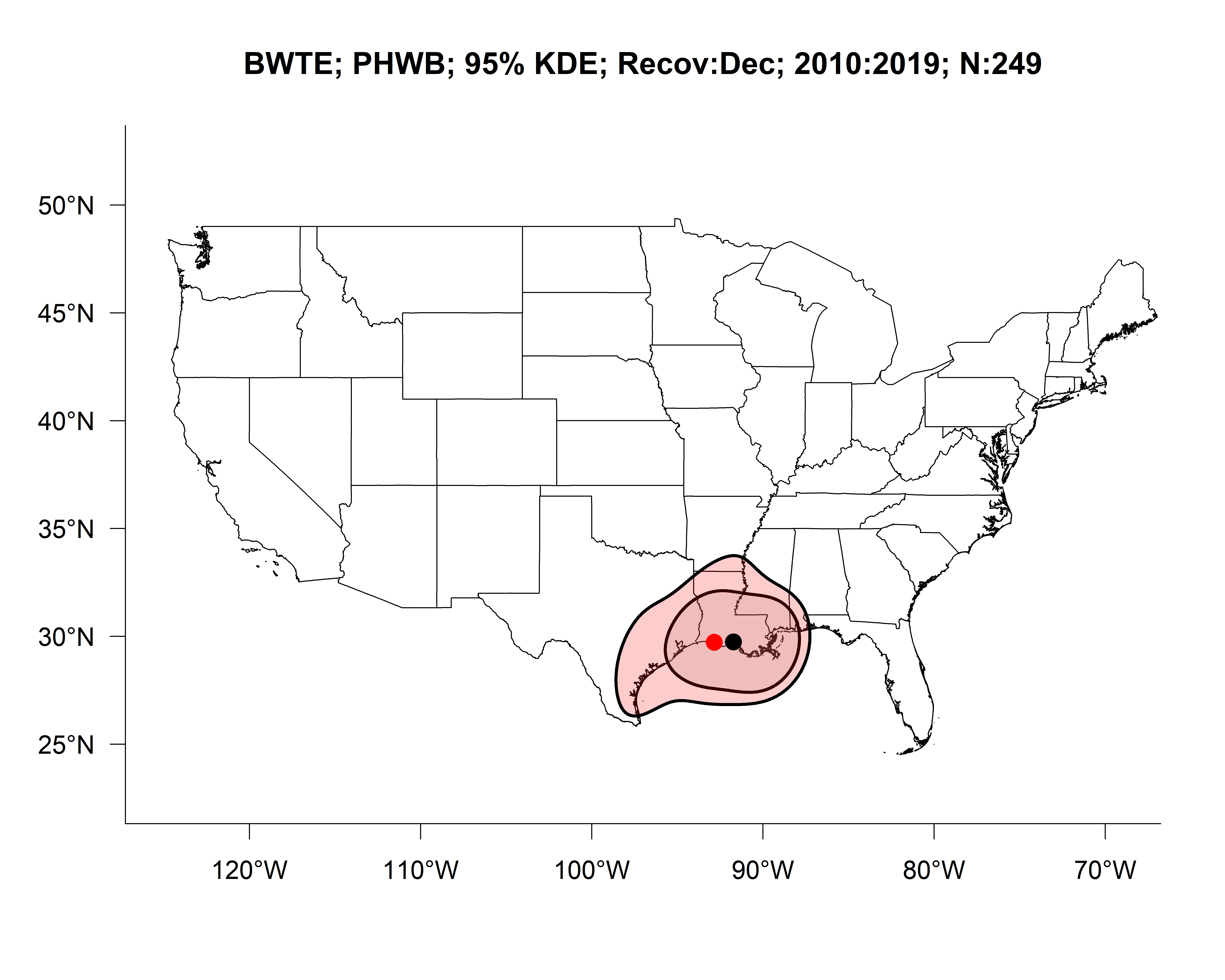

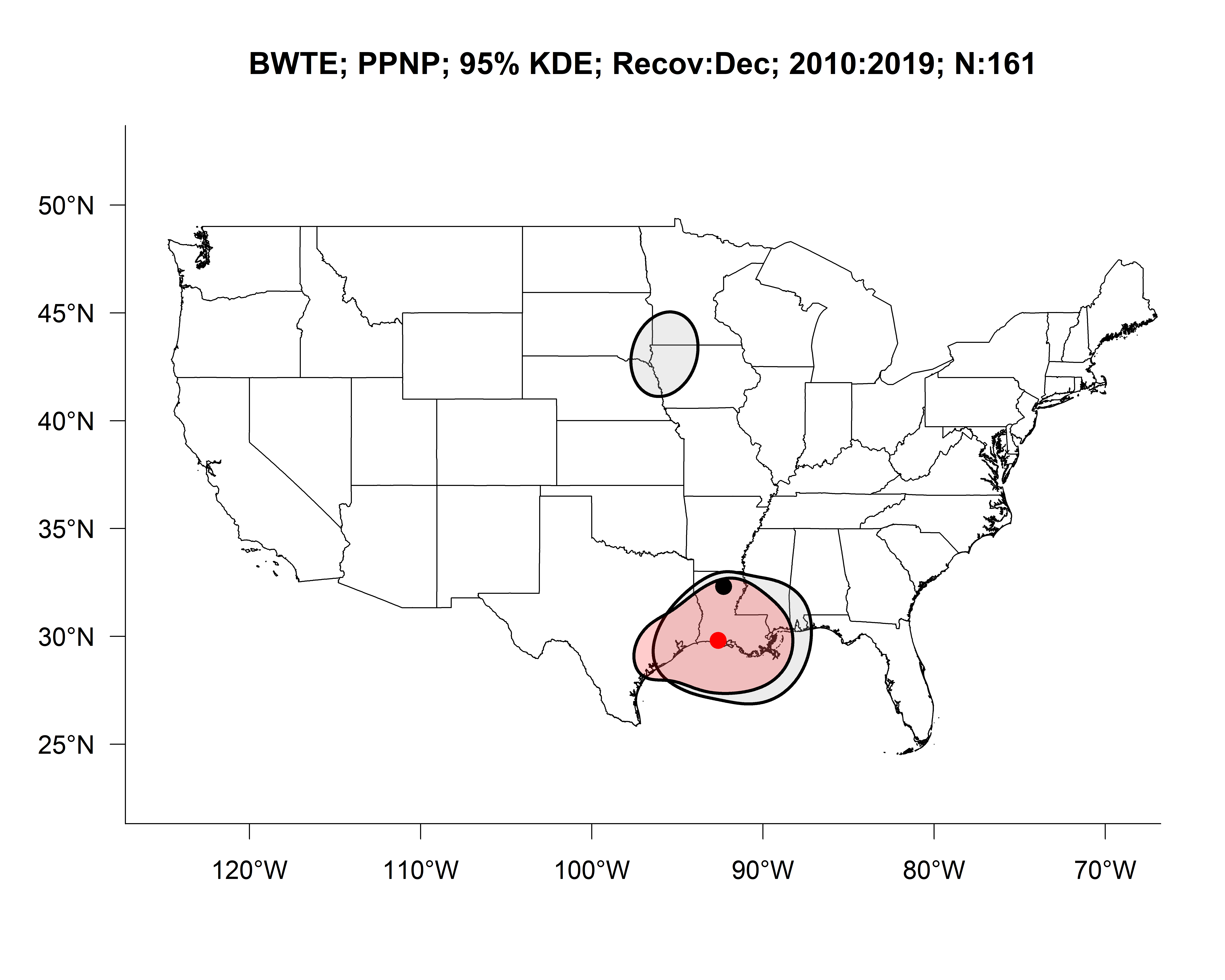

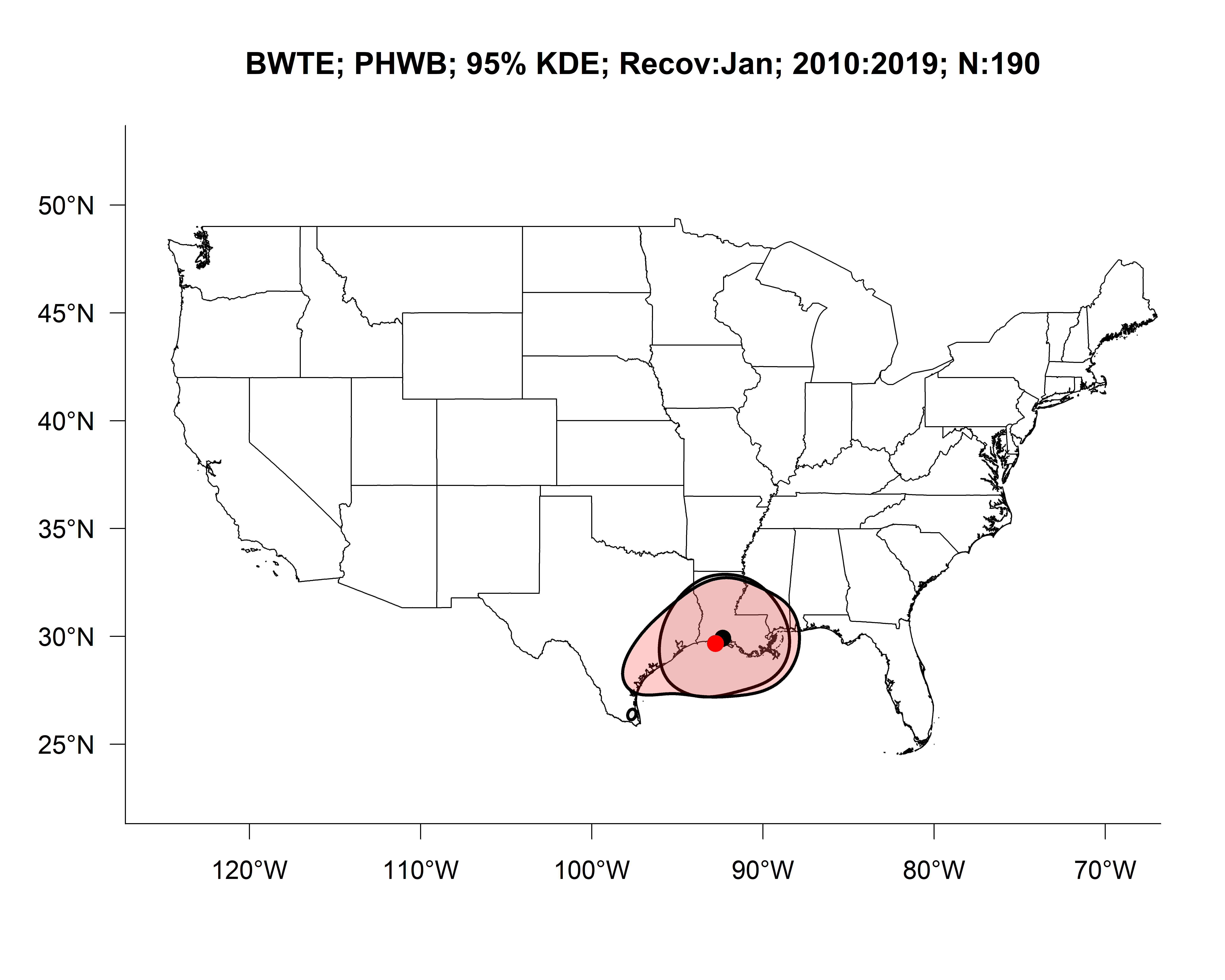

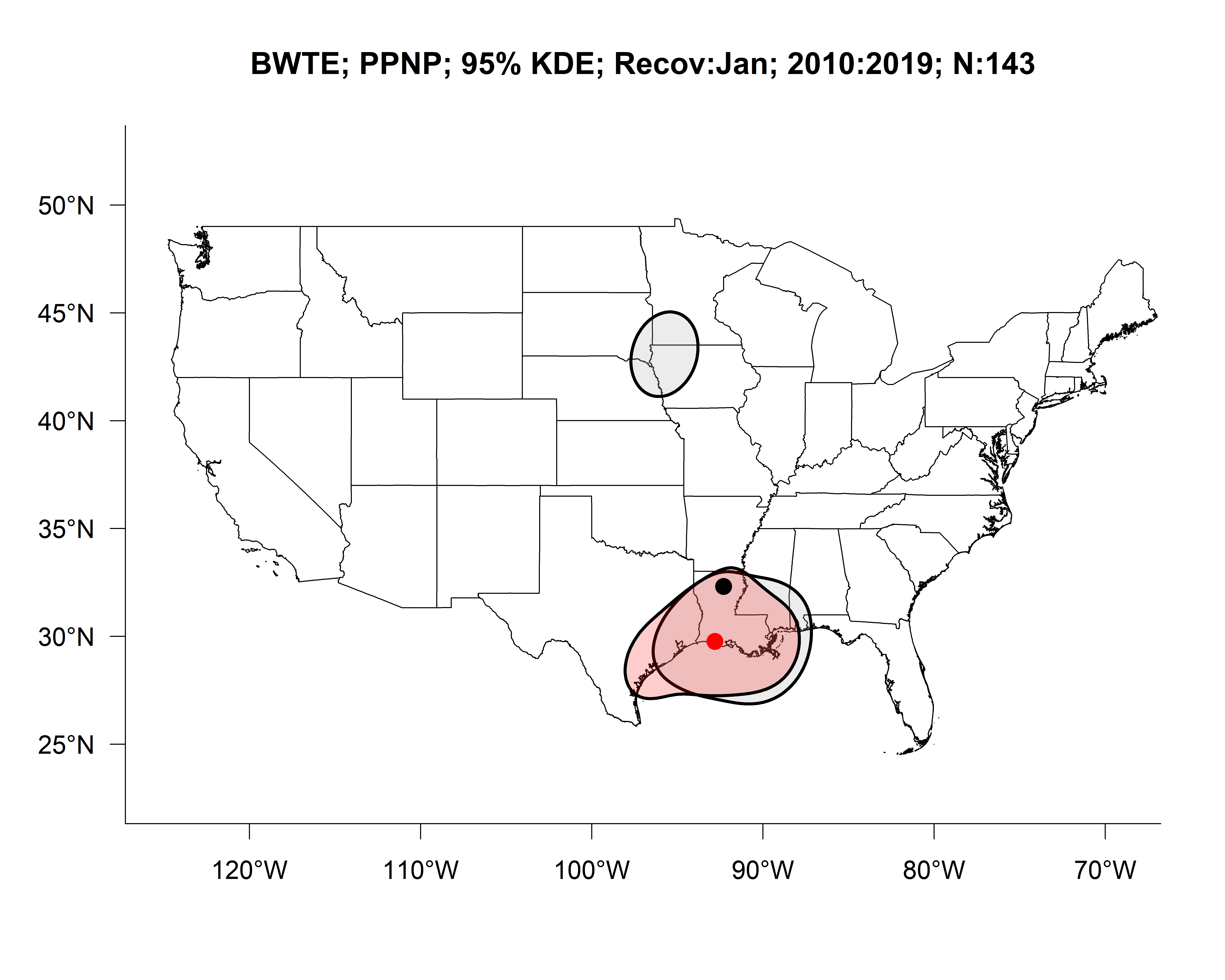


Mallard – 1960–1969 versus 2010–2019 – 50% isopleths


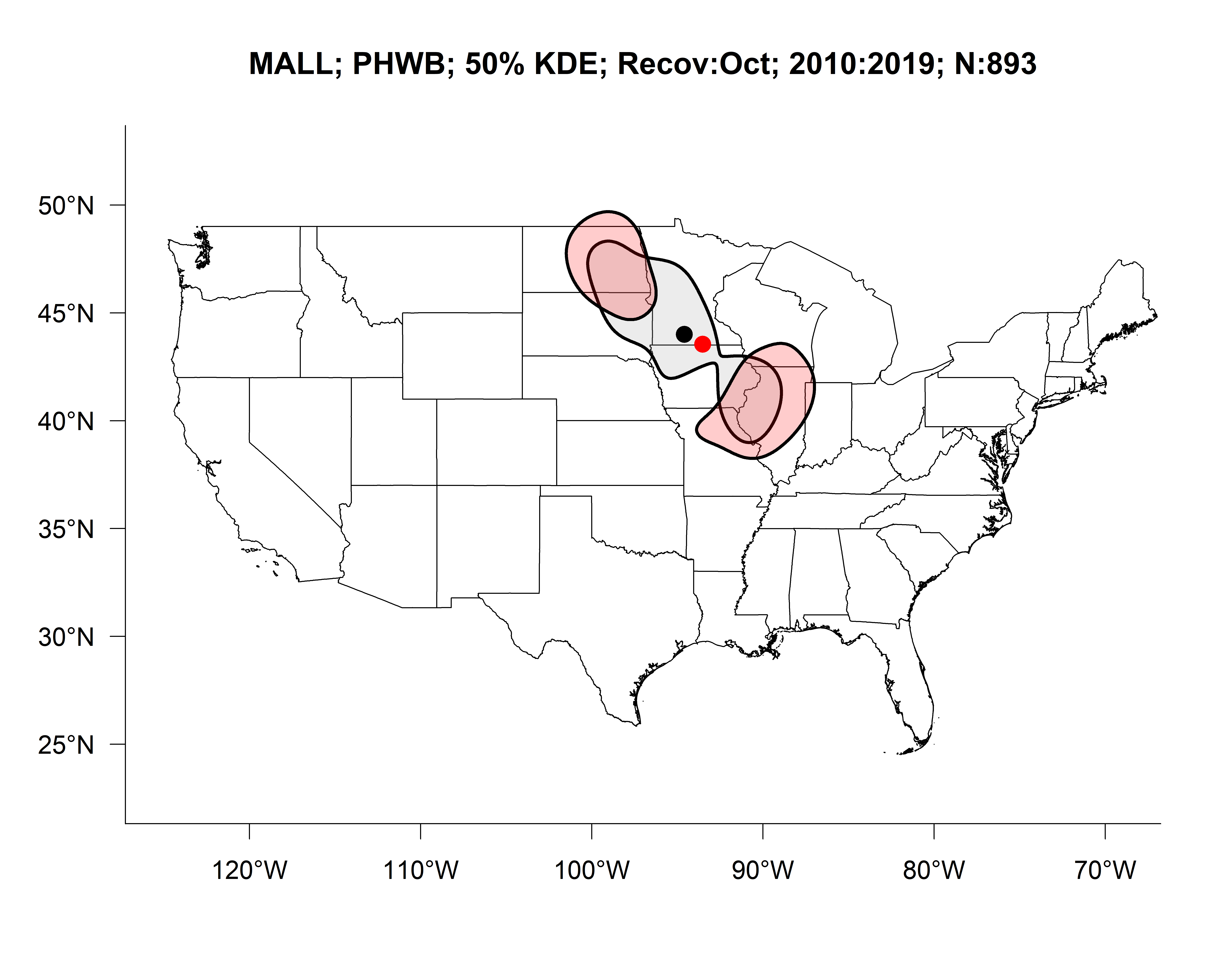

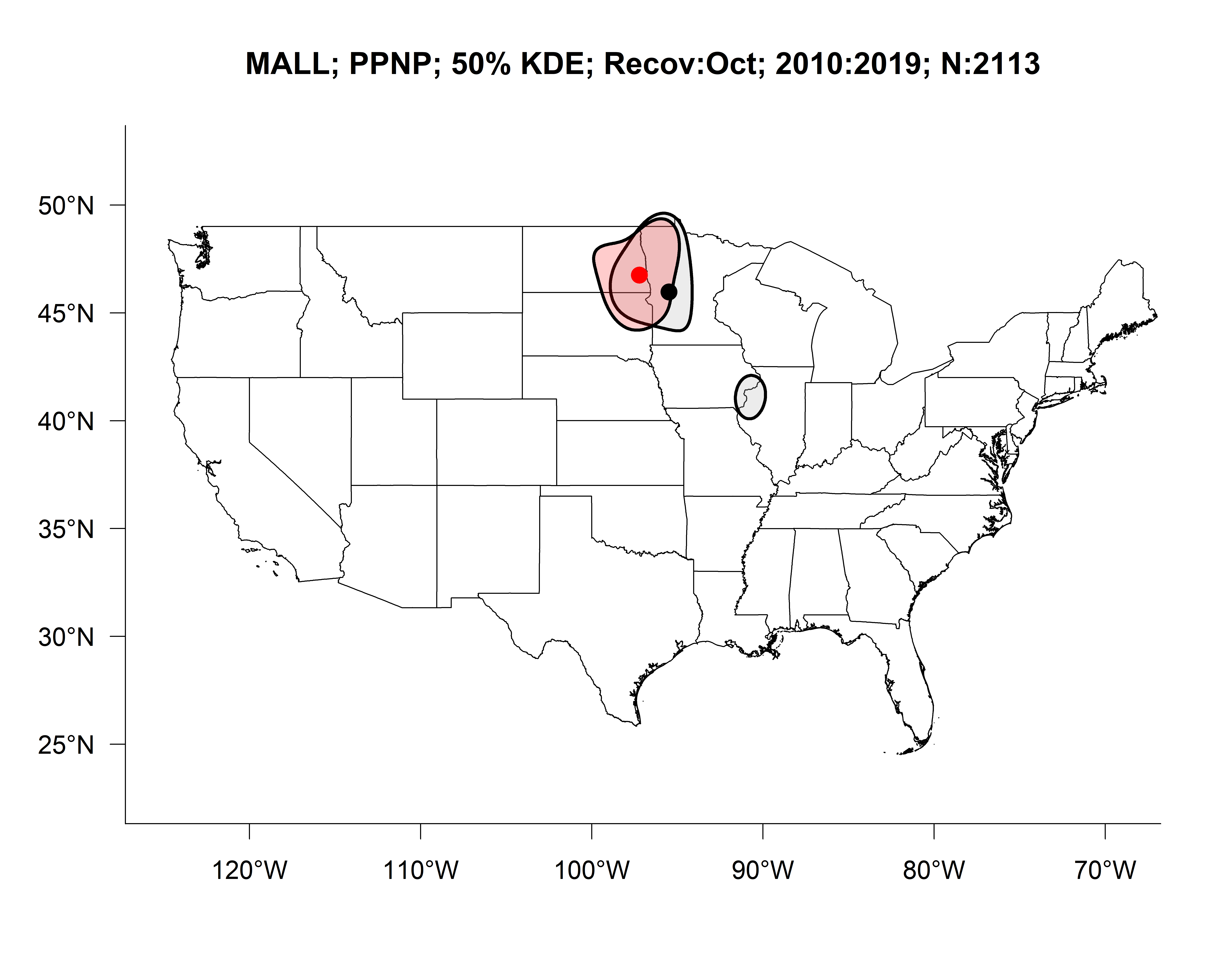

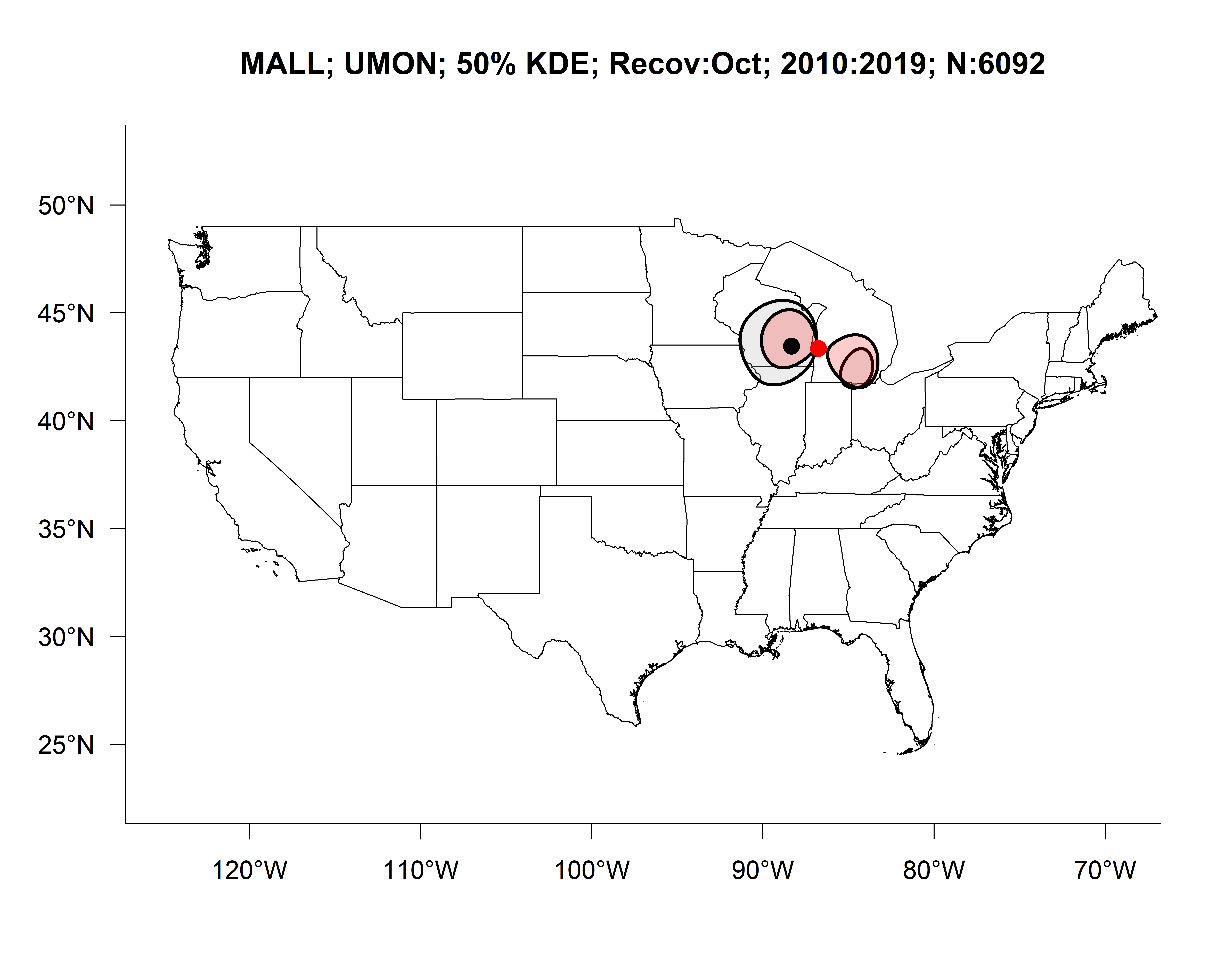

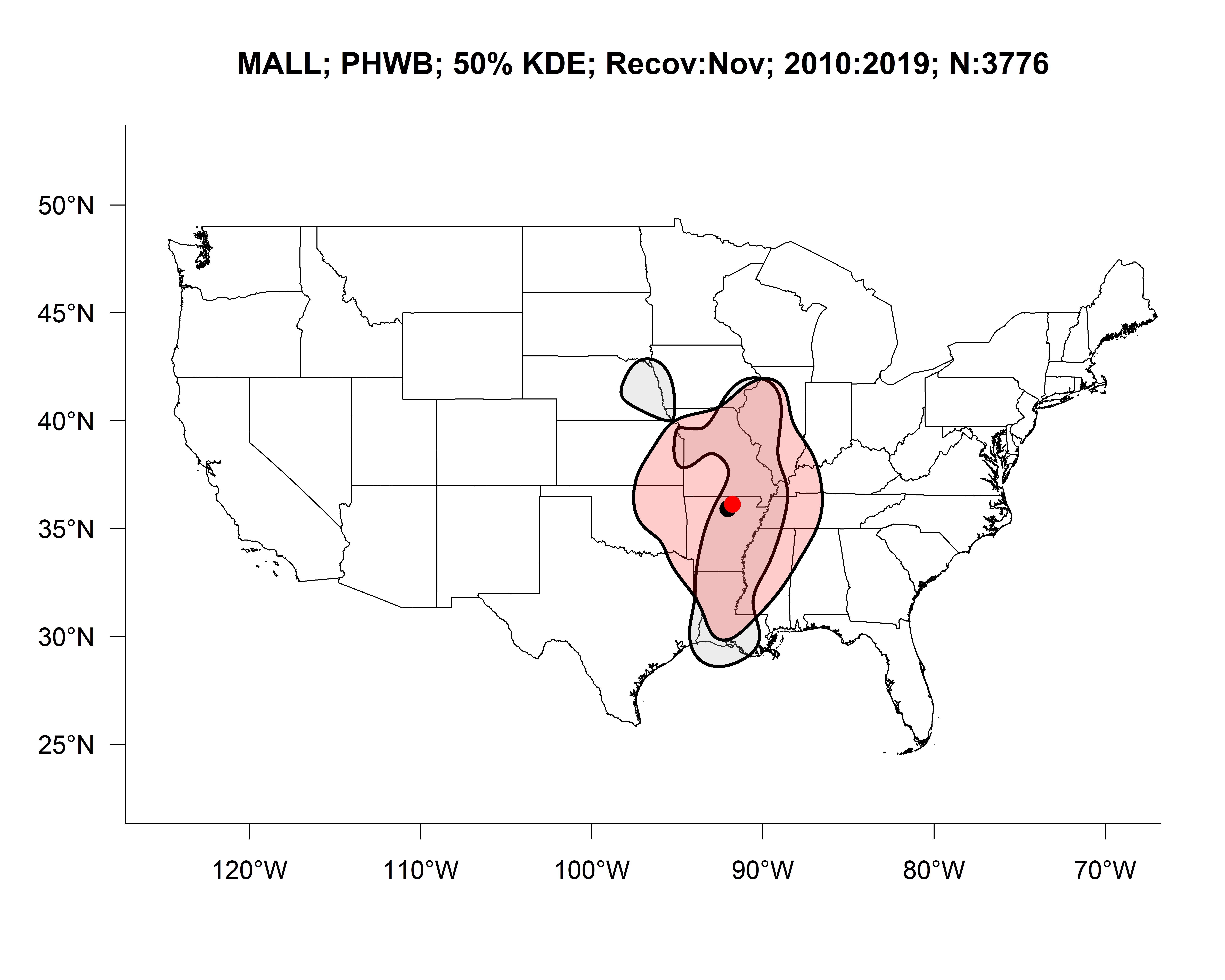

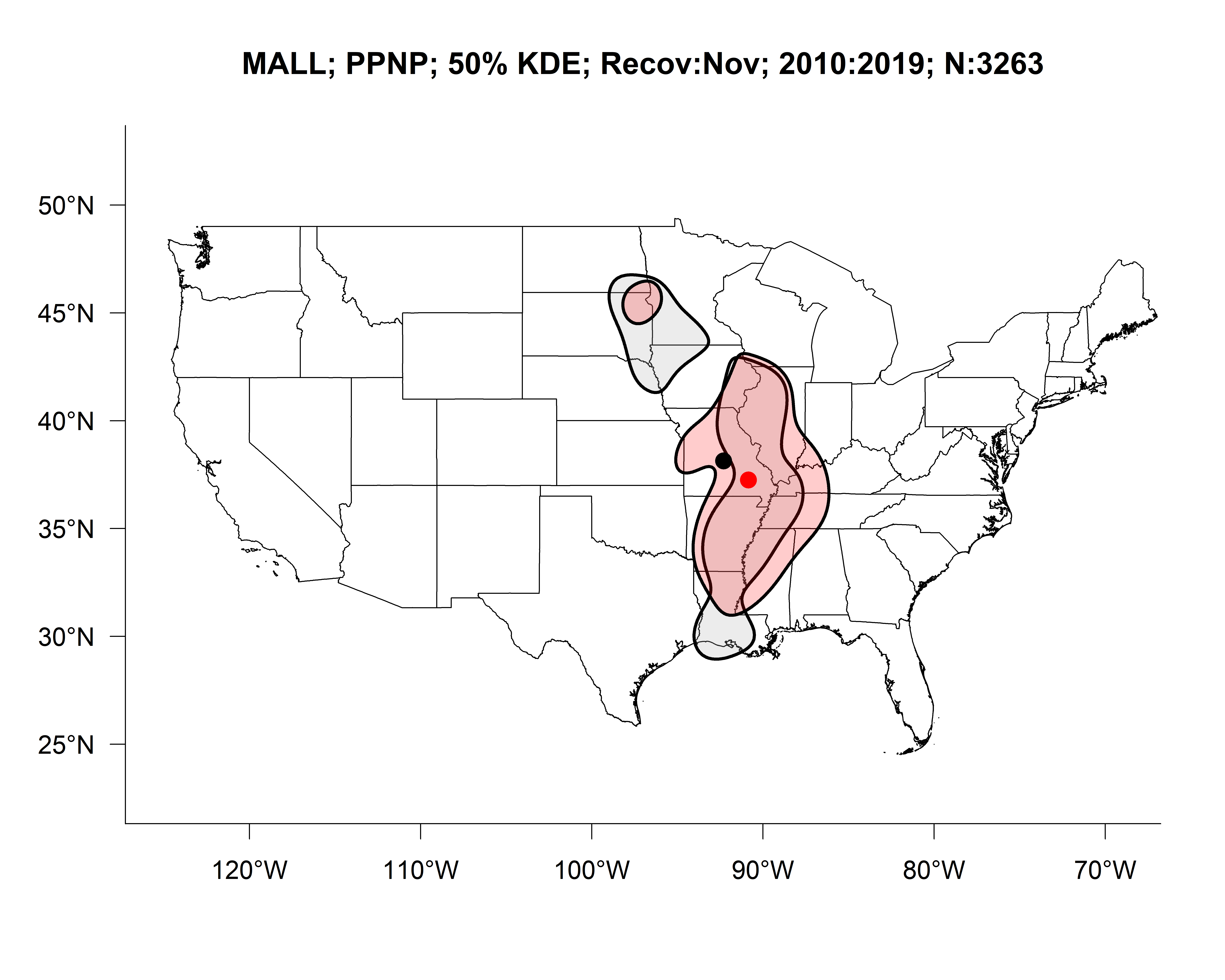

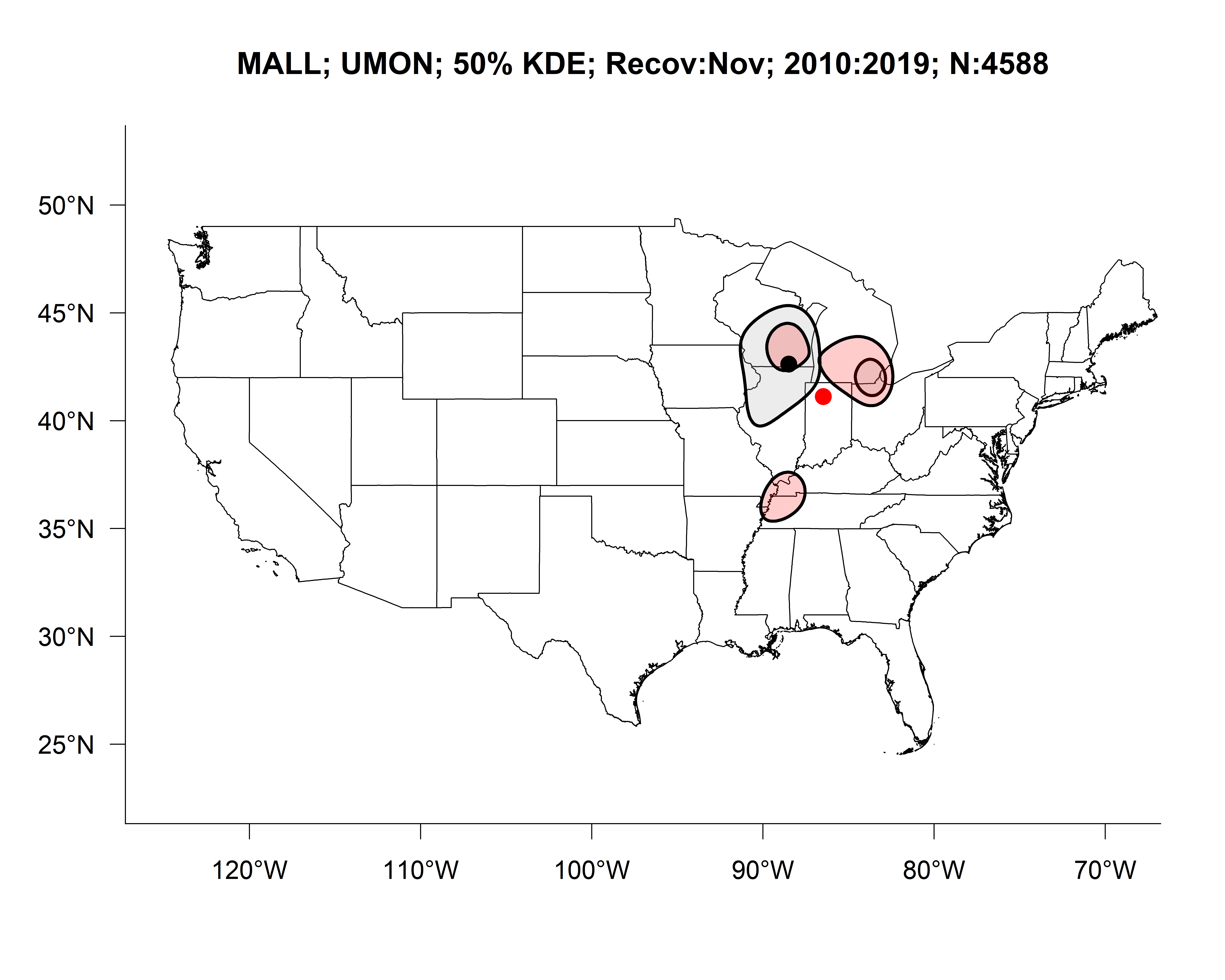

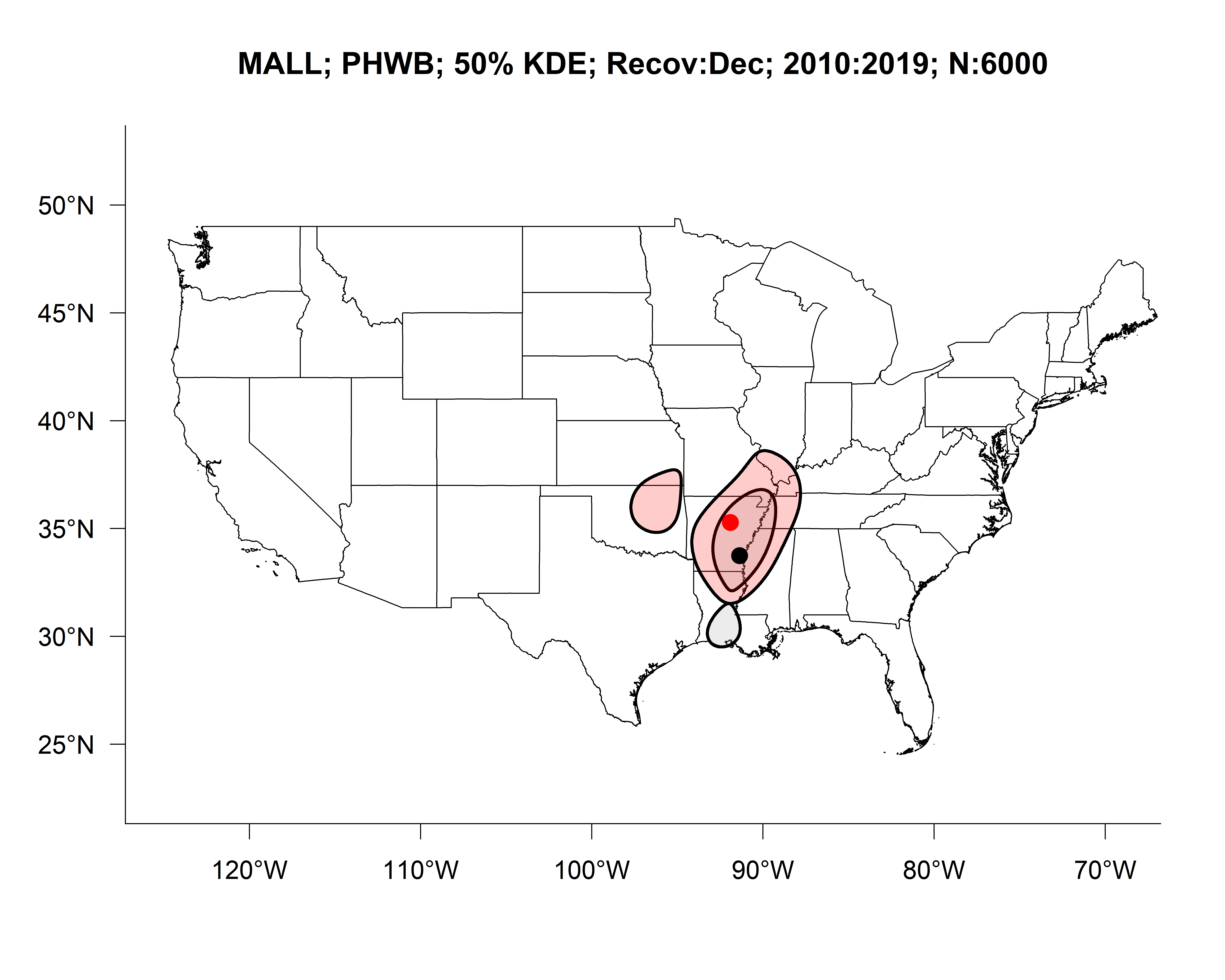

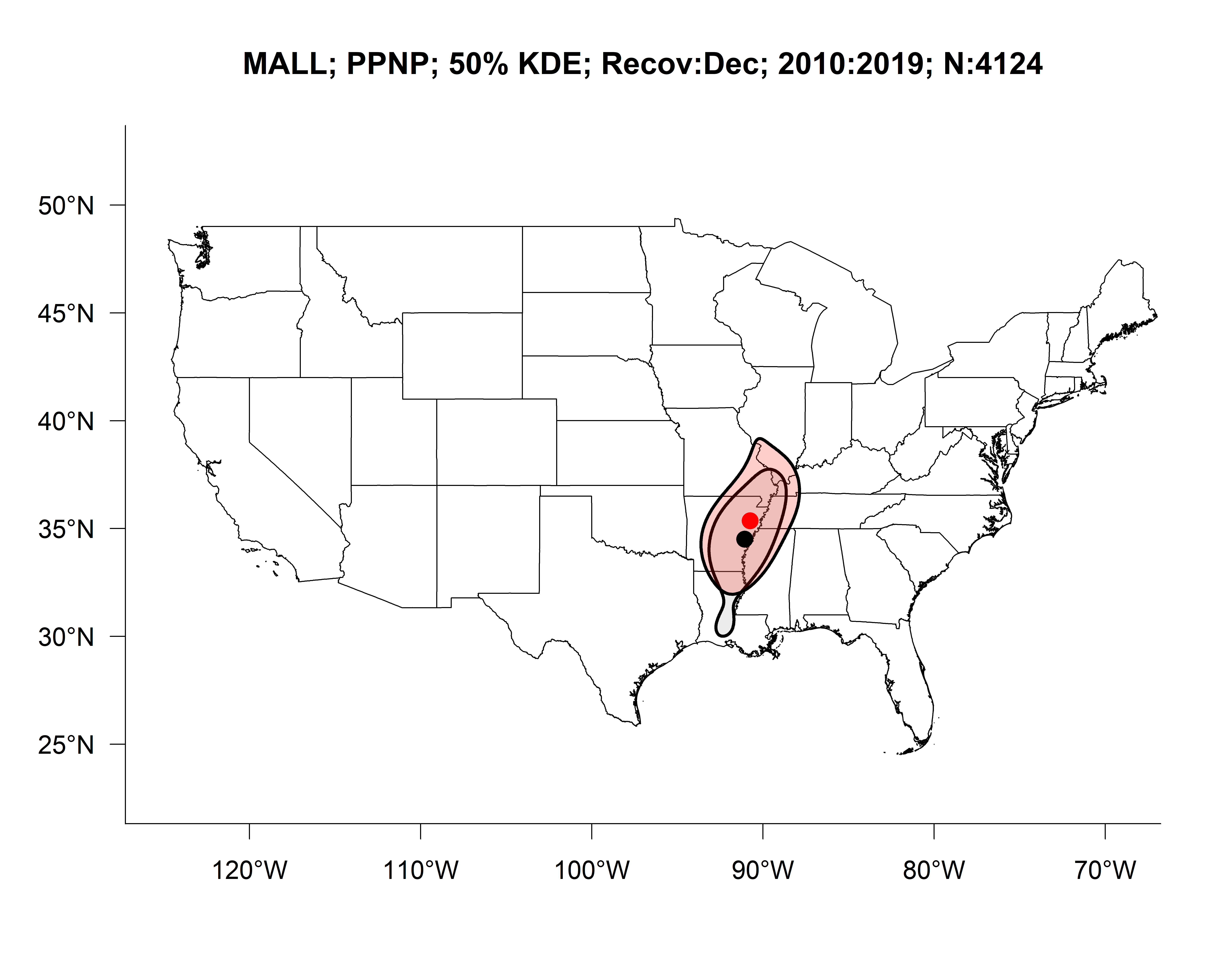

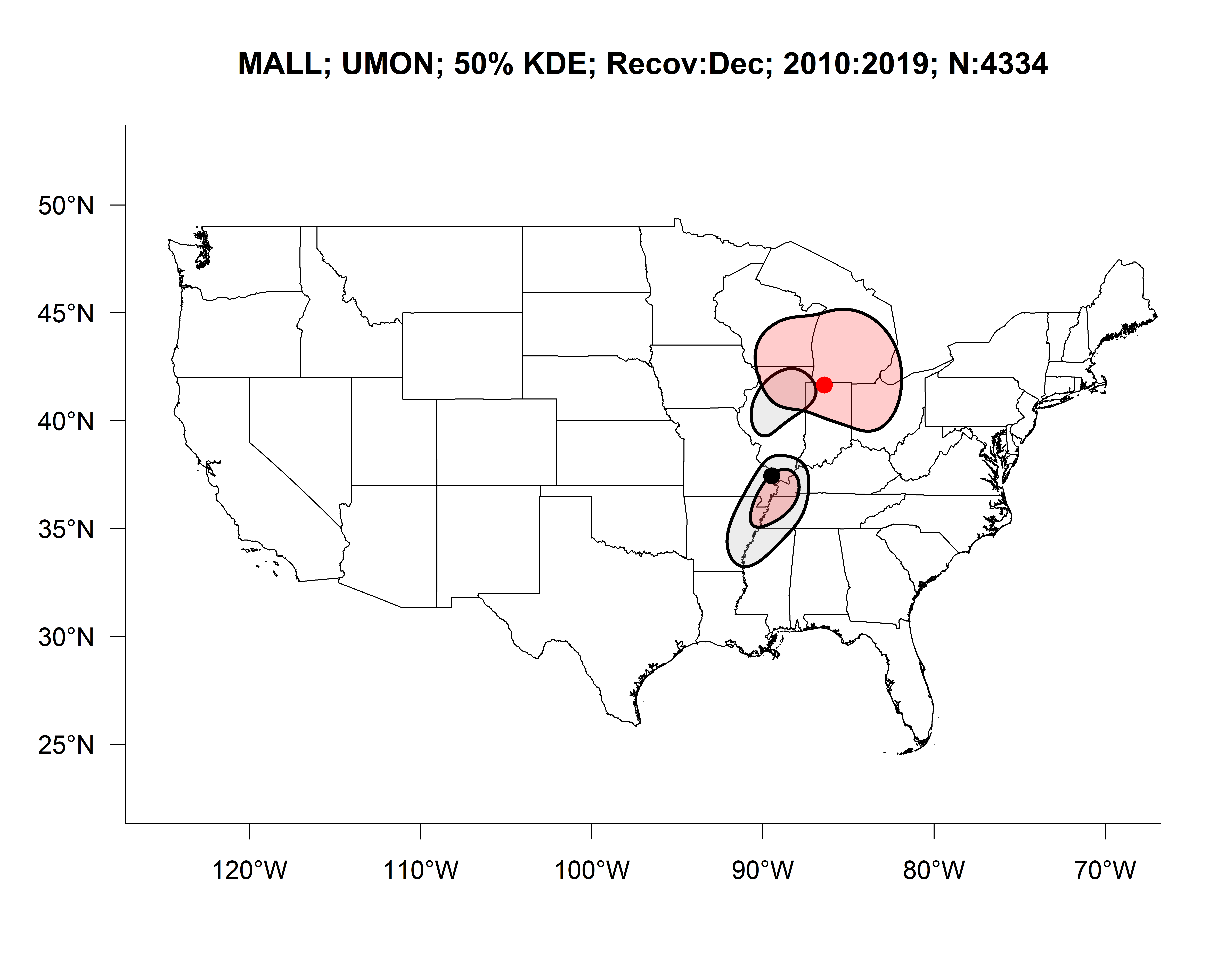

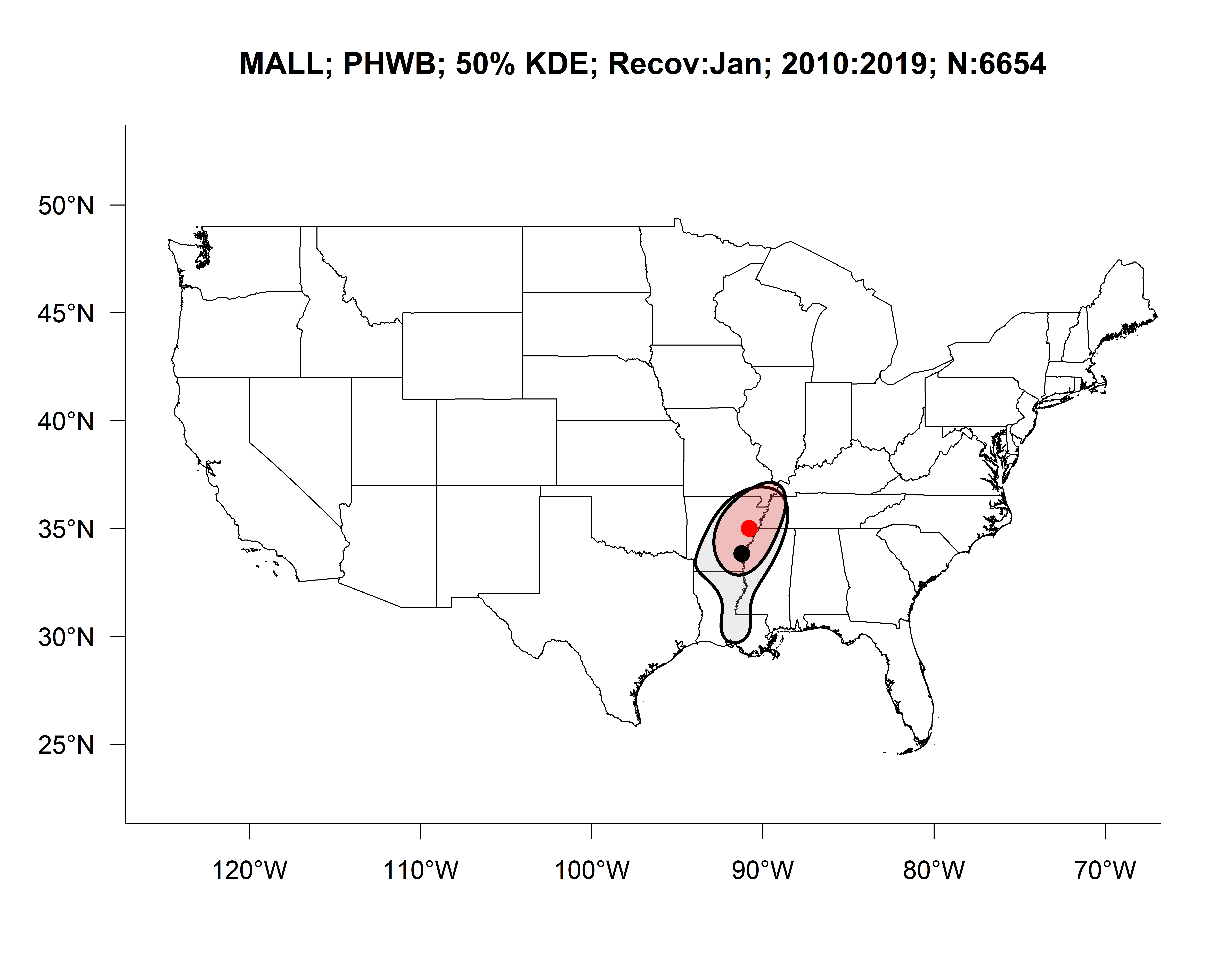

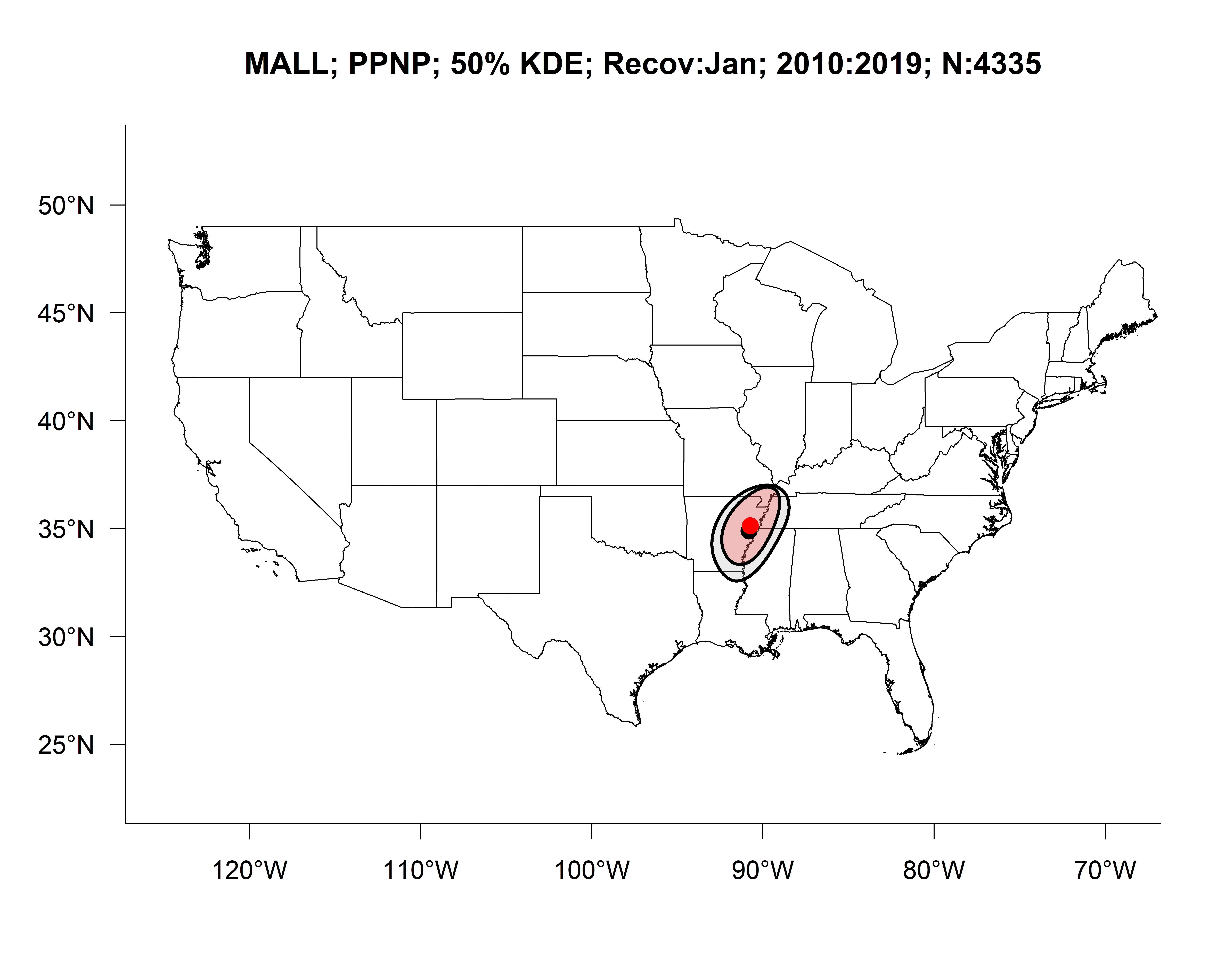

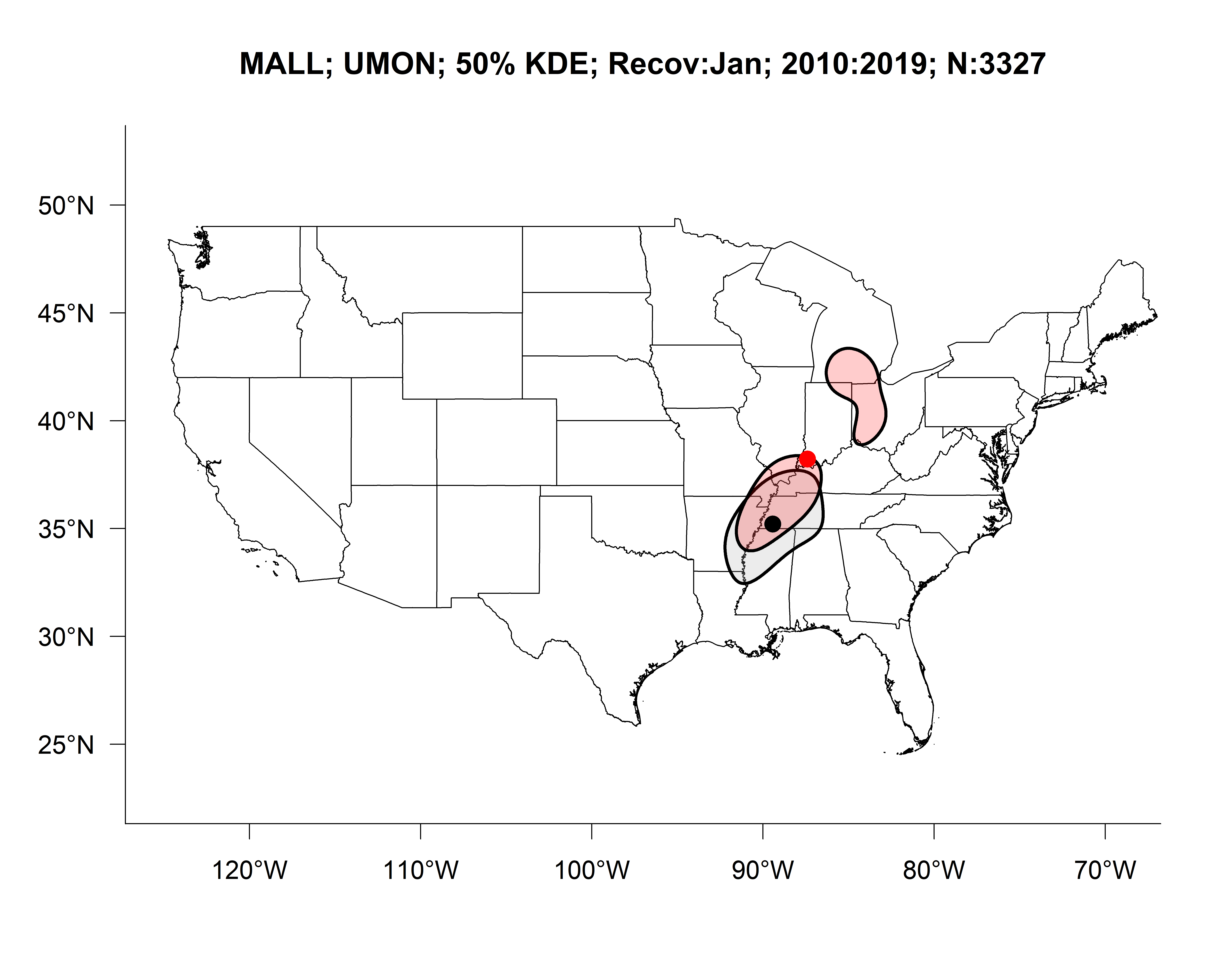


Mallard – 1960–1969 versus 2010–2019 – 95% isopleths


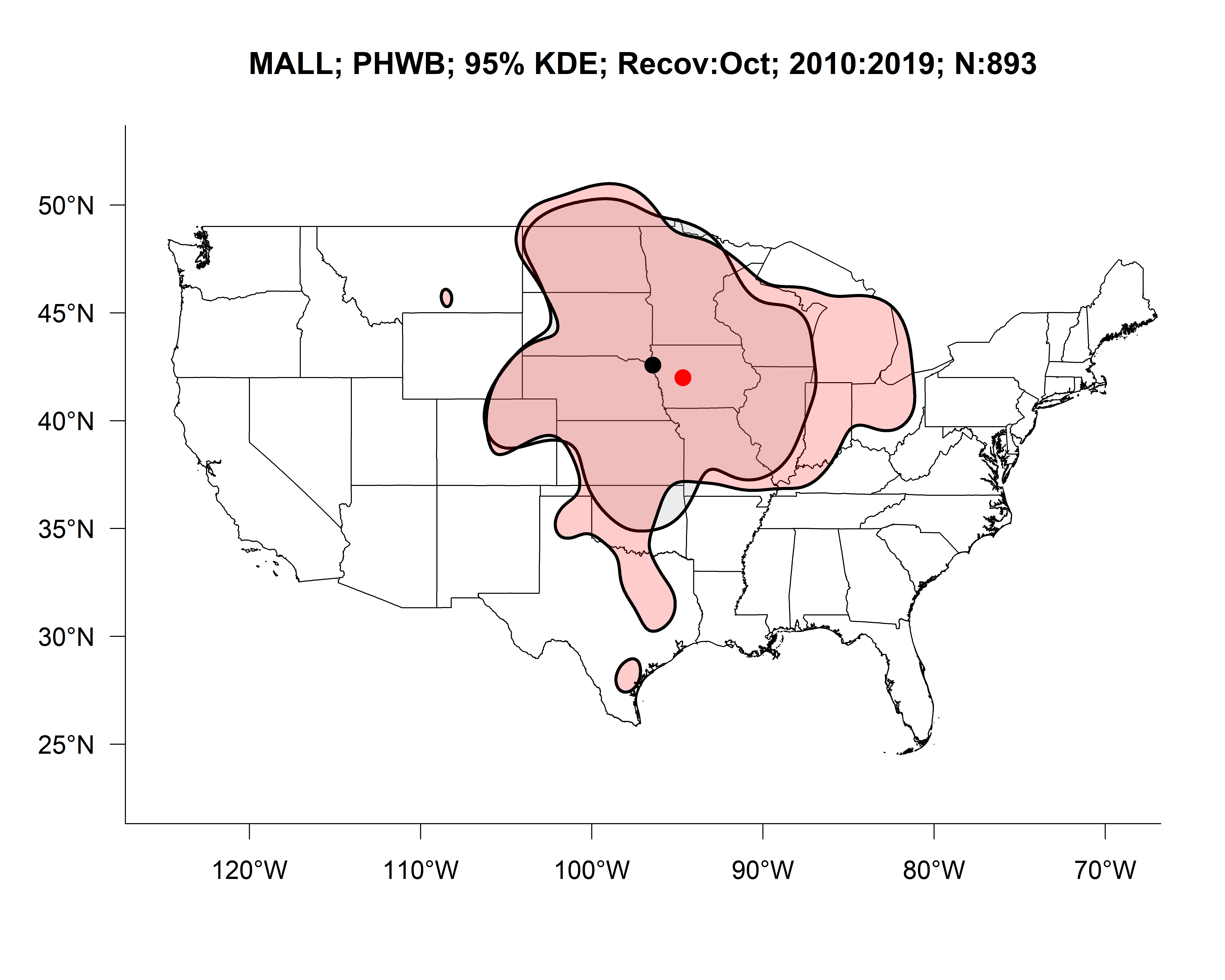

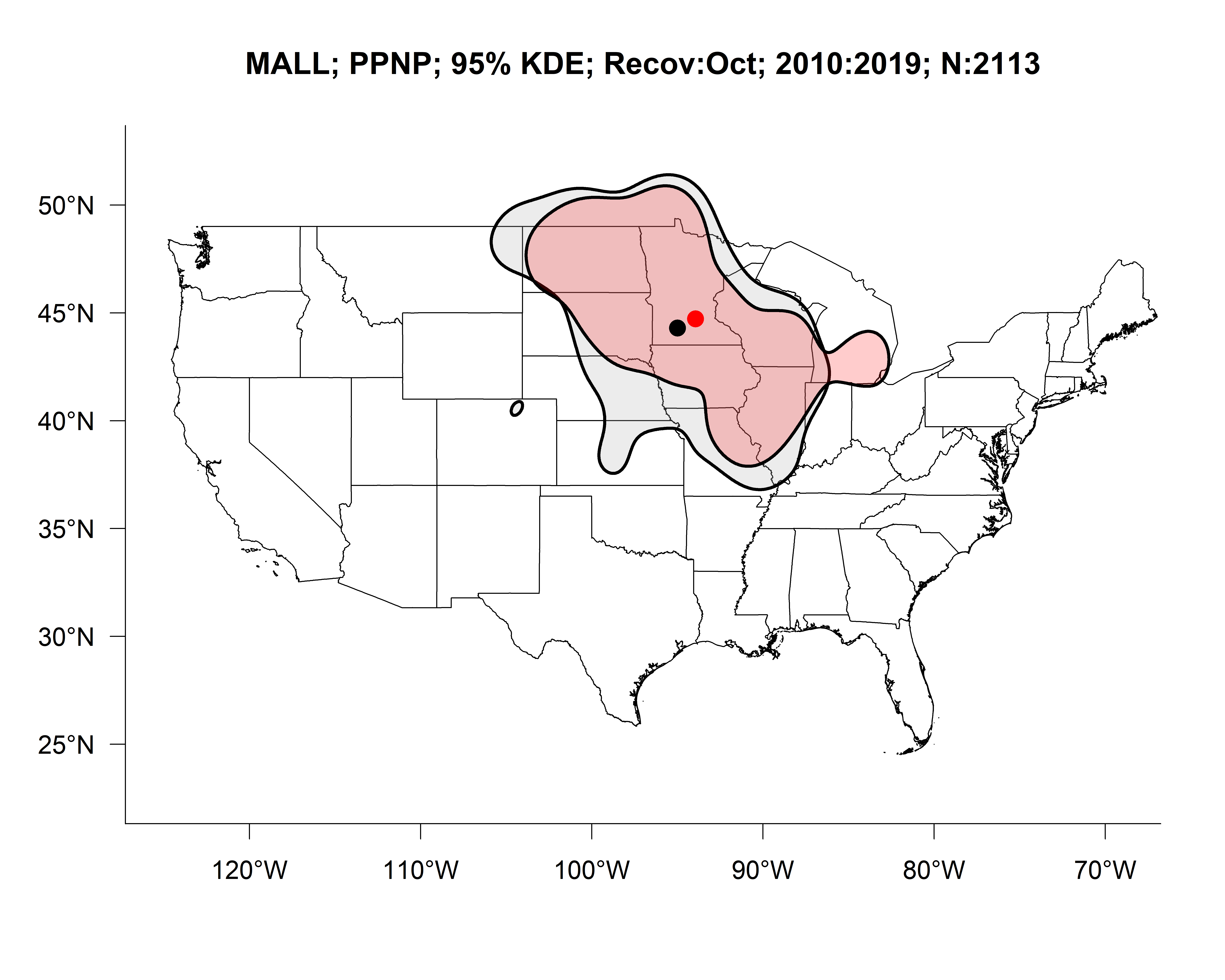

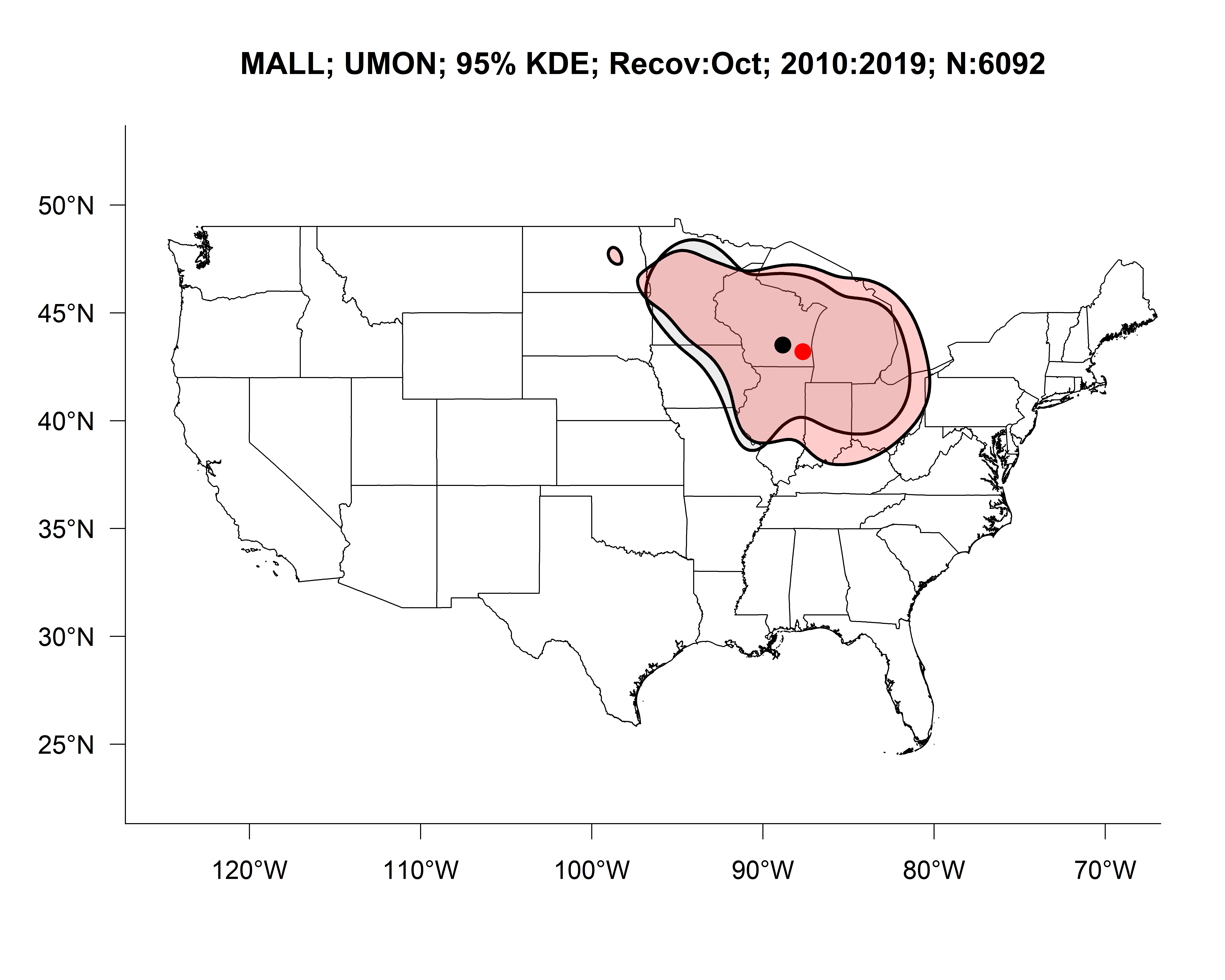


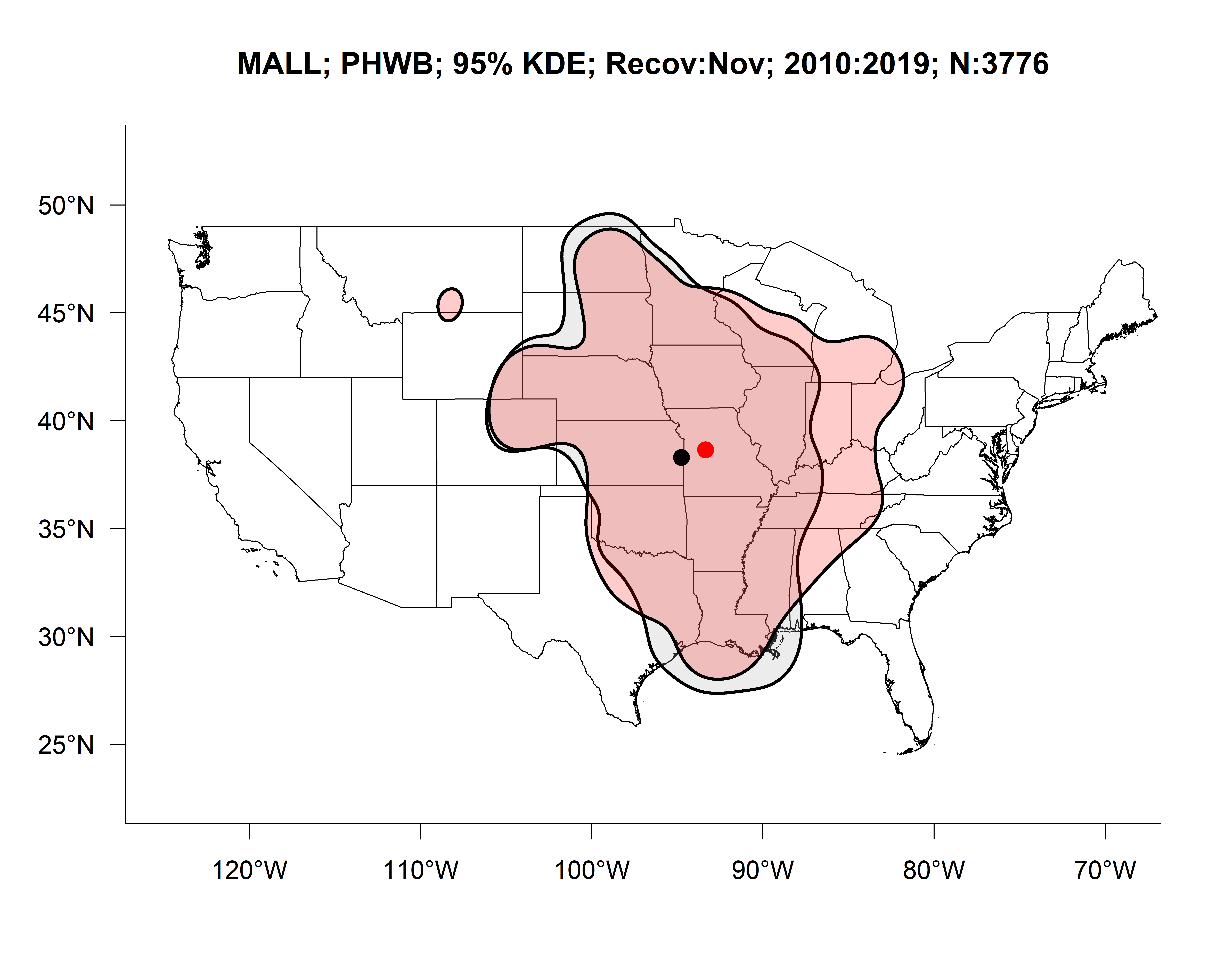

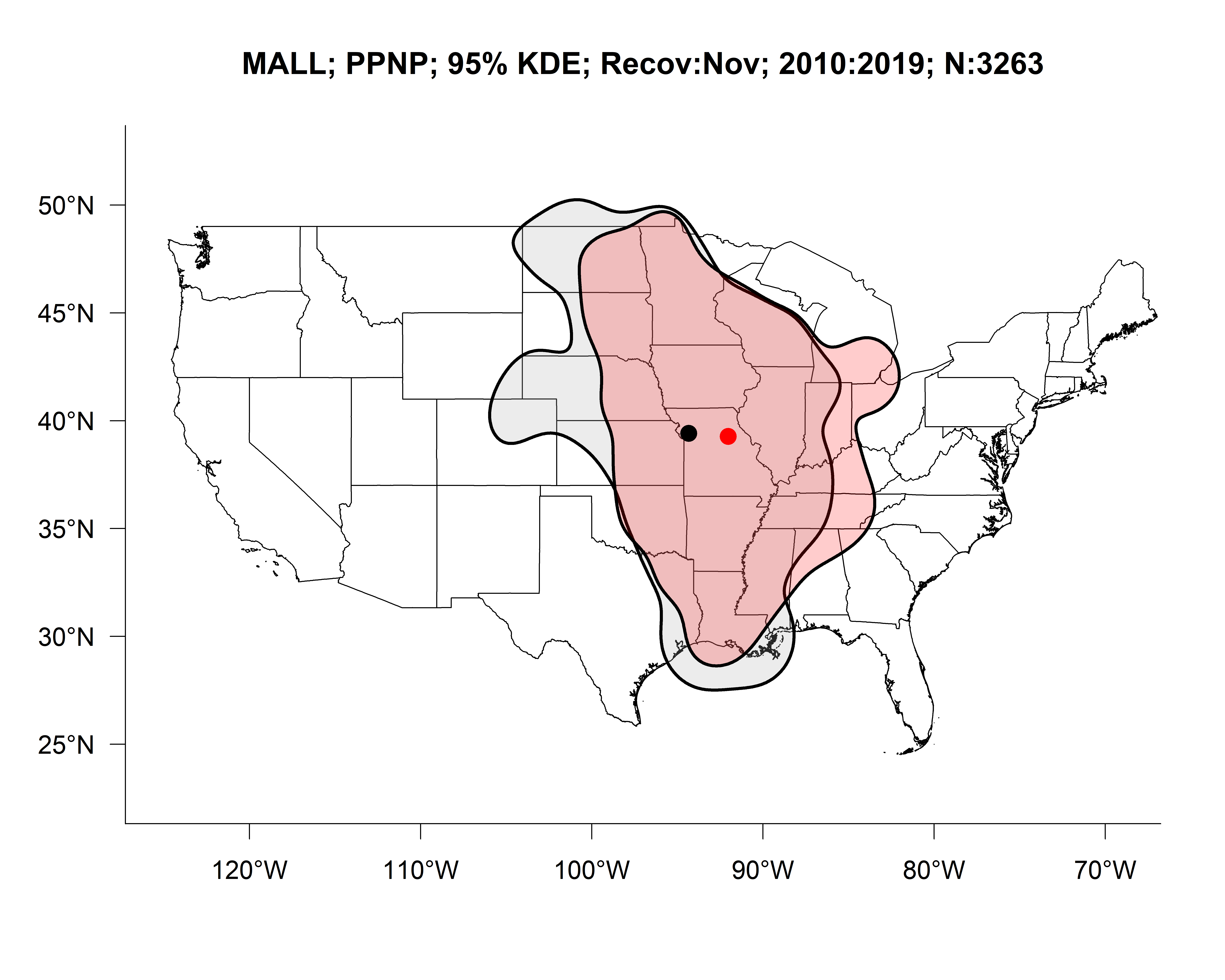

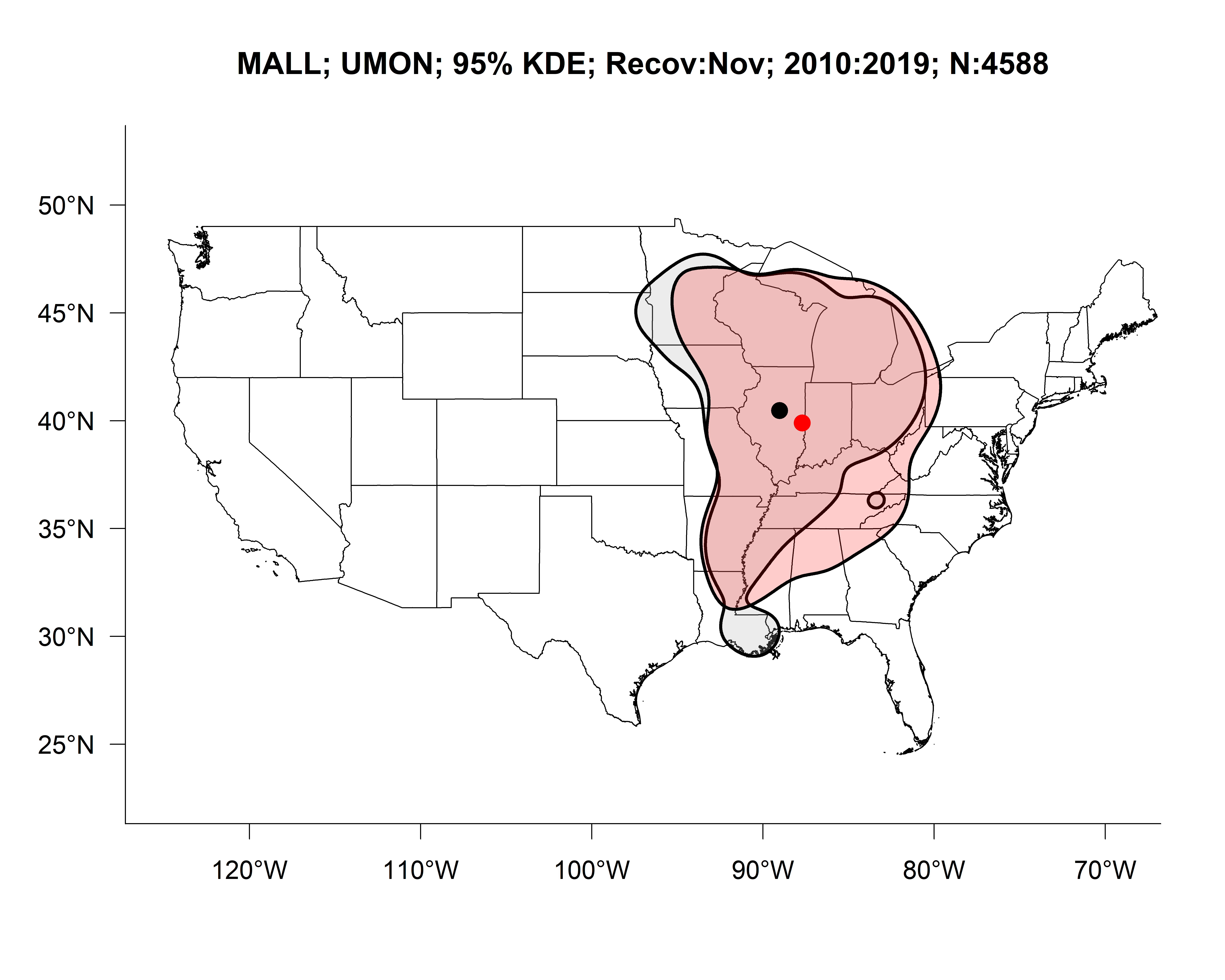


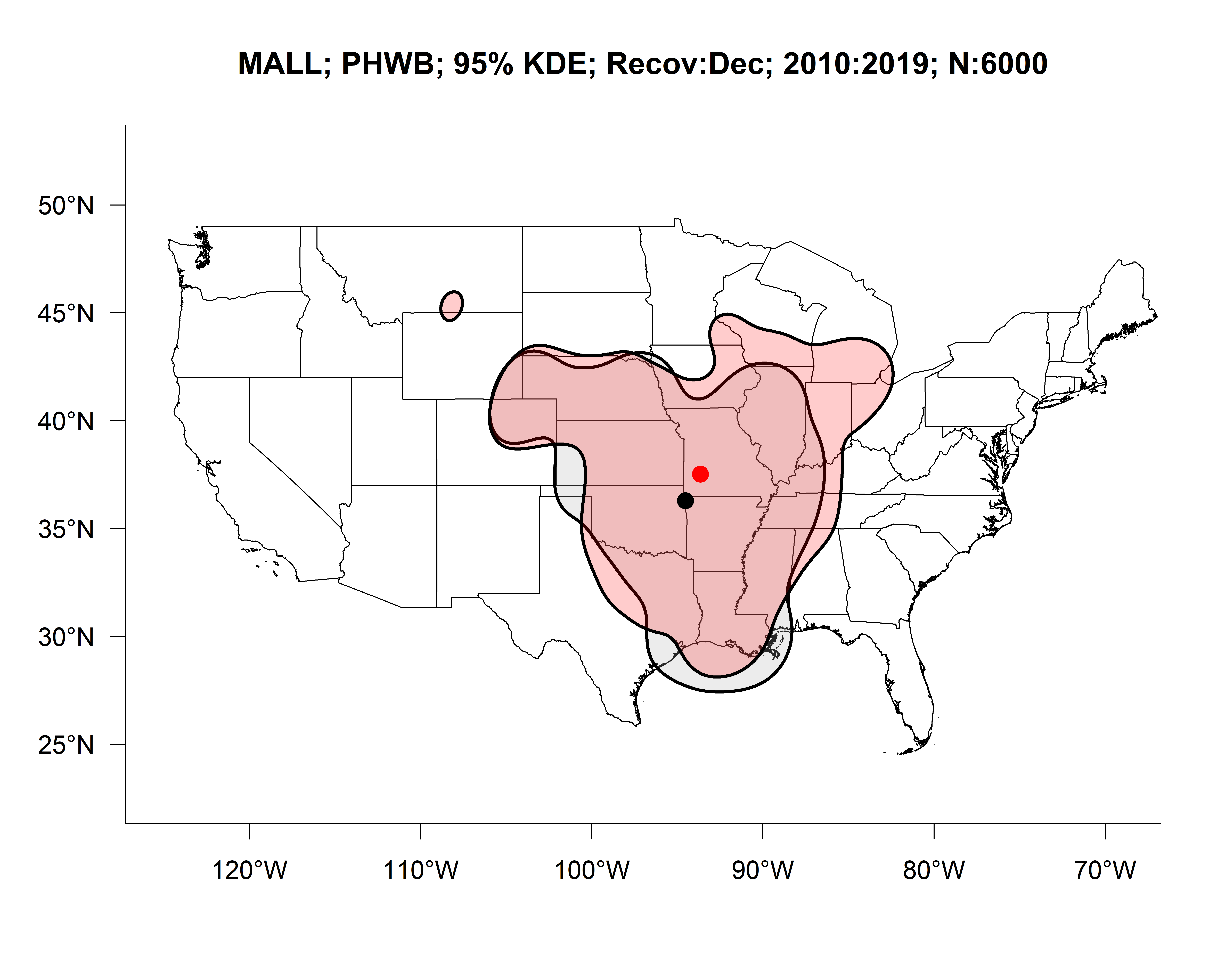

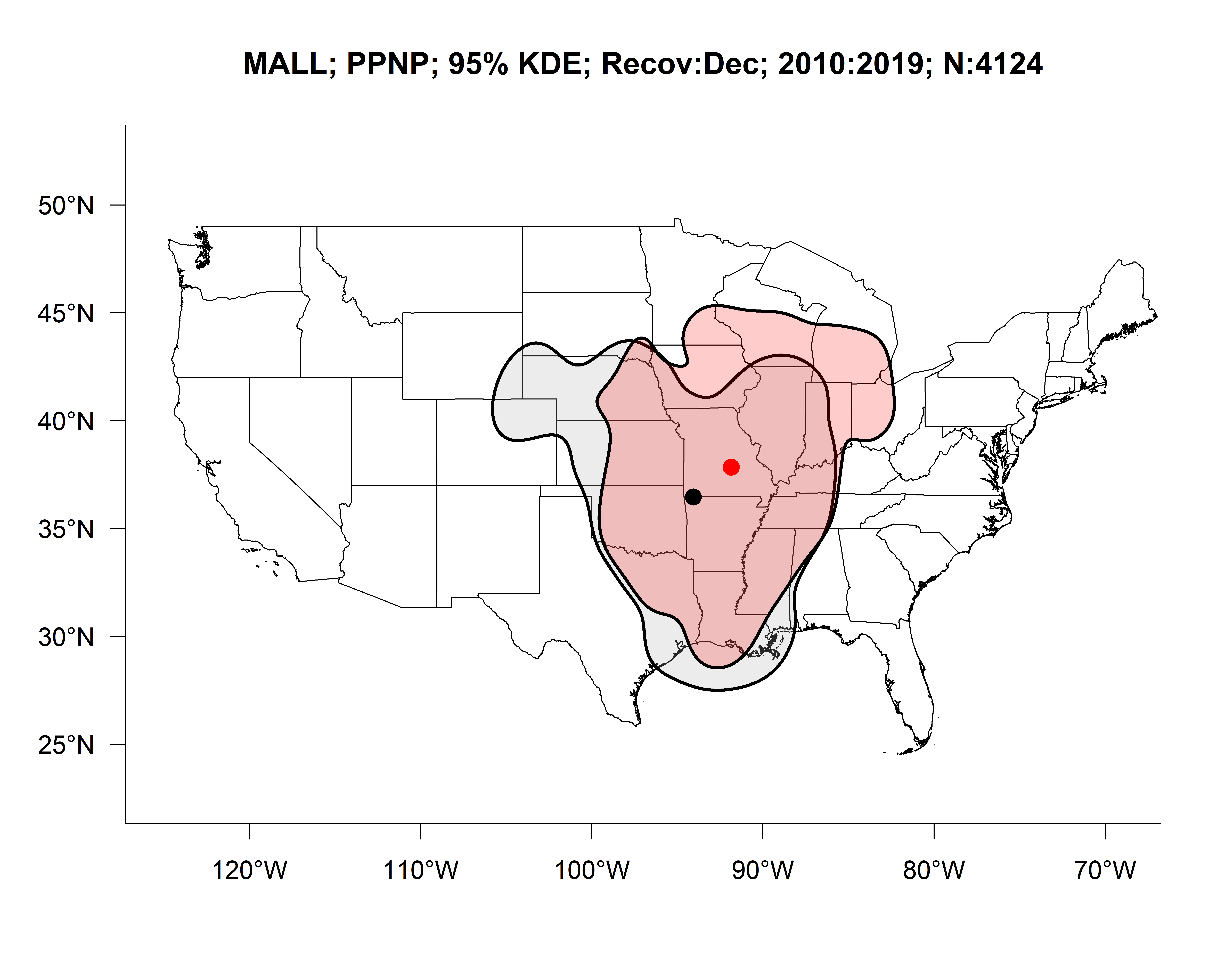

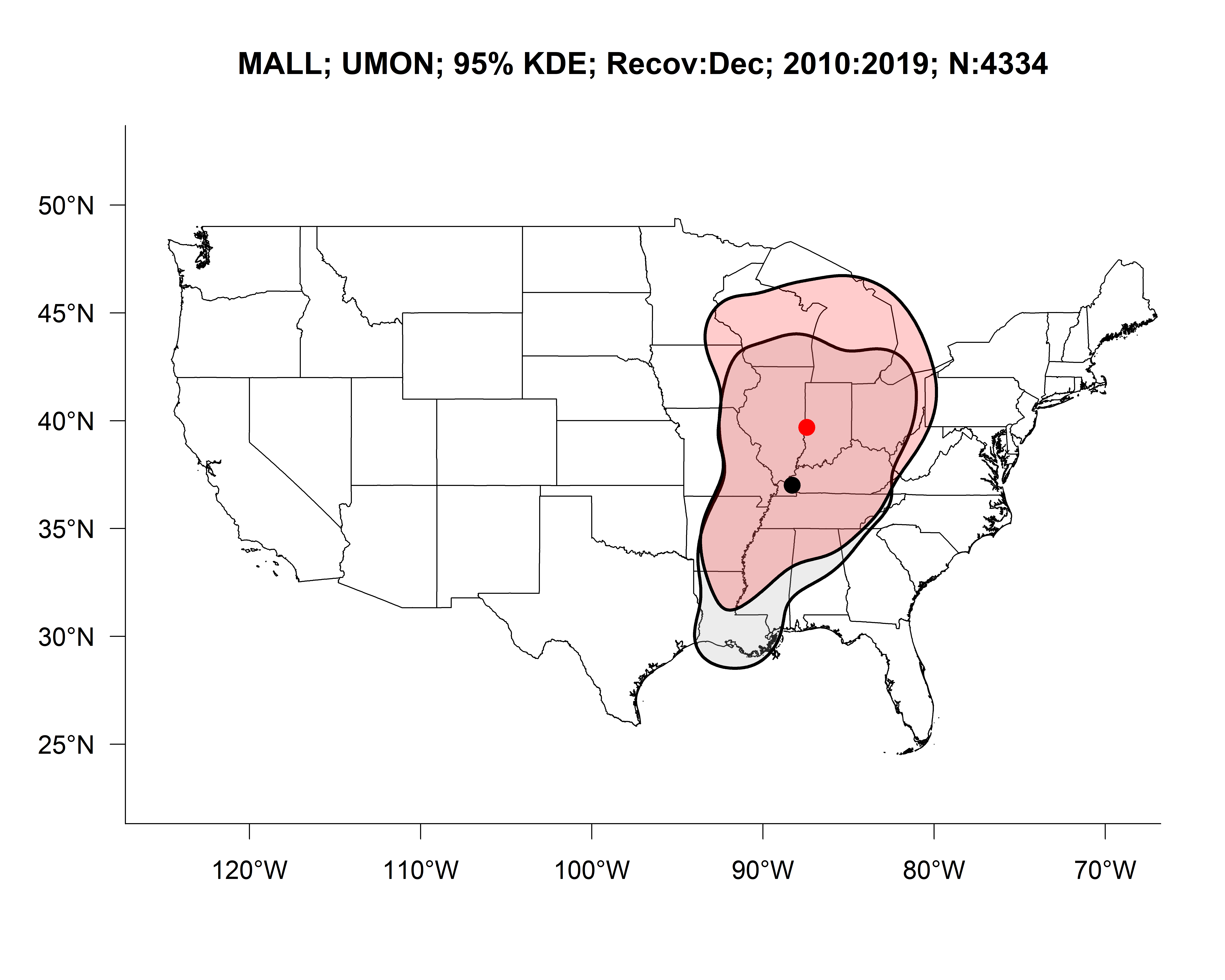


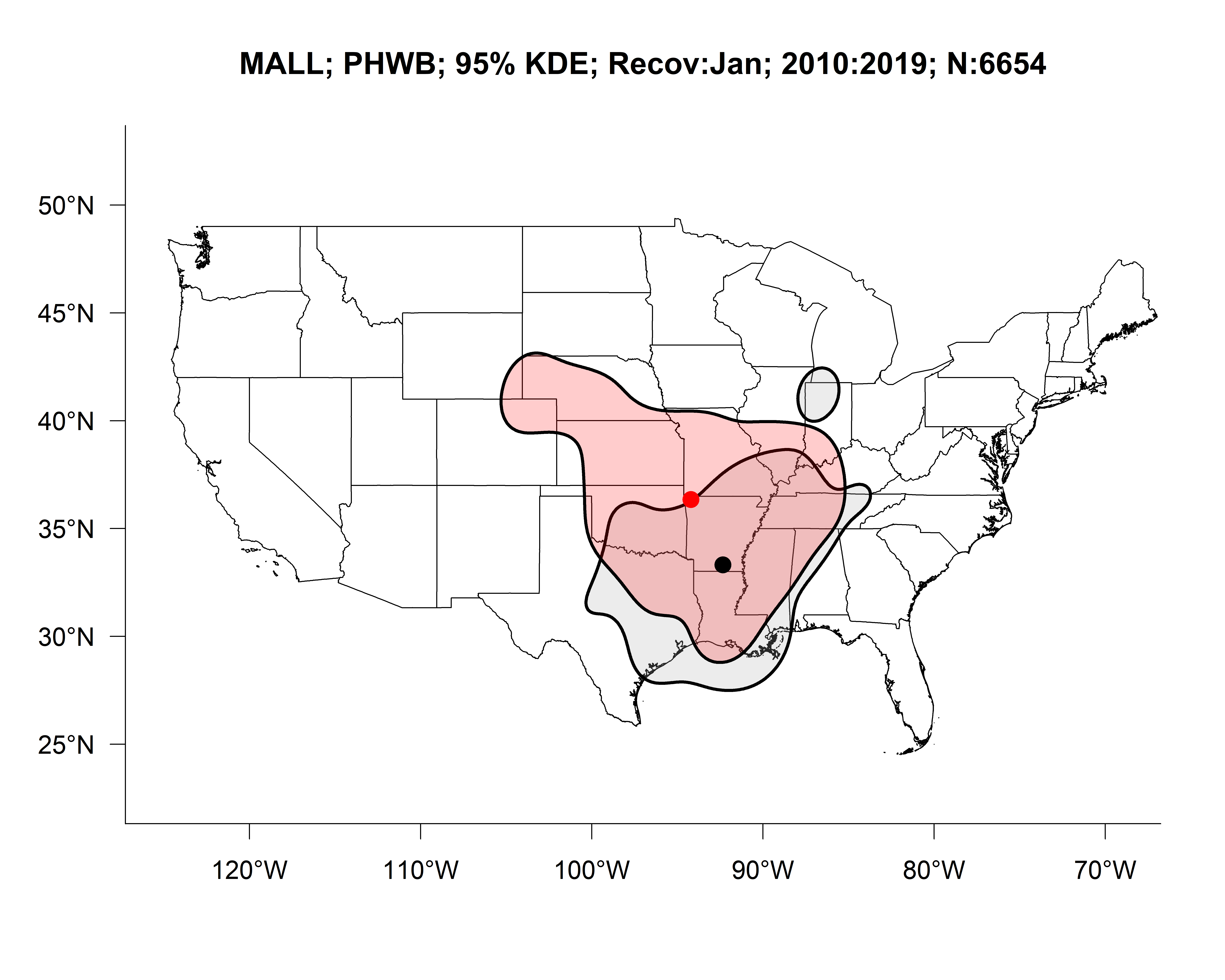

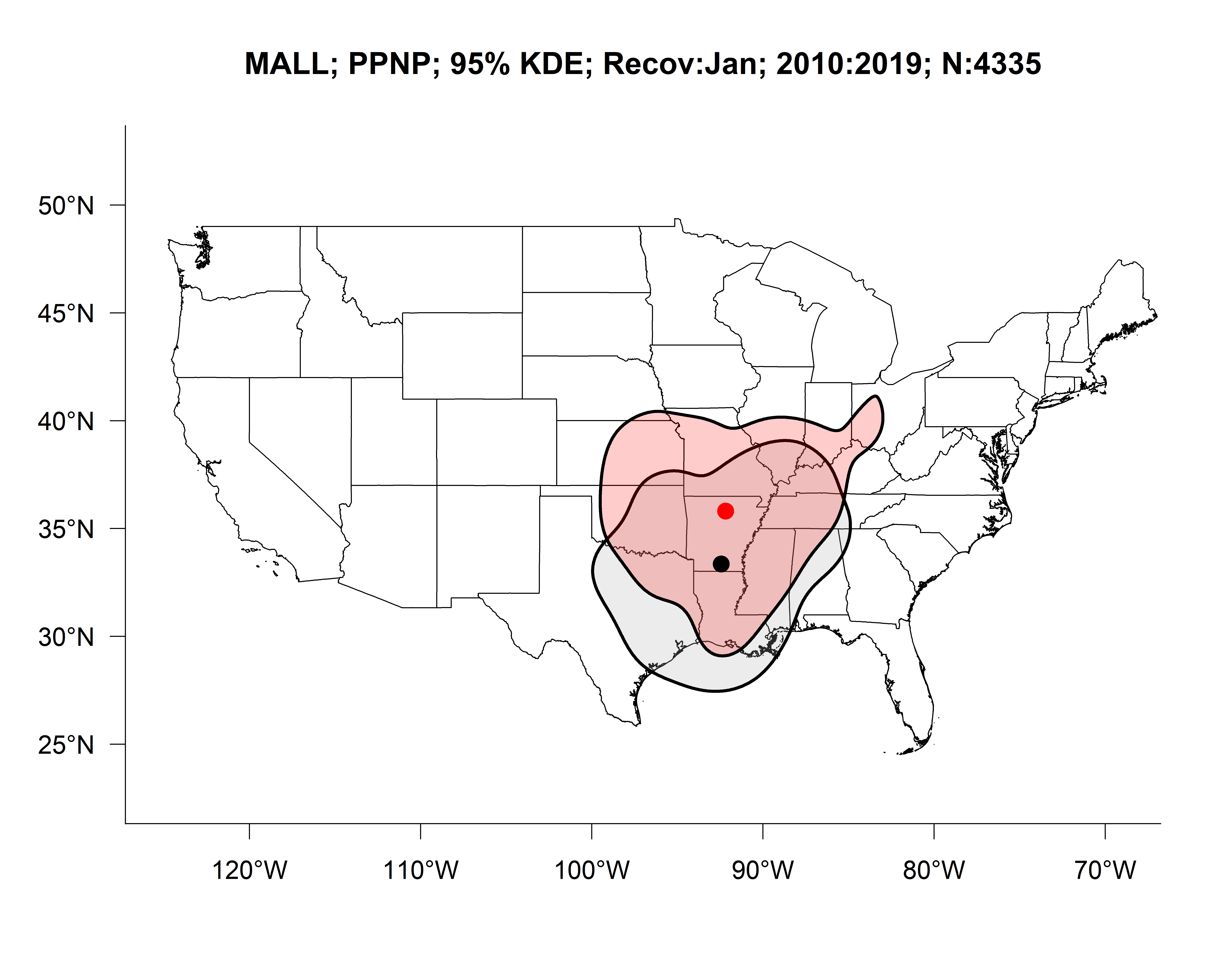

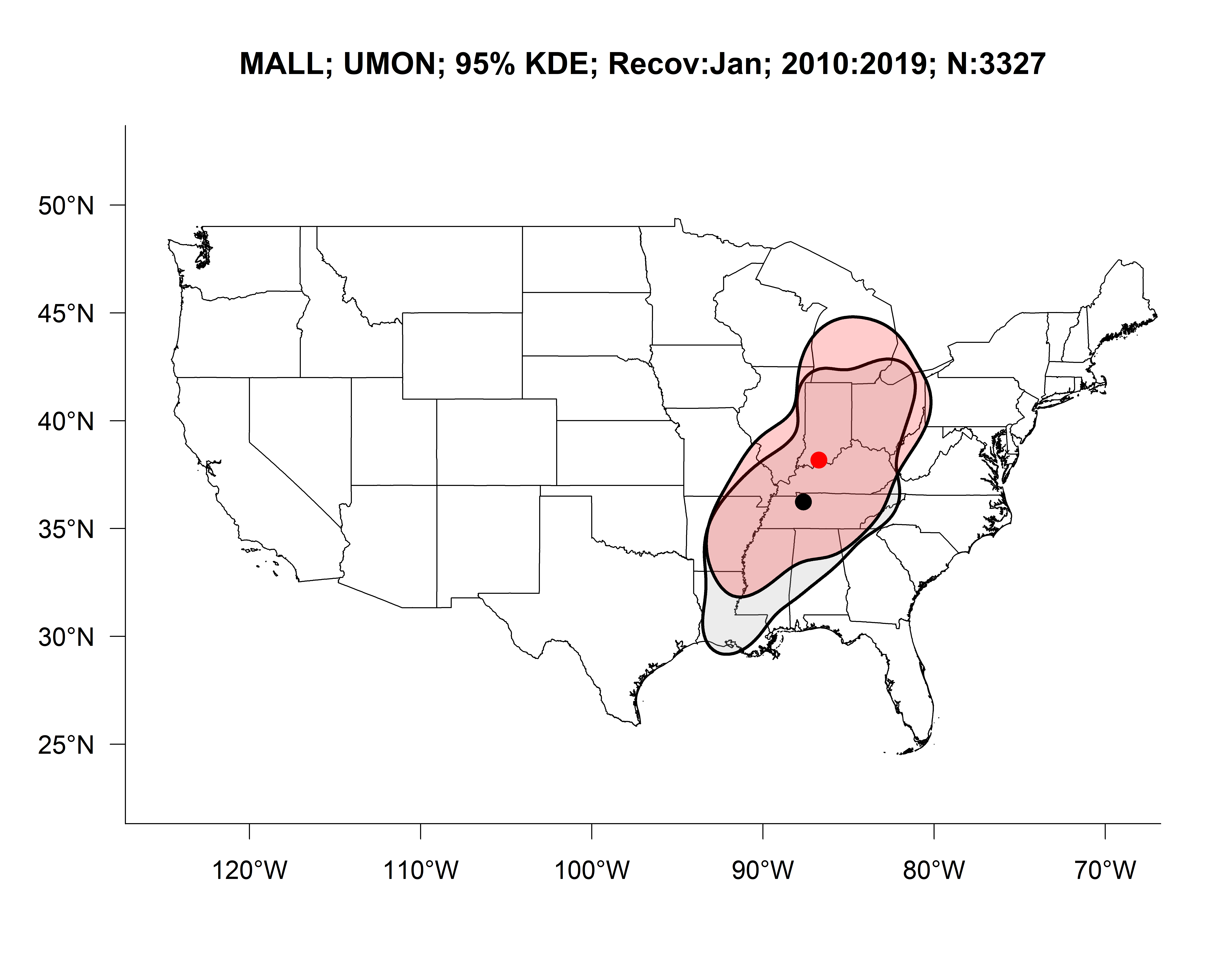


Northern pintail – 1960–1969 versus 2010–2019 – 50% isopleths


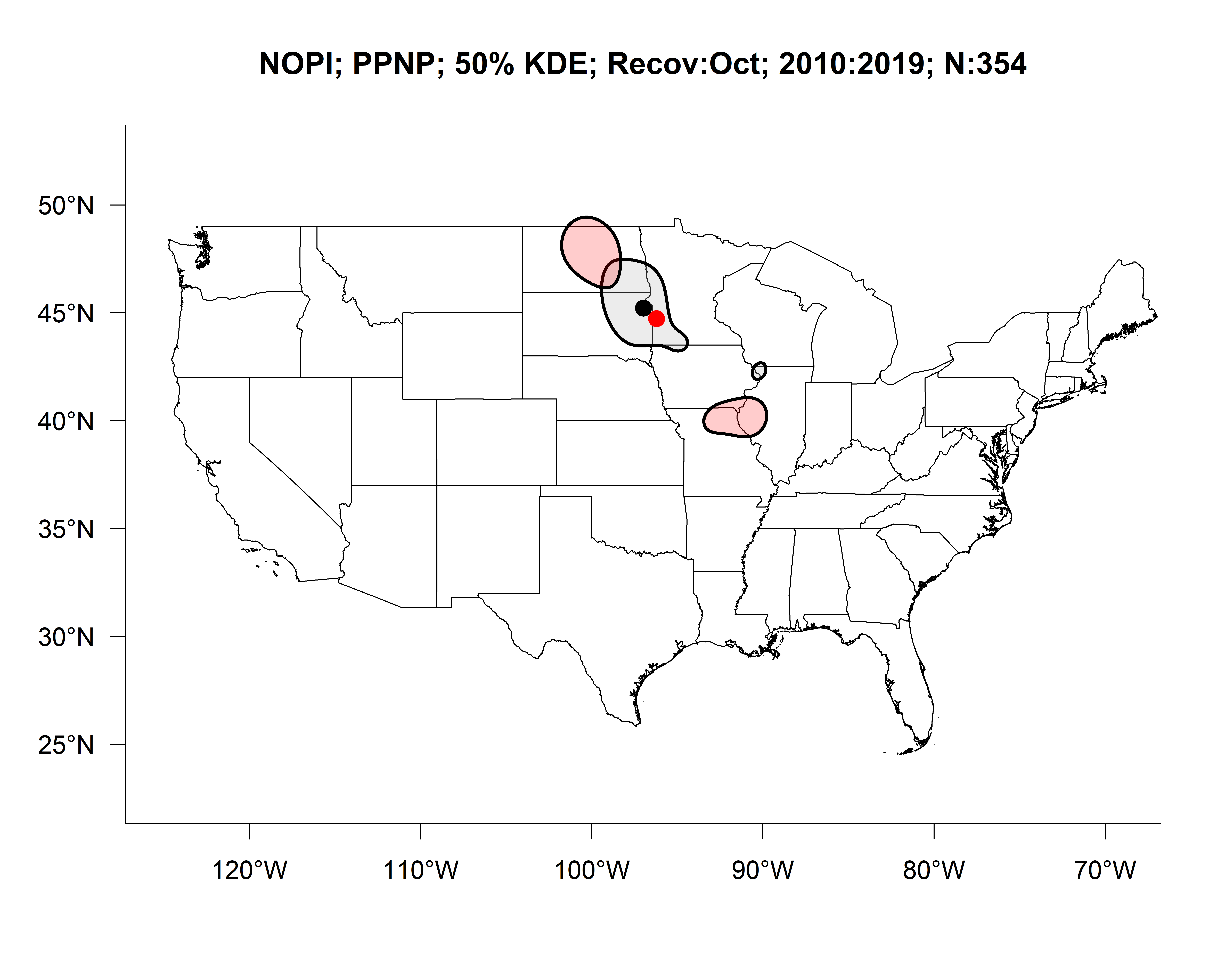

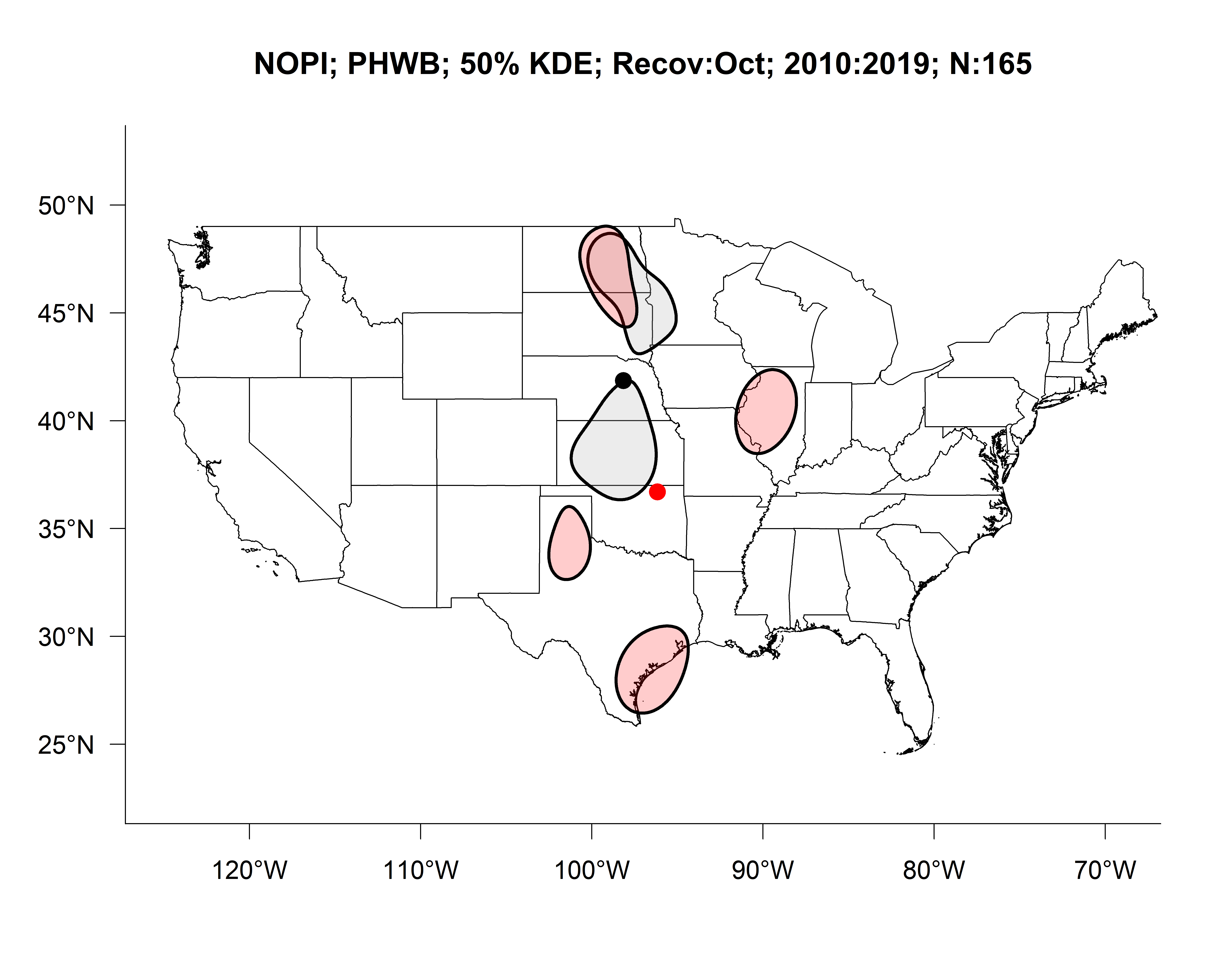

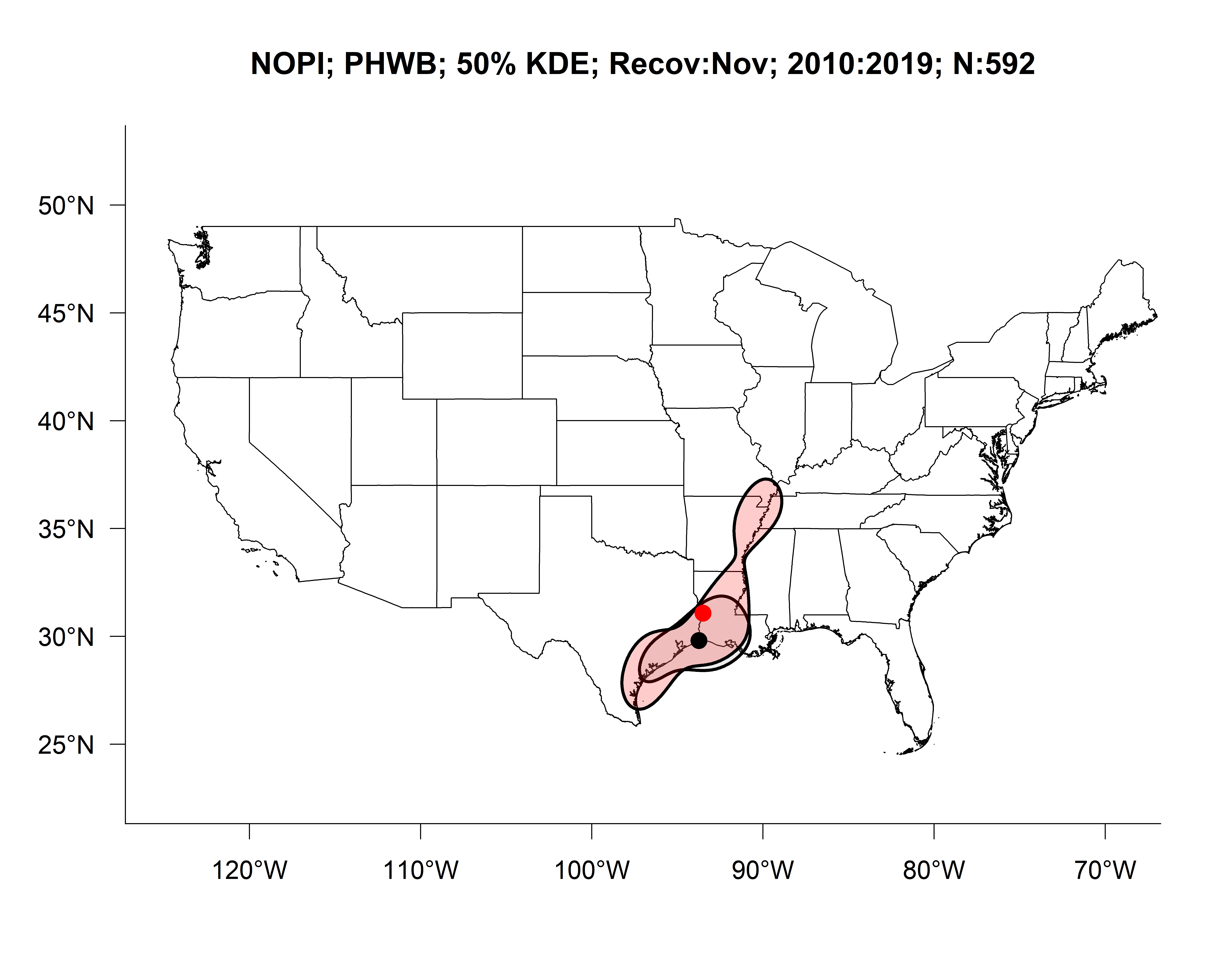

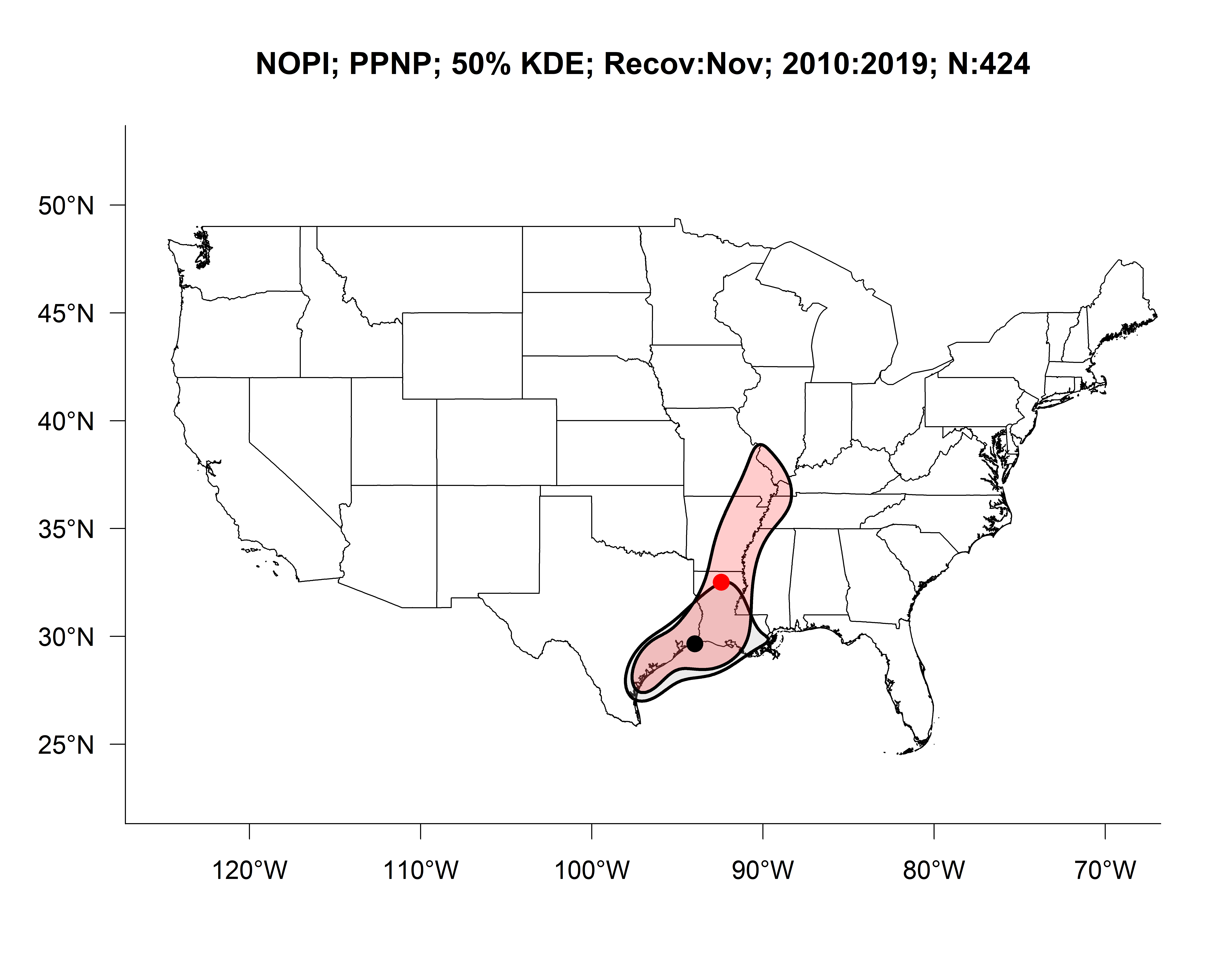

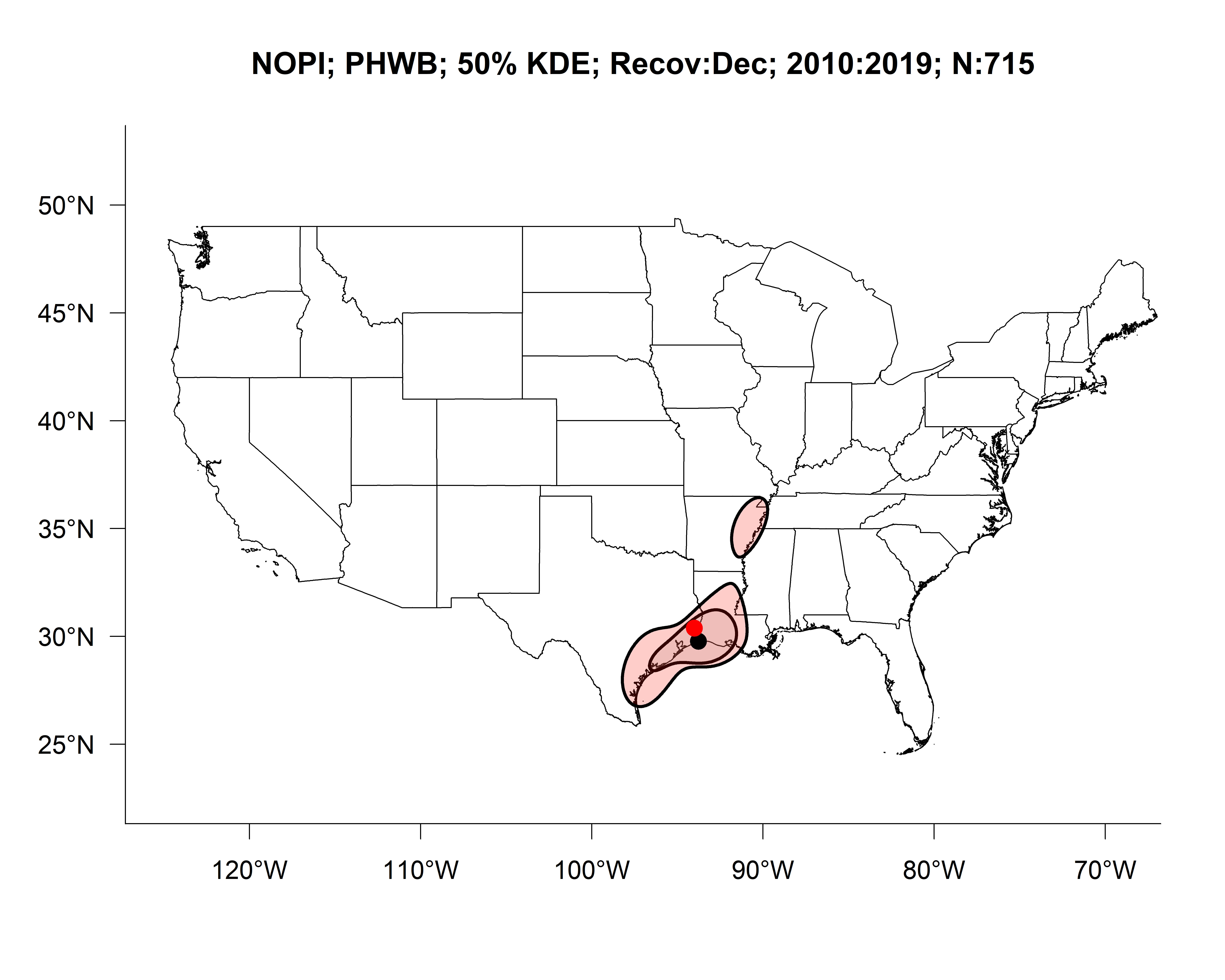

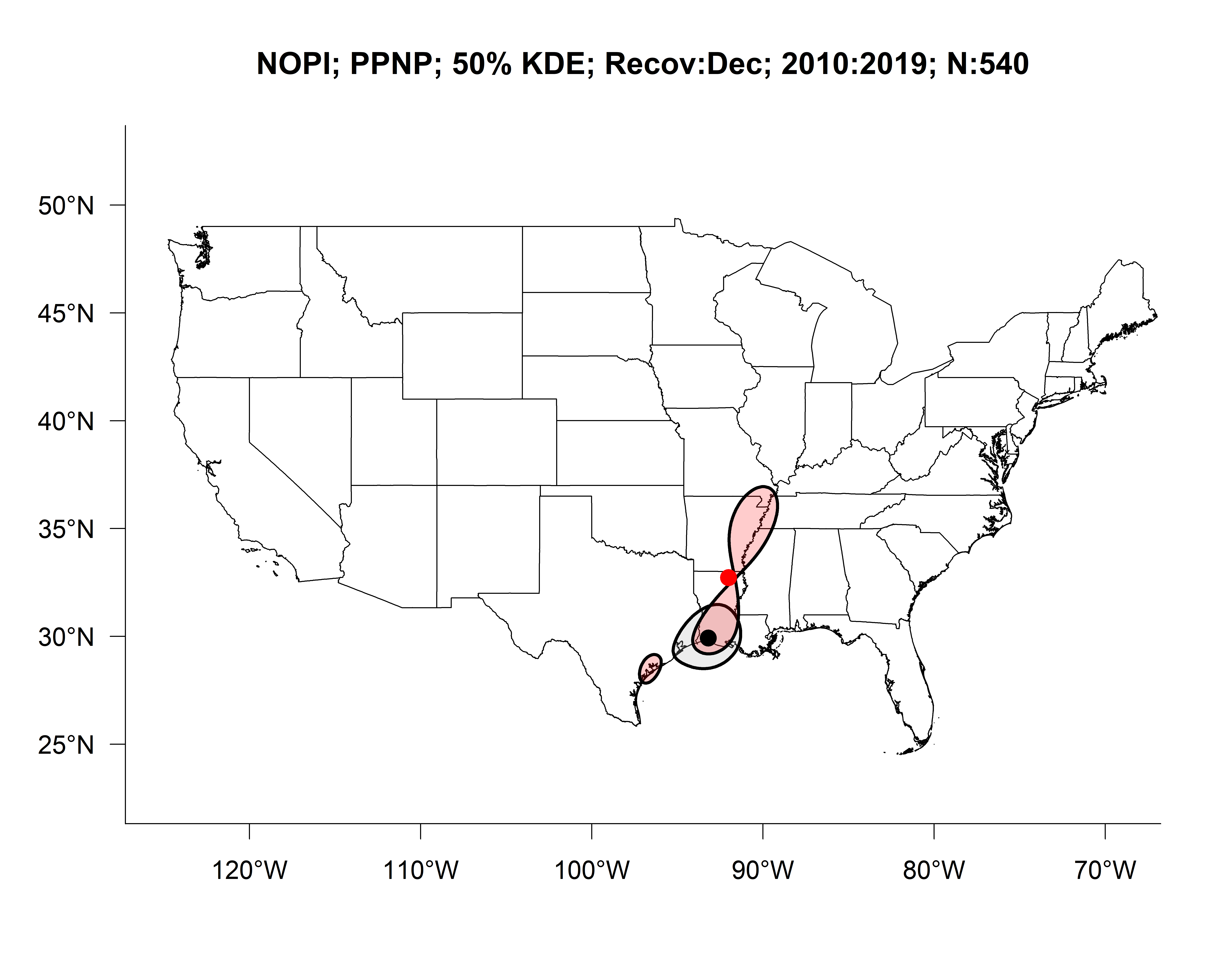

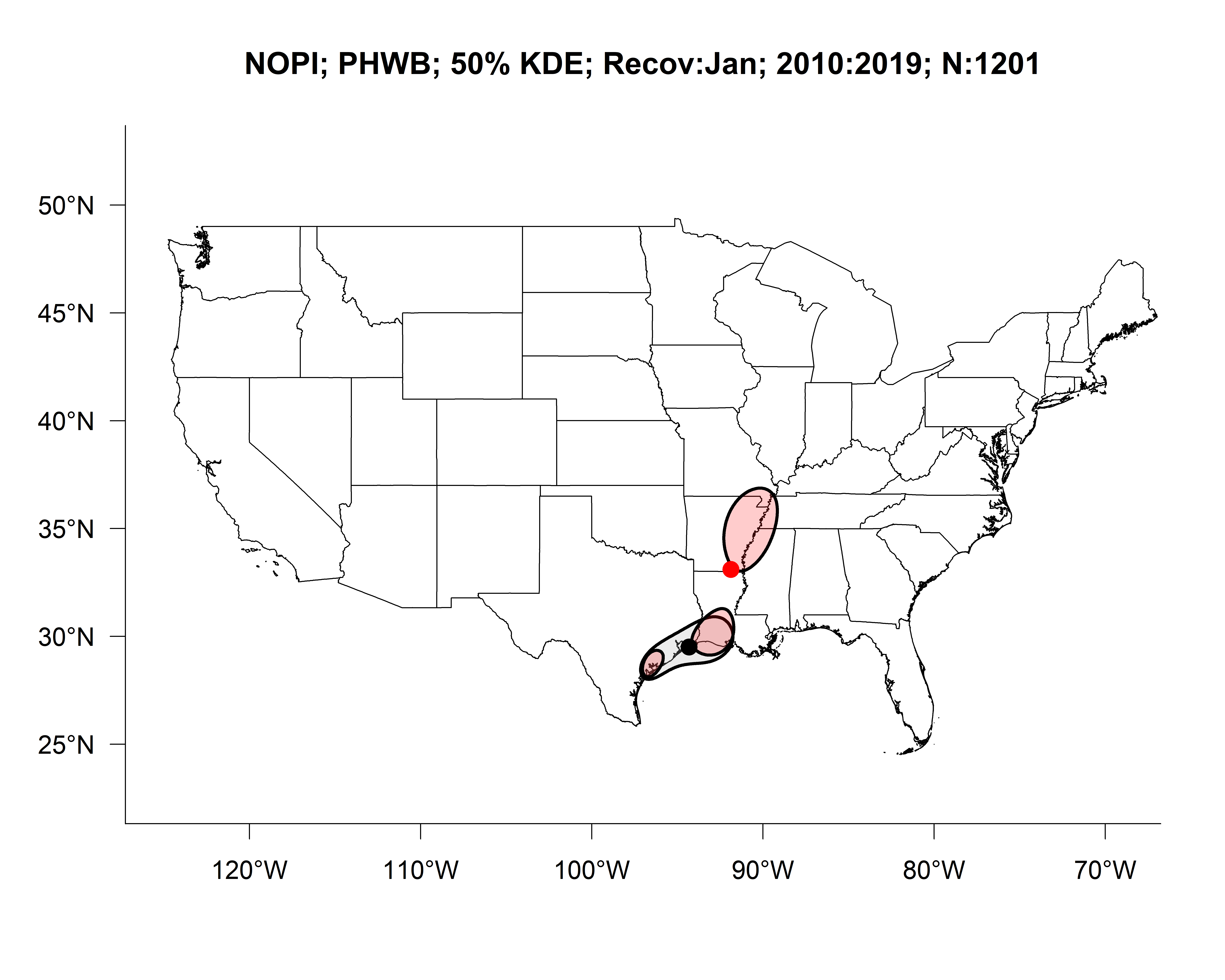

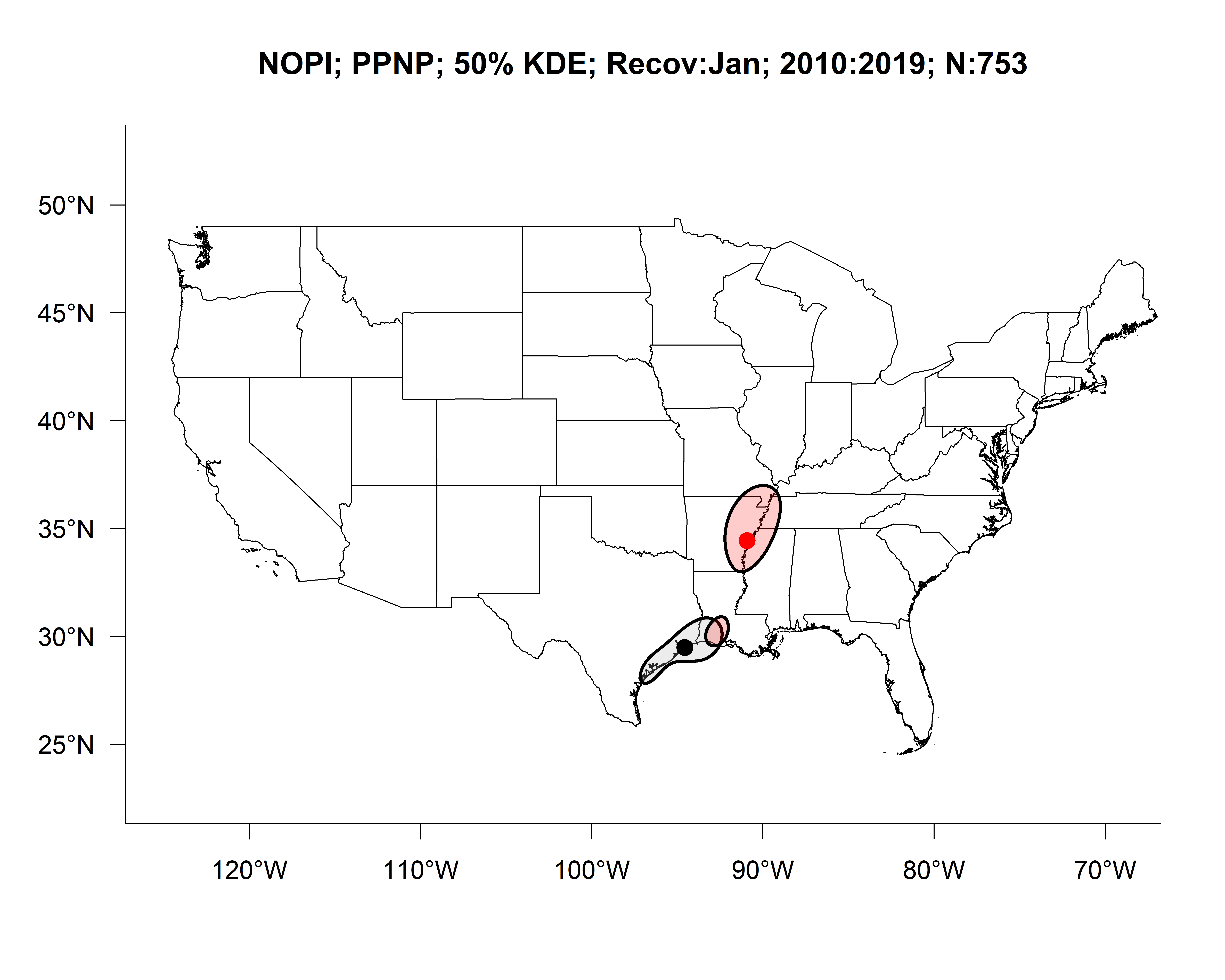


Northern pintail – 1960–1969 versus 2010–2019 – 95% isopleths


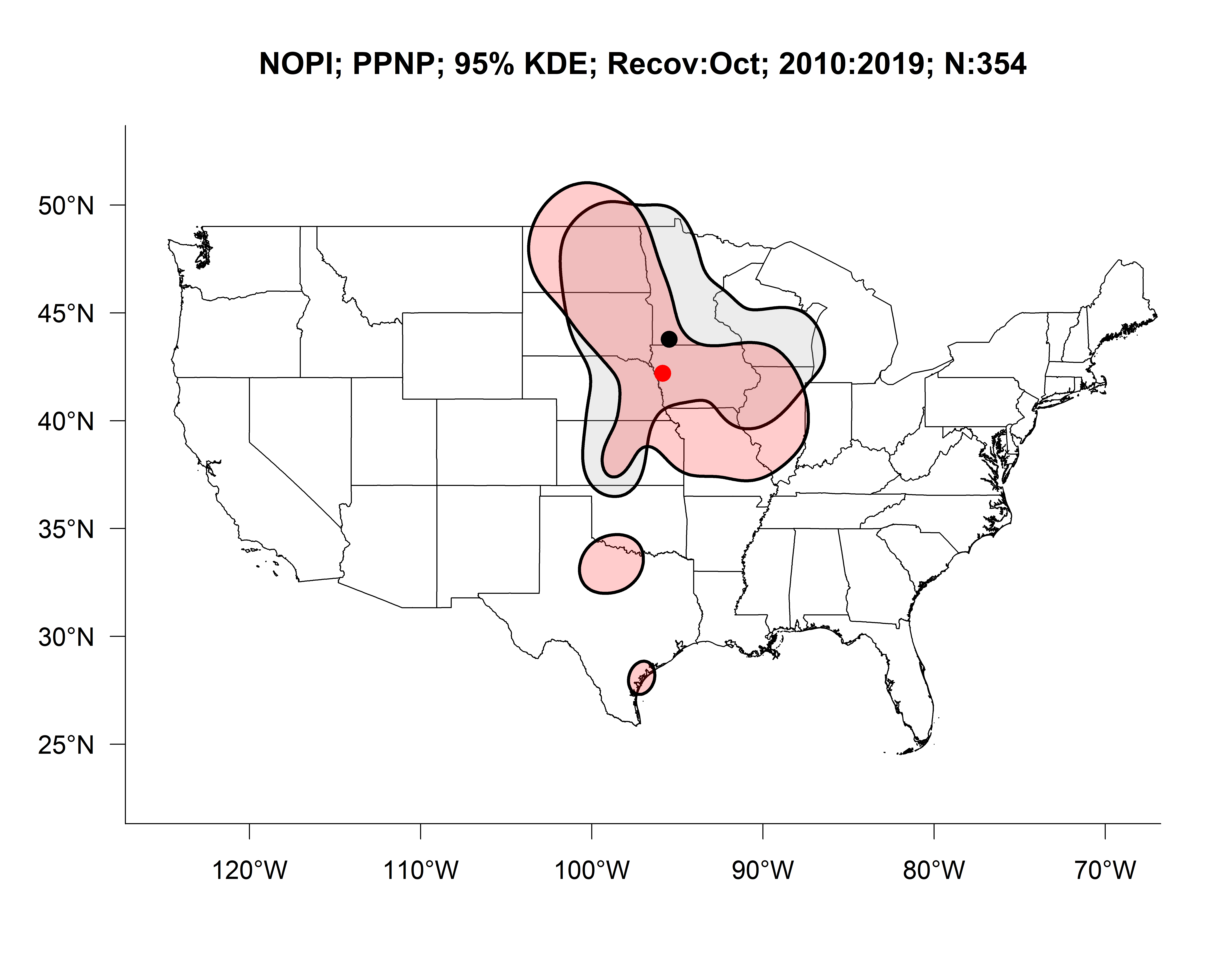

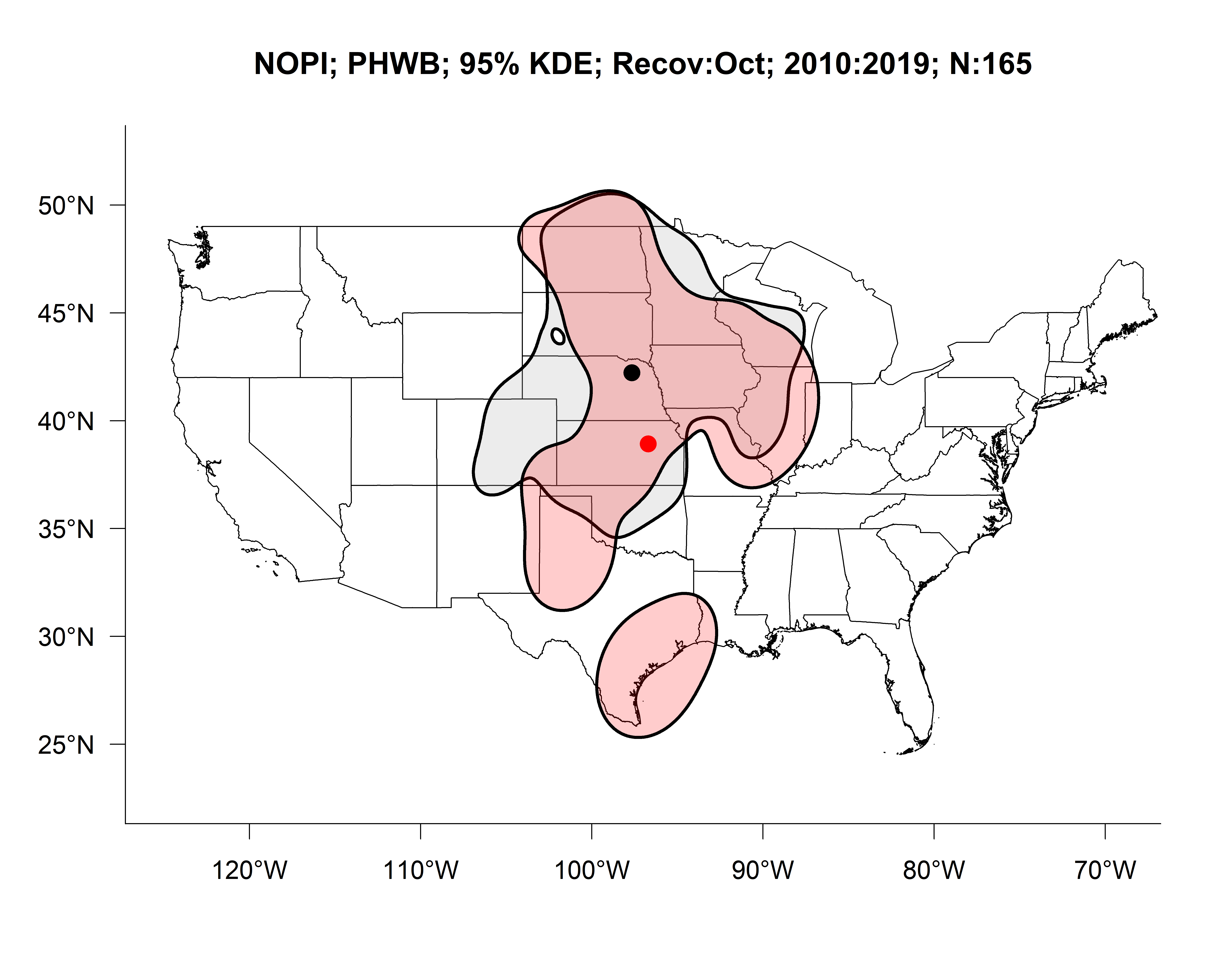

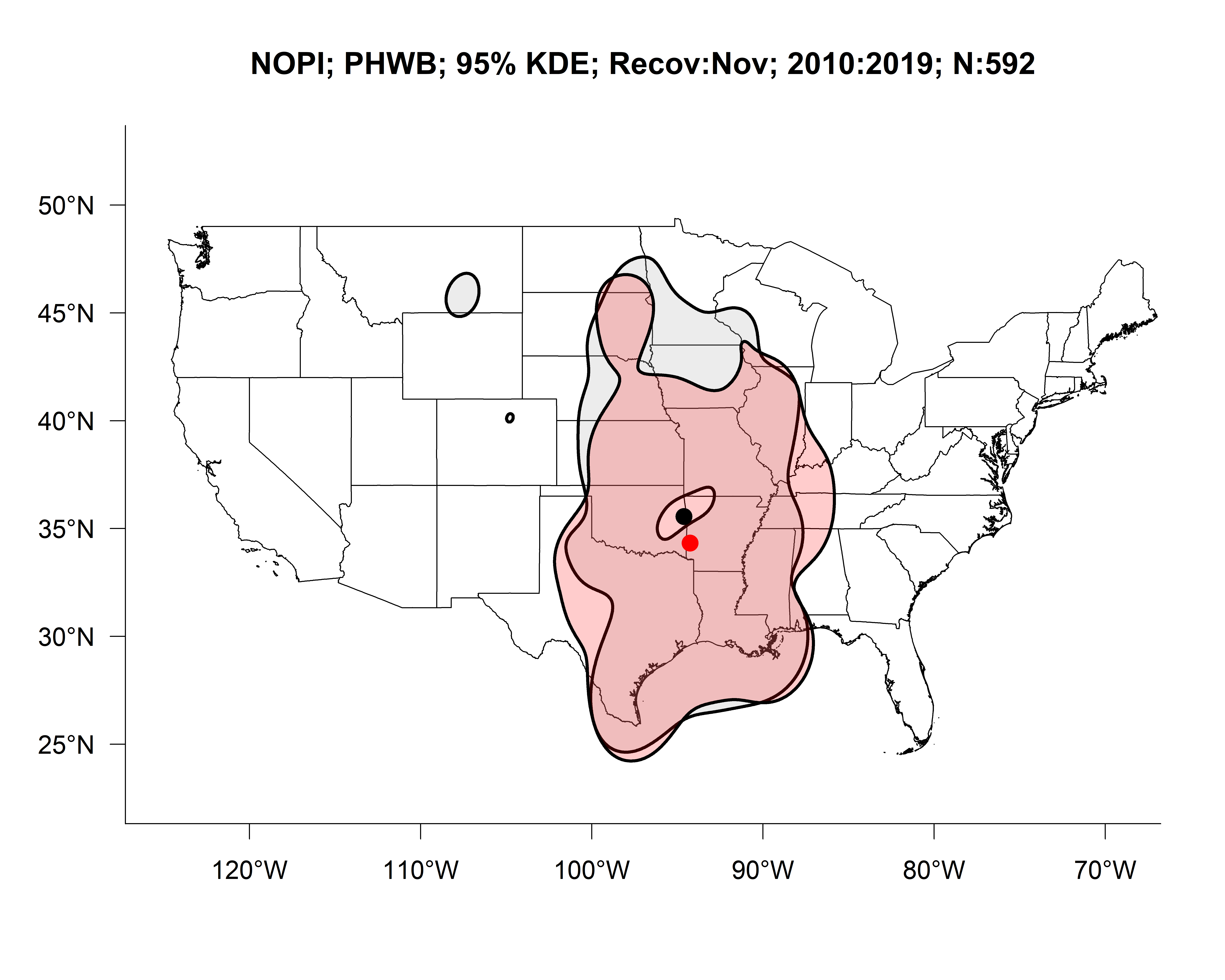

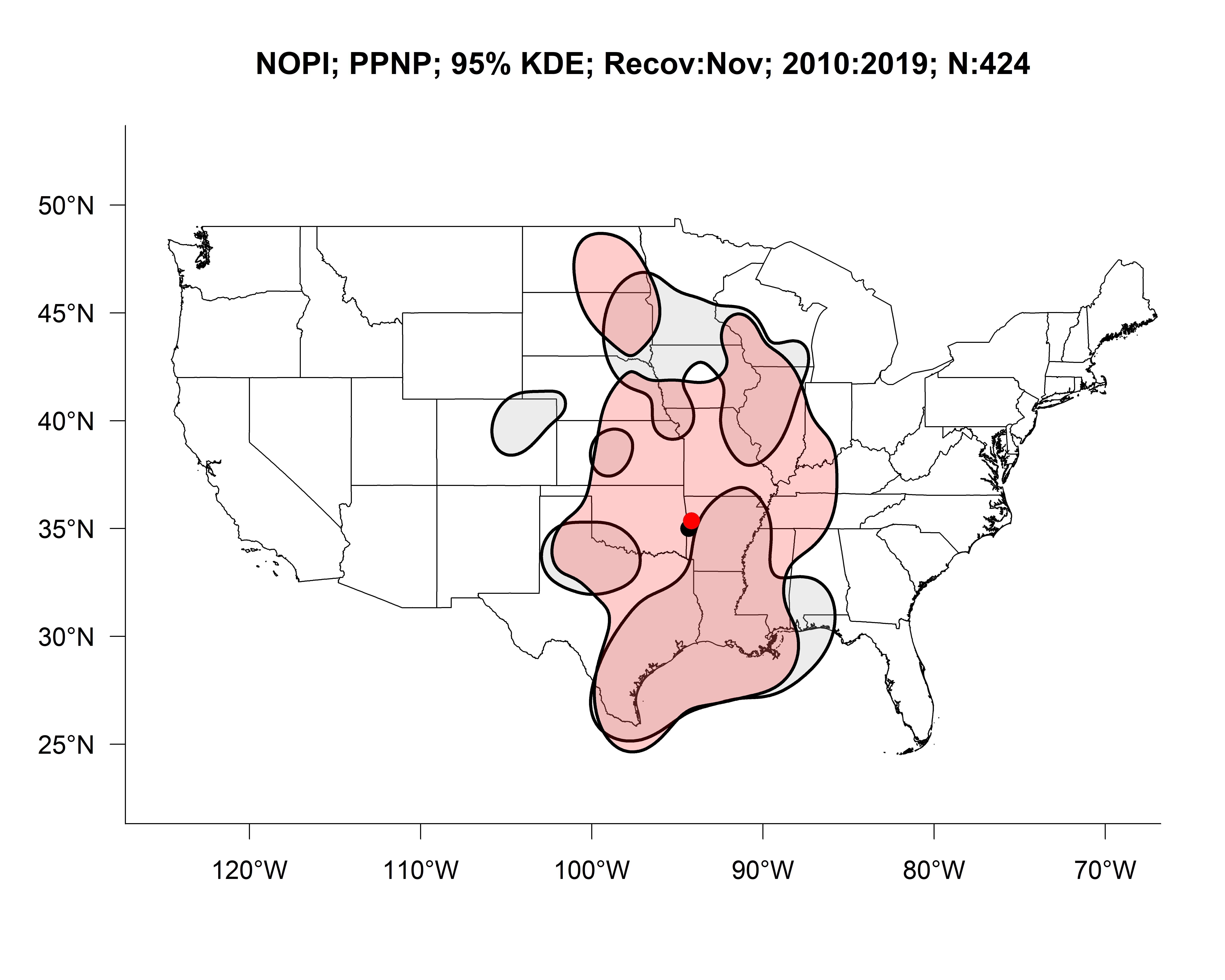

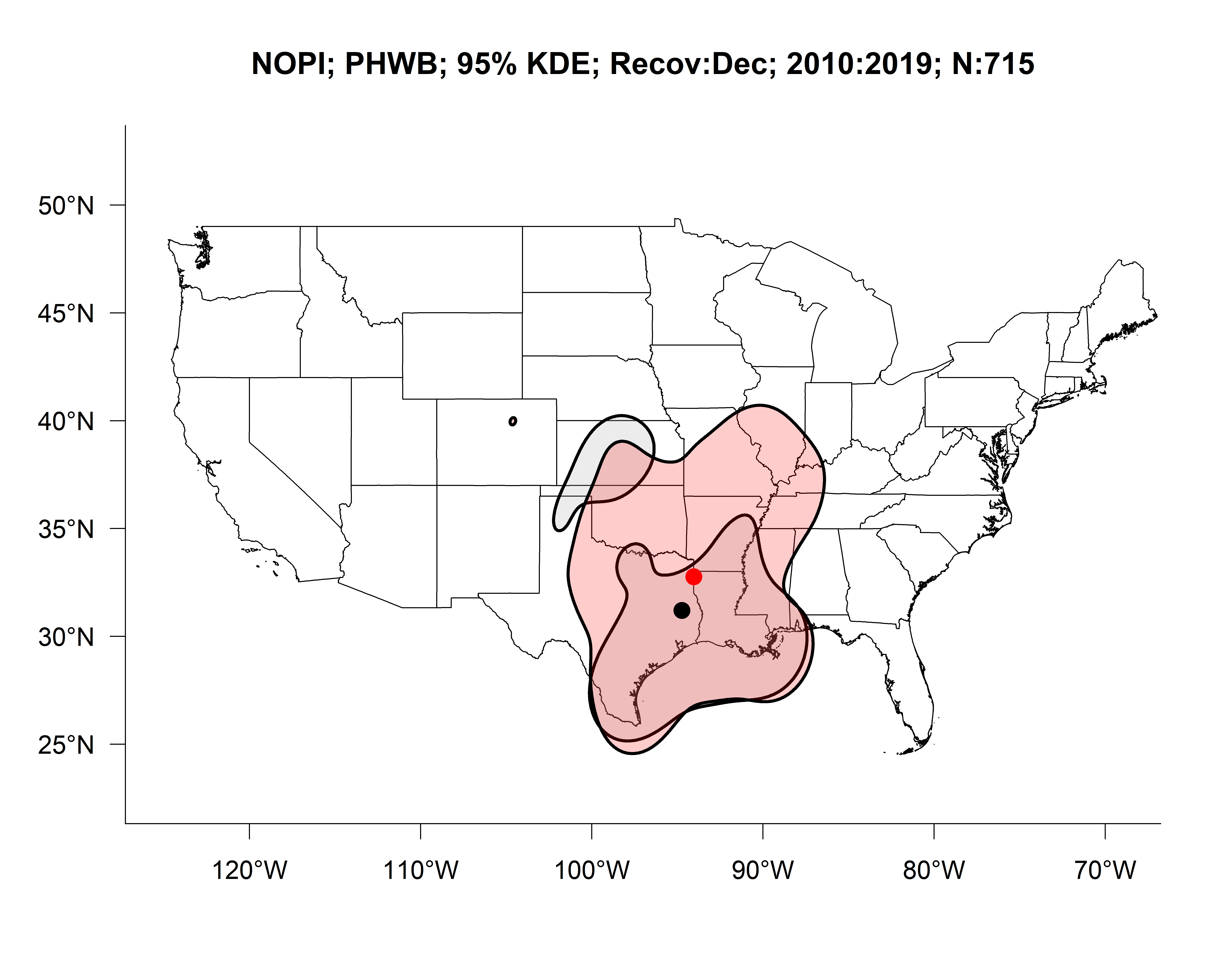

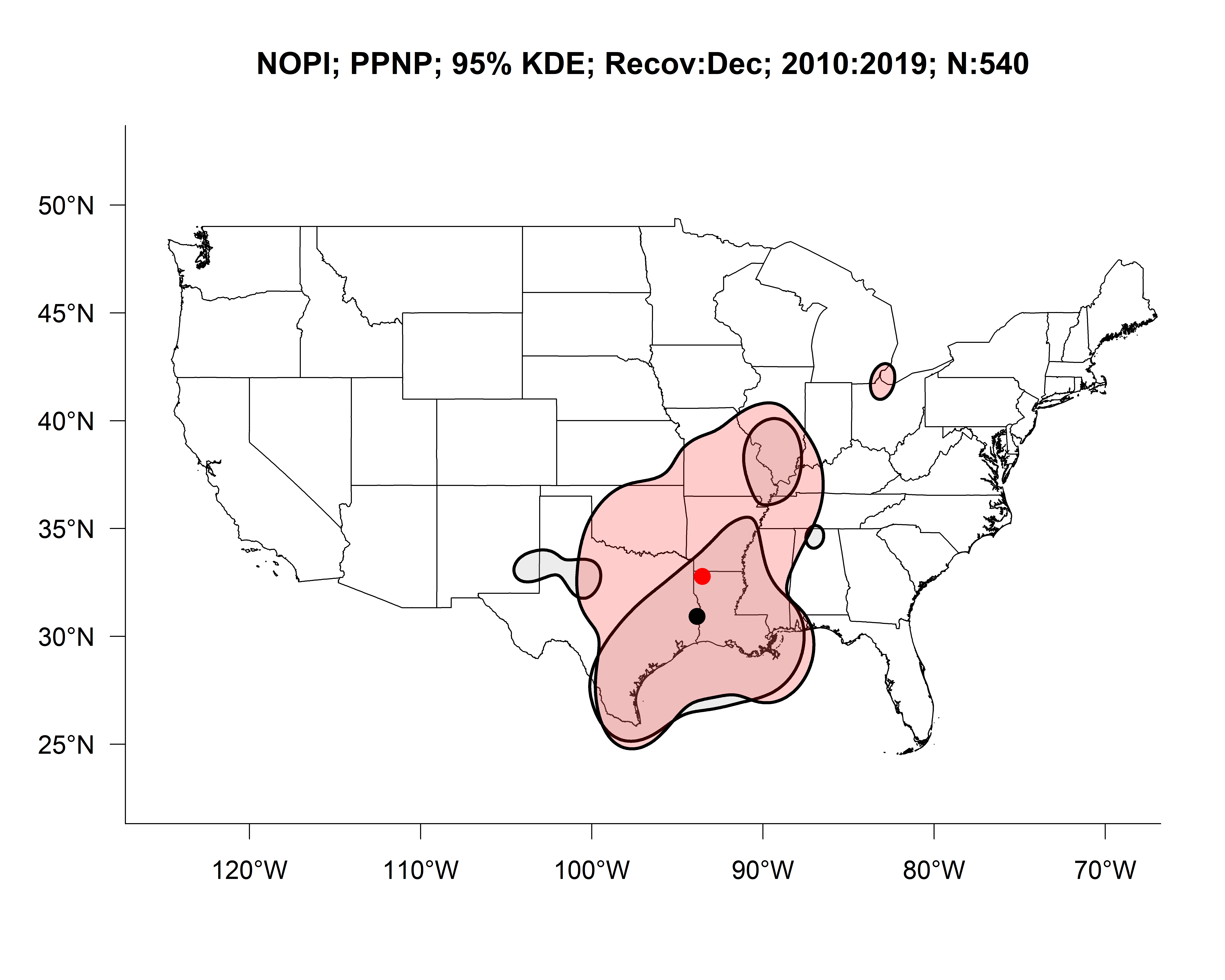

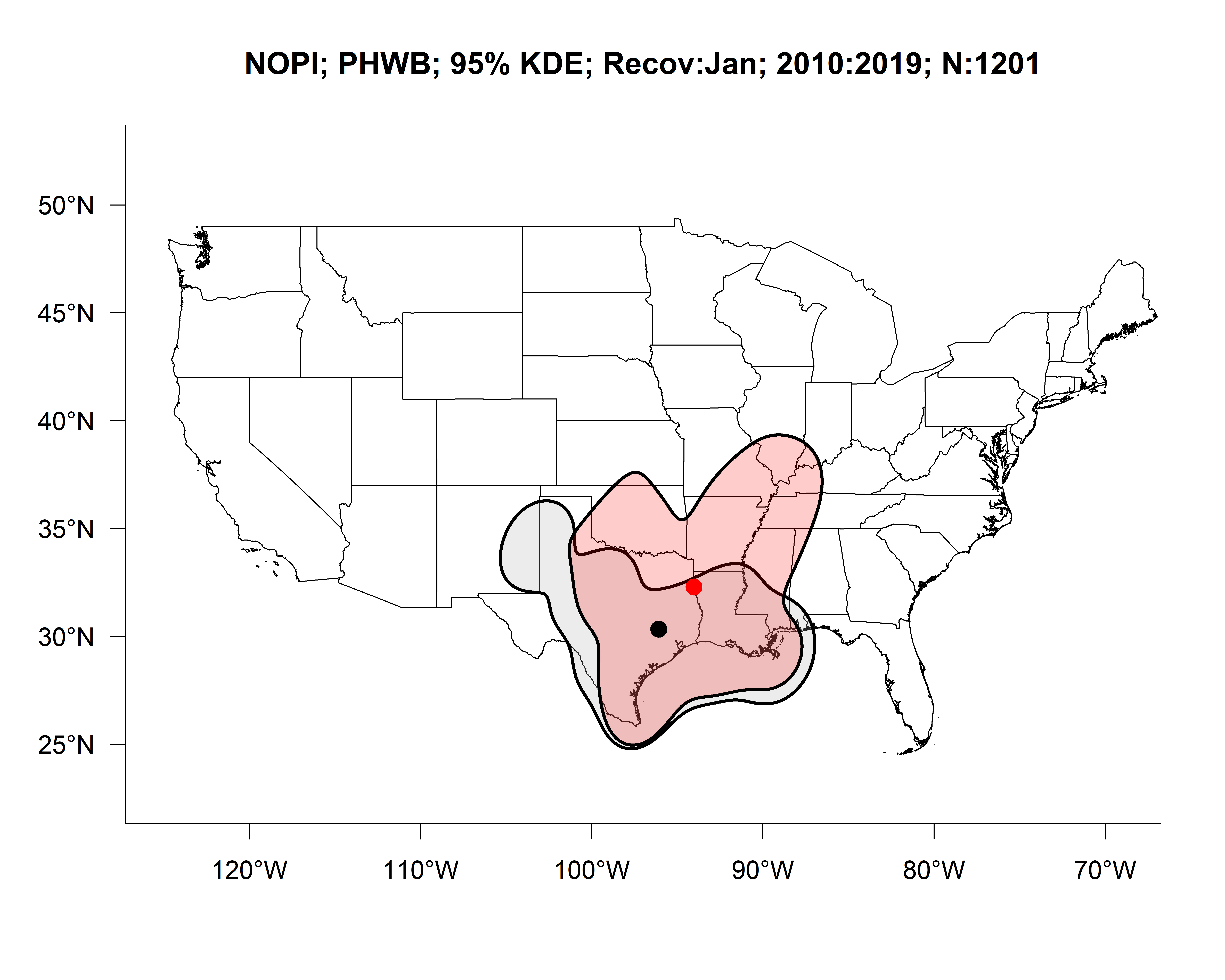

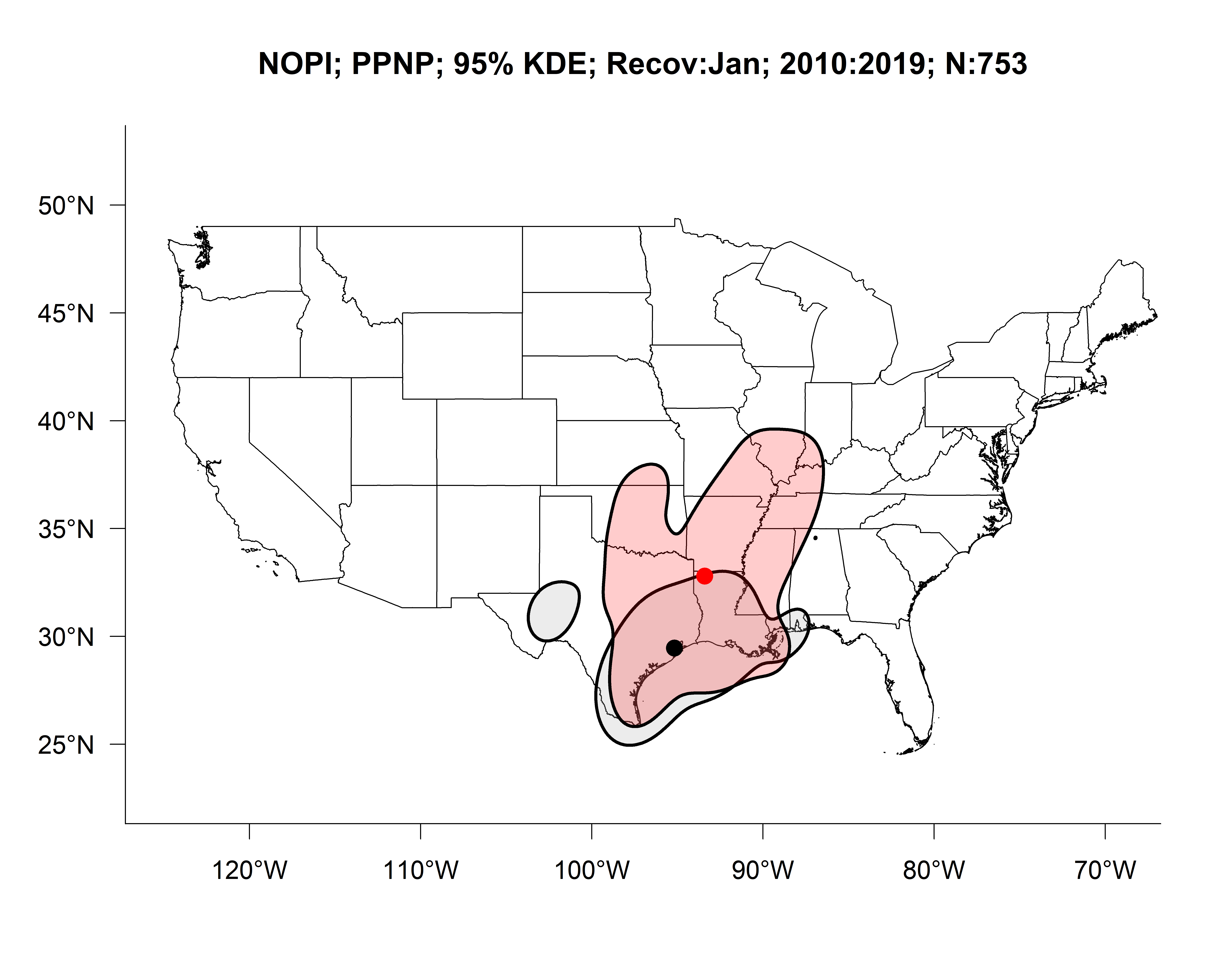


**Supplemental Figure 3.** Annual composition of banding region (% of bands) for blue-winged teal, mallards, and northern pintail bands recovered in the Central and Mississippi Flyways of North America during 1960–2019. Geographically defined banding regions include the Prairie Habitat and Western Boreal Area Joint Ventures (PHWB), the Prairie Pothole and Northern Great Plains Joint Ventures (PPNP), and the Upper Mississippi River / Great Lakes Joint Venture and Ontario (UMON).


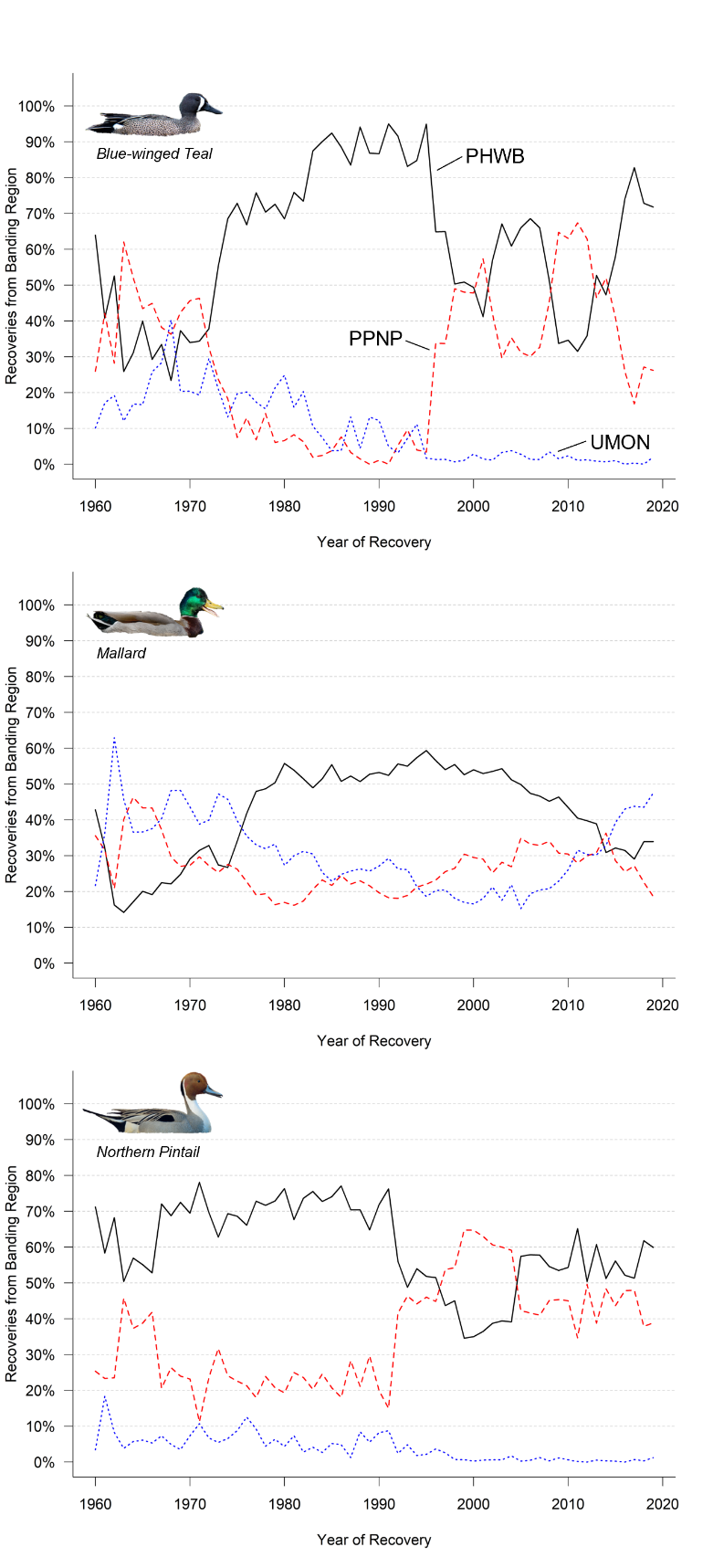

Supplement: Supplementary file 1 — Data S1: [file ECE3-14-e11331-s001.zip › Waterfowl_SpatiotemporalDynamics_KDE_2024-03-01_SupplementalMaterial.docx]
